# Supplementary material for: UPLC-ESI-MS/MS-Based Analysis of Various Edible Rosa Fruits Concerning Secondary Metabolites and Evaluation of Their Antioxidant Activities
Source: Foods. 2024 Mar 4;13(5):796. doi: 10.3390/foods13050796 (PMC10931279; doi:10.3390/foods13050796)
Supplement: Supplementary file 1 [file foods-13-00796-s001.zip › supplementary information-r2.pdf]

Table S1. List of metabolites identified from RRT-F, RSS-F, RLM-F, RDP-F and RSL-F. (The letter "a, b, c, d" represent four biological replicates)

Index	Ionization model	Q1 (Da)	Q3 (Da)	Molecular Weight (Da)	Formulas	Compounds	Class	Database	CAS	RRT-Fa	RRT-Fb	RRT-Fc	RRT-Fd	Proportion of peak area(%)	RSS-Fa	RSS-Fb	RSS-Fc	RSS-Fd	Proportion of peak area(%)	RLM-Fa	RLM-Fb	RLM-Fc	RLM-Fd	Proportion of peak area(%)	RDP-Fa	RDP-Fb	RDP-Fc	RDP-Fd	Proportion of peak area(%)	RSL-Fa	RSL-Fb	RSL-Fc	RSL-Fd	Proportion of peak area(%)	cpd_ID	kegg_map
km0071	[M+H]+	465.10	303.00	464.10	C21H20O12	Quercetin 3-O-galactoside (Hyperin)	Flavonoids	BioDeep	482-36-0	6720000	7220000	7730000	7340000	1.1976±0.7421	1970000	1760000	1840000	1950000	0.5085±0.2274	2110000	2180000	1980000	1990000	0.5021±0.2614	5030000	5200000	5230000	5300000	0.7826±0.2345	2260000	2490000	2650000	2340000	0.5057±0.2333	C10073	--
km0080	[M-H]-	283.00	268.00	284.07	C16H12O5	Biochanin A	Flavonoids	BioDeep	491-80-5	1080	1570	1480	1760	0.0002±0.0005	4890	4430	5300	4940	0.0013±0.0008	1710	1580	1580	1710	0.0004±0.0001	31300	30200	29600	32200	0.0046±0.0006	2060	1660	1650	2130	0.0004±0.0006	C00814	ko00943,ko01100,ko01110
km0081	[M+H]+	271.10	153.00	270.05	C15H10O5	Genistein (4',5,7-Trihydroxyisoflavone)	Flavonoids	BioDeep	446-72-0	13300	16300	16300	16300	0.0026±0.0022	8070	5540	6750	6010	0.0018±0.003	18100	19300	16100	16100	0.0042±0.004	35300	51000	41000	48000	0.0066±0.0105	40700	44300	51300	35100	0.0089±0.0117	C06563	ko00943,ko01100,ko01110
km0098	[M+H]+	287.00	153.00	286.05	C15H10O6	Orobol (5,7,3',4'-tetrahydroxyisoflavone)	Flavonoids	BioDeep	480-23-9	348000	507000	671000	377000	0.0078±0.0294	610000	651000	540000	380000	0.0148±0.0342	84600	80700	101000	116000	0.0232±0.039	291000	377000	212000	311000	0.0449±0.1028	193000	187000	139000	115000	0.033±0.0799	C10510	--
km0228	[M+H]+	465.00	303.00	464.10	C21H20O12	Quercetin 3-O-glucoside (Isotrifolin)	Flavonoids	BioDee	21637-25-2	636000	655000	672000	654000	1.081±0.4342	1800000	1550000	1840000	1840000	0.4751±0.3111	2010000	19400	1990000	2020000	0.4838±0.0496	4880000	4480000	4750000	5100000	0.7236±0.2427	2220000	2360000	2410000	2360000	0.4859±0.1399	C05623	ko00944,ko01100,ko01110

								p		00	00	00	00								00															
km0231	[M+H]+	287.00	153.00	286.05	C15H10O6	2'-Hydroxygenistein	Flavonoids	BioDeep	1156-78-1	46700	57700	41500	30800	0.0073±0.0219	56600	30000	44600	31300	0.011±0.0338	88900	63500	124000	65200	0.0208±0.0692	288000	313000	248000	229000	0.0407±0.0644	155000	109000	90700	142000	0.0259±0.0673	C12134	ko00943
km0235	[M+H]+	271.10	153.00	270.05	C15H10O5	Apigenin	Flavonoids	BioDeep	-	15400	15600	12700	16800	0.0025±0.0037	72400	63600	73600	56900	0.0018±0.0021	12100	13700	15900	14100	0.0034±0.0041	40300	44400	38100	36500	0.006±0.0063	38900	4380	3150	3490	0.0078±0.0117	C01477	ko00941,ko00943,ko00944,ko01100,ko01110
km0239	[M+H]+	301.10	286.00	300.06	C16H12O6	Chrysoeriol	Flavonoids	BioDeep	-	13600	14400	17400	14900	0.0025±0.0029	7260	6890	8430	9210	0.0021±0.0026	7900	9180	8010	8130	0.002±0.0017	27400	26600	26700	27300	0.0041±0.0008	4120	3840	4910	3090	0.0008±0.0014	C04293	ko00944
km0258	[M-H]-	263.00	219.00	264.14	C15H20O4	(+)-cis,trans-Abscisic acid (ABA)	Others	BioDeep	21293-29-8	1320000	1440000	1290000	1310000	0.2213±0.1045	907000	918000	909000	971000	0.2506±0.0721	608000	575000	582000	591000	0.1432±0.0135	587000	587000	561000	571000	0.0869±0.0322	677000	670000	723000	669000	0.1423±0.0263	C06082	ko00906,ko01100,ko01110,ko04075
km0329	[M-H]-	165.10	119.00	166.06	C9H10O3	3-(4-Hydroxyphenyl)propionic acid	Phenolic acids	BioDeep	501-97-3	7460000	7940000	8010000	7870000	0.1292±0.0355	215000	179000	223000	226000	0.057±0.0505	32900	38600	39100	38500	0.0091±0.0082	303000	295000	296000	342000	0.0465±0.0198	309000	343000	314000	290000	0.0653±0.041	C01744	--
km0436	[M+H]+	275.00	139.00	274.08	C15H14O5	Afzelechin (3,5,7,4'-Tetrahydroxyflavan)	Flavonoids	BioDeep	2545-00-8	412000	387000	408000	431000	0.0677±0.059	251000	247000	256000	256000	0.0683±0.0018	583000	602000	561000	589000	0.1419±0.0495	841000	816000	874000	912000	0.1297±0.0449	6340	8200	6190	6500	0.0014±0.002	C09320	ko00941,ko01110
km0532	[M+H]+	465.	303.	465.10	C21H21O12	Delphinidin 3-O-glucoside	Flavonoids	BioD	-	921	136	111	120	0.1888±0.2893	111000	118000	141000	82200	0.0306±0.0659	46500	574	37000	23000	0.01±0.0362	262000	299000	162000	227000	0.0358±0.0896	33300	47900	82300	87300	0.013±0.0544	C12138	ko00942

		10	00			(Mirtillin)		eep		000	0000	0000	0000								00															
km0548	[M-H]-	303.10	125.00	304.06	C15H12O7	Dihydroquercetin (Taxifolin)	Flavonoids	BioDeep	480-18-2	3460000	3500000	3510000	3710000	0.0586±0.0311	98400	121000	102000	97400	0.0284±0.0344	888000	882000	847000	888000	0.213±0.0435	920000	914000	977000	962000	0.1422±0.0572	52900	41600	48800	41200	0.0096±0.0118	C01617	ko00941,ko01100,ko01110
km0573	[M-H]-	441.30	169.00	442.30	C22H18O10	Epicatechin gallate (ECG)	Flavonoids	BioDeep	1257-08-5	17100	35600	33300	32400	0.0049±0.0158	35300	26900	24700	18700	0.0072±0.0191	74800	56200	76200	66000	0.0166±0.0214	318000	322000	311000	338000	0.0486±0.0071	10400	16400	11300	18000	0.0029±0.0082	-	-
km0602	[M+H]+	433.00	271.00	432.11	C21H20O10	Genistein 7-O-Glucoside (Genistin)	Flavonoids	BioDeep	529-59-9	27200	26700	41000	32900	0.0053±0.0139	86200	65600	74900	50300	0.0187±0.0416	319000	292000	310000	278000	0.0729±0.042	80200	66300	66300	73500	0.0108±0.09	24500	31800	17000	17300	0.0047±0.0147	C09126	ko00943
km0640	[M+H]+	611.20	303.00	610.19	C28H34O15	Hesperetin 7-rutinoside (Hesperidin)	Flavonoids	BioDeep	520-26-3	253000	361000	294000	290000	0.0493±0.0693	90300	90500	84600	99600	0.0247±0.0162	-	-	-	-	-	-	-	-	-	-	-	-	-	-	-	C09755	--
km0683	[M+H]+	449.00	287.00	448.10	C21H20O11	Kaempferol 3-O-galactoside (Trifolin)	Flavonoids	BioDeep	23627-87-4	7340000	7590000	7110000	7780000	1.2316±0.6493	3190000	3000000	3310000	3090000	0.8514±0.2946	6300000	5410000	6220000	6280000	1.4707±0.8998	11600000	11600000	12200000	12700000	1.8125±0.594	7390000	8950000	8740000	8360000	1.7368±1.2489	C12626	ko00944
km0686	[M+H]+	433.00	287.00	432.11	C21H20O10	Kaempferol 3-O-rhamnoside (Kaempferin)	Flavonoids	BioDeep	482-39-3	156000	150000	168000	149000	0.0258±0.0248	54600	68600	65200	67200	0.0173±0.0177	52100	41700	45200	43900	0.0111±0.0094	47100	45400	48400	46800	0.0071±0.0029	40800	46200	38200	35400	0.0083±0.0094	C16911	ko00944
km	[M	4	2	44	C21	Luteolin 7-	Flav	Bi	-	4	4	4	5	0.814	20	20	23	22	0.589	429	3	43	438	1.0046	924	888	8370	937	1.350	5810	59	62	62	1.2578	-	-

0779	+H]+	49.00	87.10	8.00	H20O11	O-glucoside	onoids	oDeep		150000	990000	900000	720000	5±1.1365	60000	30000	70000	60000	4±0.3624	0000	550000	20000	0000	±0.8673	0000	0000	000	0000	9±0.428	000	90000	00000	00000	±0.4244		
km0814	[M-H]-	301.00	151.00	302.04	C15H10O7	Morin	Flavonoids	BioDeep	6472-38-4	1930000	1990000	1890000	2010000	0.323±0.1408	5270000	5170000	5240000	5350000	0.1422±0.0131	541000	527000	506000	576000	0.1306±0.0634	1970000	1820000	1900000	2030000	0.2908±0.0793	1960000	1910000	1810000	1880000	0.3932±0.2152	C10105	--
km0818	[M+H]+	465.10	319.00	464.10	C21H20O12	Myricetin 3-O-rhamnoside (Myricitrin)	Flavonoids	BioDeep	17912-87-7	2760000	1910000	2160000	2200000	0.0375±0.0935	744000	683000	576000	562000	0.0174±0.0256	25600	18100	21200	20700	0.0052±0.0068	-	-	-	-	-	-	-	-	-	-	C10108	--
km0843	[M-H]-	271.10	151.00	272.07	C15H12O5	Naringenin	Flavonoids	BioDeep	480-41-1	2970000	3100000	2840000	2920000	0.0489±0.0251	2410000	2440000	2660000	2660000	0.0688±0.0283	174000	166000	168000	179000	0.0417±0.0104	645000	650000	621000	673000	0.0976±0.0161	175000	180000	175000	181000	0.037±0.0142	C00509	ko00941,ko00943,ko01100,ko01110
km0849	[M-H]-	271.10	151.00	272.07	C15H12O5	Naringenin chalcone	Flavonoids	BioDeep	25515-46-2	3350000	3270000	3170000	3180000	0.0536±0.0384	2870000	2830000	2840000	2970000	0.0778±0.0135	184000	190000	185000	189000	0.0455±0.0124	688000	667000	707000	745000	0.1058±0.0322	199000	192000	196000	201000	0.041±0.017	C06561	ko00941,ko01100,ko01110
km0864	[M+H]+	403.10	373.00	402.13	C21H22O8	Nobiletin	Flavonoids	BioDeep	3237-44-3	515000	422000	457000	413000	0.0075±0.0131	51500	53400	47900	46400	0.0135±0.0108	43700	48100	43000	46200	0.011±0.0067	49000	50000	45400	46900	0.0072±0.039	39400	45300	114000	46900	0.0126±0.0685	C10112	--
km0879	[M-H]-	155.00	111.00	156.02	C5H4N2O4	Orotic acid	Vitamins	BioDee	65-86-1	245000	259000	244000	291000	0.0429±0.0421	6490000	5270000	7390000	7110000	0.1773±0.2292	260000	256000	249000	246000	0.0614±0.016	432000	454000	438000	449000	0.0668±0.0222	1580000	1580000	1350000	1270000	0.3005±0.3458	C00295	ko00240,ko01100

								p		0	0	0	0								0															
km0934	[M-H]-	463.00	301.00	464.10	C21H20O12	Quercetin 4'-O-glucoside (Spiraeoside)	Flavonoids	BioDeep	20229-56-5	258000	320000	280000	277000	0.0468±0.0325	1160000	1360000	1540000	1790000	0.0395±0.0682	218000	182000	200000	202000	0.0487±0.029	1200000	1250000	1190000	1250000	0.1843±0.0513	8680000	9320000	8300000	7820000	1.7733±1.3076	-	-
km0936	[M-H]-	477.10	301.00	478.36	C21H18O13	Quercetin 7-O-β-D-Glucuronide	Flavonoids	BioDeep	-	-	-	-	-	-	-	-	-	-	-	15100000	16300000	14900000	16000000	3.7876±1.9632	4890000	4840000	5200000	5300000	0.7624±0.2964	2280000	2170000	2180000	2080000	0.4528±0.1973	-	-
km0981	[M+H]+	373.10	343.00	372.12	C20H20O7	Tangeretin	Flavonoids	BioDeep	-	272000	307000	297000	277000	0.0048±0.002	35800	36500	37900	34600	0.0098±0.0042	32300	27000	23900	23800	0.0065±0.0091	45800	41400	39300	41700	0.0063±0.0041	31600	28100	54800	30700	0.0075±0.0236	C10190	--
km0997	[M+H]+	347.30	285.00	346.25	C22H34O3	Troxerutin (Trihydroxyethyl rutin)	Flavonoids	BioDeep	7085-55-4	1310000	1060000	1270000	1220000	0.0201±0.0315	1370000	1210000	1230000	1450000	0.0356±0.0282	86300	139000	165000	114000	0.0307±0.0856	102000	179000	107000	109000	0.0188±0.058	110000	119000	231000	69000	0.0272±0.1348	-	-
km1113	[M+H]+	321.10	147.30	320.10	C16H16O7	5-O-p-Coumaroyl shikimic acid	Flavonoids	BioDeep	-	992000	101000	109000	111000	0.0174±0.0128	17000	23600	25700	20900	0.0059±0.0102	16900	15200	10200	14000	0.0034±0.0067	33000	21300	31700	33400	0.0045±0.0082	12600	5370	6470	7740	0.0017±0.0069	-	-
km1211	[M+H]+	463.20	301.10	462.20	C22H22O11	Chrysoeriol 7-O-hexoside	Flavonoids	BioDeep	-	165000	139000	145000	128000	0.0239±0.0426	1110000	96000	96600	92100	0.0268±0.0237	69700	76700	52400	76000	0.0167±0.0275	175000	184000	193000	174000	0.0274±0.0203	33200	2940	5080	2090	0.0069±0.0241	-	-
km1269	[M-H]-	329.9.	229.9.	330.10	C17H14O7	Di-O-methylquercetin	Flavonoids	BioD	-	508	530	473	466	0.0817±0.0673	1430000	1460000	1440000	1460000	0.0392±0.0082	218000	199	211000	216000	0.0513±0.0143	1420000	1400000	1360000	1450000	0.2122±0.0269	1080000	1090000	1140000	1060000	0.227±0.0155	-	-

		10	10					eep		000	000	000	000								000										0	0	0			
km1293	[M-H]-	549.20	387.10	550.20	C25H26O14	Hesperetin O-malonylhexoside	Flavonoids	BioDeep	-	381000	450000	401000	394000	0.0671±0.0351	1130000	1270000	1300000	1360000	0.0342±0.0246	10300	11200	12400	10500	0.0027±0.0026	186000	208000	189000	200000	0.0295±0.0167	121000	141000	127000	125000	0.0267±0.0174	-	-
km1300	[M+H]+	195.10	145.30	194.10	C10H10O4	Hydroxy-methoxycinnamate	Phenolic acids	BioDeep	-	467000	560000	422000	459000	0.0079±0.01	452000	472000	455000	498000	0.0127±0.0056	-	-	-	-	-	132000	111000	122000	129000	0.0186±0.0119	118000	124000	120000	113000	0.0247±0.0077	-	-
km1307	[M+H]+	479.20	317.20	478.20	C22H22O12	Isorhamnetin 5-O-hexoside	Flavonoids	BioDeep	-	1020000	1280000	1170000	1100000	0.1883±0.1456	3980000	3320000	3230000	3870000	0.0974±0.0996	1250000	1340000	1300000	1430000	0.3234±0.2005	1240000	1350000	1390000	1430000	0.2039±0.1178	107000	101000	114000	102000	0.022±0.0089	-	-
km1405	[M+H]+	286.00	124.00	285.00	C12H15NO7	Nicotinic acid-hexoside	Vitamins	BioDeep	-	204000	264000	244000	234000	0.039±0.034	93600	81100	90100	76000	0.0231±0.0221	360000	324000	336000	350000	0.0832±0.0259	490000	467000	519000	521000	0.0753±0.0357	34000	46600	32500	43200	0.0081±0.0152	-	-
km1437	[M+H]+	137.30	94.20	136.30	C7H7NO2	p-Aminobenzoate	Phenolic acids	BioDeep	-	138000	151000	139000	104000	0.022±0.0413	-	-	-	-	-	284000	285000	305000	308000	0.0718±0.0336	746000	736000	708000	774000	0.1117±0.0165	550000	585000	500000	524000	0.1123±0.0888	C00568	ko00790,ko01100
kz000028	[M+H]+	89.11	72.08	88.10	C4H12N2	Putrescine	Alkaloids	BioDeep	-	479000	522000	478000	506000	0.0082±0.0025	47500	58400	55300	43300	0.0138±0.0203	55200	45300	48200	47700	0.0119±0.0087	50600	38700	40000	52400	0.0068±0.009	46600	45600	51300	47200	0.0099±0.0036	C00134	ko00330,ko00472,ko00480,ko00960,ko01100,ko01110,ko02010
kz00	[M+H]	29	16	292.1	C14H20	Caffeoylagmatine	Alkaloid	Bio	-	29	33	28	30	0.005±0.00	1240	1130	1500	9310	0.0032±0.0	-	-	-	-	-	99200	105000	113000	107000	0.016±0.01	-	-	-	-	-	-	-

0032	+	3.16	3.04	5	N4O3		s	Deep		200	600	200	600	34	0	0	0		063										07							
kz000040	[M+H]+	558.28	207.07	557.27	C29H39N3O8	N,N'-Bis(Sinapoyl)Spermidine	Alkaloids	BioDeep	-	2470000	2270000	2110000	2100000	0.037±0.0496	56900	54700	52900	48100	0.0144±0.0117	41700	28300	4530	50200	0.01±0.0222	138000	98000	55700	114000	0.0152±0.0506	7490	5560	5670	3060	0.0011±0.0038	C21170	--
kz000047	[M-H]-	151.00	107.00	152.05	C8H8O3	Mandelic acid	Phenolic acids	BioDeep	-	2720000	2810000	3050000	2930000	0.0475±0.031	-	-	-	-	-	55800	44400	38400	34100	0.0105±0.0219	150000	210000	178000	193000	0.0276±0.0392	126000	124000	122000	137000	0.0265±0.02	C01984	--
kz000049	[M-H]-	164.04	120.00	165.04	C8H7NO3	2-(Formylamino)benzoic acid	Phenolic acids	BioDeep	-	10800000	11900000	11800000	12100000	0.1923±0.0669	1850000	1660000	1810000	2100000	0.0501±0.0431	304000	301000	320000	324000	0.0759±0.0294	632000	643000	645000	624000	0.096±0.0419	782000	793000	817000	753000	0.1634±0.0208	C05653	ko00380,ko01100
kz000050	[M-H]-	167.10	123.00	168.04	C8H8O4	Homogentisic acid	Phenolic acids	BioDeep	-	206000	679000	407000	575000	0.0076±0.0396	1120000	1310000	1080000	1310000	0.0326±0.0352	60100	42600	66500	62700	0.0141±0.025	95400	105000	96400	96000	0.0148±0.0095	-	-	-	-	-	C00544	ko00130,ko00350,ko01100
kz000051	[M-H]-	177.00	145.00	178.06	C10H10O3	4-Methoxycinnamic acid	Phenolic acids	BioDeep	-	1800000	2010000	1870000	1890000	0.0312±0.048	1070000	99500	99800	97600	0.0273±0.0128	166000	158000	159000	167000	0.0395±0.0065	338000	326000	325000	350000	0.0504±0.045	242000	247000	264000	254000	0.0523±0.0147	-	-
kz000052	[M-H]-	181.06	108.00	182.06	C9H10O4	Ethyl 3,4-Dihydroxybenzoate (Ethyl protocatechuate)	Phenolic acids	BioDeep	-	564000	665000	572000	584000	0.0098±0.059	11100	7340	6230	7500	0.0022±0.0058	4810	5990	5430	5920	0.0013±0.0015	383000	402000	385000	426000	0.0601±0.0189	11500	1480	1820	1570	0.0031±0.0052	-	-
kz	[M	1	1	19	C10	Isoferulic	Phe	Bi	-	5	6	6	6	0.104	17	18	18	17	0.048	461	4	44	487	0.1114	331	336	3470	345	0.051	8420	80	84	77	0.1696	C1	--

000053	-H]-	93.06	34.00	4.06	H10O4	Acid	nolic acids	oDeep		59000	59000	38000	68000	1±0.0649	8000	6000	2000	3000	6±0.0216	000	41000	5000	000	±0.0423	000	000	00	000	2±0.0172	00	4000	5000	3000	±0.0582	0470	
kz000055	[M-H]-	237.08	103.00	238.08	C12H14O5	3,4,5-Trimethoxycinnamic acid	Phenolic acids	BioDeep	7400-08-0	55800	63200	55700	56500	0.0095±0.0046	-	-	-	-	-	2760	2680	3090	2790	0.0007±0.0005	6070	5540	4620	5060	0.0008±0.001	31300	32800	33700	32800	0.0068±0.0015	-	-
kz000056	[M-H]-	299.10	137.10	300.08	C13H16O8	Salicylic acid glucoside	Phenolic acids	BioDeep	-	194000	202000	189000	186000	0.0318±0.0171	179000	177000	170000	186000	0.0482±0.0182	459000	464000	432000	462000	0.1104±0.0368	368000	321000	359000	362000	0.0531±0.0297	68400	70900	68700	71400	0.0145±0.0059	-	-
kz000058	[M-H]-	315.10	153.10	316.08	C13H16O9	Protocatechuic acid O-glucoside	Phenolic acids	BioDeep	137887-25-3	1010000	914000	970000	883000	0.1563±0.1846	1130000	1110000	1080000	1190000	0.305±0.1178	771000	842000	803000	876000	0.2002±0.1249	673000	712000	601000	642000	0.0991±0.079	38100	53100	50300	54500	0.0102±0.0156	-	-
kz000059	[M-H]-	319.00	145.10	320.09	C16H16O7	3-O-p-Coumaroylshikimic acid	Phenolic acids	BioDeep	-	56100	61900	48800	52800	0.0091±0.0106	168000	149000	170000	159000	0.0437±0.0221	-	-	-	-	-	38500	47600	37900	43600	0.0063±0.007	7470	12800	12800	10000	0.0022±0.0049	-	-
kz000064	[M+H]+	369.10	177.10	368.11	C17H20O9	1-O-Feruloyl quinic acid	Phenolic acids	BioDeep	-	40800	55700	45500	55400	0.0081±0.0121	166000	147000	170000	149000	0.0427±0.0296	-	-	-	-	-	19900	25500	20000	14800	0.003±0.0072	45600	58500	45000	49600	0.0103±0.0134	-	-
kz000066	[M+H]+	375.20	137.10	374.10	C19H18O8	Feruloyl syringic acid	Phenolic acids	BioDeep	-	50600	51800	46900	43200	0.008±0.009	4200	7100	11000	9640	0.0022±0.0079	118000	134000	126000	120000	0.0303±0.0219	26100	41400	43000	24100	0.0051±0.0162	22600	31100	24400	19600	0.0051±0.0097	-	-

kz000068	[M-H]-	441.30	169.00	442.09	C22H18O10	Catechin gallate	Phenolic acids	BioDeep	-	377000	473000	321000	354000	0.0063±0.0118	30500	37400	39900	24600	0.009±0.0192	58800	678000	65600	63700	0.0156±0.0116	335000	315000	306000	345000	0.049±0.0151	12400	9950	14900	13400	0.0026±0.0041	-	-
kz000070	[M-H]-	460.10	117.90	461.15	C19H27NO12	Anthranilate O-hexosyl-O-hexoside	Phenolic acids	BioDeep	-	5210000	5100000	4170000	5080000	0.0808±0.1122	5780000	7460000	7170000	5810000	0.1775±0.256	169000	1520000	170000	168000	0.04±0.0176	821000	819000	826000	832000	0.1243±0.0302	291000	284000	300000	250000	0.0584±0.0361	-	-
kz000078	[M-H]-	477.14	179.03	478.15	C23H26O11	1'-O-β-D-(3,4-Dihydroxyphenethyl)-O-caffeoyl-glucoside	Phenolic acids	BioDeep	-	-	-	-	-	-	-	-	-	-	-	952000	922000	887000	851000	0.2195±0.1035	261000	261000	330000	300000	0.0434±0.053	128000	122000	142000	131000	0.0272±0.0141	-	-
kz000118	[M+H]+	287.08	167.00	286.08	C16H14O5	Sakuranetin	Flavonoids	BioDeep	-	2990	3560	3700	3950	0.0006±0.0007	35500	37800	34600	35800	0.0097±0.0051	-	-	-	-	-	13100	11800	11500	11600	0.0018±0.0012	6450	6670	6850	5680	0.0013±0.0008	C09833	ko00941,ko01110
kz000121	[M+H]+	417.10	255.10	416.11	C21H20O9	Chrysin 5-O-glucoside (Toringin)	Flavonoids	BioDeep	-	544000	614000	532000	509000	0.0091±0.0077	69300	63400	66200	75300	0.0185±0.0118	37800	41700	39700	34500	0.0093±0.0085	157000	160000	147000	169000	0.0238±0.0086	25000	24800	25900	24500	0.0052±0.0006	-	-
kz000145	[M+H]+	269.09	137.04	268.07	C16H12O4	Formononetin	Flavonoids	BioDeep	-	1470000	1560000	1460000	1540000	0.0249±0.0079	320000	323000	324000	336000	0.0881±0.0157	29600	31000	29300	35000	0.0076±0.0063	136000	124000	124000	131000	0.0194±0.0074	2800000	287000	273000	269000	0.5765±0.214	-	-
kz000147	[M+H]+	479.00	317.10	478.11	C22H22O12	Methylquercetin O-hexoside	Flavonoids	BioDee	-	751000	858000	742000	776000	0.129±0.0681	2630000	2540000	2960000	2840000	0.0741±0.0416	1000000	89400	860000	990000	0.2274±0.1401	1030000	858000	859000	983000	0.1405±0.1104	84900	79500	65400	67800	0.0155±0.0213	-	-

								p		0	0	0	0								0															
kz000156	[M+H]+	303.08	167.00	302.08	C16H14O6	7-O-Methylerioodictyol	Flavonoids	BioDeep	51857-11-5	3090	3410	3390	2430	0.0005±0.0009	49800	45000	49600	51400	0.0132±0.0056	-	-	-	-	-	5830	4790	4820	3760	0.0007±0.0014	-	-	-	-	-	-	-
kz000159	[M-H]-	433.12	271.00	434.12	C21H22O10	Naringenin 7-O-glucoside (Prunin)	Flavonoids	BioDeep	529-55-5	632000	635000	620000	713000	0.1074±0.0944	135000000	147000000	143000000	145000000	0.3856±0.1649	775000	703000	737000	822000	0.1845±0.1057	2780000	2680000	2820000	2680000	0.4134±0.1977	412000	435000	454000	425000	0.0897±0.0183	C09099	ko00941
kz000173	[M+H]+	96.04	68.05	95.04	C5H5NO	3-Hydroxypyridine	Others	BioDeep	109-00-2	-	-	-	-	-	-	-	-	-	-	-	-	-	-	-	38400	37600	34000	41900	0.0057±0.0034	44400	38100	33200	40100	0.0081±0.0115	-	-
kz000179	[M+H]+	140.00	99.00	139.03	C6H5NO3	4-Nitrophenol	Others	BioDeep	100-02-7	8650000	6540000	6720000	7070000	1.2018±2.5905	613000000	704000000	631000000	557000000	1.6962±1.8949	4580000	4140000	4430000	3380000	1.0047±1.2895	4800000	3540000	3670000	4950000	0.6374±0.9601	5350000	5300000	5510000	4750000	1.086±0.5142	C00870	--
kz000180	[M+H]+	142.10	124.09	141.09	C6H11N3O	Histidinol	Others	BioDeep	501-28-0	17000000	19700000	18900000	18000000	3.036±1.197	1430000000	1350000000	1430000000	1490000000	3.854±1.0178	19500000	19300000	19400000	17500000	4.6018±2.5245	16900000	15400000	16100000	16700000	2.4532±0.8117	18200000	18300000	21100000	21100000	4.0897±3.3436	C00860	ko00340,ko01100,ko01110,ko01230
kz000183	[M+H]+	165.10	123.08	164.08	C10H12O2	Hinokitiol	Others	BioDeep	499-44-5	17000	16000	26700	17900	0.0032±0.0103	20800	19900	17600	19600	0.0053±0.0041	18800	14600	18700	17500	0.0042±0.0044	19400	22700	15900	21300	0.003±0.0043	19600	2530	7910	2230	0.0075±0.056	C09904	--
kz00	[M-	21	97.	4.0	C5H11O	2-Deoxyribo	Others	Bio	1721	40	46	40	50	0.0073±0.0	7610	6500	6860	6990	0.0189±0.0	-	-	-	-	-	118000	121000	128000	141000	0.0191±0.0	-	-	-	-	-	C006	ko00030,ko00240,ko01

0186	H]-	3.10	00	2	7P	se 1-phosphate		Deep	0-42-3	300	100	400	700	089	0	0	0	0	119										117						72	100
kz000190	[M-H]-	271.09	107.60	272.09	C12H16O7	Arbutin	Others	BioDeep	497-76-7	17800	18700	16500	14400	0.0028±0.004	12500	10200	11200	12500	0.0031±0.0027	143000	132000	136000	141000	0.0335±0.0072	92300	80100	85500	87200	0.013±0.0072	19200	15800	21400	15700	0.0037±0.0051	C06186	ko00010
kz000197	[M-H]-	405.13	243.06	406.13	C20H22O9	E-3,4,5'-Trihydroxy-3'-glucopyranosylstilbene	Others	BioDeep	-	3900	3890	5550	5210	0.0008±0.0018	2880	5220	3070	2630	0.0009±0.0034	-	-	-	-	-	38200	32100	37300	44500	0.0057±0.0062	16600	12900	23600	17700	0.0037±0.0085	-	-
kz000201	[M-H]-	181.07	71.01	182.08	C6H14O6	D-galacitol	Others	BioDeep	608-66-2	8190000	8490000	8330000	8260000	0.1374±0.0444	802000	782000	800000	833000	0.2176±0.0346	390000	392000	397000	398000	0.0959±0.019	2810000	2770000	2770000	2890000	0.4236±0.0394	2440000	2500000	2530000	2500000	0.5182±0.1043	-	-
kz000207	[M+H]+	481.10	319.05	480.09	C21H20O13	Cannabiscitrin	Others	BioDeep	520-14-9	-	-	-	-	-	-	-	-	-	-	-	-	-	-	-	98700	92000	101000	88600	0.0144±0.0119	-	-	-	-	-	-	-
kz000231	[M+H]+	132.10	86.10	131.06	C5H9NO3	5-Aminolevulinate	Alkaloids	BioDeep	106-60-5	3380000	3590000	3690000	3460000	0.0583±0.0252	899000	943000	938000	852000	0.2458±0.1407	400000	349000	362000	356000	0.0891±0.0441	1340000	1270000	1320000	1370000	0.1997±0.0399	1130000	1180000	2510000	1100000	0.3052±1.323	C00430	ko00260,ko00860,ko01100,ko01110,ko02010
kz000232	[M]+	184.00	125.00	184.07	C5H15NO4P+	O-Phosphocholine	Alkaloids	BioDeep	107-73-3	3680000	3660000	3540000	3690000	0.0602±0.0342	1200000	1250000	1150000	1220000	0.3261±0.1542	483000	506000	522000	464000	0.1201±0.0756	369000	363000	339000	413000	0.0559±0.0304	361000	344000	320000	347000	0.0714±0.0521	C00588	ko00564,ko01100
kz00	[M+H]	38	22	381.1	C16H23	Trans-Zeatin-9-	Alkaloid	Bio	-	10	11	10	11	0.0177±0.0	1220	1080	1260	1150	0.0318±0.0	252000	22	2270	245000	0.0578±0.022	574000	541000	627000	682000	0.0913±0.0	74500	7440	7640	7330	0.0155±0.001	-	-

0240	+	2.20	0.20	6	N5O6	N-Glucoside	s	Deep		1000	6000	0000	2000	108	00	00	00	00	186		7000	00		5					765		0	0	0	9		
kz000258	[M+H]+	256.20	124.10	256.08	C11H14NO6+	Nicotinate D-ribonucleoside	Vitamins	BioDeep	17720-18-2	638000	643000	634000	719000	0.1088±0.09	233000	250000	279000	263000	0.0693±0.0472	709000	709000	659000	670000	0.167±0.0649	983000	904000	890000	962000	0.1409±0.0499	632000	652000	639000	597000	0.1309±0.0386	C05841	ko00760,ko01100
kz000259	[M+H]+	265.10	122.10	265.11	C12H17N4OS+	Thiamine	Vitamins	BioDeep	-	21200	27900	21500	24500	0.0039±0.0048	16500	19900	17300	16800	0.0048±0.0049	12100	10800	8570	11500	0.0026±0.0035	59500	63600	51100	69800	0.0092±0.0098	37900	44600	52300	48400	0.0095±0.0115	C00378	ko00730,ko01100,ko02010,ko04122
kz000263	[M+H]+	332.13	152.07	331.13	C14H21NO8	Pyridoxine-5'-O-glucoside	Vitamins	BioDeep	-	19500	19300	19300	20700	0.0033±0.0021	16500	17100	15100	16600	0.0044±0.0028	19000	16400	18500	16300	0.0043±0.0031	15900	18600	17400	14000	0.0025±0.0036	14000	11600	13400	12300	0.0027±0.0023	C03996	--
kz000274	[M+H]+	369.12	207.10	368.09	C20H16O7	N-sinapoylhydroxycoumarin	Coumarins	BioDeep	-	58600	77600	63700	65900	0.0109±0.0114	82500	97500	109000	106000	0.0267±0.0302	74700	70900	85800	80300	0.0189±0.0162	35000	35200	37800	46700	0.0058±0.0067	134000	179000	148000	149000	0.0317±0.0385	-	-
kz000276	[M+H]+	133.10	105.10	132.07	C8H8N2	4-Aminoindole	Alkaloids	BioDeep	5192-23-4	84700	78500	55100	76900	0.0122±0.029	40900	27300	35400	41500	0.0098±0.0168	-	-	-	-	-	-	-	-	-	-	-	-	-	-	-	-	-
kz000278	[M+H]+	176.10	130.10	175.06	C10H9NO2	Indole 3-acetic acid (IAA)	Alkaloids	BioDeep	87-51-4	-	-	-	-	-	-	-	-	-	-	14300	10300	11400	15000	0.0031±0.0051	31400	38100	33700	38000	0.0053±0.0047	12300	12700	14400	10900	0.0026±0.0023	C00954	ko00380,ko01100,ko04075
kz00	[M-	89.	59.	90.03	C3H6O3	3-Hydroxypr	Organic	Bio	503-	13	13	12	11	0.0214±0.0	9070	9740	9220	9950	0.0257±0.0	107000	10	1050	104000	0.0257±0.004	161000	146000	160000	163000	0.0237±0.0	35200	2910	3910	3390	0.0071±0.007	C010	ko00240,ko00410,ko00

0284	H]-	00	00			opanoic acid	acids	Deep	66-2	9000	6000	3000	9000	252	0	0	0	0	122		6000	00		3					1		0	0	0	8	13	640,ko01100,ko01200
kz000289	[M-H]-	117.00	59.00	118.03	C4H6O4	D-Erythronolactone	Organic acids	BioDeep	15667-21-7	557000	611000	660000	617000	0.0101±0.0073	43800	62700	49600	56300	0.0144±0.0235	75000	75000	72100	89600	0.0189±0.0185	68800	69300	95800	55300	0.0109±0.0282	76600	94600	89500	77500	0.0176±0.0156	-	-
kz000290	[M-H]-	118.00	74.00	119.02	C3H5NO4	Aminomalonic acid	Organic acids	BioDeep	1068-84-4	809000	1030000	838000	790000	0.0143±0.0176	53200	61600	58300	62300	0.0159±0.0116	99000	868000	88400	92200	0.0223±0.0098	192000	171000	177000	200000	0.0279±0.0133	226000	246000	248000	239000	0.0498±0.0151	C00872	--
kz000291	[M-H]-	122.03	78.00	123.03	C6H5NO2	2-Picolinic acid	Organic acids	BioDeep	98-98-6	2380000	2170000	2450000	2190000	0.038±0.043	149000	146000	149000	153000	0.0404±0.0039	394000	3860000	386000	408000	0.0957±0.0206	146000	158000	142000	159000	0.0228±0.0108	113000	121000	116000	107000	0.0237±0.0098	C10164	ko00380,ko01100
kz000293	[M-H]-	131.04	87.00	132.04	C5H8O4	Glutaric acid	Organic acids	BioDeep	110-94-1	4140000	4740000	4730000	4630000	0.0752±0.0325	160000	155000	149000	203000	0.0451±0.0626	282000	3370000	316000	288000	0.0744±0.0732	343000	333000	305000	306000	0.0485±0.0352	215000	193000	251000	209000	0.1583±2.3013	C00489	ko00071,ko00310,ko01100
kz000294	[M-H]-	131.08	85.00	132.08	C6H12O3	2-Hydroxyisocaproic acid	Organic acids	BioDeep	13748-90-8	500000	487000	455000	367000	0.0075±0.0138	79800	87700	86500	88600	0.0232±0.0111	16100	145000	13300	15500	0.0036±0.0026	73200	67400	78600	77000	0.0112±0.0074	44400	44800	42600	35700	0.0087±0.0082	-	-
kz000296	[M-H]-	147.00	87.10	148.04	C5H8O5	Citramalate	Organic acids	BioDeep	2306-22-1	504000	423000	538000	578000	0.0085±0.0153	80700	75800	80300	89000	0.022±0.0121	249000	2300000	23900	245000	0.0585±0.0119	84300	65500	66900	76700	0.011±0.012	36000	49600	57200	38700	0.0094±0.0179	C00815	--
kz00	[M-	16	79.	170.0	C3H7O6	DL-Glycerald	Organic	Bio	591-	43	59	42	46	0.0079±0.0	5700	7450	6230	6340	0.0174±0.0	35900	32	3090	30200	0.0079±0.005	84200	82500	102000	91900	0.0136±0.0	39600	3150	3540	3210	0.0072±0.008	C006	--

0300	H]-	9.00	00	0	P	ehyde 3-phosphate	acids	Deep	59-3	300	600	100	000	134	0	0	0	0	221		700	0		6					146		0	0	0	1	61	
kz000302	[M-H]-	175.10	115.00	176.07	C7H12O5	2-Isopropylmalate	Organic acids	BioDeep	-	494000	575000	492000	732000	0.0095±0.0219	765000	308000	418000	644000	0.0144±0.0557	37900	34800	36600	39400	0.009±0.0038	455000	493000	604000	675000	0.0838±0.1388	189000	196000	174000	210000	0.04±0.0403	C02504	ko00290,ko00620,ko01100,ko01110,ko01210,ko01230
kz000304	[M-H]-	209.12	59.02	210.13	C12H18O3	(±)-Jasmonic acid	Organic acids	BioDeep	77026-92-7	735000	865000	780000	818000	0.0132±0.0054	611000	627000	617000	626000	0.1678±0.0376	9910	11600	9200	9610	0.0025±0.0028	6850	6050	5790	7220	0.001±0.0008	11900	1260	1240	1210	0.0025±0.0006	C08491	ko00592,ko01100,ko01110,ko04075
kz000306	[M+H]+	225.15	151.11	224.14	C13H20O3	Methyl jasmonate	Organic acids	BioDeep	1211-29-6	524000	540000	635000	365000	0.0009±0.0024	44900	45600	42400	42900	0.0119±0.006	-	-	-	-	-	-	-	-	-	-	-	-	-	-	-	C11512	ko00592,ko01110
kz000307	[M-H]-	289.10	97.10	290.12	C10H18N4O6	Argininosuccinic acid	Organic acids	BioDeep	2387-71-5	213000	424000	641000	372000	0.0068±0.0357	23000	19300	8860	19000	0.0048±0.0167	54800	64400	19900	15100	0.0094±0.0604	26000	24000	11200	33500	0.0035±0.0131	14800	1430	1650	1380	0.0031±0.0017	C03406	ko00220,ko00250,ko01100,ko01110,ko01230
kz000308	[M-H]-	322.40	129.70	323.21	C18H29NO4	N-[(-)-Jasmonoyl]-(L)-Isoleucine (JA-L-Ile)	Organic acids	BioDeep	120330-93-0	155000	172000	152000	152000	0.0026±0.0015	3440	3500	3540	3550	0.0095±0.0014	1400	1340	1270	1530	0.0003±0.0002	2190	1820	2160	2130	0.0003±0.0003	2190	2140	2200	2070	0.0004±0.0001	C18699	ko04075
kz000310	[M-H]-	87.01	43.00	88.02	C3H4O3	Pyruvic acid	Organic acids	BioDeep	127-17-3	2680000	3120000	2800000	2500000	0.0458±0.0433	79600	98700	1000000	1010000	0.0257±0.0271	115000	94400	104000	91500	0.0246±0.0236	101000	77200	94300	103000	0.0141±0.0155	182000	175000	187000	202000	0.0388±0.0296	C00022	ko00010,ko00020,ko00030,ko00040,ko00053,ko00250,ko00260,ko00261,ko00270,ko00290,

																																				ko00330,ko00350,ko00360,ko00430,ko00440,ko00620,ko00630,ko00650,ko00660,ko00710,ko00730,ko00760,ko00770,ko00900,ko01100,ko01110,ko01200,ko01210,ko01230
kz000311	[M-H]-	103.00	59.00	104.01	C3H4O4	Malonic acid	Organic acids	BioDeep	141-82-2	8850000	9350000	8840000	8780000	1.4792±0.5213	12700000	9320000	11400000	10800000	2.989±3.6238	12000000	12400000	10400000	11000000	2.7839±2.2732	13200000	10600000	10700000	12400000	1.7657±1.6748	8040000	9090000	10500000	8580000	1.8784±1.7054	C00383	ko00061,ko00240,ko00410,ko01100,ko01212
kz000312	[M-H]-	145.01	101.00	146.02	C5H6O5	A-Ketoglutaric acid	Organic acids	BioDeep	328-50-7	4920000	5650000	5440000	5370000	0.0882±0.0246	3600000	3260000	3450000	3360000	0.0925±0.0367	818000	6800000	7840000	696000	0.1809±0.1482	741000	743000	713000	799000	0.1129±0.0245	472000	464000	390000	476000	0.0938±0.1063	C00026	ko00020,ko00040,ko00053,ko00220,ko00250,ko00300,ko00310,ko00340,ko00430,ko00630,ko00650,ko00660,ko01100,ko01110,ko01200,ko01210,ko01230
kz000410	[M+H]+	359.1	197.0	358.13	C16H22O9	Sweroside	Terpenoids	BioDe	93236-44	2620	3200	2840	2390	0.0456±0.0592	262000	253000	230000	259000	0.0679±0.0432	73400	5450	7390	64400	0.0162±0.021	277000	277000	266000	260000	0.0407±0.0216	540000	553000	530000	525000	0.1117±0.0375	C17071	--

		3	0					ep	-3	00	00	00	00								0															
kz000412	[M-H]-	345.12	183.20	346.13	C15H22O9	Aucubin	Terpenoids	BioDeep	479-98-1	396000	442000	427000	371000	0.0068±0.0056	13500	13800	14700	16200	0.0039±0.0028	195000	179000	187000	188000	0.0455±0.0097	131000	130000	126000	140000	0.0199±0.0034	26600	33700	32900	29200	0.0064±0.0058	C09771	--
kz000414	[M+H]+	109.08	92.00	108.07	C6H8N2	2-Picolylamine	Alkaloids	BioDeep	-	690000	651000	680000	662000	0.0111±0.0089	38500	33000	28500	31000	0.0089±0.0122	33700	47300	54700	48500	0.0112±0.0226	67500	65500	74500	57200	0.01±0.0132	27600	32100	31900	24300	0.006±0.0065	C17926	--
kz000417	[M-H]-	273.08	167.00	274.08	C15H14O5	Phloretin	Flavonoids	BioDeep	-	15300	16400	14100	18400	0.0026±0.0035	5220	6650	4270	5220	0.0014±0.0029	5030	7330	5070	4830	0.0014±0.0031	58800	55700	56200	59900	0.0087±0.0013	3490	2950	2370	2860	0.0006±0.0011	C00774	ko00941,ko01110
kz000419	[M-H]-	435.13	166.70	436.14	C21H24O10	Phlorizin	Flavonoids	BioDeep	-	9940	11200	7070	7910	0.0015±0.0038	16000	13000	16400	19400	0.0044±0.0065	11000	10800	12200	18600	0.0032±0.0088	109000	93500	109000	112000	0.016±0.011	-	-	-	-	-	C01604	ko00941,ko01110
kz000422	[M+H]+	273.08	153.00	272.07	C15H12O5	Butin	Flavonoids	BioDeep	-	212000	216000	221000	218000	0.0358±0.0151	175000	177000	181000	190000	0.0489±0.0135	119000	120000	121000	126000	0.0295±0.0085	430000	414000	410000	455000	0.0644±0.012	121000	123000	125000	124000	0.0256±0.0056	C09614	ko00941
kz000424	[M-H]-	287.06	135.00	288.06	C15H12O6	Eriodictyol	Flavonoids	BioDeep	-	271000	296000	255000	254000	0.0444±0.0363	62400	63200	61500	63900	0.017±0.0042	66700	64000	65600	63700	0.0158±0.0029	796000	825000	795000	836000	0.1226±0.0288	36600	34500	31700	29100	0.0069±0.0071	C05631	ko00941,ko01100,ko01110
kz000431	[M+H]+	451.1	289.0	450.12	C21H22O11	Eriodictyol 7-O-glucoside	Flavonoids	BioDe	-	8010	9350	9930	9690	0.0153±0.014	106000	84000	88200	116000	0.0266±0.0383	56000	5450	54700	57600	0.0135±0.0026	496000	540000	533000	498000	0.078±0.0539	20600	18600	22000	12400	0.0038±0.0082	-	-

		2	0					ep		0	0	0	0								0															
kz000434	[M-H]-	271.06	151.00	272.07	C15H12O5	Pinobanksin	Flavonoids	BioDeep	-	327000	317000	301000	305000	0.0517±0.0428	274000	281000	281000	292000	0.0763±0.0178	186000	179000	183000	190000	0.0448±0.0077	681000	671000	655000	680000	0.1013±0.021	199000	191000	202000	195000	0.0409±0.011	C09826	ko00941
kz000440	[M-H]-	449.11	151.00	450.12	C21H22O11	Astilbin	Flavonoids	BioDeep	-	170000	193000	172000	181000	0.0295±0.0107	727000	808000	790000	798000	0.2113±0.1074	58000	40800	46800	45000	0.0116±0.0162	786000	760000	780000	822000	0.1186±0.0156	10900	19200	16000	16100	0.0032±0.007	C17449	--
kz000441	[M-H]-	463.12	301.00	464.13	C22H24O11	Hesperetin 5-O-glucoside	Flavonoids	BioDeep	-	11900000	12300000	12600000	12700000	2.0444±0.842	4680000	4800000	4910000	4970000	1.3093±0.2704	5610000	5730000	6090000	5440000	1.3905±0.8037	12500000	13000000	12900000	12900000	1.9343±0.678	6470000	5940000	6360000	6300000	1.3034±0.6216	-	-
kz000446	[M+H]+	203.22	83.00	202.22	C10H26N4	Spermine	Alkaloids	BioDeep	-	6620000	7200000	6970000	6730000	1.1361±0.2774	4780000	4800000	4950000	4740000	1.3035±0.2918	6970000	6950000	6790000	6880000	1.677±0.2654	4920000	4760000	5070000	5870000	0.7761±0.5372	6180000	6610000	7060000	5290000	1.3041±1.2677	C00750	ko00330,ko00410,ko00480,ko00770,ko01100
kz000466	[M-H]-	121.03	92.00	122.04	C7H6O2	4-Hydroxybenzaldehyde	Phenolic acids	BioDeep	-	91100	110000	113000	102000	0.0172±0.015	109000	8200	8590	6120	0.0229±0.0541	377000	419000	402000	438000	0.0995±0.0703	420000	449000	442000	498000	0.0681±0.036	1820000	1910000	2020000	1950000	0.4001±0.1328	C00633	ko01100
kz000475	[M-H]-	137.02	93.00	138.03	C7H6O3	4-Hydroxybenzoic acid	Phenolic acids	BioDeep	-	1250000	1340000	1280000	1250000	0.2114±0.0618	743000	812000	757000	898000	0.2171±0.1837	769000	951000	1030000	1010000	0.2288±0.3099	3810000	3990000	3770000	4190000	0.5937±0.1826	4600000	5270000	5780000	5180000	1.0813±0.7915	C00156	ko00130,ko00790,ko01100,ko01110

kz000477	[M-H]-	137.06	119.05	138.07	$C_8H_{10}O_2$	Tyrosol	Phenolic acids	BioDeep	143236-03-7	62200	66700	64100	86100	0.0012±0.0022	7560	7760	10600	5270	0.0021±0.0059	6190	143000	10400	11200	0.0026±0.0084	18600	20200	22000	27600	0.0033±0.0051	26700	16200	17800	19200	0.0042±0.0103	C06044	ko00350,ko01100
kz000479	[M-H]-	147.05	77.01	148.05	$C_9H_8O_2$	Cinnamic acid	Phenolic acids	BioDeep	-	89700	99400	101000	98400	0.016±0.0063	447000	466000	463000	473000	0.1251±0.0322	35500	370000	33400	37900	0.0087±0.0049	7800	7020	6850	6190	0.0011±0.0012	31900	32100	38300	34200	0.0071±0.0049	C00423	ko00130,ko00360,ko00940,ko00998,ko01100,ko01110
kz000484	[M-H]-	151.04	136.00	152.05	$C_8H_8O_3$	Vanillin	Phenolic acids	BioDeep	485-72-3	1470000	1610000	1490000	1610000	0.2551±0.0912	3690000	3530000	2170000	2970000	0.0838±0.1948	1910000	2030000	1930000	1970000	0.4767±0.1879	4930000	4960000	4680000	5160000	0.7434±0.1598	4540000	4760000	4640000	4770000	0.9727±0.3645	C00755	ko00998,ko01100,ko01110
kz000487	[M-H]-	153.02	108.00	154.03	$C_7H_6O_4$	2,5-Dihydroxybenzoic acid	Phenolic acids	BioDeep	-	1460000	1820000	1810000	1710000	0.2803±0.2418	27200000	26900000	27900000	28700000	0.7486±0.1209	510000	4710000	5200000	468000	0.1197±0.0628	2160000	2140000	2170000	2420000	0.3348±0.1093	1880000	19100000	18500000	19000000	0.3921±0.1452	C00628	ko00350,ko01100
kz000489	[M-H]-	153.02	109.00	154.03	$C_7H_6O_4$	Protocatechuic acid	Phenolic acids	BioDeep	-	6160000	7320000	7210000	6810000	1.134±0.6547	115000000	109000000	113000000	115000000	3.0568±0.4845	2030000	1810000	1760000	1800000	0.4495±0.2426	8710000	8510000	8460000	9440000	1.3227±0.2903	7890000	83700000	74900000	78800000	1.6449±0.9927	C02230	ko01100,ko01110
kz000491	[M+H]+	165.05	119.05	164.05	$C_9H_8O_3$	p-Coumaric acid	Phenolic acids	BioDeep	-	6650000	6310000	7310000	6730000	0.1116±0.1125	1050000	90900	1020000	1030000	0.0271±0.0141	147000	149000	156000	173000	0.038±0.0286	321000	283000	329000	344000	0.0481±0.0326	408000	436000	408000	362000	0.0838±0.0566	C00811	ko00130,ko00350,ko00940,ko00950,ko00998,ko01100,ko01110
kz0004	[M-H]-	165.	119.	166.06	$C_9H_{10}O_3$	3-(4-Hydroxyphenyl)-	Phenolic	BioD	-	774	927	815	864	0.1394±0.073	216000	253000	207000	235000	0.0617±0.0627	44700	318	44900	38800	0.0097±0.0143	344000	302000	308000	379000	0.0501±0.0393	294000	330000	309000	312000	0.0647±0.0318	C01744	--

95		06	00			propionic acid	acids	eep		000	000	000	000								00															
kz000496	[M-H]-	167.03	108.00	168.04	C8H8O4	Vanillic acid	Phenolic acids	BioDeep	-	482000	501000	482000	500000	0.0812±0.0265	120000	124000	120000	144000	0.0343±0.0288	626000	5560000	579000	586000	0.1426±0.0501	996000	1040000	1070000	1060000	0.1571±0.0662	1370000	1330000	1350000	1330000	0.2797±0.0821	C06672	--
kz000500	[M-H]-	177.06	162.00	178.06	C10H10O3	Riboprine	Phenolic acids	BioDeep	-	6050	10800	6880	8870	0.0013±0.0038	4990	5830	5910	5900	0.0015±0.0012	21200	18900	20600	21900	0.005±0.0026	43100	46300	44600	43900	0.0067±0.0034	480000	512000	559000	509000	0.107±0.0452	C16427	ko00908,ko01110
kz000501	[M+H]+	179.07	147.10	178.06	C10H10O3	Methyl p-coumarate	Phenolic acids	BioDeep	-	1980000	2310000	2150000	2070000	0.0351±0.0144	93900	97900	90200	91400	0.0253±0.0133	199000	1900000	177000	200000	0.0465±0.0219	364000	359000	345000	330000	0.0527±0.0343	188000	198000	207000	193000	0.0408±0.0079	-	-
kz000502	[M+H]+	179.07	91.00	178.06	C10H10O3	Coniferaldehyde	Phenolic acids	BioDeep	-	138000	164000	129000	170000	0.0025±0.0035	-	-	-	-	-	76400	62800	64400	76100	0.017±0.0155	57600	67700	51900	65600	0.0091±0.0103	36000	50200	43500	42100	0.0089±0.0115	C02666	ko00940,ko01100,ko01110
kz000504	[M-H]-	179.03	135.05	180.04	C9H8O4	Caffeic acid	Phenolic acids	BioDeep	-	885000	1080000	1020000	1020000	0.1651±0.0962	189000	197000	144000	162000	0.0469±0.0729	148000	127000	120000	156000	0.0335±0.0376	514000	503000	529000	579000	0.08±0.0322	112000	104000	122000	122000	0.0239±0.0186	C01197	ko00940,ko01100,ko01110
kz000505	[M-H]-	179.07	146.00	180.08	C10H12O3	Coniferyl alcohol	Phenolic acids	BioDeep	-	3420	3630	3210	3880	0.0006±0.0006	-	-	-	-	-	48900	50200	50000	51700	0.0122±0.0037	65000	51900	51600	67600	0.0089±0.0106	22500	16200	26300	14700	0.0041±0.0105	C00590	ko00940,ko00998,ko01100,ko01110
kz00	[M-	18	15	182.0	C9H10O	Syringic Aldehyde	Phenoli	Bio	-	13	15	13	13	0.0232±0.0	1540	1280	2210	1170	0.0042±0.0	275000	25	2420	272000	0.0635±0.030	413000	347000	335000	375000	0.0554±0.0	421000	4410	4810	4610	0.0937±0.043	-	-

0507	H]-	1.05	1.00	6	4		c acids	Deep		3000	5000	5000	9000	117	0	0	0	0	123		7000	00		1					473		00	00	00	5		
kz000511	[M-H]-	193.05	134.01	194.06	C10H10O4	Ferulic acid	Phenolic acids	BioDeep	-	571000	654000	659000	597000	0.1024±0.062	1790000	2080000	1910000	1880000	0.0518±0.0392	409000	405000	449000	460000	0.1047±0.069	327000	371000	337000	341000	0.0519±0.0358	842000	841000	839000	794000	0.1723±0.0445	C01494	ko00940,ko01100,ko01110
kz000512	[M-H]-	197.05	123.00	198.05	C9H10O5	Syringic acid	Phenolic acids	BioDeep	-	183000	194000	186000	184000	0.0308±0.0085	83900	99800	92400	78800	0.024±0.0282	224000	211000	212000	220000	0.0527±0.0075	325000	322000	298000	309000	0.0473±0.023	230000	201000	246000	227000	0.047±0.036	C10833	--
kz000514	[M-H]-	207.07	133.10	208.07	C11H12O4	3,4-Dimethoxycinnamic acid	Phenolic acids	BioDeep	-	520000	612000	578000	618000	0.0096±0.0057	18900	21200	20400	21300	0.0055±0.0032	43500	45200	46100	46500	0.011±0.0043	51600	52500	48900	55400	0.0079±0.0024	57100	52100	58900	54200	0.0116±0.0055	-	-
kz000515	[M-H]-	207.07	192.00	208.07	C11H12O4	Sinapinaldehyde	Phenolic acids	BioDeep	104758-06-7	124000	121000	119000	115000	0.002±0.0015	4000	3910	5210	6010	0.0013±0.0025	26000	23300	27900	22900	0.0061±0.0057	36500	38700	35900	39400	0.0057±0.002	209000	203000	238000	210000	0.0447±0.0236	C05610	ko00940,ko01100,ko01110
kz000517	[M-H]-	209.08	179.00	210.09	C11H14O4	Sinapyl alcohol	Phenolic acids	BioDeep	61395	-	-	-	-	-	-	-	-	-	-	58600	44900	45200	44700	0.0117±0.015	104000	122000	111000	133000	0.0177±0.0161	-	-	-	-	-	C02325	ko00940,ko01100,ko01110
kz000520	[M-H]-	223.06	193.00	224.07	C11H12O5	Sinapic acid	Phenolic acids	BioDeep	55696-57-6	318000	376000	465000	318000	0.0061±0.0138	26900	30800	29500	22800	0.0074±0.0104	27700	43000	40400	34700	0.0089±0.0178	55600	64300	57800	78100	0.0096±0.0128	20200	19100	22600	16900	0.0041±0.004	C00482	ko00940,ko01100,ko01110
kz00	[M-	23	14	238.0	C12H14	Methyl sinapate	Phenoli	Bio	9067	11	12	83	10	0.0018±0.0	6620	7670	8910	6480	0.002±0.00	10300	95	8270	9320	0.0023±0.001	13300	14400	15000	17800	0.0023±0.0	2940	2100	1590	2160	0.0005±0.001	-	-

0522	H]-	7.08	7.00	8	O5		c acids	Deep	38-33-8	600	100	80	900	035					3		70			9					024					2		
kz000530	[M-H]-	291.01	247.00	292.02	C13H8O8	Brevifolin carboxylic acid	Phenolic acids	BioDeep	-	2090000	3490000	2900000	3640000	0.4978±1.276	669000	393000	364000	443000	0.1265±0.3767	-	-	-	-	-	572000	565000	380000	607000	0.0799±0.1416	-	-	-	-	-	-	-
kz000532	[M-H]-	315.07	153.02	316.08	C13H16O9	Protocatechuic acid-4-glucoside	Phenolic acids	BioDeep	-	5660000	6820000	5930000	5910000	0.1003±0.063	2540000	2650000	3100000	2720000	0.0744±0.0601	-	-	-	-	-	1190000	1010000	1140000	1160000	0.1696±0.1089	598000	627000	628000	644000	0.1298±0.052	-	-
kz000533	[M-H]-	315.07	153.08	316.08	C13H16O9	2,5-Dihydroxybenzoic acid O-hexside	Phenolic acids	BioDeep	41514-64-1	2680000	2910000	2440000	3060000	0.0046±0.0051	373000	341000	127000	140000	0.0067±0.036	118000	113000	105000	113000	0.0273±0.0109	396000	291000	419000	299000	0.0531±0.1074	117000	120000	84800	97400	0.0218±0.0373	-	-
kz000536	[M-H]-	325.06	125.00	326.06	C14H14O9	5-Galloylshikimic acid	Phenolic acids	BioDeep	-	986000	1180000	1240000	1130000	0.187±0.1727	24800	29100	28300	25200	0.0073±0.0065	-	-	-	-	-	96800	97800	104000	114000	0.0155±0.0088	40200	40300	44000	34600	0.0083±0.0063	-	-
kz000537	[M-H]-	325.06	93.00	326.06	C14H14O9	3-Galloylshikimic acid	Phenolic acids	BioDeep	-	2280000	2820000	2670000	2800000	0.0436±0.0343	6690	8290	6490	5910	0.0019±0.0031	-	-	-	-	-	26100	24600	22500	26000	0.0037±0.0019	10300	11000	11200	14100	0.0024±0.0039	-	-
kz000539	[M-H]-	327.14	165.09	328.15	C16H24O7	3-Hydroxy-4-isopropylbenzylalcohol 3-	Phenolic acids	BioDeep	-	5920000	6060000	5480000	6690000	0.01±0.0104	83800	71800	73900	86200	0.0213±0.0177	26800	20600	25700	26500	0.006±0.0064	165000	171000	151000	165000	0.0246±0.0129	47500	43500	45700	40600	0.0092±0.0059	-	-

						glucoside																														
kz000540	[M-H]-	329.12	167.00	330.13	C15H22O8	3,4,5-Trimethoxyphenyl-β-D-Glucopyranoside	Phenolic acids	BioDeep	6743-96-0	1300000	1280000	1200000	1170000	0.2046±0.1847	438000	476000	472000	452000	0.1243±0.058	7300000	7180000	7450000	7710000	1.8015±0.5411	2150000	2100000	2020000	2200000	0.3191±0.0552	704000	707000	657000	702000	0.1441±0.083	-	-
kz000542	[M-H]-	331.07	169.01	332.07	C13H16O10	1-O-Galloyl-β-D-glucose	Phenolic acids	BioDeep	-	1150000	1360000	1200000	1160000	0.2008±0.1285	1920000	1900000	1930000	1940000	0.5201±0.0388	3520000	3310000	3510000	3600000	0.8471±0.2332	4350000	4140000	4060000	4410000	0.639±0.1244	3020000	3010000	2940000	2910000	0.6176±0.2011	-	-
kz000544	[M-H]-	337.09	191.10	338.10	C16H18O8	3-O-p-Coumaroyl quinic acid	Phenolic acids	BioDeep	910028-78-3	60000	66700	49000	61100	0.0098±0.0142	628000	551000	596000	565000	0.1583±0.0894	19200	25900	17200	21700	0.0051±0.0096	830000	836000	898000	971000	0.1331±0.0754	625000	665000	622000	565000	0.1287±0.0767	C12208	ko00940,ko00941,ko00945,ko01110
kz000546	[M-H]-	337.09	173.10	338.10	C16H18O8	1-O-p-Coumaroyl quinic acid	Phenolic acids	BioDeep	-	89200	88200	97900	86200	0.0149±0.0135	102000	80700	89800	89500	0.0245±0.0227	-	-	-	-	-	106000	113000	124000	130000	0.0178±0.0145	-	-	-	-	-	-	-
kz000548	[M+H]+	339.11	147.04	338.10	C16H18O8	Trans-3-O-p-coumaric quinic acid	Phenolic acids	BioDeep	-	234000	325000	314000	341000	0.05±0.0817	2140000	2050000	2030000	1620000	0.5308±0.6741	81400	73800	76600	78500	0.0189±0.005	1090000	1110000	893000	883000	0.1501±0.2052	493000	533000	474000	538000	0.106±0.0847	-	-
kz000549	[M-H]-	341.09	179.04	342.10	C15H18O9	1-O-[(E)-Caffeoyl]-β-D-glucopyranose	Phenolic acids	BioDeep	-	645000	781000	696000	631000	0.1135±0.1028	665000	624000	667000	629000	0.1748±0.0575	408000	391000	345000	395000	0.0935±0.0601	919000	859000	1000000	872000	0.1377±0.1218	314000	295000	304000	325000	0.0644±0.0406	-	-
kz0005	[M-H]-	341.	179.	342.13	C16H22O8	Coniferin	Phenolic	BioD	-	754	753	713	649	0.1186±0.1252	714000	653000	648000	654000	0.1806±0.0931	413000	394	396000	380000	0.0962±0.0297	878000	902000	933000	942000	0.1378±0.0482	328000	338000	316000	330000	0.0682±0.0325	C00761	ko00940

50		12	00				acids	eep		000	000	000	000								000															
kz000551	[M+H]+	347.10	153.02	346.09	C14H18O10	Methyl 6-O-galloyl-β-D-glucopyranoside	Phenolic acids	BioDeep	-	-	-	-	-	-	1040000	1010000	1180000	1100000	0.0293±0.0166	-	-	-	-	-	-	-	-	-	-	500000	544000	491000	483000	0.1049±0.0592	-	-
kz000552	[M-H]-	353.09	135.10	354.10	C16H18O9	4-O-Caffeoyl quinic acid (criptochlorogenic acid)	Phenolic acids	BioDeep	-	39800	56500	51200	50800	0.0082±0.0109	11900	6690	21500	11600	0.0035±0.0164	-	-	-	-	-	32500	33000	33200	26600	0.0047±0.006	-	-	-	-	-	-	-
kz000553	[M-H]-	353.09	191.00	354.10	C16H18O9	Neochlorogenic acid(5-O-Caffeoylquinic acid)	Phenolic acids	BioDeep	-	1790000	1920000	2260000	2300000	0.0341±0.0494	82800	72300	83300	98400	0.0228±0.026	12900	140000	19500	14400	0.0037±0.0074	112000	94100	99300	111000	0.0157±0.0105	-	-	-	-	-	C17147	--
kz000554	[M-H]-	353.09	191.01	354.10	C16H18O9	Chlorogenic acid	Phenolic acids	BioDeep	-	3190000	3440000	4110000	3780000	0.0599±0.079	3200000	2920000	2930000	3370000	0.084±0.0532	55600	49100	51000	47700	0.0124±0.0073	184000	202000	205000	216000	0.0304±0.0179	77900	68700	71000	74900	0.0152±0.0115	C00852	ko00940,ko00941,ko00945,ko01110
kz000555	[M-H]-	353.09	191.10	354.10	C16H18O9	1-Caffeoylquinic acid	Phenolic acids	BioDeep	-	1430000	1680000	1790000	1700000	0.0272±0.0252	62100	63100	70000	64300	0.0175±0.0083	70100	46500	51800	70900	0.0145±0.0285	71600	78500	84500	78600	0.0118±0.0096	-	-	-	-	-	-	-
kz000557	[M-H]-	359.10	182.10	360.11	C15H20O10	Glucosyringic Acid	Phenolic acids	BioDeep	-	1310000	1380000	1290000	1270000	0.0217±0.0103	79000	76400	78900	86900	0.0217±0.0099	1010000	1030000	969000	1000000	0.2437±0.074	289000	274000	269000	290000	0.0423±0.0091	45800	58700	42100	45000	0.01±0.0156	-	-
kz	[M	3	2	37	C16	Trihydroxy	Phe	Bi	-	3	3	3	3	0.060	39	43	36	42	0.109	343	3	30	315	0.0783	178	159	1730	175	0.258	1130	11	10	94	0.0222	-	-

000560	-H]-	71.10	49.06	2.11	H20O10	cinnamoyl quinic acid	nolic acids	oDeep		16000	99000	81000	64000	2±0.048	8000	1000	9000	1000	6±0.0853	000	22000	9000	000	±0.0291	0000	0000	000	0000	2±0.1171	00	4000	6000	400	±0.0182		
kz000567	[M+H]+	427.05	295.05	426.04	C17H14O13	Ditartaroyl-hydroxylcoumarin	Phenolic acids	BioDeep	-	153000	165000	154000	169000	0.0265±0.012	112000	102000	106000	95200	0.0281±0.0205	116000	102000	104000	96600	0.0254±0.0178	212000	220000	227000	208000	0.0327±0.0215	141000	123000	146000	124000	0.0277±0.0212	-	-
kz000573	[M-H]-	471.09	307.05	472.10	C23H20O11	p-Coumaroylferuloyltartaric acid	Phenolic acids	BioDeep	-	144000	153000	145000	154000	0.0246±0.0079	3540	3950	3270	3320	0.0095±0.0041	25000	19400	2280	22700	0.0055±0.0049	224000	218000	222000	240000	0.0341±0.0056	51500	5770	5300	4640	0.0108±0.0085	-	-
kz000575	[M-H]-	477.14	169.00	478.15	C23H26O11	3,5-Di-O-galloylshikimic acid	Phenolic acids	BioDeep	-	191000	253000	311000	211000	0.004±0.0103	-	-	-	-	-	-	-	-	-	-	14900	18500	21000	17200	0.0027±0.0043	-	-	-	-	-	-	-
kz000577	[M-H]-	481.06	275.00	482.07	C20H18O14	Hexahydroxy-diphenoyl-glucose	Phenolic acids	BioDeep	-	8140000	8610000	9840000	8490000	1.4486±1.5082	719000	660000	743000	725000	0.1925±0.0705	2030000	1840000	1920000	1920000	0.4684±0.1258	834000	833000	840000	893000	0.1281±0.0173	868000	884000	938000	844000	0.1836±0.0347	-	-
kz000580	[M-H]-	483.08	169.01	484.09	C20H20O14	1,6-Di-O-Galloyl-D-Glucose	Phenolic acids	BioDeep	522-12-3	-	-	-	-	-	5860	4350	4730	6270	0.0143±0.0233	483000	536000	553000	573000	0.1304±0.1041	72800	49000	72300	62300	0.0097±0.0173	34900	4320	1720	3670	0.0069±0.0239	-	-
kz000584	[M-H]-	499.15	163.10	500.15	C22H28O13	5-O-p-Coumaroyl quinic acid O-hexoside	Phenolic acids	BioDeep	-	13100	12400	12200	12700	0.0021±0.0017	3410	3540	3390	3430	0.0093±0.0032	14600	11000	1420	8080	0.0029±0.0073	80400	78000	88000	102000	0.0131±0.0132	2260	5740	7010	4710	0.001±0.004	-	-

kz000592	[M-H]-	521.13	359.08	522.14	C24H26O13	Rosmarinyl Glucoside	Phenolic acids	BioDeep	-	445000	454000	256000	342000	0.0062±0.0198	40300	69200	50600	52500	0.0144±0.0343	428000	440000	392000	415000	0.1018±0.0524	169000	127000	155000	137000	0.0222±0.0299	-	-	-	-	-	-	-
kz000596	[M-H]-	547.17	223.10	548.17	C23H32O15	β-D-Furanofructosyl-α-D-(6-mustard acyl)glucoside	Phenolic acids	BioDeep	-	506000	621000	524000	648000	0.0095±0.0116	-	-	-	-	-	21100	26200	24500	31100	0.0063±0.0103	36800	35500	33100	36500	0.0053±0.019	6000	6420	4940	6890	0.0013±0.002	-	-
kz000612	[M-H]-	787.10	465.10	788.1	C34H28O22	1,3,4,6-Tetra-O-Galloyl-D-Glucose	Phenolic acids	BioDeep	-	274000	370000	366000	393000	0.0058±0.009	34100	31300	35600	23500	0.0084±0.0148	-	-	-	-	-	86200	71600	87200	143000	0.0145±0.0424	-	-	-	-	-	-	-
kz000613	[M-H]-	787.10	617.08	788.1	C34H28O22	1,2,3,6-Tetra-O-Galloyl-D-Glucose	Phenolic acids	BioDeep	-	189000	221000	245000	232000	0.0366±0.0423	69500	46100	61700	53400	0.0156±0.0267	17000	106000	13200	10200	0.0031±0.0072	217000	214000	204000	252000	0.0334±0.0215	32100	46700	27700	26800	0.0069±0.0191	-	-
kz000698	[M]+	449.11	287.06	449.1	C21H21O11+	Cyanidin-3-O-galactoside	Flavonoids	BioDeep	-	636000	671000	934000	900000	0.013±0.0311	131000	156000	204000	240000	0.0049±0.0126	27500	167000	24200	29900	0.006±0.0133	42400	62200	62200	49700	0.0082±0.0164	60000	61600	51300	51900	0.0117±0.0122	C08647	--
kz000700	[M]+	449.11	287.06	449.1	C21H21O11+	Cyanidin-3-O-glucoside (Kuromanin)	Flavonoids	BioDeep	50281-42-0	634000	900000	796000	687000	0.0124±0.0192	156000	129000	206000	155000	0.0044±0.0082	29300	138000	17200	23800	0.0051±0.016	27600	49100	33900	23800	0.0051±0.0178	57400	36100	49800	63400	0.0108±0.0263	C08604	ko00942
kz000703	[M]+	465.10	303.05	465.10	C21H21O12+	Delphinidin-3-O-glucoside (Mirtillin)	Flavonoids	BioDee	-	1020000	1410000	1280000	1520000	0.2152±0.3739	1750000	1720000	1950000	1640000	0.0477±0.0345	125000	408000	93200	92900	0.0213±0.0822	491000	221000	302000	352000	0.0514±0.1676	-	-	-	-	-	C12138	ko00942

								p		00	00	00	00																							
kz000704	[M-2H]-	465.08	285.04	467.10	C24H19O10+	Cyanidin-O-syringic acid	Flavonoids	BioDeep	-	366000	424000	412000	405000	0.0663±0.0241	387000	384000	386000	418000	0.1065±0.0355	419000	416000	391000	409000	0.0994±0.0287	497000	473000	500000	500000	0.0743±0.0227	-	-	-	-	-	-	-
kz000733	[M]+	627.16	303.05	627.16	C27H31O17+	Delphinidin-3,5-O-diglucoside	Flavonoids	BioDeep	-	-	-	-	-	-	-	-	-	-	-	-	-	-	-	-	16400	14700	12700	21600	0.0025±0.0049	8380	8370	6830	13300	0.0019±0.0063	C16312	ko00942
kz000779	[M-H]-	299.06	284.00	300.06	C16H12O6	Diosmetin	Flavonoids	BioDeep	-	277000	270000	253000	259000	0.0044±0.0036	13200	13300	17700	16000	0.0041±0.0054	20100	17900	19400	19600	0.0047±0.0017	52700	52600	48000	49500	0.0076±0.0043	8730	9260	9080	6650	0.0017±0.0023	C10038	--
kz000791	[M-H]-	431.10	269.10	432.11	C21H20O10	Apigenin 7-O-glucoside(Cosmosiin)	Flavonoids	BioDeep	-	266000	240000	220000	253000	0.0041±0.0055	72000	66700	82100	81400	0.0204±0.0172	256000	241000	241000	280000	0.0618±0.0399	76600	79600	56200	86600	0.0112±0.0176	23200	32900	25700	20600	0.0053±0.0105	C04608	ko00944
kz000792	[M+H]+	433.11	271.01	432.11	C21H20O10	Apigenin 5-O-glucoside	Flavonoids	BioDeep	-	419000	505000	382000	343000	0.0068±0.013	1040000	93100	94300	1050000	0.0268±0.0153	389000	405000	381000	423000	0.0971±0.0474	84100	89800	92500	115000	0.0143±0.0167	28700	33500	31500	29100	0.0064±0.0039	-	-
kz000795	[M+H]+	449.11	287.20	448.10	C21H20O11	Luteolin-7-O-glucoside(Cynaroside)	Flavonoids	BioDeep	-	6220000	6980000	4970000	6970000	1.0372±1.8263	2470000	2450000	2580000	2670000	0.6876±0.1861	5170000	5050000	6010000	5210000	1.3035±1.135	10900000	11000000	10400000	11400000	1.6466±0.338	7900000	7840000	8020000	7420000	1.6201±0.3661	C03951	ko00944
kz0007	[M-H]-	461.	285.	462.08	C21H18O12	Tetahydroxy-flavone-7-	Flavonoids	BioD	-	447	661	624	569	0.0095±0.0155	22100	27400	21400	26400	0.0066±0.0087	3510000	332	3150000	3540000	0.8214±0.3601	942000	983000	975000	985000	0.1465±0.0482	928000	932000	939000	912000	0.1929±0.0325	-	-

97		07	05			O-β-D-glucuronide		eep		00	00	00	00								00003	0														
kz000798	[M+H]+	463.09	287.06	462.08	C21H18O12	Luteolin-7-O-glucuronide	Flavonoids	BioDeep	6866-69-9	63200	82700	70600	73100	0.0119±0.0109	20800	28200	32800	25700	0.0073±0.0134	3080000	100000	3250000	3080000	0.7605±0.2722	937000	945000	950000	1030000	0.1455±0.0323	880000	885000	812000	838000	0.1776±0.1027	-	-
kz000800	[M-H]-	461.11	299.10	462.12	C22H22O11	Chrysoeriol-5-O-hexoside	Flavonoids	BioDeep	-	3350000	2920000	2940000	2730000	0.4943±0.7564	1110000	1120000	1110000	1240000	0.3097±0.1506	1380000	1340000	1350000	1390000	0.3318±0.0291	3110000	2840000	3000000	3050000	0.4523±0.1626	2060000	2180000	2100000	2180000	0.443±0.188	-	-
kz000812	[M-H]-	521.09	329.20	522.10	C23H22O14	Tricin O-saccharic acid	Flavonoids	BioDeep	-	616000	682000	585000	626000	0.1036±0.0636	210000	228000	208000	196000	0.057±0.0448	411000	383000	369000	399000	0.0949±0.033	1020000	1030000	1080000	1090000	0.1591±0.0541	632000	584000	632000	701000	0.1326±0.1225	-	-
kz000830	[M-H]-	607.17	299.00	608.17	C28H32O15	Chrysoeriol-7-O-rutinoside	Flavonoids	BioDeep	-	624000	668000	620000	576000	0.1027±0.075	443000	453000	420000	425000	0.1178±0.0603	10400	18300	15100	16100	0.0036±0.0085	16600	16800	17000	12300	0.0024±0.004	14200	15800	13300	17400	0.0032±0.0044	-	-
kz000831	[M+H]+	609.18	301.00	608.17	C28H32O15	Diosmin	Flavonoids	BioDeep	-	1140000	1340000	1320000	1320000	0.2111±0.1144	615000	589000	609000	631000	0.1653±0.0278	24500	18800	16900	17600	0.0047±0.0078	21400	26700	18600	26200	0.0035±0.0055	17100	16600	19000	20100	0.0038±0.0036	C10039	--
kz000841	[M+H]+	655.17	207.00	654.16	C32H30O15	Luteolin-O-sinapoylhexoside	Flavonoids	BioDee	-	21000	25100	25700	25900	0.004±0.0036	3840	4130	4560	3950	0.0011±0.0008	-	-	-	-	-	5460	4790	4550	3240	0.0007±0.0015	3130	3150	2020	2870	0.0006±0.0012	-	-

								p																												
kz000864	[M-H]-	285.04	151.00	286.05	C15H10O6	Kaempferol	Flavonoids	BioDeep	520-18-3	810000	901000	856000	895000	0.0143±0.0034	18800	17800	20700	15500	0.0049±0.0059	17000	165000	14500	18900	0.0041±0.0041	27800	24800	31600	31100	0.0043±0.0046	81900	77100	82100	80600	0.0167±0.0062	C05903	ko00941,ko00944,ko01100,ko01110
kz000866	[M+H]+	303.05	137.02	302.04	C15H10O7	Quercetin	Flavonoids	BioDeep	117-39-5	1650000	1960000	1810000	1760000	0.0296±0.0133	724000	602000	666000	810000	0.0189±0.0217	110000	1620000	151000	122000	0.0332±0.064	297000	275000	215000	290000	0.0405±0.0507	216000	215000	202000	199000	0.0433±0.0226	C00389	ko00941,ko00944,ko01100,ko01110
kz000872	[M+H]+	319.04	153.00	318.04	C15H10O8	Myricetin	Flavonoids	BioDeep	529-44-2	-	-	-	-	-	-	-	-	-	-	125000	137000	127000	141000	0.0322±0.0208	13400	16700	14500	16600	0.0023±0.0023	-	-	-	-	-	C10107	ko00941,ko00944,ko01110
kz000881	[M+H]+	435.09	303.00	434.08	C20H18O11	Avicularin	Flavonoids	BioDeep	572-30-5	15100000	17000000	17300000	16500000	0.2719±0.1253	1320000	1340000	1500000	1430000	0.3779±0.1843	-	-	-	-	-	1610000	1580000	1490000	1690000	0.2399±0.0672	189000	216000	170000	198000	0.0402±0.0453	-	-
kz000884	[M+H]+	449.11	287.06	448.10	C21H20O11	Kaempferol-3-O-glucoside (Astragalin)	Flavonoids	BioDeep	480-10-4	8050000	10100000	9610000	9210000	1.5236±1.1484	3450000	3750000	3420000	3600000	0.9622±0.5298	6450000	5790000	5930000	5380000	1.4309±0.9874	11200000	11800000	12600000	12600000	1.8169±1.0428	8890000	8530000	9300000	7710000	1.7877±1.0915	C12249	ko00944,ko01110
kz000886	[M-H]-	449.07	316.00	450.08	C20H18O12	Myricetin-3-O-arabinoside	Flavonoids	BioDeep	132679-85-7	22800	18600	15500	9970	0.0028±0.0012	-	-	-	-	-	-	-	-	-	-	44700	41400	45600	37800	0.0064±0.0068	102000	80200	89900	71400	0.0178±0.0272	-	-
kz00	[M-	46	30	464.1	C21H20	Quercetin-3-O-β-D-	Flavonoi	Bio	482-	19	22	22	22	0.3631±0.1	7490	7950	7760	8510	0.2145±0.1	921000	86	7610	822000	0.2045±0.148	201000	192000	2000000	206000	0.3011±0.0	1110000	1120	1150	9990	0.2274±0.098	C100	--

0888	H]-	3.09	0.00	0	O12	Galactoside (Hyperin)	ds	Deep	36-0	90000	40000	90000	80000	967	00	00	00	00	095		2000	00		6	0	0		0	615		000	000	00	9	73	
kz000889	[M-H]-	463.09	301.00	464.10	C21H20O12	Spiraeoside	Flavonoids	BioDeep	20229-56-5	12300000	12100000	13300000	12100000	2.0582±1.6529	4820000	4930000	4940000	5290000	1.3512±0.4659	5650000	5730000	6130000	5920000	1.4244±0.6318	12600000	12300000	12300000	12600000	1.8773±0.3657	6070000	6350000	6040000	6420000	1.2939±0.6516	-	-
kz000890	[M-H]-	463.09	300.70	464.10	C21H20O12	Quercetin-7-O-Glucoside	Flavonoids	BioDeep	491-50-9	24000000	22000000	20800000	27500000	0.039±0.0661	102000	141000	126000	166000	0.0362±0.0712	193000	196000	179000	192000	0.0462±0.0186	1120000	1110000	1100000	1220000	0.1714±0.0347	7270000	802000	771000	754000	1.587±0.5793	-	-
kz000892	[M+H]+	465.10	303.10	464.10	C21H20O12	6-Hydroxykaempferol-7-O-glucoside	Flavonoids	BioDeep	-	64600000	73200000	66700000	66300000	1.1174±0.3741	1640000	1630000	1810000	1660000	0.4558±0.1944	2010000	2050000	1850000	1920000	0.4759±0.23	4660000	4680000	4200000	5050000	0.7±0.3633	2460000	240000	247000	203000	0.486±0.3743	-	-
kz000904	[M-H]-	593.13	285.00	594.14	C30H26O13	Tiliroside	Flavonoids	BioDeep	20316-62-5	28500000	33000000	27000000	28800000	0.4839±0.401	3020000	3100000	3260000	3390000	0.8634±0.3543	6500000	6260000	6020000	6630000	1.544±0.5236	17500000	18500000	18400000	19100000	2.7703±0.9403	9700000	989000	10100000	987000	2.056±0.3243	C17140	--
kz000905	[M-H]-	593.15	285.00	594.16	C27H30O15	Kaempferol-3-O-rutinoside (Nicotiflorin)	Flavonoids	BioDeep	17650-84-9	270000	267000	258000	273000	0.0441±0.0271	628000	638000	547000	615000	0.1643±0.1294	8050000	7280000	7360000	7880000	1.8571±0.6512	788000	737000	713000	825000	0.1153±0.0454	1740000	186000	188000	186000	0.3814±0.1218	C21833	ko00944,ko01110
kz00	[M-	59	28	594.1	C27H30	Kaempferol-3-O-	Flavonoi	Bio	1729	25	24	25	22	0.0405±0.0	5710	5770	5670	5120	0.1507±0.0	749000	70	7240	741000	1.7744±0.215	760000	716000	686000	797000	0.1114±0.0	1800000	1610	1780	1500	0.3475±0.267	-	-

0906	H]-	3.15	5.00	6	O15	robinobioside(Biorobin)	ds	Deep	7-56-2	0000	3000	8000	8000	376	00	00	00	00	991	0	60000	000	0	7					443		000	000	000			
kz000907	[M+H]+	595.117	449.11	594.16	C27H30O15	Kaempferol-3-O-glucoside-7-O-rhamnoside	Flavonoids	BioDeep	-	7630	7810	5940	3480	0.001±0.0042	8050	6720	10800	14300	0.0027±0.0087	86200	98400	96000	119000	0.0243±0.0339	9270	6210	8430	10600	0.0013±0.0025	30000	27400	31900	21600	0.0058±0.0083	-	-
kz000908	[M-H]-	609.15	300.00	610.15	C27H30O16	Quercetin-3-O-robinobioside	Flavonoids	BioDeep	52525-35-6	698000	814000	653000	679000	0.1173±0.1179	15800000	15500000	17100000	15800000	0.4341±0.1608	6160000	5310000	6220000	6070000	1.4435±0.9171	3140000	3460000	3250000	3510000	0.5035±0.2353	1980000	2030000	2180000	2100000	0.4307±0.1398	-	-
kz000909	[M-H]-	609.15	301.00	610.15	C27H30O16	Quercetin-3-O-rutinoside(Rutin)	Flavonoids	BioDeep	153-18-4	609000	722000	616000	606000	0.1053±0.0805	13700000	13500000	13300000	13600000	0.366±0.0803	5070000	4540000	4780000	5170000	1.1882±0.5483	2570000	2740000	2680000	2760000	0.4052±0.1463	1470000	1570000	1620000	1390000	0.3141±0.1388	C05625	ko00944,ko01100,ko01110
kz000911	[M+H]+	611.16	303.10	610.15	C27H30O16	Quercetin-7-O-rutinoside	Flavonoids	BioDeep	147714-62-3	595000	703000	559000	609000	0.1017±0.0971	11100000	12500000	12200000	10300000	0.312±0.3075	3660000	3790000	4110000	4150000	0.9551±0.634	2340000	2500000	2290000	2230000	0.3531±0.2486	1300000	1320000	1510000	1440000	0.2894±0.1792	-	-
kz000912	[M-H]-	623.16	315.02	624.17	C28H32O16	Isorhamnetin-3-O-rutinoside(Narcissin)	Flavonoids	BioDeep	604-80-8	887000	827000	980000	873000	0.0148±0.017	2180000	1970000	2560000	2450000	0.0619±0.063	1040000	1070000	1020000	1020000	0.2523±0.0814	264000	263000	299000	303000	0.0425±0.0299	91900	91200	92100	91100	0.019±0.0045	-	-
kz0009	[M+H]+	627.	303.	626.15	C27H30O17	6-Hydroxykaempferol-	Flavonoids	BioD	-	780	998	981	998	0.0155±0.0164	17900	21200	14500	15600	0.0047±0.0088	83000	101	9170	113000	0.0236±0.0323	917000	724000	914000	825000	0.1275±0.1457	70700	61700	79200	61600	0.0142±0.0148	-	-

14		16	10			3,6-O-Diglucoside		eep		00	00	00	00								000															
kz000915	[M+H]+	627.16	303.10	626.15	C27H30O17	6-Hydroxykaempferol-7,6-O-Diglucoside	Flavonoids	BioDeep	-	340000	395000	391000	379000	0.0621±0.0286	44600	46000	41800	44400	0.012±0.0063	107000	120000	107000	126000	0.028±0.0245	380000	400000	405000	387000	0.0593±0.0313	174000	162000	160000	168000	0.0345±0.0211	-	-
kz000919	[M+H]+	741.22	433.00	740.22	C33H40O19	Robinin(Kaempferol-3-O-gal-rham-7-O-rham)	Flavonoids	BioDeep	301-19-9	-	-	-	-	-	-	-	-	-	-	200000	186000	181000	184000	0.0456±0.0158	-	-	-	-	-	5960	10400	12300	7480	0.0019±0.0054	C10178	--
kz000920	[M-H]-	771.20	609.10	772.21	C33H40O21	Quercetin-O-rutinoside-hexose	Flavonoids	BioDeep	-	13500	9170	9410	7380	0.0016±0.0061	-	-	-	-	-	9630	9780	6930	9780	0.0022±0.0033	9370	11500	13100	8660	0.0016±0.0035	21900	27500	18400	22100	0.0047±0.0082	-	-
kz000923	[M+H]+	803.19	303.10	802.18	C33H38O23	6-Hydroxykaempferol-3,6-O-Diglucoside-7-O-Glucuronic Acid	Flavonoids	BioDeep	-	32400	50600	57000	42300	0.0075±0.0201	19100	18400	18600	10900	0.0045±0.0109	13500	19900	9270	10700	0.0032±0.0118	11600	8570	9580	14100	0.0016±0.0032	3650	5640	7270	5180	0.0011±0.0028	-	-
kz000969	[M+H]+	275.09	139.00	274.08	C15H14O5	Afzelechin(3,5,7,4'-Tetrahydroxyflavan)	Flavonoids	BioDeep	2545-00-8	457000	412000	458000	474000	0.0745±0.0804	248000	260000	261000	261000	0.0697±0.0188	695000	663000	635000	644000	0.1602±0.0536	905000	836000	980000	1010000	0.1405±0.1021	6340	6490	8270	3760	0.0013±0.0036	C09320	ko00941,ko01110
kz000971	[M-H]-	289.07	245.00	290.08	C15H14O6	Catechin	Flavonoids	BioDeep	154-23-4	3080000	3040000	2770000	2920000	0.4881±0.412	1380000	1420000	1440000	1320000	0.3762±0.1762	5540000	5030000	5110000	5640000	1.2952±0.5777	5760000	5540000	5700000	5790000	0.8591±0.1877	25700	27900	31500	16400	0.0053±0.0123	C06562	ko00941,ko01110

kz000973	[M-H]-	305.07	125.00	306.07	C15H14O7	Epigallocatechin (EGC)	Flavonoids	BioDeep	970-74-1	3600000	3190000	3110000	2930000	0.0053±0.0081	190000	129000	177000	162000	0.0044±0.0068	119000	1150000	109000	119000	0.0281±0.0092	231000	202000	222000	217000	0.0329±0.0196	-	-	-	-	-	C12136	ko00941,ko01110
kz000974	[M+H]+	307.08	139.00	306.07	C15H14O7	Gallocatechin	Flavonoids	BioDeep	970-73-0	4470000	5050000	5810000	6060000	0.0088±0.0139	277000	198000	184000	218000	0.0059±0.0112	193000	1780000	166000	182000	0.0437±0.0222	363000	371000	374000	384000	0.0562±0.0133	-	-	-	-	-	C12127	ko00941,ko01110
kz000978	[M-H]-	451.10	341.06	452.11	C24H20O9	Catechin-(7,8-bc)-4β-(3,4-dihydroxyphenyl)-dihydro-2-(3H)-pyranone	Flavonoids	BioDeep	-	22700000	25400000	23900000	25500000	0.4023±0.1396	9350000	9420000	9630000	9430000	0.2559±0.0396	97200	97700	98900	95200	0.0236±0.0063	420000	457000	467000	433000	0.067±0.0475	29000	30000	34100	30900	0.0064±0.0033	-	-
kz000979	[M-H]-	451.10	341.06	452.11	C24H20O9	Catechin-(7,8-bc)-4α-(3,4-dihydroxyphenyl)-dihydro-2-(3H)-pyranone	Flavonoids	BioDeep	-	22400000	24800000	23000000	24400000	0.3904±0.1037	9940000	8970000	9530000	9550000	0.2569±0.0913	109000	97300	99600	103000	0.0248±0.0086	480000	465000	473000	473000	0.0713±0.0202	26900	35500	25100	30500	0.0061±0.01	-	-
kz000992	[M-H]-	357.13	151.00	358.14	C20H22O6	Pinoresinol	Lignans	BioDeep	487-36-5	45500000	50500000	47700000	46000000	0.0783±0.0241	10700000	11000000	11500000	12100000	0.0306±0.0134	61400	639000	56100	67300	0.0151±0.0113	430000	429000	370000	431000	0.0625±0.039	152000	142000	147000	149000	0.0307±0.0143	C05366	ko00998,ko01100,ko01110
kz000995	[M-H]-	417.16	402.10	418.16	C22H26O8	Syringaresinol	Lignans	BioDeep	21453-69-0	1060000	1170000	1060000	1150000	0.0183±0.0071	199000	216000	219000	231000	0.0058±0.0033	93300	919000	91300	96500	0.0227±0.0048	107000	110000	106000	110000	0.0163±0.0041	29700	25800	28400	29700	0.0059±0.0047	C10889	--
kz00	[M-	51	35	520.1	C26H32	Terpineol monO-	Lignans	Bio	-	57	62	49	50	0.9104±1.2	2380	2130	2630	2500	0.6514±0.4	112000	12	1310	138000	0.0308±0.028	157000	167000	1500000	171000	0.243±0.11	814000	8350	9020	8600	0.1772±0.056	-	-

0997	H]-	9.19	7.14	9	O11	glucoside		Deep		70000	70000	80000	30000	46	000	000	000	000	817		6000	00		8	0	0		0	03		00	00	00	8		
kz001008	[M+H]+	102.05	56.05	101.05	C4H7NO2	1-Aminocyclopropane-1-carboxylic acid	Others	BioDeep	22059-21-8	16000	30100	22600	18100	0.0036±0.0112	11400	13500	12900	15500	0.0036±0.0044	13200	14200	13200	14600	0.0034±0.0019	12000	12500	13600	11700	0.0019±0.0017	14800	20800	28000	20900	0.0044±0.0102	C01234	ko00270,ko01100,ko01110
kz001014	[M+H]+	126.10	68.00	125.10	C6H11N3	1-Methylhistamine	Others	BioDeep	6481-48-7	-	-	-	-	-	-	-	-	-	-	14200	12000	7130	10600	0.0027±0.007	17200	10700	12100	14200	0.002±0.0041	149000	151000	156000	149000	0.0314±0.0022	-	-
kz001018	[M-H]-	138.02	79.96	139.03	C3H9NO3S	3-Amino-1-propionic sulfonic acid	Others	BioDeep	3687-18-1	11600	9220	12300	10500	0.0018±0.0034	9880	7830	10500	11600	0.0027±0.0039	13300	10200	1100	7050	0.0025±0.0061	44300	45500	43200	48100	0.0068±0.0017	19100	1830	2360	1520	0.0039±0.0062	-	-
kz001020	[M+H]+	144.05	113.20	143.04	C6H9NOS	4-Methyl-5-thiazoleethanol	Others	BioDeep	137-00-8	4650000	5520000	5160000	5030000	0.084±0.0359	4190000	4330000	4610000	4400000	0.1186±0.0408	1510000	1510000	1450000	1530000	0.3647±0.0819	202000	205000	185000	208000	0.0301±0.0121	70100	7540	7310	6680	0.0148±0.0058	C04294	ko00730,ko01100
kz001025	[M-H]-	175.02	85.03	176.03	C6H8O6	D-Glucurono-6,3-lactone	Others	BioDeep	32449-92-6	528000	737000	730000	674000	0.011±0.016	63100	48200	61900	59700	0.0157±0.0168	19000	23000	24900	25000	0.0056±0.0073	533000	539000	535000	544000	0.0811±0.019	403000	41300	42300	40400	0.0854±0.0052	C02670	ko00053,ko01100
kz001032	[M+H]+	222.10	84.05	221.09	C8H15NO6	N-Acetyl-D-galactosamine	Others	BioDeep	1811-31-0	1500000	1480000	1400000	1320000	0.0236±0.0229	75000	84800	77400	72700	0.021±0.0174	57900	58000	41300	63700	0.0134±0.0229	200000	170000	190000	207000	0.0289±0.019	173000	162000	155000	138000	0.0326±0.031	-	-

kz001041	[M-H]-	300.05	96.90	301.06	C8H16NO9P	N-Acetyl-D-glucosamine 1-phosphate	Others	BioDeep	-	791000	910000	831000	861000	0.014±0.0039	83800	85700	82900	84700	0.0228±0.0064	46700	465000	41600	43800	0.0109±0.0057	197000	199000	196000	191000	0.0295±0.0131	36600	39300	35400	37500	0.0077±0.0046	C04256	--
kz001074	[M-H]-	169.01	125.00	170.02	C7H6O5	Gallic acid	Phenolic acids	BioDeep	149-91-7	2110000	3260000	2710000	3140000	0.4612±0.8828	196000	224000	190000	179000	0.0534±0.0608	141000	113000	118000	130000	0.0305±0.0258	4740000	4700000	4720000	5470000	0.739±0.3541	151000	150000	156000	140000	0.031±0.0087	C01424	--
kz001075	[M-H]-	183.03	124.00	184.04	C8H8O5	Methyl gallate	Phenolic acids	BioDeep	99-24-1	401000	361000	370000	328000	0.0604±0.0857	66100	61000	69500	71200	0.0181±0.0093	65700	538000	53700	50300	0.0136±0.0149	515000	574000	513000	487000	0.0788±0.0723	126000	128000	151000	131000	0.0278±0.0176	-	-
kz001076	[M-H]-	197.05	124.00	198.05	C9H10O5	Ethyl gallate	Phenolic acids	BioDeep	831-61-8	6430000	6750000	7400000	6860000	0.1133±0.0817	210000	226000	370000	262000	0.0072±0.0187	43500	319000	42800	33800	0.0092±0.014	7440000	7250000	7260000	7940000	1.126±0.1586	98600	106000	87900	99000	0.0204±0.0189	-	-
kz001079	[M-H]-	335.04	124.00	336.05	C15H12O9	Galloyl Methyl gallate	Phenolic acids	BioDeep	-	497000	682000	598000	588000	0.0097±0.011	54600	58600	56800	54300	0.0152±0.0074	-	-	-	-	-	194000	193000	158000	205000	0.0282±0.0257	79100	75600	71900	59000	0.0148±0.0178	-	-
kz001084	[M-H]-	455.35	455.36	456.36	C30H48O3	Ursolic acid	Terpenoids	BioDeep	77-52-1	640000	927000	576000	616000	0.0113±0.0287	65200	56400	68700	77500	0.0181±0.021	82200	73300	73100	94100	0.0196±0.0224	1750000	1510000	1570000	1600000	0.2424±0.1527	371000	319000	372000	305000	0.071±0.0653	C08988	--
kz001086	[M-H]-	469.33	423.33	470.34	C30H46O4	2,3-Dihydroxy 5(6),12(13)diene ursolic	Terpenoids	BioDee	-	134000	153000	124000	144000	0.0229±0.0205	104000	93500	95800	104000	0.0269±0.0131	-	-	-	-	-	-	-	-	-	-	9240	10600	9350	10200	0.002±0.0016	-	-

						acid		p		0	0	0	0																							
kz001087	[M-H]-	471.35	471.35	472.36	C30H48O4	Maslinic acid	Terpenoids	BioDeep	4373-41-5	6970000	7920000	6620000	7660000	1.2034±0.9448	10900000	11400000	11300000	11500000	3.0506±0.8147	4670000	4490000	4590000	4900000	1.1333±0.3483	11500000	12300000	11400000	11700000	1.7685±0.8367	12100000	11600000	11600000	11500000	2.4332±0.9548	C16939	--
kz001088	[M-H]-	471.35	471.35	472.36	C30H48O4	3,24-Dihydroxy-17,21-semiacetal-12(13)oleanolic acid	Terpenoids	BioDeep	-	6860000	7830000	6650000	7340000	1.1832±0.7399	10700000	11000000	11500000	11100000	2.9961±0.7881	4520000	4350000	4380000	4730000	1.0926±0.3404	11800000	11800000	10800000	12000000	1.7483±0.6231	11500000	12000000	11100000	11900000	2.4185±1.35	-	-
kz001089	[M-H]-	471.35	471.35	472.36	C30H48O4	2-Hydroxyoleanolic acid	Terpenoids	BioDeep	26707-60-8	6890000	7740000	6820000	7550000	1.1966±0.6156	10900000	10700000	11000000	11000000	2.9488±0.109	4590000	4350000	4440000	4880000	1.1095±0.4766	11900000	11800000	11000000	11700000	1.749±0.6238	11600000	11800000	11200000	11800000	2.4131±1.1531	-	-
kz001090	[M-H]-	485.36	453.34	486.37	C31H50O4	Methoxyursolic acid	Terpenoids	BioDeep	-	183000	186000	162000	187000	0.0297±0.027	340000	328000	353000	370000	0.094±0.035	22300	23200	21100	22200	0.0054±0.0024	1040000	958000	962000	1020000	0.15±0.0464	4570000	4470000	4440000	4250000	0.9216±0.3225	-	-
kz001091	[M-H]-	487.34	469.23	488.35	C30H48O5	Euscaphic acid	Terpenoids	BioDeep	53155-25-2	10900000	11200000	10600000	10900000	1.801±0.7927	15100000	15800000	15300000	16000000	4.2075±1.3466	4040000	3900000	3970000	4130000	0.9748±0.1639	7540000	7450000	7400000	7730000	1.1352±0.1143	11000000	10900000	10800000	10800000	2.2616±0.6899	C17890	--
kz001093	[M-H]-	617.41	455.40	618.41	C36H58O8	Oleanolic acid 2-O-β-D-glucopyranoside	Terpenoids	BioDeep	14162-53-9	23200	24700	23900	24900	0.004±0.001	33200	32500	34800	35700	0.0092±0.0026	4950	4360	3680	4230	0.001±0.0012	17000	15400	14800	15900	0.0024±0.0013	52000	52500	51800	52800	0.0109±0.0036	-	-

kz001096	[M+H]+	104.10	60.10	103.10	C5H13NO	Choline	Alkaloids	BioDeep	62-49-7	290000	318000	314000	295000	0.0502±0.0179	1110000	1260000	1210000	1170000	0.0321±0.0202	294000	306000	282000	293000	0.0714±0.0286	392000	383000	338000	401000	0.057±0.0327	348000	367000	345000	351000	0.0734±0.0295	C00114	ko00260,ko00564,ko01100,ko02010
kz001097	[M+H]+	118.09	59.00	117.08	C5H11NO2	Betaine	Alkaloids	BioDeep	107-43-7	111000	123000	108000	116000	0.0189±0.009	46800	46100	45700	53100	0.013±0.0084	14900	13800	17900	16600	0.0038±0.0045	182000	180000	157000	173000	0.0261±0.0171	58900	67700	67300	64700	0.0134±0.007	C00719	ko00260,ko01100,ko02010
kz001100	[M+H]+	138.05	78.00	137.05	C7H7NO2	Nicotinic Acid Methyl Ester(Methyl Nicotinate)	Alkaloids	BioDeep	93-60-7	287000	362000	369000	346000	0.0562±0.058	227000	207000	195000	193000	0.0556±0.0472	255000	256000	265000	265000	0.0633±0.0176	186000	181000	182000	202000	0.0283±0.0064	29100	31300	31800	27700	0.0062±0.0026	-	-
kz001101	[M+H]+	138.05	94.07	137.05	C7H7NO2	Trigonelline	Alkaloids	BioDeep	535-83-1	2550000	2870000	2550000	2610000	0.4366±0.1808	4580000	4510000	4160000	4540000	1.2036±0.6205	665000	6330000	607000	645000	0.1549±0.043	6780000	6420000	6320000	6610000	0.9849±0.2788	7280000	7440000	6950000	6980000	1.4896±0.6818	C01004	ko00760
kz001103	[M+H]+	140.03	94.00	139.03	C6H5NO3	6-Hydroxynicotinic acid	Alkaloids	BioDeep	5006-66-6	8930	14100	8010	11700	0.0018±0.005	3440	3110	3390	2680	0.0085±0.0097	15000	8770	8120	8790	0.0025±0.0075	76400	75000	7480	82700	0.0116±0.0022	7930	6930	8200	7490	0.0159±0.0107	C01020	ko00760,ko01100
kz001105	[M+H]+	147.13	87.20	146.12	C7H16NO2	Acetylcholine	Alkaloids	BioDeep	60-31-1	1380000	1840000	1750000	1950000	0.0285±0.0416	1150000	1230000	1350000	1500000	0.0353±0.0372	152000	1520000	163000	152000	0.0376±0.0163	445000	447000	456000	509000	0.0699±0.0281	309000	290000	304000	294000	0.0622±0.0228	C08201	--
kz001113	[M+H]+	243.0	145.0	242.08	C12H10N4O2	Lumichrome	Alkaloids	BioDe	1086-80	5050	3840	3020	4720	0.0007±0.0021	14400	11300	14700	14900	0.0037±0.0041	44900	4340	4580	49800	0.0112±0.0062	14000	12000	14700	15100	0.0021±0.0019	37000	2990	3410	4120	0.0074±0.0113	C01727	ko00740

		9	0					ep	-2												0															
kz001117	[M+H]+	343.330	240.700	342.29	C19H38N2O3	Cocamidopropyl betaine	Alkaloids	BioDeep	4292/10/8	7540	9700	8930	8700	0.0014±0.0012	13100	13000	13500	11800	0.0035±0.0022	38800	155000	13700	13800	0.0049±0.0289	14700	17000	17400	18100	0.0025±0.0021	14500	16100	39600	29800	0.0052±0.0237	-	-
kz001154	[M+H]+	123.06	79.90	122.05	C6H6N2O	Nicotinamide	Vitamins	BioDeep	98-92-0	860000	954000	907000	907000	0.1497±0.0105	680000	672000	663000	706000	0.184±0.0466	182000	1820000	171000	181000	0.0435±0.0125	414000	400000	400000	433000	0.062±0.006	336000	344000	384000	333000	0.0725±0.03	C00153	ko00760,ko01100
kz001155	[M+H]+	124.04	78.00	123.03	C6H5NO2	Nicotinic acid	Vitamins	BioDeep	59-67-6	981000	1090000	981000	1040000	0.1689±0.0569	4620000	4800000	4550000	5020000	0.1285±0.0584	931000	9310000	885000	916000	0.2226±0.0542	543000	547000	546000	566000	0.083±0.0127	464000	457000	526000	440000	0.098±0.0537	C00253	ko00760,ko00960,ko01100,ko01110
kz001156	[M+H]+	170.08	134.10	169.07	C8H11NO3	Pyridoxine	Vitamins	BioDeep	65-23-6	109000	117000	113000	112000	0.0186±0.0031	43600	40600	40100	43100	0.0113±0.0046	28200	215000	24400	22300	0.0059±0.0066	56100	51000	46700	53200	0.0078±0.0052	55100	62500	54900	53400	0.0117±0.0084	C00314	ko00750,ko01100
kz001157	[M+H]+	184.06	148.00	183.05	C8H9NO4	4-Pyridoxic acid	Vitamins	BioDeep	82-82-6	165000	146000	195000	170000	0.0028±0.0047	13600	11100	12000	13200	0.0034±0.0028	9380	9150	9040	9130	0.0022±0.0002	24600	25100	20700	27200	0.0037±0.0033	28600	30700	30400	28600	0.0061±0.0014	C00847	ko00750,ko01100
kz001158	[M+H]+	220.12	202.10	219.11	C9H17NO5	D-Pantothenic Acid	Vitamins	BioDeep	79-83-4	696000	852000	732000	836000	0.0128±0.0109	411000	547000	481000	470000	0.0129±0.0165	504000	4800000	482000	538000	0.1218±0.0561	610000	559000	576000	627000	0.0893±0.0271	71200	73100	135000	76500	0.0184±0.0583	C00864	ko00410,ko00770,ko01100,ko01110
kz0011	[M+H]+	377.	243.	376.14	C17H20N4	Riboflavin	Vitamins	BioD	83-88	236	253	236	277	0.0414±0.0355	317000	332000	297000	328000	0.0862±0.0509	1000000	105	948000	989000	0.2424±0.1174	225000	196000	204000	207000	0.0314±0.0185	298000	331000	383000	320000	0.0691±0.0575	C00255	ko00740,ko01100,ko01110,ko0201

63		15	00		O6			eep	-5	000	000	000	000								0000															0
kz001175	[M-H]-	177.02	133.20	178.03	C9H6O4	Esculetin	Coumarins	BioDeep	305-01-1	17300000	18800000	19500000	18700000	0.0307±0.014	57400	47200	46500	40400	0.013±0.0199	38600	484000	51400	56800	0.0119±0.0192	167000	131000	136000	138000	0.0216±0.0246	87900	68100	97000	79500	0.0173±0.0235	C09263	--
kz001177	[M+H]+	207.07	151.00	206.06	C11H10O4	Scoparone	Coumarins	BioDeep	120-08-1	14800000	15400000	14200000	13900000	0.0241±0.0157	77200	73600	68700	70400	0.0196±0.0122	22300	22500	15200	20600	0.0049±0.0082	-	-	-	-	-	26300	21900	21800	23100	0.0048±0.0052	C09311	--
kz001184	[M+H]+	325.09	163.00	324.08	C15H16O8	Skimmin	Coumarins	BioDeep	93-39-0	28500000	32600000	29500000	28800000	0.0493±0.0222	83900	77400	83400	107000	0.0238±0.0325	88300	72800	93300	81500	0.0204±0.0207	84200	72900	70000	80500	0.0116±0.0082	408000	394000	388000	428000	0.0842±0.0576	-	-
kz001188	[M-H]-	339.07	177.00	340.08	C15H16O9	Esculin(6,7-Dihydroxy Coumarin-6-glucoside)	Coumarins	BioDeep	531-75-9	6420000	5140000	3600000	4460000	0.0081±0.0275	55000	64300	78000	55600	0.0171±0.0286	124000	123000	112000	113000	0.0287±0.0154	40000	54000	43000	50300	0.0071±0.0096	199000	208000	193000	171000	0.0401±0.0305	C09264	--
kz001191	[M-H]-	485.13	323.20	486.14	C21H26O13	4-hydroxycoumarin di-glucoside	Coumarins	BioDeep	-	2120000	2090000	2100000	2100000	0.0348±0.0196	1050000	1120000	1000000	1170000	0.0294±0.0212	-	-	-	-	-	9010	7840	5710	8740	0.0012±0.0021	-	-	-	-	-	-	-
kz001193	[M-H]-	283.06	268.00	284.07	C16H12O5	Prunetin	Flavonoids	BioDeep	552-59-0	1190	1610	1390	1350	0.0002±0.0002	4990	4750	5300	5400	0.0014±0.0006	1840	1850	2030	1670	0.0004±0.0004	30800	32800	31000	33300	0.0048±0.0016	2100	1610	2540	1950	0.0004±0.0007	C10521	ko00943
kz00	[M+H]	11	91.	117.0	C8H7N	Indole	Alkaloid	Bio	120-	10	13	15	13	0.0022±0.0	1420	1380	1270	1410	0.0037±0.0	12300	16	1310	16200	0.0035±0.005	10500	15500	13100	13300	0.002±0.00	15100	1120	1480	1280	0.0028±0.003	C004	ko00380,ko00400,ko00

1198	+	8.07	00	6			s	Deep	72-9	300	800	200	900	038	0	0	0	0	021		300	0		3					33		0	0	0	7	63	402,ko01100,ko01110
kz001199	[M+H]+	146.06	91.00	145.05	C9H7NO	Indole-3-carboxaldehyde	Alkaloids	BioDeep	487-89-8	296000	378000	379000	299000	0.0056±0.0081	24200	31900	19700	33300	0.0074±0.0177	69600	70800	73900	76000	0.0176±0.0078	529000	492000	496000	537000	0.0774±0.0185	499000	541000	469000	496000	0.1043±0.0745	C08493	--
kz001203	[M-H]-	160.04	116.00	161.05	C9H7NO2	Indole-3-carboxylic acid	Alkaloids	BioDeep	771-50-6	292000	321000	326000	362000	0.0054±0.0051	27900	33900	32100	29300	0.0083±0.0082	32900	34500	43000	37600	0.009±0.0114	446000	433000	468000	482000	0.0689±0.0261	546000	572000	599000	559000	0.1182±0.0209	-	-
kz001261	[M+H]+	104.07	68.80	103.06	C4H9NO2	γ-Aminobutyric acid	Organic acids	BioDeep	1956/12/2	9680	13200	12000	11600	0.0019±0.0021	67300	65000	65600	59200	0.0174±0.011	10400	7750	9570	9480	0.0023±0.0024	15700	14700	13400	17200	0.0023±0.0018	9050	11600	16700	9210	0.0024±0.0067	C00334	ko00250,ko00330,ko00410,ko00650,ko00760,ko01100
kz001264	[M-H]-	103.04	59.10	104.05	C4H8O3	3-Hydroxybutyrate	Organic acids	BioDeep	300-85-6	71100000	74100000	83700000	74300000	1.2522±1.1462	10700000	7480000	9520000	9250000	2.4969±3.4274	10100000	110000000	10100000	11000000	2.5657±1.4596	11200000	8790000	9260000	10700000	1.5038±1.4845	7130000	7500000	9240000	7430000	1.6236±1.5659	-	-
kz001268	[M-H]-	111.01	67.00	112.02	C5H4O3	2-Furanoic acid	Organic acids	BioDeep	88-14-2	11300000	12100000	11300000	10500000	0.1866±0.1294	968000	952000	1020000	934000	0.262±0.0978	368000	339000	382000	348000	0.0873±0.0458	1000000	984000	973000	1090000	0.1524±0.0355	986000	1040000	1080000	1010000	0.2138±0.0381	C01546	ko01100
kz001275	[M-H]-	117.02	73.00	118.03	C4H6O4	Methylmalonic acid	Organic acids	BioDee	516-05-2	2490000	2780000	2650000	2520000	0.4309±0.1498	1610000	1690000	1640000	1630000	0.4445±0.1523	2750000	2600000	2730000	2790000	0.6606±0.1439	5330000	5280000	5380000	5570000	0.8126±0.1208	6550000	7010000	7060000	6800000	1.4247±0.3199	C02170	ko00240,ko00280,ko00640,ko01100

								p		00	00	00	00								00															
kz001276	[M-H]-	117.02	73.00	118.03	C4H6O4	Succinic acid	Organic acids	BioDeep	110-15-6	2490000	2670000	2230000	2550000	0.4104±0.3561	15800000	16700000	16500000	16600000	0.4438±0.1359	2780000	2810000	2920000	2770000	0.6857±0.2393	5500000	5380000	5420000	5700000	0.8291±0.0539	6540000	6820000	7170000	6970000	1.429±0.4385	C00042	ko00020,ko00190,ko00250,ko00310,ko00350,ko00360,ko00620,ko00630,ko00640,ko00650,ko00760,ko00920,ko01100,ko01110,ko01200
kz001279	[M+H]+	120.07	74.10	119.06	C4H9NO3	L-Homoserine	Organic acids	BioDeep	672-15-1	2460000	2750000	2430000	2780000	0.043±0.0281	3640000	3620000	3780000	3430000	0.0979±0.0435	102000	107000	97100	89000	0.024±0.0202	321000	325000	312000	360000	0.0496±0.018	220000	239000	548000	225000	0.0634±0.3102	C00263	ko00260,ko00270,ko00300,ko00920,ko01100,ko01110,ko01230
kz001280	[M-H]-	124.01	80.00	125.01	C2H7NO3S	2-Aminoethanesulfonic acid	Organic acids	BioDeep	107-35-7	4040	4140	3690	3910	0.0007±0.0005	3650	4670	4600	2890	0.0011±0.0024	4810	4200	2920	2890	0.0009±0.0023	6350	6680	7480	9550	0.0011±0.0019	7170	6520	8070	7750	0.0052±0.0734	C00245	ko00430,ko00920,ko01100,ko02010
kz001281	[M-H]-	129.02	85.00	130.03	C5H6O4	Citraconic acid	Organic acids	BioDeep	498-23-7	13400000	15300000	14800000	14300000	2.3846±0.7091	40300000	40100000	40600000	40800000	1.0944±0.0987	426000	378000	475000	475000	0.1066±0.1101	1260000	1300000	1240000	1170000	0.1875±0.1286	3110000	3400000	3260000	2960000	0.6613±0.3079	C02226	ko00290,ko00660,ko01100,ko01210
kz001283	[M+H]+	132.10	69.00	131.09	C6H13NO2	6-Aminocaproic acid	Organic acids	BioDeep	60-32-2	33200	31800	33200	36200	0.0056±0.005	98500	94100	92700	93500	0.0256±0.0091	31500	29700	31300	31700	0.0075±0.0016	128000	122000	112000	128000	0.0185±0.0085	98000	88800	175000	87900	0.0232±0.0799	C02378	ko01100
kz00	[M-	13	59.	132.0	C4H4O5	Oxaloacetic acid	Organic anic	Bio	328-	-	-	-	-	-	2240	3430	1650	1270	0.0058±0.0	30500	31	3900	48400	0.0091±0.020	38500	39600	37400	34100	0.0056±0.0	58500	6330	5920	5960	0.0125±0.005	C000	ko00010,ko00020,ko00

1284	H]-	1.00	20	1			acids	Deep	42-7						0	0	0	0	267		200	0		2					049		0	0	0	2	36	250,ko00620,ko00630,ko00710,ko01100,ko01110,ko01200,ko01210,ko01230
kz001285	[M-H]-	131.03	87.00	132.04	C5H8O4	2-Methylsuccinic acid	Organic acids	BioDeep	498-21-5	54200000	64500000	61300000	58900000	0.0985±0.0469	20200000	19300000	17200000	15300000	0.0488±0.0653	302000	272000	251000	253000	0.0655±0.0519	700000	665000	706000	722000	0.1053±0.0288	594000	711000	672000	623000	0.135±0.0905	-	-
kz001287	[M-H]-	131.07	85.00	132.08	C6H12O3	(S)-(-)-2-Hydroxyisocaproic acid	Organic acids	BioDeep	13748-90-8	29600000	30200000	30000000	30000000	0.0495±0.019	58000000	61100000	58300000	64400000	0.1635±0.08	85200	65500	80000	61000	0.0177±0.0268	476000	514000	506000	566000	0.0777±0.0424	240000	259000	319000	262000	0.056±0.0571	-	-
kz001289	[M-H]-	133.01	71.00	134.02	C4H6O5	Malic acid	Organic acids	BioDeep	636-61-3	64900000	98800000	76600000	70800000	0.1279±0.2466	48700000	41600000	49300000	53900000	0.1307±0.1187	149000	1320000	172000	164000	0.0375±0.0424	1550000	1510000	1530000	1640000	0.2347±0.025	865000	914000	1070000	923000	0.1958±0.1381	-	-
kz001291	[M-H]-	141.02	59.00	142.03	C6H6O4	Trans,trans-Muconic acid	Organic acids	BioDeep	3588-17-8	1040000	965000	997000	1040000	0.1672±0.1469	11700000	12100000	12200000	11500000	0.3214±0.1191	1100000	1070000	1130000	1090000	0.2668±0.0679	1430000	1420000	1440000	1480000	0.2175±0.0351	1110000	1120000	1790000	1210000	0.2708±0.6002	-	-
kz001292	[M+H]+	146.08	86.00	145.07	C6H11NO3	4-Acetamidobutyric acid	Organic acids	BioDeep	3025-96-5	2130000	2650000	2220000	2230000	0.038±0.0321	13600000	14700000	14200000	14300000	0.0384±0.0158	92900	108000	98500	89300	0.0236±0.023	1310000	1220000	1220000	1320000	0.191±0.0468	177000	189000	229000	217000	0.0422±0.0458	C02946	ko00330,ko01100
kz001293	[M+H]+	146.0	87.00	145.09	C5H11N3O2	4-Guanidinobutyric acid	Organic acids	BioDe	463-00-3	1330	1420	1160	1380	0.2184±0.2248	5670000	5790000	6200000	5820000	0.1588±0.0547	1670000	1660	1790000	1710000	0.4152±0.1692	4950000	5080000	4990000	5480000	0.7723±0.1947	2550000	2610000	2640000	2510000	0.5358±0.0475	C01035	ko00330,ko01100

		9						ep		000	000	000	000								000															
kz001294	[M-H]-	145.05	101.00	146.06	C6H10O4	Adipic Acid	Organic acids	BioDeep	124-04-9	55100	50900	62900	57600	0.0094±0.0122	707000	672000	474000	978000	0.0191±0.0549	82000	949000	62400	82400	0.0196±0.0334	80200	49100	92600	63100	0.0108±0.0302	129000	152000	1220000	142000	0.0834±1.0756	C06104	ko01100
kz001295	[M-H]-	145.05	101.00	146.06	C6H10O4	2-Methylglutaric acid	Organic acids	BioDeep	617-62-9	61200	72700	65400	67000	0.011±0.0046	707000	695000	401000	938000	0.0185±0.0589	89100	88500	78100	74400	0.0201±0.0181	50600	46300	60100	48700	0.0078±0.0104	104000	97700	1150000	124000	0.0748±1.0395	-	-
kz001297	[M-H]-	147.07	59.00	148.07	C6H12O4	(Rs)-Mevalonic acid	Organic acids	BioDeep	150-97-0	10900000	12200000	12200000	12000000	1.9518±0.6631	43100000	44200000	44200000	44300000	1.1892±0.2275	510000	360000	409000	401000	0.102±0.1401	1090000	1100000	1050000	1070000	0.1625±0.0583	2000000	2080000	1960000	1980000	0.417±0.1636	-	-
kz001298	[M-H]-	149.01	87.00	150.02	C4H6O6	L-(+)-Tartaric acid	Organic acids	BioDeep	87-69-4	234000	310000	307000	324000	0.0484±0.0679	2080000	2230000	2050000	2170000	0.0577±0.029	69800	568000	65100	64600	0.0156±0.0111	2620000	2870000	2740000	3060000	0.4253±0.2192	337000	363000	333000	314000	0.07±0.0399	C00898	ko00630,ko01100
kz001302	[M-H]-	153.02	109.00	154.03	C7H6O4	2,3-Dihydroxybenzoic Acid	Organic acids	BioDeep	303-38-8	6600000	7700000	7190000	7190000	1.1829±0.3626	11200000	11300000	11200000	12100000	3.0974±1.0214	2090000	1810000	1900000	1890000	0.4671±0.2245	8890000	9050000	8800000	9660000	1.3713±0.2637	8000000	8370000	7510000	7640000	1.639±0.9834	C00196	ko01110
kz001307	[M-H]-	165.02	77.00	166.03	C8H6O4	Phthalic acid	Organic acids	BioDeep	88-99-3	18000	33700	29900	21600	0.0042±0.0133	-	-	-	-	-	-	-	-	-	-	37100	56400	51500	47900	0.0073±0.0133	35200	21500	29000	18600	0.0054±0.0156	C01606	ko01100,ko02010

kz001308	[M-H]-	165.04	75.00	166.05	C5H10O6	D-Xylonic acid	Organic acids	BioDeep	526-91-0	3270000	4700000	4110000	4050000	0.6637±0.9029	1330000	1340000	1590000	1460000	0.3866±0.2862	9060000	8520000	8270000	8430000	2.083±0.6307	6410000	6650000	6820000	7140000	1.0182±0.3668	5100000	4780000	5090000	4790000	1.027±0.3636	C00502	ko00040,ko01100
kz001311	[M+H]+	175.02	99.00	174.02	C6H6O6	Trans-Citridic acid	Organic acids	BioDeep	4023-65-8	376000	413000	362000	274000	0.0588±0.1202	351000	308000	317000	261000	0.0837±0.1055	307000	179000	290000	181000	0.0581±0.163	233000	172000	212000	219000	0.0315±0.0369	229000	226000	233000	220000	0.0472±0.0078	C02341	ko00660,ko01100
kz001312	[M-H]-	173.05	93.00	174.05	C7H10O5	Shikimic acid	Organic acids	BioDeep	138-59-0	1030000	893000	985000	824000	0.1545±0.2519	121000	123000	119000	85600	0.0304±0.051	501000	418000	467000	427000	0.1101±0.0822	243000	255000	252000	228000	0.0369±0.0284	160000	155000	165000	152000	0.0328±0.0076	C04493	ko00400,ko01100,ko01110,ko01230
kz001313	[M-H]-	173.08	111.00	174.09	C8H14O4	SubericAcid	Organic acids	BioDeep	505-48-6	815000	1060000	1070000	949000	0.016±0.0191	102000	81500	95700	81400	0.0244±0.0276	93500	921000	109000	104000	0.0242±0.0207	123000	82600	83200	98800	0.0146±0.0274	264000	221000	447000	228000	0.0599±0.2027	C08278	--
kz001315	[M-H]-	187.10	125.00	188.10	C9H16O4	Anchoic Acid	Organic acids	BioDeep	123-99-9	3830000	4150000	3850000	4080000	0.6568±0.1837	2780000	2710000	2830000	2780000	0.7507±0.0684	4670000	4640000	4590000	4760000	1.1341±0.1719	3650000	3620000	3760000	3800000	0.559±0.1438	9710000	9480000	13000000	9730000	2.1733±2.9357	C08261	--
kz001317	[M-H]-	191.02	111.01	192.03	C6H8O7	Citric Acid	Organic acids	BioDeep	77-92-9	854000	641000	669000	569000	0.1134±0.3043	6040000	8480000	8610000	1010000	0.2246±0.4411	1370000	1070000	1130000	1150000	0.2866±0.2766	1090000	1040000	1240000	1220000	0.173±0.1419	908000	597000	520000	509000	0.132±0.4066	C00158	ko00020,ko00250,ko00630,ko01100,ko01110,ko01200,ko01210,ko01230
kz00	[M-	19	85.	192.0	C7H12O	Quinic Acid	Organic	Bio	77-	40	43	42	46	0.7109±0.3	2120	2190	2150	2220	0.5871±0.1	798000	74	7830	707000	1.8458±0.957	536000	534000	549000	545000	0.0816±0.0	1120000	1220	1100	1100	0.236±0.1308	C002	ko00400,ko01100

1318	H]-	1.06	00	6	6		acids	Deep	95-2	70000	40000	00000	10000	938	000	000	000	000	461	0	90000	000	0	3					245		000	000	000		96	
kz001319	[M-H]-	193.04	72.80	194.04	C6H10O7	D-Galacturonic acid(GalA)	Organic acids	BioDeep	685-73-4	4110000	4900000	5230000	5930000	0.0831±0.1409	685000	600000	656000	597000	0.1717±0.1159	473000	500000	489000	491000	0.1187±0.044	13700000	13100000	13000000	15400000	2.0777±1.0409	5900000	622000	603000	600000	1.2553±0.353	C00333	ko00040,ko00053,ko00520,ko01100,ko02010
kz001325	[M-H]-	229.14	211.00	230.15	C12H22O4	Dodecanedioic aicd	Organic acids	BioDeep	693-23-2	8730	10300	9270	9760	0.0016±0.0007	9830	8780	8740	9200	0.0025±0.0014	11200	11500	10900	10700	0.0027±0.0011	13200	13400	12600	12400	0.0019±0.0011	13800	1250	2190	1280	0.0032±0.0084	C02678	--
kz001331	[M+H]+	577.13	287.00	576.13	C30H24O12	Procyanidin A1	Flavonoids	BioDeep	103883-03-0	27200	35600	34800	31100	0.0053±0.006	5190	4980	5200	4950	0.0137±0.0038	13600	11900	2270	22700	0.0043±0.0141	44300	43500	37600	35200	0.0061±0.0077	-	-	-	-	-	-	-
kz001332	[M-H]-	577.14	407.10	578.14	C30H26O12	Procyanidin B2	Flavonoids	BioDeep	29106-49-8	1190000	1320000	1240000	1410000	0.2129±0.1546	469000	462000	484000	444000	0.1258±0.0495	2330000	2080000	2170000	2270000	0.5376±0.1887	1200000	1170000	1200000	1380000	0.1863±0.0921	-	-	-	-	-	C17639	--
kz001333	[M-H]-	577.14	407.10	578.14	C30H26O12	Procyanidin B3	Flavonoids	BioDeep	23567-23-9	9010000	9470000	8710000	9340000	0.1509±0.0671	355000	305000	328000	331000	0.0892±0.0507	1730000	1550000	1620000	1770000	0.4052±0.195	917000	889000	871000	990000	0.1381±0.0379	-	-	-	-	-	-	-
kz001335	[M-H]-	577.14	425.10	578.14	C30H26O12	Procyanidin B1	Flavonoids	BioDee	20315-25-7	99300	10500	10200	10800	0.1711±0.0611	387000	340000	373000	334000	0.097±0.0691	1890000	16400	1740000	1850000	0.4325±0.2122	987000	958000	965000	1070000	0.1499±0.034	-	-	-	-	-	-	-

								p		0	00	00	00								00															
kz001340	[M+H]+	867.21	577.13	866.21	C45H38O18	Procyanidin C2	Flavonoids	BioDeep	37064-31-6	73400	84700	72200	81500	0.0129±0.0086	33500	36800	40900	28100	0.0094±0.015	229000	237000	224000	218000	0.0552±0.0243	49200	68900	63600	84200	0.01±0.0198	-	-	-	-	-	-	-
kz001380	[M+H]+	757.22	303.05	756.21	C33H40O20	Quercetin-O-rutinoside-O-rhamnoside	Flavonoids	BioDeep	-	8630	6660	3420	7390	0.0011±0.0049	5900	4710	6820	7840	0.0017±0.0034	216000	170000	160000	222000	0.0466±0.0708	10200	6680	8430	6760	0.0012±0.0026	175000	162000	162000	146000	0.0335±0.025	-	-
kz001562	[M+H]+	127.04	97.00	126.03	C6H6O3	5-Hydroxymethylfurfural	Phenolic acids	BioDeep	-	19500000	19100000	19900000	18400000	0.3179±0.2362	1520000	1610000	1720000	1660000	0.4402±0.2012	2390000	24500000	2220000	2210000	0.5635±0.317	2100000	2140000	2070000	2070000	0.316±0.1206	2380000	2660000	2540000	2700000	0.5345±0.3441	C11101	--
kz001581	[M+H]+	479.15	317.06	478.15	C23H26O11	Calceorioside B	Others	BioDeep	105471-98-5	11400000	12900000	12000000	11400000	0.1968±0.0883	403000	459000	437000	409000	0.1156±0.0837	1340000	13200000	1490000	1390000	0.3368±0.2008	1540000	1280000	1290000	1460000	0.2098±0.1636	106000	67700	117000	113000	0.021±0.047	-	-
kz001584	[M+H]+	331.08	287.05	330.07	C17H14O7	Quercetin-3',4'-dimethyl ether	Flavonoids	BioDeep	33306-29-4	17600	30300	28900	23200	0.0041±0.0105	5000	6880	5920	7800	0.0017±0.0032	-	-	-	-	-	28600	33800	36500	33500	0.005±0.0056	-	-	-	-	-	-	-
kz001586	[M+H]+	479.12	317.06	478.11	C22H22O12	Isotamarixin	Flavonoids	BioDeep	-	31400	30000	22700	23200	0.0044±0.0102	9220	22100	15700	9520	0.0038±0.0171	18800	13200	18700	14700	0.004±0.0066	106000	99500	108000	79000	0.0148±0.0234	177000	168000	220000	182000	0.0388±0.0394	-	-
kz00	[M+H]	14	84.	143.0	C7H13N	Stachydrine	Alkaloid	Bio	-	90	95	85	85	0.1469±0.0	1030	9890	1050	1020	0.2765±0.0	397000	37	4400	409000	0.0983±0.069	101000	861000	830000	893000	0.1354±0.1	476000	4780	5210	4920	0.1022±0.029	C101	--

1598	+	4.10	10	9	O2		s	Deep		0000	2000	4000	2000	983	000	00	000	000	439		2000	00		5	0				14		00	00	00	1	72	
kz001637	[M+H]+	213.08	167.07	212.07	C10H12O5	Oresbiusin A	Phenolic acids	BioDeep	-	264000	265000	272000	293000	0.0045±0.0032	44400	32200	35100	33200	0.0098±0.0152	226000	202000	236000	212000	0.0532±0.035	414000	419000	423000	371000	0.0614±0.0529	126000	137000	138000	137000	0.028±0.0109	-	-
kz001642	[M-H]-	387.17	207.10	388.17	C18H28O9	5'-Glucopyranosyloxyjasmanic acid	Phenolic acids	BioDeep	-	413000	388000	398000	422000	0.0067±0.0056	70900	80100	89400	82200	0.0218±0.0194	41000	33200	38600	45100	0.0096±0.0111	70600	74300	66200	62900	0.0103±0.0096	157000	154000	137000	145000	0.0309±0.0243	-	-
kz001671	[M-H]-	299.06	284.04	300.06	C16H12O6	5,7,2'-Trhiyroxy-8-methoxyflavone	Flavonoids	BioDeep	-	270000	295000	247000	261000	0.0044±0.0037	13800	16500	16700	17100	0.0043±0.004	21400	16300	19800	20300	0.0047±0.0048	47900	47300	44300	52500	0.0072±0.0031	9180	7200	8850	7680	0.0017±0.0019	-	-
kz001680	[M+H]+	329.10	268.08	328.09	C18H16O6	6-hydroxy-5,7,4'-trimethoxyflavone	Flavonoids	BioDeep	-	-	-	-	-	-	-	-	-	-	-	-	-	-	-	-	35800	35500	33500	36700	0.0053±0.0012	-	-	-	-	-	C14985	--
kz001684	[M+H]+	343.12	299.09	342.11	C19H18O6	Tetramethylluteolin (3',4',5,7-Tetramethoxyflavone)	Flavonoids	BioDeep	-	7450	8340	7820	8170	0.0013±0.0003	-	-	-	-	-	-	-	-	-	-	-	-	-	-	-	-	-	-	-	-	-	-
kz001695	[M+H]+	461.10	285.08	460.10	C22H20O11	wogonoside	Flavonoids	BioDeep	-	188000	208000	225000	232000	0.0035±0.0035	6610	6900	5630	5790	0.0017±0.0019	-	-	-	-	-	13600	11400	14200	13500	0.002±0.0019	-	-	-	-	-	-	-
kz00	[M-	46	28	462.0	C21H18	Kaempferol-3-O-β-	Flavonoi	Bio	-	62	63	63	69	0.0107±0.0	2730	2510	1810	2650	0.0066±0.0	354000	30	3130	349000	0.8048±0.457	988000	102000	1020000	108000	0.1548±0.0	865000	9450	8920	8760	0.186±0.0718	-	-

1702	H]-	1.07	5.04	8	O12	D-glucuronide	ds	Deep		000	600	600	200	068	0	0	0	0	116	0	90000	000	0	1		0		0	388		00	00	00			
kz001706	[M+H]+	197.12	161.10	196.11	C11H16O3	Isololiolide	Others	BioDeep	38274-00-9	53200	64400	68300	65400	0.0104±0.0109	51600	54600	52400	49500	0.0141±0.0077	15200	12400	13800	11200	0.0032±0.004	99200	98400	98100	104000	0.0151±0.008	261000	254000	243000	254000	0.0526±0.0279	-	-
kz001710	[M+H]+	229.12	201.13	228.12	C15H16O2	Stilbostemin B	Others	BioDeep	162411-67-8	13500	15800	12700	13200	0.0023±0.0022	9690	6600	11000	8800	0.0024±0.0048	6100	5430	7310	4810	0.0014±0.0026	4950	5310	7630	6400	0.0009±0.0019	6650	4860	6170	6210	0.0012±0.0017	-	-
kz001726	[M-H]-	811.45	487.34	812.46	C42H68O15	Prunellin A	Others	BioDeep	-	43300	46500	38800	40800	0.007±0.0066	22100	23500	30500	31900	0.0073±0.0123	-	-	-	-	-	-	-	-	-	-	-	-	-	-	-	-	-
kz001739	[M+H]+	617.11	303.05	616.11	C28H24O16	Quercetin-3-O-(2''-galloyl)-β-D-glucoside	Flavonoids	BioDeep	-	919000	1110000	933000	1100000	0.1674±0.1563	350000	335000	333000	352000	0.0927±0.0254	14700	16600	12900	5800	0.003±0.0116	1960000	1960000	2030000	2050000	0.3016±0.0812	28800	30700	18000	13700	0.0047±0.0173	-	-
kz001740	[M+H]+	769.09	303.05	768.12	C35H28O20	Quercetin-3-O-(2'',3''-digalloyl)-β-D-glucopyranoside	Flavonoids	BioDeep	-	18100	34900	36700	24600	0.0047±0.0168	13300	9960	13300	18200	0.0037±0.0087	-	-	-	-	-	20300	25800	35600	41200	0.0046±0.0135	15000	18600	13600	16900	0.0033±0.0049	-	-
kz001742	[M-H]-	305.03	273.00	306.04	C14H10O8	Methyl Brevifolin carboxylate	Others	BioDeep	154702-76	13500	22200	23100	19500	0.3218±0.7929	604000	533000	406000	584000	0.1439±0.2445	392000	41100	281000	350000	0.0871±0.14	3230000	3230000	2830000	3200000	0.4706±0.2647	3840000	3260000	3410000	3110000	0.7081±0.6909	-	-

								p	-8	00	00	00	00								0															
kz001750	[M-H]-	785.09	301.00	786.09	C34H26O22	Tercatain	Phenolic acids	BioDeep	103744-87-2	248000	229000	376000	421000	0.0053±0.0194	-	-	-	-	-	-	-	-	-	-	-	-	-	-	-	-	-	-	-	-	-	-
kz001819	[M-H]-	137.03	108.02	138.03	C7H6O3	Salicylic acid	Phenolic acids	BioDeep	-	507000	423000	415000	583000	0.008±0.0179	603000	649000	599000	719000	0.0174±0.0146	89100	76800	66000	67200	0.0182±0.0244	106000	92300	87100	99800	0.0145±0.0107	137000	137000	159000	134000	0.0294±0.0166	C00805	ko00360,ko01100,ko01110,ko04075
kz001820	[M-H]-	237.04	121.03	238.05	C11H10O6	benzoylmalic acid	Phenolic acids	BioDeep	-	1340000	1420000	1190000	1170000	0.0211±0.0252	-	-	-	-	-	-	-	-	-	-	-	-	-	-	-	-	-	-	-	-	-	-
kz001824	[M-H]-	279.05	147.04	280.06	C13H12O7	cinnamoyl tartaric acid	Phenolic acids	BioDeep	-	1030000	1120000	1040000	865000	0.0167±0.0216	55500	66000	56500	59400	0.0161±0.0149	-	-	-	-	-	-	-	-	-	-	-	-	-	-	-	-	-
kz001834	[M-H]-	309.06	193.05	310.07	C14H14O8	feruloylmalic acid	Phenolic acids	BioDeep	-	-	-	-	-	-	-	-	-	-	-	-	-	-	-	-	-	-	-	-	-	68600	81100	75000	68500	0.0152±0.0107	-	-
kz001864	[M-H]-	461.07	167.04	462.08	C21H18O12	vnilloylcaffeoyltartaric acid	Phenolic acids	BioDeep	-	-	-	-	-	-	-	-	-	-	-	31400	24600	27700	21500	0.0064±0.0098	66600	62100	61200	77000	0.01±0.0078	-	-	-	-	-	-	-
kz001880	[M-H]-	531.1	337.0	532.12	C25H24O13	feruloylsinapoyltartaric acid	Phenolic acid	BioDe	-	3190	3250	2690	2840	0.0049±0.0061	111000	160000	165000	144000	0.0039±0.0067	20700	18100	16700	19900	0.0046±0.0038	151000	161000	148000	181000	0.0241±0.0164	21900	15100	22600	20400	0.0042±0.007	-	-

		2	6				s	ep		0	0	0	0								0															
kz001973	[M+H]+	903.26	287.066	902.25	C42H46O22	Kaempferol-3-O-(cinnamoyl)-sophoroside-7-O-glucose	Flavonoids	BioDeep	-	9030	10900	9010	9290	0.0016±0.0013	3280	3840	4360	3650	0.001±0.0012	-	-	-	-	-	4080	4630	4060	5520	0.0007±0.0009	29300	2600	2460	2460	0.0054±0.0053	-	-
kz002008	[M+H]+	303.04	257.04	302.04	C15H10O7	3,5,7,4'--Tetrahydroxy-Coumaronochromone	Others	BioDeep	-	183000	192000	255000	199000	0.0342±0.067	43600	39900	46200	42800	0.0117±0.0057	69900	43300	5490	44800	0.0129±0.0281	238000	124000	117000	165000	0.0242±0.082	71400	9270	9750	7490	0.0174±0.0226	-	-
kz002063	[M-H]-	633.08	300.99	634.08	C27H22O18	Galloyl-HHDP(Hexahydroxydiphenoyl)-glueopyranose	Phenolic acids	BioDeep	-	6660000	6400000	6350000	7750000	0.1122±0.1461	61600	39000	70800	70900	0.0163±0.0385	-	-	-	-	-	70700	132000	58400	59100	0.0121±0.055	191000	158000	153000	150000	0.0339±0.0435	-	-
kz002065	[M-H]-	447.06	315.01	448.06	C20H16O12	4-α-L-Rhamnopyranosyl-ellagic acid	Phenolic acids	BioDeep	-	4090000	4370000	3970000	3970000	0.0677±0.0354	279000	336000	338000	312000	0.0856±0.0776	473000	530000	595000	527000	0.1293±0.1352	2270000	2490000	2540000	2240000	0.3601±0.3218	6610000	6110000	5870000	6170000	1.2881±0.9131	-	-
kz002144	[M+H]+	187.04	131.00	186.03	C11H6O3	Angelicin	Coumarins	BioDeep	523-50-2	-	-	-	-	-	-	-	-	-	-	55700	60300	7070	71600	0.0157±0.0197	-	-	-	-	-	-	-	-	-	-	C09060	ko01110
kz002164	[M+H]+	287.06	241.05	286.05	C15H10O6	Isoluteolin(Orobol)(5,7,3',4'-tetrahydroxyisoflavone)	Flavonoids	BioDeep	480-23-9	5120	6560	6640	7290	0.0011±0.0016	4970	5510	5140	5770	0.0014±0.001	17100	10700	1490	9540	0.0032±0.0083	48800	43200	22600	43700	0.0059±0.017	25200	2060	1690	2570	0.0046±0.0098	C10510	--
kz0021	[M+H]+	625.	317.	624.17	C28H32O16	2'-Hydoxy,5-methoxy	Flavonoids	BioD	-	540	594	523	586	0.0093±0.0053	97600	131000	115000	133000	0.0322±0.0453	567000	728	47800	441000	0.1347±0.3232	148000	192000	124000	143000	0.0229±0.0456	35300	4200	6350	4560	0.0097±0.0226	-	-

96		17	06			Genistein-O-rhamnosyl-glucoside		eep		00	00	00	00								000															
kz002197	[M+H]+	641.16	317.066	640.16	C28H32O17	2'-Hydoxy,5-methoxy Genistein-4',7-O-diglucoside	Flavonoids	BioDeep	-	12400	15300	12100	12300	0.0021±0.0024	52500	58700	58100	48600	0.0147±0.0144	-	-	-	-	-	119000	114000	85900	100000	0.0158±0.0229	40100	31900	50700	56100	0.0093±0.0229	-	-
kz002202	[M+H]+	463.16	301.11	462.15	C23H26O10	Farrerol 7-O-glucoside	Flavonoids	BioDeep	-	9930	10900	11300	12600	0.0018±0.002	83500	85100	88600	82100	0.023±0.0084	-	-	-	-	-	-	-	-	-	-	-	-	-	-	-	-	-
kz002211	[M+H]+	435.09	303.06	434.08	C20H18O11	Quercetin 3-O-β-D-xylopyranoside	Flavonoids	BioDeep	549-32-6	1870000	1900000	1980000	1750000	0.3099±0.2435	1710000	1670000	1800000	1670000	0.4633±0.1482	-	-	-	-	-	1800000	1780000	1630000	2080000	0.2743±0.2031	261000	239000	266000	321000	0.0566±0.082	-	-
kz002212	[M-H]-	461.11	314.04	462.12	C22H22O11	Azalein (Azaleatin-3-O-rhamnoside)	Flavonoids	BioDeep	29028-02-2	37600	67200	37900	50200	0.0079±0.0251	36000	46300	39900	31300	0.0104±0.0186	-	-	-	-	-	-	-	-	-	-	-	-	-	-	-	-	-
kz002214	[M+H]+	465.10	303.06	464.10	C21H20O12	isohyperoside	Flavonoids	BioDeep	35589-21-0	6680000	7010000	6990000	6990000	1.1426±0.2979	1810000	1690000	1860000	1740000	0.4801±0.1686	2000000	2030000	2120000	2100000	0.5015±0.1779	4570000	4920000	4750000	5250000	0.7342±0.3207	2410000	2350000	2260000	2360000	0.4879±0.2484	-	-
kz002219	[M-H]-	357.13	151.04	358.14	C20H22O6	Epipinoresinol	Lignans	BioDee	24404-50-0	458000	494000	462000	421000	0.0758±0.0582	111000	112000	116000	116000	0.0308±0.0044	64100	57700	54600	63900	0.0146±0.0097	400000	433000	356000	440000	0.0613±0.0494	147000	154000	153000	146000	0.0312±0.005	-	-

								p		0	0	0	0																							
kz002220	[M-H]-	537.20	375.15	538.21	C26H34O12	Olivil-4'-O-β-D-glucoside	Lignans	BioDeep	76880-93-8	1480000	1670000	1550000	1550000	0.0258±0.0053	85800	81900	84700	81300	0.0226±0.0065	37700	32000	31300	28900	0.0079±0.0083	335000	335000	349000	345000	0.0514±0.0173	77100	77000	86200	85400	0.0169±0.0102	-	-
kz002221	[M-H]-	549.20	387.15	550.21	C27H34O12	Eucommin A	Lignans	BioDeep	99633-12-2	3900000	3400000	4370000	3980000	0.0648±0.0986	1140000	1240000	1180000	1210000	0.0323±0.0145	6340	8040	14600	6950	0.0022±0.0095	178000	185000	176000	162000	0.0265±0.0212	123000	120000	137000	114000	0.0256±0.0141	C10560	--
kz002223	[M-H]-	349.15	187.10	350.16	C15H26O9	Eucommia	Others	BioDeep	-	246000	258000	295000	303000	0.0045±0.0055	26700	33000	34900	38500	0.009±0.0127	16100	16300	18800	24300	0.0046±0.0092	15800	19400	18200	15100	0.0026±0.0037	96000	111000	93100	88900	0.0202±0.0197	-	-
kz002295	[M-H]-	579.21	519.24	580.22	C28H36O13	Syringaresinol-4'-O-β-D-monO-glucoside	Lignans	BioDeep	137038-13-2	378000	478000	446000	421000	0.0071±0.0056	-	-	-	-	-	215000	2360000	269000	309000	0.0626±0.1006	517000	457000	440000	502000	0.0722±0.0434	41000	53000	42200	31900	0.0087±0.0172	-	-
kz002337	[M+H]+	611.15	287.05	610.15	C27H30O16	Luteolin-7-O-β-D-gentiobioside	Flavonoids	BioDeep	-	157000	143000	211000	196000	0.0029±0.0068	4320	6770	11100	9730	0.0022±0.008	-	-	-	-	-	78100	73500	67900	103000	0.0121±0.0194	5840	7930	6700	6030	0.0014±0.0018	-	-
kz002367	[M+H]+	222.08	130.06	221.07	C11H11NO4	Methyl dioxindole-3-acetate	Others	BioDeep	57061-18-4	483000	616000	678000	639000	0.01±0.015	17200	26800	16900	20600	0.0055±0.0133	34500	34400	29800	27700	0.0077±0.0083	51500	47700	57100	55500	0.008±0.0063	-	-	-	-	-	-	-
kz002419	[M-H]-	580.36	506.33	581.26	C33H35N5O5	Ergotamine	Alkaloids	BioDee	113-15-5	819000	883000	845000	855000	1.4045±0.1575	18100000	16900000	18600000	18300000	4.8613±1.3237	9900000	946000	9680000	9770000	2.3585±0.1528	6740000	6310000	6350000	6820000	0.9879±0.2069	4440000	4500000	4320000	4000000	0.8969±0.4395	-	-

								p		00	00	00	00								00															
kz002485	[M-H]-	203.08	116.05	204.04	C11H8O4	3-methoxy-juglone	Quinones	BioDeep	-	9950	9400	6450	9400	0.0015±0.0035	7380	11400	9650	8520	0.0025±0.0049	33500	351000	41700	46000	0.0095±0.0143	138000	153000	125000	153000	0.0214±0.0179	1840	3950	6720	3410	0.0008±0.004	-	-
kz002486	[M-H]-	337.06	175.02	338.10	C16H18O8	1,4,8-trihydroxynaphthalene-1-O-β-D-glucopyranoside	Quinones	BioDeep	-	66400	70100	68400	71000	0.0114±0.0032	51500	41300	44100	44200	0.0122±0.0116	43200	458000	65700	48800	0.0124±0.0256	60000	60800	57000	71600	0.0094±0.0069	14700	11200	13100	14900	0.0028±0.0041	-	-
kz002546	[M-H]-	299.08	137.02	300.08	C13H16O8	Isosalicylic acid O-glycoside	Phenolic acids	BioDeep	88847-06-7	61200	68900	57400	60100	0.0102±0.0082	58300	63200	70600	60000	0.017±0.0144	355000	311000	350000	362000	0.0837±0.0474	234000	218000	208000	236000	0.0337±0.0134	48300	55600	66700	54800	0.0117±0.0131	-	-
kz002548	[M-H]-	325.09	163.04	326.10	C15H18O8	p-Coumaric acid-O-glycoside	Phenolic acids	BioDeep	-	593000	575000	546000	488000	0.0911±0.1174	50000	45400	62100	58800	0.0146±0.0188	91600	83100	84000	92200	0.0213±0.0089	294000	301000	272000	282000	0.0433±0.0241	96600	101000	99000	114000	0.0214±0.0199	-	-
kz002549	[M-H]-	329.09	209.04	330.10	C14H18O9	Vanillic acid glycoside	Phenolic acids	BioDeep	-	197000	155000	113000	147000	0.0025±0.0081	14600	12100	11900	17200	0.0038±0.0064	11100	16700	7180	11600	0.0028±0.0098	101000	113000	104000	107000	0.016±0.0094	19500	22000	19000	18700	0.0041±0.0032	-	-
kz002551	[M-H]-	385.11	223.06	386.12	C17H22O10	Isosinapic acid-hexoside	Phenolic acids	BioDeep	-	684000	525000	647000	602000	0.0102±0.0188	83500	93100	95000	117000	0.0263±0.0359	79000	75300	69500	73100	0.018±0.0084	51500	38000	45900	40800	0.0066±0.0094	-	-	-	-	-	-	-
kz0025	[M-H]-	385.	223.	386.12	C17H22O10	Sinapic acid-glycoside	Phenolic	BioD	-	239	317	315	287	0.0048±0.0057	13900	13500	13000	17500	0.0039±0.0052	66900	725	7330	78900	0.0177±0.0129	127000	121000	121000	112000	0.0181±0.0132	45900	40800	40000	39700	0.0087±0.0072	-	-

52		11	06				acids	eep		00	00	00	00								00															
kz002554	[M+H]+	165.05	147.04	164.05	C9H8O3	p-Hydroxycinnamic acid	Phenolic acids	BioDeep	-	1670000	1840000	1930000	1790000	0.0298±0.0173	22000	22000	35300	34900	0.0077±0.0194	49200	486000	42800	52000	0.0117±0.0088	108000	90500	96400	96300	0.0147±0.0113	105000	114000	117000	105000	0.0229±0.0074	-	-
kz002556	[M-H]-	299.08	137.02	300.08	C13H16O8	4-O-glucosyl-4-hydroxybenzoic acid	Phenolic acids	BioDeep	33228-65-8	660000	759000	631000	584000	0.0109±0.0135	61900	67000	66300	70000	0.0179±0.0086	337000	3060000	317000	361000	0.0802±0.0509	190000	248000	198000	221000	0.0323±0.0406	46600	63200	68200	65900	0.0127±0.0192	-	-
kz002557	[M-H]-	301.09	139.05	302.10	C13H18O8	4-O-glucosyl-3,4-dihydroxybenzyl alcohol	Phenolic acids	BioDeep	38934-20-2	671000	692000	800000	615000	0.0115±0.0166	20200	17900	20300	18200	0.0052±0.0032	145000	1340000	142000	153000	0.0349±0.016	89400	90000	98600	87100	0.0138±0.0109	-	-	-	-	-	-	-
kz002558	[M-H]-	341.09	179.03	342.10	C15H18O9	6-O-caffeoyl-β-glucose	Phenolic acids	BioDeep	-	8670000	9080000	7960000	7340000	0.1365±0.1663	706000	651000	665000	671000	0.1821±0.0632	423000	4770000	428000	484000	0.1102±0.0853	953000	927000	1010000	974000	0.1457±0.0688	333000	327000	306000	319000	0.0668±0.0382	-	-
kz002560	[M-H]-	355.10	193.05	356.11	C16H20O9	6-O-feruloyl-α-glucose	Phenolic acids	BioDeep	-	249000	105000	262000	184000	0.0033±0.0159	26000	22800	31100	28600	0.0073±0.0086	265000	2970000	277000	310000	0.0699±0.0535	122000	121000	118000	138000	0.0188±0.008	18200	18400	15600	11300	0.0033±0.0068	-	-
kz002604	[M-H]-	489.10	285.04	490.11	C23H22O12	Kaempferol-3-O-(6''-acetyl)-glucoside	Flavonoids	BioDeep	-	323000	371000	316000	329000	0.0055±0.0035	12600	9540	12700	13600	0.0033±0.0044	251000	2140000	230000	249000	0.0573±0.0345	57800	51300	55700	57700	0.0084±0.0038	41500	41100	31400	29300	0.0075±0.0137	-	-
kz0026	[M-H]-	521.	359.	522.21	C26H34O11	Lariciresinol glucopyra	Others	BioD	-	177	202	184	195	0.0313±0.0101	83800	73100	89500	93200	0.0229±0.0202	-	-	-	-	-	60800	65300	68300	48200	0.0092±0.0157	28300	27300	36300	24800	0.006±0.0087	-	-

07		20	15			noside		eep		000	000	000	000																							
kz002665	[M-H]-	455.35	455.34	456.36	C30H48O3	Betulinic acid	Terpenoids	BioDeep	472-15-1	1220	2200	558	806	0.0002±0.0014	4850	3440	3400	5190	0.0011±0.0024	621	1070	660	1130	0.0002±0.0007	9950	7390	8520	5090	0.0012±0.0033	8690	10100	13500	7730	0.0021±0.0046	C08619	--
kz002668	[M+H]+	459.35	441.33	458.34	C30H48O4	Ovalifoliolides B	Terpenoids	BioDeep	-	18000	19700	16700	15900	0.0029±0.0032	17100	17000	17300	15600	0.0045±0.0025	-	-	-	-	-	3950	2930	4810	6110	0.0007±0.0019	-	-	-	-	-	-	-
kz002673	[M-H]-	449.11	287.06	450.12	C21H22O11	Eriodictyol-O-glucoside	Flavonoids	BioDeep	-	3230000	3660000	3220000	3390000	0.0557±0.0238	1290000	1320000	1420000	1410000	0.3678±0.1353	119000	116000	120000	131000	0.0295±0.0149	1590000	1540000	1560000	1620000	0.2378±0.0299	40100	44900	51300	39100	0.0091±0.009	-	-
kz002678	[M-H]-	505.10	300.03	506.11	C23H22O13	Quercetin-3-O-(6''-O-acetyl)-galactoside	Flavonoids	BioDeep	-	955000	887000	830000	813000	0.0144±0.0184	171000	154000	146000	204000	0.0046±0.0066	229000	231000	225000	228000	0.0555±0.0107	22300	20100	22300	20600	0.0032±0.0022	26400	23900	23500	18800	0.0048±0.0065	-	-
kz002687	[M-H]-	535.18	373.13	536.19	C26H32O12	Nortrachelogenin 4-O-β-D-glucoside	Lignans	BioDeep	-	147000	162000	176000	151000	0.0263±0.0241	65000	62700	81600	73600	0.0191±0.0209	40200	30000	28500	39600	0.0084±0.0139	46500	46000	41300	54400	0.0071±0.0061	38400	26100	19100	32700	0.0061±0.0186	-	-
kz002783	[M+H]+	595.14	287.06	594.14	C30H26O13	Kaempferol 3-O-β-d-(6''-O-(E)-p-coumaroyl) glucopyranoside	Flavonoids	BioDeep	-	23000000	23900000	22500000	24600000	0.3882±0.1963	23300000	20900000	27500000	24300000	0.6486±0.657	3950000	3910000	3680000	3610000	0.9208±0.4153	12500000	11300000	11800000	12300000	1.805±0.6448	5850000	5970000	6620000	5920000	1.265±0.4365	-	-
kz	[M	6	3	62	C28	Sexangula	Flav	Bi	-	5	5	6	4	0.008	13	98	13	13	0.033	446	5	59	836	0.1446	122	145	1790	147	0.022	3070	35	43	46	0.0081	-	-

002788	+H]+	25.18	17.07	4.17	H32O16	retin 3-glucoside-7-rhamnoside	onoids	oDeep		4800	2900	2700	5000	9±0.0163	3000	300	3000	1000	5±0.0421	000	03000	4000	000	±0.4155	000	000	00	000	4±0.0385	0	000	800	800	±0.0156		
kz002807	[M-H]-	131.03	87.05	132.04	C5H8O4	Dimethyl malonic acid	Organic acids	BioDeep	595-46-0	43100	52700	51300	51000	0.0082±0.0057	14800	15500	23200	14700	0.0046±0.0108	33500	24600	23200	23900	0.0064±0.0109	69400	52900	58700	54600	0.0089±0.0117	48300	52100	67400	49800	0.0113±0.0152	-	-
kz002820	[M+H]+	509.13	347.07	508.12	C23H24O13	Limocitrin 7-glucoside	Flavonoids	BioDeep	-	-	-	-	-	-	98400	116000	125000	102000	0.0299±0.0341	-	-	-	-	-	-	-	-	-	-	-	-	-	-	-	-	-
kz002829	[M+H]+	479.12	317.06	478.11	C22H22O12	Isorhamnetin-7-O-glucoside	Flavonoids	BioDeep	-	892000	1280000	1280000	1130000	0.1886±0.3111	4110000	4160000	4210000	3830000	0.1104±0.0562	1420000	1360000	1110000	1440000	0.3238±0.3486	1430000	1280000	1220000	1450000	0.2025±0.1248	118000	116000	105000	99300	0.0228±0.0197	-	-
kz002853	[M+H]+	144.14	84.08	143.13	C8H17NO	Dihydroisopelletierine	Alkaloids	BioDeep	-	579000	641000	620000	568000	0.0994±0.0526	592000	629000	570000	602000	0.1619±0.0883	202000	201000	177000	193000	0.047±0.0269	588000	519000	528000	563000	0.0828±0.0401	157000	153000	162000	154000	0.0325±0.0063	-	-
kz002862	[M+H]+	278.95	158.99	277.20	C17H27NO2	Dihydrosedinine	Alkaloids	BioDeep	-	258000	272000	248000	252000	0.0425±0.0219	2570000	2710000	2330000	2560000	0.0688±0.0516	601000	579000	557000	596000	0.1418±0.0353	234000	231000	211000	207000	0.0333±0.026	227000	207000	232000	181000	0.044±0.042	-	-
kz002898	[M+H]+	217.12	161.10	216.12	C14H16O2	Liguhodgsonal	Terpenoids	BioDeep	64185-18-8	12100	12100	12300	9660	0.0019±0.0029	11400	10200	10700	13200	0.0031±0.0032	13300	12400	12500	13000	0.0031±0.0006	13100	11400	13700	9920	0.0018±0.003	12300	11000	10500	11800	0.0024±0.0022	-	-

kz002910	[M+H]+	249.15	185.10	248.14	C15H20O3	Parthenolide	Terpenoids	BioDeep	20554-84-1	1220000	1430000	1150000	1270000	0.0209±0.0185	295000	288000	279000	280000	0.0773±0.0296	45800	485000	50200	52300	0.012±0.0074	69600	70700	64800	80400	0.0107±0.0069	6830	8510	8730	7250	0.0016±0.0016	C07609	--
kz002912	[M+H]+	249.15	185.13	248.14	C15H20O3	Santamarin	Terpenoids	BioDeep	4290-13-5	-	-	-	-	-	-	-	-	-	-	533000	611000	479000	533000	0.1311±0.1415	31800	24700	29500	39000	0.0047±0.0076	26600	28700	29600	20900	0.0055±0.0071	-	-
kz002933	[M+H]+	427.20	265.14	426.19	C21H30O9	Ixerin D	Terpenoids	BioDeep	-	1950000	2010000	2060000	1980000	0.033±0.0138	61200	66100	61700	54000	0.0165±0.0158	34100	290000	33000	32900	0.0078±0.0046	89000	63400	83500	91000	0.0123±0.0173	76800	95400	94200	86800	0.0183±0.0151	-	-
kz002936	[M+H]+	483.13	153.00	482.12	C25H22O10	Silibinin	Flavonoids	BioDeep	-	-	-	-	-	-	-	-	-	-	-	798000	758000	777000	833000	0.1924±0.0619	54600	53200	56700	58300	0.0084±0.0026	3050	3420	3400	3010	0.0007±0.0003	C07610	--
kz002949	[M+H]+	301.07	286.05	300.06	C16H12O6	Hispidulin	Flavonoids	BioDeep	189109-41-9	139000	125000	138000	133000	0.0022±0.0023	7160	7660	7900	7490	0.002±0.0009	7460	6660	8140	6540	0.0018±0.0018	28700	26700	27300	30200	0.0043±0.0012	2290	4210	3370	3720	0.0007±0.0017	C10058	--
kz002960	[M+H]+	345.10	330.07	344.09	C18H16O7	Eupatilin	Flavonoids	BioDeep	-	4350	5340	6080	4440	0.0008±0.0016	11700	11900	12800	13200	0.0034±0.0015	-	-	-	-	-	19700	19100	19200	22200	0.003±0.0013	-	-	-	-	-	C10040	--
kz002962	[M+H]+	345.10	312.06	344.09	C18H16O7	Penduletin (5,4'-Dihydroxy-3,6,7-trimethoxyflavone)	Flavonoids	BioDeep	-	3440	3420	3880	3010	0.0006±0.0008	9680	9620	10100	9960	0.0027±0.0003	-	-	-	-	-	13200	14500	14100	11800	0.002±0.0023	-	-	-	-	-	-	-

kz002975	[M+H]+	461.12	285.08	460.12	C22H20O11	Acacetin-7-O-glucuronide	Flavonoids	BioDeep	5213-27-1	28800	26200	22400	29600	0.0044±0.0076	-	-	-	-	-	-	-	-	-	-	10000	14100	12100	12100	0.0018±0.0027	-	-	-	-	-	-	-
kz002976	[M+H]+	463.09	287.05	462.08	C21H18O12	Scutellarin (Scutellarein-7-O-glucuronide)	Flavonoids	BioDeep	537-98-4	66000	83900	65400	80700	0.0122±0.0156	23500	26100	23300	27600	0.0068±0.0057	3320000	2890000	3160000	3370000	0.7739±0.4326	956000	968000	848000	984000	0.1415±0.0753	885000	834000	831000	797000	0.174±0.0892	-	-
kz002977	[M+H]+	463.12	301.07	462.12	C22H22O11	Diosmetin-7-O-galactoside	Flavonoids	BioDeep	-	155000	141000	133000	137000	0.0234±0.0293	94100	89800	98100	84500	0.0248±0.0161	63900	55600	64900	70100	0.0155±0.0135	152000	170000	165000	184000	0.0253±0.0163	19300	21500	37300	24400	0.0053±0.0153	-	-
kz002978	[M+H]+	463.12	301.07	462.12	C22H22O11	Pratensein 7-O-glucopyranoside	Flavonoids	BioDeep	-	6540	7790	9180	14200	0.0016±0.0068	74000	72800	67000	68200	0.0191±0.0118	-	-	-	-	-	3760	4540	5190	4280	0.0007±0.001	-	-	-	-	-	-	-
kz002981	[M+H]+	477.10	301.07	476.10	C22H20O12	Diosmetin-7-O-glucuronide	Flavonoids	BioDeep	35110-20-4	50400	39900	38800	37800	0.0069±0.0153	-	-	-	-	-	-	-	-	-	-	-	-	-	-	-	-	-	-	-	-	-	-
kz002982	[M+H]+	479.12	317.07	478.11	C22H22O12	6-methoxykaempferol-3-O-glucoside	Flavonoids	BioDeep	-	1080000	1120000	1230000	1150000	0.1892±0.1351	402000	388000	395000	433000	0.1094±0.0431	1480000	1330000	1370000	1490000	0.3444±0.1477	1560000	1420000	1370000	1360000	0.2153±0.1597	94600	126000	118000	110000	0.0233±0.0251	-	-
kz002983	[M+H]+	495.1	333.0	494.11	C22H22O13	Patuletin-3-O-β-D-glucopyranoside	Flavonoids	BioDe	-	7570	7310	7540	9590	0.0132±0.0224	-	-	-	-	-	-	-	-	-	-	-	-	-	-	-	-	-	-	-	-	-	-

		1	7					ep		0	0	0	0																							
kz002990	[M+H]+	595.17	449.10	594.16	C27H30O15	Luteolin-7-O-rutinoside	Flavonoids	BioDeep	6812-98-2	47000	72100	71200	53900	0.01±0.0226	78900	126000	116000	87800	0.0277±0.0632	966000	10900000	1100000	1450000	0.28±0.5048	106000	129000	114000	101000	0.017±0.0223	299000	302000	311000	312000	0.0636±0.0191	-	-
kz002995	[M+H]+	551.10	303.05	550.10	C24H22O15	Quercetin-7-O-(6'-O-malonyl)-β-D-glucoside	Flavonoids	BioDeep	-	36500	43300	47100	56600	0.0076±0.0162	24200	31300	22200	27200	0.0071±0.0116	42200	44300	42000	62500	0.0116±0.0237	115000	98300	102000	124000	0.0165±0.0134	10100	8180	12200	12000	0.0022±0.0039	-	-
kz003038	[M-H]-	675.41	397.14	676.42	C38H60O10	3-O-(2-O-Acetyl-β-D-glucopyranosyl) oleanolic acid	Terpenoids	BioDeep	-	594000	631000	617000	540000	0.0984±0.0835	986000	956000	876000	975000	0.2566±0.1505	231000	260000	250000	259000	0.0608±0.0397	2510000	2310000	2450000	2570000	0.3707±0.1132	1970000	2130000	1930000	1980000	0.4165±0.2226	-	-
kz003083	[M+H]+	477.14	315.08	476.13	C23H24O11	5,2'-Dihydroxy-7,8-dimethoxyflavone glycosides	Flavonoids	BioDeep	-	-	-	-	-	-	2630	3360	2530	3400	0.0008±0.0013	-	-	-	-	-	39700	38600	38700	46800	0.0062±0.0041	-	-	-	-	-	-	-
kz003097	[M-H]-	477.06	169.01	478.07	C21H18O13	3,4-Di-O-galloyl-shikimic acid	Phenolic acids	BioDeep	537-11-5	1750000	1810000	1680000	1690000	0.0286±0.0156	107000	131000	142000	98200	0.0324±0.0565	142000	1240000	140000	124000	0.0322±0.0222	980000	953000	1080000	1040000	0.1528±0.0942	352000	370000	329000	342000	0.0724±0.0457	-	-
kz003098	[M-H]-	495.08	343.07	496.09	C21H20O14	3-O-Digalloyl quinic acid	Phenolic acids	BioDeep	-	79000	133000	115000	109000	0.0179±0.0385	16900	24400	17800	12900	0.0049±0.0137	4070000	3710000	3800000	3940000	0.9429±0.2408	144000	175000	160000	194000	0.0253±0.0278	36900	37200	35100	39700	0.0077±0.0058	-	-
kz	[M	5	3	51	C19	Di-O-	Phe	Bi	-	3	2	3	2	0.482	85	81	82	84	0.225	757	7	75	718	0.184±	303	295	3340	269	0.045	1620	17	15	16	0.0339	-	-

003099	-H]-	15.116	41.11	6.17	H32O16	Glucose-quinic acid	nolic acids	oDeep		120000	710000	030000	790000	2±0.622	6000	0000	2000	0000	1±0.0513	000	92000	9000	000	0.0929	000	000	00	000	4±0.0516	00	0000	5000	5000	±0.0193		
kz003100	[M-H]-	331.07	169.02	332.07	C13H16O10	5-O-Galloylhamamelose	Phenolic acids	BioDeep	22138-51-8	6410000	7120000	7050000	6980000	1.1373±0.2848	1590000	1620000	1630000	1540000	0.4316±0.1542	1220000	1110000	1190000	1160000	0.2844±0.0854	6080000	6240000	6370000	6670000	0.9557±0.2786	679000	706000	676000	698000	0.1435±0.0566	-	-
kz003103	[M-H]-	633.07	301.00	634.08	C27H22O18	Gemin D	Phenolic acids	BioDeep	-	5800000	6270000	6970000	6000000	0.1034±0.0994	69000	69300	36800	60400	0.016±0.0429	-	-	-	-	-	69500	54700	80600	93500	0.0112±0.0226	126000	160000	150000	121000	0.0289±0.0338	-	-
kz003104	[M-H]-	785.09	301.00	786.09	C34H26O22	Nobotanin D	Phenolic acids	BioDeep	-	253000	290000	384000	368000	0.0053±0.0124	-	-	-	-	-	-	-	-	-	-	-	-	-	-	-	-	-	-	-	-	-	-
kz003106	[M-H]-	935.08	633.07	936.09	C41H28O26	Cuspinin	Phenolic acids	BioDeep	-	797000	1000000	1180000	1010000	0.0164±0.0294	10300	15600	13200	11700	0.0034±0.0066	-	-	-	-	-	17900	10400	16400	25100	0.0026±0.0083	-	-	-	-	-	C10212	--
kz003108	[M+H]+	465.10	303.06	464.10	C21H20O12	mlyricetin 3-O-B-D-glucopyranoside	Flavonoids	BioDeep	-	900000	905000	893000	759000	0.1429±0.1678	204000	221000	205000	263000	0.0604±0.0712	236000	232000	254000	266000	0.06±0.0385	609000	583000	521000	682000	0.0901±0.0755	425000	334000	308000	288000	0.0705±0.1331	-	-
kz003109	[M-H]-	477.10	314.05	478.11	C22H22O12	Mearnsitrin	Flavonoids	BioDee	-	22400	28600	27200	22700	0.0416±0.0513	111000	124000	104000	123000	0.0313±0.0286	558000	52000	503000	537000	0.1287±0.0411	500000	460000	453000	449000	0.0702±0.0428	36800	36300	36800	27000	0.0071±0.0092	-	-

								p		0	0	0	0								0															
kz003116	[M+H]+	171.03	81.03	170.02	C7H6O5	2,4,6-trihydroxybenzoic acid	Organic acids	BioDeep	-	105000	71100	128000	90800	0.0164±0.0535	1120000	1110000	1150000	1310000	0.0317±0.0218	35300	199000	24000	27700	0.0065±0.0149	96900	86600	94200	121000	0.015±0.0179	41700	38400	30800	29800	0.0073±0.0127	-	-
kz003147	[M+H]+	359.15	327.12	358.14	C20H22O6	Clemaphenol A	Lignans	BioDeep	36260-60-8	170000	226000	175000	188000	0.0313±0.0385	5620	7860	9510	4470	0.0019±0.0061	12700	13500	13300	12300	0.0031±0.0017	79500	82100	64000	79000	0.0115±0.0117	-	-	-	-	-	C17808	--
kz003151	[M+H]+	389.16	181.04	388.15	C21H24O7	Ailantinol E	Others	BioDeep	-	233000	271000	298000	280000	0.0045±0.0048	10700	8320	10600	10500	0.0027±0.0028	400000	349000	347000	366000	0.0888±0.0467	130000	127000	125000	131000	0.0193±0.029	41100	42400	39900	35200	0.0082±0.006	-	-
kz003158	[M+H]+	213.07	167.06	212.06	C12H8N2O2	Ailanindole	Alkaloids	BioDeep	159903-51-2	45000	51200	56600	50600	0.0084±0.0084	12900	20800	24500	24100	0.0056±0.0142	40000	310000	43400	27900	0.0086±0.0177	161000	171000	311000	230000	0.0329±0.1075	17800	15700	6400	2030	0.0061±0.0459	-	-
kz003166	[M+H]+	193.05	178.02	192.04	C10H8O4	6-Hydroxy-7-methoxycoumarin	Coumarins	BioDeep	776-86-3	11100	24800	16100	15600	0.0028±0.0104	83100	86300	76200	83500	0.0223±0.0143	1150000	1090000	1100000	1070000	0.268±0.0665	21000	20200	20500	18900	0.003±0.0021	973000	95900	1050000	98400	0.2061±0.0548	-	-
kz003262	[M-H]-	187.10	123.10	188.05	C11H8O3	Ayapin	Coumarins	BioDeep	494-56-4	2260000	2420000	2260000	2270000	0.038±0.0117	1690000	1680000	1720000	1640000	0.0455±0.012	280000	261000	273000	286000	0.0668±0.0191	235000	232000	202000	246000	0.0345±0.0219	598000	576000	819000	582000	0.1334±0.2078	C18078	--
kz003362	[M-H]-	665.2	503.1	666.22	C31H38O16	Tubuloside C	Phenolic acid	BioDe	-	6930	6840	6470	5100	0.0105±0.0192	38400	37500	27700	51000	0.0105±0.0253	11300000	1100	10700000	11200000	2.6861±0.432	206000	227000	232000	212000	0.0331±0.026	274000	2770000	2840000	3150000	0.0598±0.0486	-	-

		1	8				s	ep		0	0	0	0								0000															
kz003370	[M+H]+	375.13	177.05	374.12	C16H22O10	Swertiamarin	Terpenoids	BioDeep	17388-39-5	454000	500000	564000	576000	0.0086±0.0108	16400	21200	15200	11000	0.0043±0.0121	47000	50500	63400	55300	0.0132±0.0182	44600	53800	58000	51400	0.0078±0.0097	-	-	-	-	-	-	-
kz003497	[M-H]-	331.07	169.01	332.07	C13H16O10	3-O-Galloyl-β-D-glucose	Phenolic acids	BioDeep	-	7020000	7250000	7100000	6640000	1.1571±0.6803	1620000	1630000	1600000	1630000	0.4384±0.0939	1150000	1110000	1230000	1150000	0.282±0.1307	6240000	6260000	6320000	6860000	0.9674±0.2183	696000	655000	692000	680000	0.1416±0.0525	-	-
kz003498	[M-H]-	331.07	169.01	332.07	C13H16O10	2-O-Galloyl-β-D-glucose	Phenolic acids	BioDeep	-	1490000	1710000	1550000	1440000	0.2554±0.1768	2550000	2560000	2520000	2540000	0.688±0.1515	4530000	4520000	4600000	4600000	1.1093±0.1982	5510000	5400000	5030000	5660000	0.8137±0.255	3760000	4010000	3940000	3820000	0.807±0.1852	-	-
kz003504	[M-H]-	951.08	933.07	952.08	C41H28O27	Geraniinic acid C	Phenolic acids	BioDeep	-	19400	33400	22200	14600	0.0037±0.015	-	-	-	-	-	-	-	-	-	-	23500	25100	18300	26200	0.0035±0.0047	58100	6160	6720	4600	0.0121±0.016	-	-
kz003517	[M-H]-	299.08	137.02	300.08	C13H16O8	Glucosyloxybenzoic acid	Phenolic acids	BioDeep	39015-76-4	695000	830000	644000	506000	0.011±0.0261	65100	60100	61600	54000	0.0163±0.0136	285000	265000	294000	277000	0.0681±0.0282	227000	198000	214000	218000	0.0323±0.0169	57600	6700	6070	5700	0.0126±0.0086	-	-
kz003518	[M-H]-	315.11	153.06	316.12	C14H20O8	5-(2-Hydroxyethyl)-2-O-glucosylohenol	Phenolic acids	BioDeep	-	715000	707000	620000	657000	0.1116±0.1156	890000	858000	806000	756000	0.224±0.1864	588000	559000	602000	607000	0.1432±0.0469	497000	406000	411000	434000	0.0659±0.0608	30100	3880	5530	4260	0.0086±0.0198	-	-

kz003519	[M-H]-	315.11	153.10	316.12	C14H20O8	Cimidahurinine	Phenolic acids	BioDeep	-	960000	1040000	944000	832000	0.1559±0.1718	11600000	12000000	12300000	11600000	0.3213±0.1086	809000	854000	835000	814000	0.2014±0.08	644000	573000	554000	785000	0.096±0.1273	44000	46300	54200	43300	0.0097±0.0077	-	-
kz003540	[M-H]-	449.11	287.06	450.12	C21H22O11	Dihydrokaempferol-7-O-glucoside	Flavonoids	BioDeep	-	3120000	3740000	3240000	3070000	0.0543±0.0452	13100000	13800000	15600000	14600000	0.386±0.2561	128000	110000	116000	110000	0.0282±0.0175	1510000	1460000	1480000	1580000	0.2272±0.0216	49900	45500	41400	42200	0.0093±0.0094	-	-
kz003549	[M-H]-	471.35	471.35	472.36	C30H48O4	Hederagenin	Others	BioDeep	465-99-6	7120000	7700000	6880000	7730000	1.2148±0.698	1030000000	1080000000	1140000000	1140000000	2.9685±1.1961	4560000	4480000	4580000	4750000	1.1165±0.2544	11700000	12300000	11700000	12000000	1.7985±0.6557	11900000	11600000	11700000	11900000	2.449±0.8945	-	-
kz003572	[M+H]+	595.14	147.04	594.14	C30H26O13	Biondnoid I	Flavonoids	BioDeep	142750-32-1	6230000	6880000	6530000	6250000	0.1069±0.0363	6420000	6710000	7360000	7880000	0.1917±0.1548	1200000	1210000	1110000	1190000	0.2863±0.1101	4280000	3780000	3910000	4060000	0.6041±0.2935	1870000	1870000	2020000	2120000	0.4097±0.2886	-	-
kz003634	[M+H]+	371.10	209.05	370.13	C17H22O9	Sinapaldehyde Glucoside	Phenolic acids	BioDeep	-	-	-	-	-	-	2600000	2410000	2540000	2430000	0.0675±0.024	-	-	-	-	-	-	-	-	-	-	22400	14200	21600	20700	0.0041±0.0079	-	-
kz003636	[M-H]-	477.14	161.05	478.15	C23H26O11	Plantainoside A	Phenolic acids	BioDeep	-	1200000	1580000	1300000	1500000	0.023±0.0264	51900	54600	46400	63000	0.0146±0.0184	28000	28700	25700	34500	0.0071±0.0089	102000	95700	84200	93800	0.0142±0.0108	-	-	-	-	-	-	-
kz0036	[M+H]+	449.	287.	448.10	C21H20O11	Quercetin-3-O-α-L-rhamnopy	Flavonoids	BioD	-	255	294	298	276	0.4633±0.2684	100000	912000	946000	773000	0.2457±0.2774	1880000	179	158000	1860000	0.4319±0.2999	3720000	3990000	3560000	4000000	0.5754±0.2733	2510000	264000	258000	243000	0.5279±0.1375	-	-

90		11	05			ranoside		eep		0000	0000	0000	0000		0						0000	0									0	0	0			
kz003693	[M+H]+	611.16	303.05	610.15	C27H30O16	Quercetin-3-O-(2-O-α-L-rhamnopyranosyl)-β-D-galactopyranoside	Flavonoids	BioDeep	-	7830	133000	9800	124000	0.0018±0.0044	120000	9790	13400	11000	0.0031±0.0038	225000	216000	269000	284000	0.0604±0.0803	8520	10800	9890	8820	0.0014±0.0019	160000	125000	164000	164000	0.0319±0.0408	-	-
kz003714	[M-H]-	331.07	169.01	332.07	C13H16O10	6-O-Galloyl-β-D-glucose	Phenolic acids	BioDeep	13186-19-1	6440000	7400000	7070000	6970000	1.1501±0.3018	16400000	15800000	16700000	15400000	0.4349±0.1659	1140000	1160000	1060000	1150000	0.2741±0.1124	6230000	6470000	6560000	6780000	0.9814±0.3067	666000	720000	622000	660000	0.1388±0.1014	-	-
kz003716	[M-H]-	467.08	169.01	468.09	C20H20O13	Maplexin D (2,4-Di-O-Galloyl-1,5-Anhydro-D-Glucitol)	Phenolic acids	BioDeep	-	460000	554000	471000	472000	0.0807±0.0588	70900	51900	55500	77400	0.0173±0.0315	-	-	-	-	-	56900	67000	67900	127000	0.0119±0.0438	-	-	-	-	-	-	-
kz003717	[M-H]-	467.08	169.01	468.09	C20H20O13	Maplexin C (2,3-Di-O-Galloyl-1,5-Anhydro-D-Glucitol)	Phenolic acids	BioDeep	-	489000	686000	654000	705000	0.1043±0.1669	116000	124000	122000	89900	0.0306±0.0455	-	-	-	-	-	117000	104000	158000	100000	0.0181±0.0438	-	-	-	-	-	-	-
kz003718	[M-H]-	483.08	169.01	484.09	C20H20O14	2,3-Di-O-Galloyl-D-Glucose	Phenolic acids	BioDeep	-	1090000	1760000	1560000	1360000	0.0237±0.0489	56700	52500	43300	51700	0.0138±0.0162	27100	219000	17100	16000	0.005±0.0119	802000	836000	954000	916000	0.1323±0.1107	142000	167000	140000	121000	0.0296±0.0374	-	-
kz0037	[M-H]-	497.	169.	498.10	C21H22O14	Methyl 4,6-di-O-galloyl-D-	Phenolic	BioD	-	384	395	425	405	0.0665±0.04	64400	62200	58300	83400	0.0181±0.0287	17600	263	15400	28800	0.0054±0.016	23600	19700	17400	23900	0.0032±0.004	5310	14200	7900	11600	0.002±0.0083	-	-

19		09	01			glucoside	acids	eep		000	000	000	000								00															
kz003720	[M+H]+	619.13	153.02	618.12	C28H26O16	Maplexin H	Phenolic acids	BioDeep	-	286000	340000	396000	340000	0.0562±0.0837	183000	170000	198000	148000	0.0473±0.0575	30800	18500	25500	23500	0.006±0.0116	519000	505000	590000	567000	0.0822±0.0622	81600	5670	6240	5950	0.0135±0.0249	-	-
kz003721	[M-H]-	633.11	169.01	634.12	C28H26O17	Maplexin G	Phenolic acids	BioDeep	-	55700	84100	71300	70300	0.0116±0.0185	20800	25300	28800	29500	0.0071±0.0102	19900	19100	29700	24800	0.0057±0.0122	164000	160000	191000	163000	0.0256±0.0264	49300	5950	4720	5020	0.0107±0.0119	-	-
kz003728	[M+H]+	389.14	209.08	388.14	C17H24O10	Geniposide	Terpenoids	BioDeep	-	43600	45900	55400	49000	0.008±0.0104	17100	23500	25400	23500	0.0061±0.0097	32500	26700	29100	22800	0.0067±0.0095	1130000	1470000	1600000	1450000	0.2132±0.3229	102000	8400	104000	150000	0.0229±0.0628	C09781	--
kz003748	[M+H]+	553.23	207.07	552.22	C27H36O12	7'-O-Sinapoyljasminoside L	Terpenoids	BioDeep	-	354000	383000	284000	353000	0.0567±0.0836	38800	31400	34600	40600	0.0098±0.0102	-	-	-	-	-	494000	458000	318000	456000	0.065±0.1115	9970	9920	8350	7570	0.0019±0.0025	-	-
kz003755	[M+H]+	597.155	303.055	596.14	C26H28O16	Quercetin-3-sambubioside	Flavonoids	BioDeep	83048-35-5	-	-	-	-	-	7390	8800	6640	5880	0.0019±0.0037	52400	58200	50800	70800	0.0141±0.0219	67300	72600	79500	92800	0.0117±0.0142	1160000	106000	123000	116000	0.2396±0.1343	-	-
kz003763	[M+H]+	443.39	425.38	442.38	C30H50O2	Betulin	Terpenoids	BioDeep	473-98-3	13500	13500	15700	16300	0.0024±0.0031	12800	11400	9220	11700	0.0031±0.0042	10100	6060	4970	6850	0.0017±0.0051	63900	73500	63600	69200	0.0102±0.0074	98700	9320	110000	101000	0.0209±0.012	C08618	--
kz0037	[M+H]+	471.	425.	470.34	C30H46O4	Uncargenin D	Terpenoids	BioD	-	578	656	472	597	0.0095±0.0145	106000	119000	112000	116000	0.0306±0.0175	-	-	-	-	-	204000	193000	215000	184000	0.03±0.0272	32700	3430	3740	3840	0.0074±0.0056	-	-

66		35	34					eep		00	00	00	00																						
kz003767	[M+H]+	471.335	453.334	470.34	C30H46O4	Obtusilin	Terpenoids	BioDeep	105870-59-5	49200	53200	51000	49800	0.0084±0.002	99200	86100	113000	92400	0.0264±0.0288	6010	4210	6730	4210	0.0013±0.0031	90900	93300	94700	93200	0.014±0.0052	193000	182000	201000	187000	0.0396±0.0136	-	-
kz003793	[M-H]-	481.114	319.08	482.11	C21H22O13	Dihydromyricetin-O-glucoside	Flavonoids	BioDeep	-	39600	32400	27200	31200	0.0054±0.0128	19700	20500	18000	23900	0.0056±0.0065	6910	10200	13800	9560	0.0025±0.0071	36900	39800	36900	41500	0.0058±0.0025	18900	23800	32700	24700	0.0052±0.0106	-	-
kz003794	[M-H]-	343.111	181.05	344.11	C15H20O9	Syringic Aldehyde-glucoside	Phenolic acids	BioDeep	87687-52-3	51200	43500	56100	41100	0.0079±0.0165	48300	52000	72900	46100	0.0148±0.0324	296000	220000	210000	260000	0.0598±0.0868	-	-	-	-	-	-	-	-	-	-	-	-
kz003796	[M-H]-	329.11	167.03	330.10	C14H18O9	1'-O-vanilloyl-β-D-glucoside	Phenolic acids	BioDeep	-	13700	28700	26300	27300	0.0039±0.013	15100	17600	9110	6780	0.0033±0.0142	11900	12800	17500	10900	0.0032±0.0074	525000	487000	513000	545000	0.078±0.0218	28600	31400	38300	28200	0.0066±0.0078	-	-
kz003797	[M-H]-	367.14	205.08	368.15	C18H24O8	3-Prenyl-4-O-β-D-glucopyranosyloxy-4-hydroxyl-benzoic acid	Phenolic acids	BioDeep	-	16100	25100	21200	16600	0.0032±0.0074	-	-	-	-	-	194000	230000	239000	236000	0.0547±0.0564	-	-	-	-	-	30300	33900	22600	39900	0.0066±0.0164	-	-
kz003809	[M-H]-	451.12	289.07	452.13	C21H24O11	Sieboldin	Flavonoids	BioDeep	-	13000	10000	10700	16200	0.0021±0.0061	9490	16500	13300	14700	0.0037±0.0083	163000	138000	139000	181000	0.0377±0.0463	126000	115000	127000	138000	0.0191±0.0102	-	-	-	-	-	-	-
kz	[M	5	2	53	C24	Kaempfer	Flav	Bi	-	3	4	3	4	0.006	19	16	21	21	0.005	532	4	44	473	0.0112	510	473	5290	528	0.007	1260	13	13	94	0.0025	-	-

003811	+H]+	35.111	87.06	4.10	H22O14	ol-malonyl-3-O-glucoside	onoids	oDeep		0100	3500	1000	5800	2±0.0149	200	400	000	600	3±0.0055	00	0700	000	00	±0.0112	00	00	0	00	7±0.0037	0	800	000	40	±0.0037		
kz003812	[M+H]+	565.12	317.08	564.11	C25H24O15	Isorhamnetin O-malonylglucoside	Flavonoids	BioDeep	-	3890	4860	3740	2730	0.0006±0.0017	3010	2430	2780	3230	0.0008±0.0008	13100	8900	12400	8520	0.0026±0.0055	7660	7230	6110	7360	0.0011±0.0009	-	-	-	-	-	-	-
kz003815	[M+H]+	595.14	287.05	594.14	C30H26O13	Kaempferol 3-O-(6''-trans-p-Coumaroyl)-β-D-glucopyranoside	Flavonoids	BioDeep	-	32000	36600	23700	25400	0.0049±0.0119	17000	16500	18100	19000	0.0048±0.0023	93900	74100	9390	87000	0.0212±0.021	211000	188000	230000	257000	0.0333±0.0374	35500	34000	49100	28300	0.0076±0.0162	-	-
kz003821	[M+H]+	601.12	287.06	600.11	C28H24O15	Kampferol 3-O-(6″-galloyl)-β-D-galactopyranoside	Flavonoids	BioDeep	169565-72-4	144000	181000	193000	139000	0.0271±0.0501	65700	59200	55900	52100	0.0158±0.017	12600	9790	10300	6520	0.0024±0.0059	264000	236000	240000	322000	0.0399±0.0471	-	-	-	-	-	-	-
kz003823	[M+H]+	601.12	287.06	600.11	C28H24O15	Kampferol 3-O-(2″-galloyl)-β-D-galactopyranoside	Flavonoids	BioDeep	-	173000	193000	185000	186000	0.0304±0.03	76100	70200	64500	56000	0.0181±0.0251	-	-	-	-	-	241000	243000	242000	284000	0.038±0.021	-	-	-	-	-	-	-
kz003829	[M+H]+	617.11	303.05	616.11	C28H24O16	Quercetin 3-O-(6′′-galloyl)-β-D-galactopyranoside	Flavonoids	BioDeep	-	9470000	10500000	10100000	11800000	0.1728±0.1741	3610000	3890000	3580000	3380000	0.0979±0.0723	8630	13900	15900	14800	0.0032±0.0081	2100000	1930000	2000000	2100000	0.3063±0.0866	28200	22300	26400	16600	0.0049±0.0103	-	-
kz003833	[M+H]+	743.20	303.05	742.20	C32H38O20	Quercetin-O-pentosyl-O-rhamnosi	Flavonoids	BioDee	-	-	-	-	-	-	6660	6950	7840	7560	0.002±0.0013	14900	7960	20100	10500	0.0032±0.013	-	-	-	-	-	84200	8780	8670	11500	0.0195±0.0337	-	-

						de-O-glucoside		p																												
kz003848	[M-H]-	451.10	341.06	452.11	C24H20O9	Cinchonain Ic	Flavonoids	BioDeep	85081-22-7	330000	386000	324000	338000	0.0568±0.0397	1070000	1110000	1100000	1190000	0.0302±0.0124	7620	9820	9560	9370	0.0022±0.0027	66900	59400	67800	63900	0.0097±0.0065	2710	2960	3290	4070	0.0007±0.0013	-	-
kz003849	[M-H]-	521.20	359.14	522.21	C26H34O11	dihydrodehydrodiconiferyl alcohol 4-O-β-D-glucopyranosides	Lignans	BioDeep	-	504000	522000	507000	491000	0.0836±0.0369	2210000	2370000	2120000	2260000	0.0606±0.0363	585000	541000	513000	522000	0.1313±0.0663	1820000	1670000	1770000	1920000	0.2704±0.0968	545000	528000	590000	520000	0.1134±0.0413	-	-
kz003850	[M-H]-	523.21	361.14	524.23	C26H36O11	(-)-secoisolariciresinol 4-O-β-D-glucopyranoside	Lignans	BioDeep	-	98300	1000000	88200	85100	0.0154±0.0172	412000	313000	467000	592000	0.012±0.0297	2710000	2640000	2530000	2640000	0.6393±0.1404	325000	339000	304000	356000	0.0499±0.0236	98200	112000	99400	96100	0.0211±0.0144	-	-
kz003854	[M-H]-	385.19	223.13	386.19	C19H30O8	Roseoside	Others	BioDeep	54835-70-0	468000	491000	513000	467000	0.0801±0.0467	369000	349000	351000	376000	0.0977±0.0307	90400	75100	88300	97700	0.0213±0.0209	132000	145000	159000	174000	0.023±0.024	68100	75200	82500	74100	0.0156±0.009	-	-
kz003858	[M+H]+	127.04	69.03	126.03	C6H6O3	Pyrogallol	Others	BioDeep	87-66-1	16400000	15600000	13900000	14100000	0.2481±0.3207	18000000	18900000	17800000	19300000	0.5006±0.2175	1680000	17300000	16900000	1730000	0.4152±0.1095	1460000	1250000	1300000	1160000	0.1951±0.2173	1870000	1970000	2040000	2160000	0.418±0.2877	-	-
kz003859	[M+H]+	619.40	437.34	618.39	C39H54O6	p-Coumaroyl leuscaphic acid	Terpenoids	BioDeep	-	906000	1290000	915000	1030000	0.017±0.0294	59600	75800	78600	64700	0.0189±0.0251	313000	1640000	285000	242000	0.0609±0.1519	103000	116000	97600	105000	0.0159±0.0128	89200	96500	73800	95600	0.0185±0.0254	-	-
kz	[M	6	4	63	C39	Caffeoyl	Terp	Bi	-	6	5	5	7	0.108	43	50	36	43	0.117	936	8	11	986	0.0237	146	124	1710	153	0.223	5440	50	47	46	0.1029	-	-

003860	+H]+	35.39	35.32	4.39	H54O7	hawthorn acid	enoids	oDeep		92000	83000	88000	48000	1±0.1955	0000	1000	2000	7000	2±0.17	00	7300	1000	00	±0.025	0000	0000	000	0000	9±0.3029	00	0000	3000	1000	±0.0876		
kz003861	[M+H]+	665.40	409.30	664.40	C40H56O8	3-O-Trans-feruloyl euscaphic acid	Terpenoids	BioDeep	-	813000	951000	872000	846000	0.0144±0.0062	27700	38000	26000	41400	0.009±0.0207	13700	10600	12800	13900	0.0031±0.0033	42000	47800	43500	40000	0.0065±0.0065	20500	17400	14200	17100	0.0036±0.006	-	-
kz003862	[M+H]+	409.35	187.15	408.34	C29H44O	3β-Hydroxy-28-norurs-17,19,21-trien	Terpenoids	BioDeep	-	101000	117000	103000	105000	0.0176±0.0081	226000	272000	266000	285000	0.0709±0.0676	-	-	-	-	-	-	-	-	-	-	-	-	-	-	-	-	-
kz003863	[M+H]+	455.35	409.35	454.34	C30H46O3	Sanguisorbigenin	Terpenoids	BioDeep	-	776000	871000	737000	883000	0.1348±0.1203	1130000	1130000	1090000	1280000	0.3131±0.2088	223000	236000	237000	238000	0.0568±0.024	2300000	2280000	2300000	2380000	0.349±0.0454	1390000	1380000	1320000	1260000	0.2781±0.1375	-	-
kz003872	[M+H]+	501.32	233.15	500.31	C30H44O6	Lup-12-en-15α,19β-diol-3,11-dioxo-28-oic acid	Terpenoids	BioDeep	-	306000	336000	294000	309000	0.0051±0.0027	1010000	95800	96100	97400	0.0264±0.0074	-	-	-	-	-	9600	7350	7860	9110	0.0013±0.0014	108000	119000	119000	113000	0.0238±0.0077	-	-
kz003946	[M-H]-	343.10	181.05	344.11	C15H20O9	Dihydrocaffeoylglucose	Phenolic acids	BioDeep	-	187000	430000	243000	8410	0.0039±0.0281	9120	20700	3410	11800	0.0031±0.02	24800	617000	80700	37400	0.0125±0.0621	58800	52200	56200	58200	0.0085±0.0037	66000	69300	71100	71200	0.0144±0.0052	-	-
kz004013	[M+H]+	225.15	95.08	224.14	C13H20O3	Solatuberenol A	Others	BioDeep	-	836000	819000	783000	730000	0.131±0.1318	3720000	4730000	5350000	6050000	0.1341±0.2545	89000	83800	87700	88800	0.0212±0.0037	193000	194000	218000	202000	0.0304±0.0216	129000	116000	103000	81000	0.0223±0.043	-	-
kz	[M	3	1	32	C15	Cis-p-	Oth	Bi	-	5	5	4	4	0.084	50	52	44	47	0.013	770	9	88	977	0.0215	283	298	2680	298	0.043	7410	94	95	10	0.0192	-	-

004015	-H]-	25.09	63.04	6.10	H18O8	coumaric acid 4-O-glucoside	ers	oDeep		47000	19000	80000	99000	6±0.0885	600	900	500	100	2±0.0121	00	0900	700	00	±0.0225	000	000	00	000	2±0.0183	0	200	800	5000	±0.0275		
kz004021	[M+H]+	132.10	57.10	131.09	C6H13NO2	6-Deoxyfagomine	Alkaloids	BioDeep	197449-09-5	11700	8740	11200	10200	0.0017±0.0035	25800	29000	24200	25300	0.0071±0.0067	10500	10800	9570	13200	0.0027±0.0037	30700	29100	31800	31600	0.0046±0.0019	23600	20000	40400	23000	0.0055±0.0176	-	-
kz004035	[M+H]+	188.07	118.07	187.06	C11H9NO2	3-Indoleacrylic acid	Alkaloids	BioDeep	1204-06-4	3690	4500	2850	2850	0.0006±0.0015	4520	5930	2230	5290	0.0012±0.0045	20100	17000	18300	21300	0.0047±0.0041	85400	83900	79500	85500	0.0126±0.0032	3570	4090	18100	3250	0.0015±0.0143	-	-
kz004133	[M-H]-	175.02	87.01	176.03	C6H8O6	L-Ascorbic acid	Vitamins	BioDeep	50-81-7	382000	406000	385000	438000	0.0665±0.047	564000	632000	636000	671000	0.1693±0.1141	132000	127000	122000	116000	0.0302±0.0161	643000	828000	676000	759000	0.1095±0.1279	255000	317000	298000	304000	0.061±0.0542	C00072	ko00053,ko00480,ko01100,ko01110
kz004169	[M+H]+	479.12	317.06	478.11	C22H22O12	Isorhamnetin 3-O-β-D-Glucoside	Flavonoids	BioDeep	5041-82-7	1150000	1280000	1220000	994000	0.1917±0.239	410000	508000	414000	398000	0.1172±0.1568	1410000	1260000	1450000	1210000	0.3239±0.2766	1330000	1260000	1230000	1520000	0.2009±0.1345	128000	112000	82500	109000	0.0225±0.0435	-	-
kz004170	[M+H]+	479.12	317.07	478.11	C22H22O12	Rhamnetin 3-O-β-D-Glucoside	Flavonoids	BioDeep	27875-34-9	1130000	1230000	1190000	1140000	0.1936±0.0563	419000	400000	411000	409000	0.1109±0.0197	1440000	1480000	1460000	1350000	0.3484±0.1724	1500000	1290000	1260000	1500000	0.2089±0.1524	99400	107000	114000	105000	0.0221±0.008	-	-
kz004171	[M+H]+	595.17	287.05	594.16	C27H30O15	Kaempferol 3-O-β-D-neohesperidoside	Flavonoids	BioDee	32602-81-6	17000	16700	20200	12700	0.0275±0.0663	352000	236000	310000	308000	0.0815±0.1242	3480000	41200	3490000	3870000	0.9098±0.8475	402000	339000	492000	344000	0.0596±0.1186	848000	750000	785000	785000	0.1648±0.1085	-	-

								p		0	0	0	0								00															
kz0041 72	[M+H]+	597.15	303.05	596.14	C26H28O16	Quercetin 3-O-β-D-xylopyranosyl(1→2)-β-D-galactopyranoside	Flavonoids	BioDeep	-	-	-	-	-	-	13400	6640	10700	8090	0.0026±0.008	53900	55400	48300	49700	0.0126±0.0085	-	-	-	-	-	965000	110000	128000	119000	0.2354±0.2478	-	-
kz0041 73	[M+H]+	611.16	303.05	610.15	C27H30O16	Quercetin 3-O-β-D-neohesperidoside	Flavonoids	BioDeep	117611-67-3	552000	732000	510000	641000	0.1003±0.1671	1240000	1160000	1140000	1180000	0.3193±0.1309	4140000	3790000	4520000	4310000	1.0185±0.7355	2420000	2290000	2270000	2190000	0.3459±0.2048	1610000	1290000	1250000	1400000	0.289±0.3926	-	-
kz0041 74	[M-H]-	401.14	269.10	402.15	C18H26O10	Benzyl-O-β-D-glucopyranose-β-D-xyranoside	Others	BioDeep	-	1130000	1120000	942000	1270000	0.0184±0.0288	814000	722000	865000	738000	0.0212±0.0167	175000	1340000	146000	155000	0.037±0.0364	236000	248000	193000	260000	0.0353±0.038	3020	21600	11600	8090	0.0023±0.0161	-	-
kz0042 31	[M+H]+	505.35	469.33	504.35	C30H48O6	Isothankunic acid	Terpenoids	BioDeep	-	2090000	2370000	2420000	2570000	0.039±0.0328	7390000	7650000	7450000	8590000	0.2102±0.1383	40000	3700000	45900	36700	0.0097±0.0107	71200	62500	63000	63100	0.0098±0.0068	8890	6230	7860	7820	0.0016±0.0024	-	-
kz0042 33	[M+H]+	505.35	459.35	504.35	C30H48O6	Terminolic acid	Terpenoids	BioDeep	564-13-6	568000	579000	562000	599000	0.0095±0.0046	149000	146000	154000	166000	0.0416±0.0186	14300	178000	15900	18500	0.004±0.0049	13900	13100	11300	13400	0.0019±0.0015	92600	9300	9050	8940	0.019±0.006	-	-
kz0042 60	[M-H]-	471.35	471.35	472.36	C30H48O4	Corosolic acid	Terpenoids	BioDeep	4547-24-4	-	-	-	-	-	-	-	-	-	-	1890000	1820000	1860000	1950000	0.457±0.1	392000	327000	355000	372000	0.0545±0.0368	-	-	-	-	-	-	-
kz	[M	2	1	24	C15	Osthole	Cou	Bi	48	6	8	6	7	0.001	19	21	22	20	0.005	-	-	-	-	-	622	630	5640	526	0.000	1180	12	81	13	0.0024	C0	--

004263	+H]+	45.12	59.20	4.11	H16O3		marins	oDeep	4-12-8	070	190	940	590	2±0.0013	400	400	500	000	6±0.0039						0	0		0	9±0.0009	0	900	00	700	±0.0056	9280	
kz004425	[M+H]+	741.23	433.12	740.22	C33H40O19	Luteolin-O-rutinoside-O-rhamnoside	Flavonoids	BioDeep	-	-	-	-	-	-	-	-	-	-	-	202000	180000	187000	194000	0.0464±0.0159	-	-	-	-	-	7060	11600	5650	7240	0.0016±0.0054	-	-
kz004567	[M-H]-	433.04	301.00	434.05	C19H14O12	Ellagic Acid 4-O-Xylopyranoside	Phenolic acids	BioDeep	139163-18-1	1970000	2740000	2720000	2650000	0.4149±0.6128	4780000	4440000	4490000	4240000	1.2144±0.6833	5290000	4520000	4560000	4400000	1.1401±0.8543	3290000	3990000	2650000	4340000	0.5363±1.0323	2380000	2650000	2510000	2640000	0.5293±0.3078	-	-
kz004595	[M-H]-	951.07	933.10	952.08	C41H28O27	Geraniin	Phenolic acids	BioDeep	70855-41-3	29800	27400	45100	59600	0.0067±0.0306	-	-	-	-	-	-	-	-	-	-	22600	15500	21700	23400	0.0031±0.005	52800	53500	55400	39400	0.0104±0.0136	C10230	--
kz004596	[M-H]-	985.12	953.10	986.12	C42H34O28	Methyl neochebulagate	Others	BioDeep	-	35100	52300	58900	49500	0.0081±0.0187	-	-	-	-	-	-	-	-	-	-	-	-	-	-	-	-	-	-	-	-	-	-
kz004598	[M-H]-	483.08	169.01	484.09	C20H20O14	1,4-di-O-galloyl-β-D-glcose	Phenolic acids	BioDeep	-	-	-	-	-	-	52500	58100	47200	58800	0.0147±0.0156	539000	509000	508000	555000	0.1283±0.0431	42400	34200	56200	50800	0.0069±0.0145	40200	34700	29400	32900	0.0071±0.0106	-	-
kz004626	[M-H]-	471.35	471.35	472.36	C30H48O4	pomolic acid	Terpenoids	BioDeep	13849-91-7	7390000	7680000	6600000	7780000	1.2162±1.1152	10700000	11200000	11500000	11300000	3.0233±0.8739	4700000	461000	4640000	4940000	1.148±0.318	11300000	11600000	11300000	12100000	1.7447±0.3096	12100000	11500000	11500000	11700000	2.4337±1.1096	-	-

kz004627	[M-H]-	471.335	471.335	472.36	C30H48O4	alphitolic acid	Terpenoids	BioDeep	19533-92-7	07060000	07830000	06810000	07560000	1.2075±0.6857	10500000	11200000	11000000	11200000	2.9696±1.0273	4640000	04320000	4470000	4870000	1.1119±0.4684	11300000	11800000	11800000	11900000	1.7644±0.5979	11700000	12000000	11700000	11600000	2.4432±0.6437	-	-
kz004630	[M-H]-	471.335	471.335	472.36	C30H48O4	2α-hydroxyursolic acid	Terpenoids	BioDeep	-	324000	353000	301000	350000	0.0548±0.043	568000	568000	573000	583000	0.155±0.0162	101000	30700	37500	38500	0.0125±0.0775	7950000	7540000	7490000	7860000	1.1623±0.2619	608000	593000	594000	564000	0.1226±0.0414	-	-
kz004632	[M-H]-	485.333	423.333	486.33	C30H46O5	isoceanothic acid	Terpenoids	BioDeep	-	2740000	2930000	2800000	2910000	0.4698±0.0995	6370000	6550000	6370000	6600000	1.7514±0.4582	144000	129000	130000	136000	0.0327±0.0118	1150000	1140000	1130000	1160000	0.1727±0.0326	2370000	2390000	2490000	2400000	0.5015±0.0691	-	-
kz004636	[M+H]+	635.339	453.339	634.39	C39H54O7	2α-hydroxypyracrenic acid	Terpenoids	BioDeep	-	322000	291000	332000	327000	0.0526±0.0564	175000	186000	181000	213000	0.051±0.0421	75500	68700	68400	75500	0.0175±0.0074	721000	721000	787000	709000	0.1109±0.0786	213000	251000	292000	236000	0.0514±0.0561	-	-
kz004665	[M-H]-	303.05	151.03	304.06	C15H12O7	5,7,3',4',5’-pentahydroxydihydroflavone	Flavonoids	BioDeep	-	50600	63400	56400	61700	0.0096±0.0078	16700	12800	11600	11500	0.0036±0.0068	-	-	-	-	-	18300	13100	13300	10700	0.0021±0.005	-	-	-	-	-	-	-
kz004667	[M+H]+	465.09	303.06	464.10	C21H20O12	Myricetin-O-rhamnoside	Flavonoids	BioDeep	-	261000	302000	286000	303000	0.0475±0.0224	299000	126000	127000	124000	0.0458±0.2358	170000	147000	184000	179000	0.0413±0.0382	979000	866000	991000	1040000	0.146±0.0901	6560000	6910000	6670000	5980000	1.3569±0.6713	-	-
kz0046	[M+H]+	225.	85.0	224.14	C13H20O3	Annuionone D	Others	BioD	-	103	104	101	997	0.0168±0.089	245000	215000	186000	218000	0.0585±0.0684	218000	221	200000	202000	0.0511±0.0274	736000	606000	812000	869000	0.1138±0.1545	622000	694000	626000	604000	0.1323±0.0794	-	-

78		15	0					eep		000	000	000	00								000															
kz004697	[M-H]-	465.13	303.06	466.11	C21H22O12	Taxifolin-3'-O-β-D-glucoside	Flavonoids	BioDeep	83-30-7	306000	364000	337000	294000	0.0537±0.0504	128000	129000	152000	124000	0.036±0.0328	143000	142000	164000	142000	0.0359±0.0287	323000	339000	336000	318000	0.0496±0.0286	177000	136000	180000	150000	0.0334±0.0419	-	-
kz004698	[M-H]-	491.19	315.12	492.20	C25H32O10	7S,8R-threo-3′,9,9′-trihydroxy-3-methoxy-4′,7-epoxy-neolignan-4-O-α-L-rhamnopyranoside	Lignans	BioDeep	-	77700000	103000000	104000000	106000000	1.609±2.2427	178000000	184000000	169000000	175000000	0.4778±0.241	-	-	-	-	-	-	-	-	-	-	-	-	-	-	-	-	-
kz004703	[M-H]-	523.22	475.20	524.23	C26H36O11	secoisolariciresinol 9-O-β-D-glucopyranoside	Lignans	BioDeep	63320-67-2	21200	23900	22900	19800	0.0036±0.0032	4780	7750	3330	6050	0.0015±0.0053	458000	474000	541000	485000	0.1191±0.0975	339000	310000	296000	287000	0.0465±0.04	-	-	-	-	-	-	-
kz004735	[M-H]-	361.16	181.09	362.17	C20H26O6	Ligraminol E	Others	BioDeep	-	22900	24700	26500	22900	0.004±0.0033	6820	6930	8120	7130	0.002±0.0014	2810	4080	5070	5320	0.0011±0.0028	19200	18500	19100	20100	0.0029±0.005	2770	2620	4470	4170	0.0007±0.0019	-	-
kz004738	[M-H]-	377.16	329.14	378.17	C20H26O7	4,7,9,9′-Tetrahydroxy-3,3′-dimethoxy-8-O-4′-neolignan	Others	BioDeep	-	284000	308000	309000	286000	0.049±0.0215	48100	63900	54700	54700	0.015±0.0193	57300	54100	53100	51200	0.0131±0.0056	469000	434000	427000	477000	0.0681±0.0223	13900	19700	13700	12700	0.0031±0.0065	-	-
kz0047	[M+H]+	120.	103.	119.07	C8H9N	N-Benzylmethylene	Alkaloids	BioD	-	720	952	925	779	0.0014±0.0018	20900	22000	23600	23600	0.0061±0.003	10100	955	8840	8570	0.0023±0.0016	75100	74300	71100	77200	0.0112±0.0019	42800	41000	78000	40500	0.0104±0.0347	-	-

56		08	05			isomethylamine		eep		0	0	0	0								0															
kz004762	[M+H]+	542.32	483.25	541.32	C28H48NO7P	2-Hydroxy-5,8,11,14,17-icosapentaenoyloxy]propyl-2-(trimethylammonio)ethyl phosphate	Alkaloids	BioDeep	-	1490000	1680000	1460000	1500000	0.2529±0.1397	2140000	2260000	2300000	2460000	0.6194±0.312	287000	2960000	296000	303000	0.0719±0.0226	1010000	954000	999000	1020000	0.1501±0.0374	1790000	1820000	1850000	1830000	0.3789±0.0783	-	-
kz004768	[M-H]-	493.12	169.01	494.13	C19H26O15	monogalloyl-diglucose	Phenolic acids	BioDeep	98767-37-4	2220000	2660000	2740000	2530000	0.0419±0.0345	-	-	-	-	-	-	-	-	-	-	17200	20100	23100	18500	0.003±0.0044	-	-	-	-	-	-	-
kz004771	[M-H]-	451.12	289.07	452.13	C21H24O11	Epicatechin glucoside	Flavonoids	BioDeep	-	1590000	1470000	1680000	1550000	0.2601±0.2676	6970000	6980000	6580000	6890000	0.1855±0.0728	817000	7860000	827000	862000	0.2001±0.0676	457000	381000	449000	498000	0.0672±0.0599	15600	12700	18300	19100	0.0034±0.0061	-	-
kz004774	[M-H]-	563.18	255.07	564.18	C27H32O13	(2R)-Pinocembrin-7-neohesperidoside	Flavonoids	BioDeep	-	700000	795000	711000	775000	0.0123±0.0054	1180000	1140000	1180000	1260000	0.0322±0.0098	24500	249000	25300	26400	0.0061±0.0022	3080	5760	5680	3720	0.0007±0.0022	-	-	-	-	-	-	-
kz005037	[M+H]+	531.15	177.05	512.13	C26H24O11	Caffeoylferuloylshikimic?acid	Phenolic acids	BioDeep	-	7930	10100	8170	10700	0.0015±0.0024	4320	3660	1970	2320	0.0008±0.0031	2080	24110	2670	2330	0.0006±0.0007	4270	5560	3700	4100	0.0007±0.0013	117000	120000	110000	111000	0.0238±0.0128	-	-
kz00	[M+H]	36	17	368.0	C16H16	Scopoletin ?Beta-D-	Coumar	Bio	1327	23	27	26	22	0.0041±0.0	8540	8250	8690	7950	0.0226±0.0	-	-	-	-	-	12300	13700	7820	13200	0.0018±0.0	31500	2130	2650	2790	0.0056±0.009	-	-

5043	+	9.16	7.06	7	O10	Glucuronide	ins	Deep	52-65-9	9000	4000	3000	8000	035	0	0	0	0	095										039		0	0	0	5		
kz005046	[M+H]+	451.12	289.08	450.12	C21H22O11	Dihydrokaempferol-3-O-β-D-glucoside	Flavonoids	BioDeep	-	1430000	1990000	2300000	1870000	0.0312±0.0674	65000	49900	58900	50500	0.0152±0.0193	150000	157000	144000	135000	0.0356±0.0252	263000	263000	218000	227000	0.0366±0.0393	223000	265000	241000	179000	0.0471±0.0689	-	-
kz005049	[M+H]+	407.13	245.08	406.13	C20H22O9	Salireposide	Phenolic acids	BioDeep	16290-07-6	2620000	2750000	2940000	2930000	0.4641±0.2978	1790000	1920000	1880000	2160000	0.524±0.3955	61200	48100	49800	56600	0.0131±0.0128	-	-	-	-	-	-	-	-	-	-	-	-
kz005054	[M+H]+	417.12	255.06	416.11	C21H20O9	Chrysin-7-glucoside	Flavonoids	BioDeep	1357621-91-0	553000	655000	569000	603000	0.0098±0.0051	82700	76600	82000	78900	0.0217±0.0066	48700	41600	46700	43900	0.011±0.0065	172000	165000	173000	172000	0.0257±0.0079	25400	24800	22700	21600	0.0049±0.004	-	-
kz005055	[M+H]+	433.11	271.06	432.11	C21H20O10	Galangin-7-glucoside	Flavonoids	BioDeep	1355573-86-2	339000	500000	364000	440000	0.0068±0.0121	109000	75900	80000	67300	0.0225±0.0497	389000	420000	348000	362000	0.0924±0.0823	101000	92600	76100	86000	0.0134±0.016	30200	31700	32700	27000	0.0063±0.0038	-	-
kz005078	[M+H]+	449.11	287.07	448.10	C21H20O11	Kaempferol-7-O-β-D-glucopyranoside	Flavonoids	BioDeep	-	6930000	7790000	7790000	7170000	1.2248±0.5945	2870000	3090000	3050000	3140000	0.8218±0.3268	6100000	5480000	6150000	4350000	1.3421±2.0391	11800000	11800000	11300000	12900000	1.8002±0.519	8950000	8740000	9100000	7660000	1.7891±1.1066	-	-
kz005079	[M+H]+	449.11	287.06	448.10	C21H20O11	Kaempferol-4'-O-β-D-glucopyranoside	Flavonoids	BioDeep	-	7070000	7650000	7360000	7450000	1.219±0.102	3080000	2270000	2720000	2520000	0.7159±0.8977	5640000	6020000	5870000	6310000	1.4493±0.7744	11400000	11600000	11500000	12400000	1.7671±0.327	8240000	8930000	8770000	8310000	1.7795±0.4786	-	-

										0	0	0	0								0															
kz005084	[M+H]+	611.16	287.066	610.15	C27H30O16	(Kaempferol-3-O-β-D-galactoside-4'O-β-D-glucoside)	Flavonoids	BioDeep	-	18700	13000	12400	12700	0.0024±0.0074	20700	21200	19800	22200	0.0057±0.0028	23600	19500	27500	23600	0.0057±0.0079	66900	71800	78100	89700	0.0115±0.0125	21300	36100	34100	31600	0.0064±0.013	-	-
kz005088	[M+H]+	479.12	317.066	478.11	C22H22O12	Tricetin?4'-methyl?ether-3'-O-β-D-glucoside	Flavonoids	BioDeep	-	1180000	1340000	1200000	1220000	0.2038±0.075	4240000	4080000	3680000	4440000	0.1112±0.0878	1370000	1360000	1460000	1490000	0.3452±0.1636	1530000	1340000	1250000	1400000	0.208±0.1615	96000	87400	121000	118000	0.0219±0.0333	-	-
kz005132	[M-H]-	593.13	285.044	594.14	C30H26O13	Luteolin-caffeoyl-O-rhamnoside	Flavonoids	BioDeep	70-16-6	2720000	3150000	2770000	2900000	0.476±0.2064	32300000	30300000	31800000	31500000	0.8515±0.1758	6390000	5860000	58500000	6320000	1.4836±0.4991	18000000	18200000	18400000	18900000	2.7704±0.5657	9710000	10300000	10000000	9620000	2.0595±0.5283	-	-
kz005133	[M+H]+	609.14	287.066	608.14	C27H28O16	Luteolin-O-glucuronate-O-rhamnoside	Flavonoids	BioDeep	-	-	-	-	-	-	-	-	-	-	-	111000	139000	140000	146000	0.0326±0.041	-	-	-	-	-	-	-	-	-	-	-	-
kz005136	[M-H]-	469.33	469.33	470.34	C30H46O4	Camaldulenic acid	Terpenoids	BioDeep	71850-15-2	3920000	4390000	3820000	4250000	0.6759±0.3805	80200000	76900000	83200000	84400000	2.1953±0.561	627000	5060000	523000	536000	0.1331±0.112	19600000	19900000	19600000	20400000	2.9965±0.5036	9280000	9150000	9170000	8860000	1.8951±0.4932	-	-
kz005137	[M+H]+	193.04	133.03	192.04	C10H8O4	Isoscopoletin (6-Hydroxy-7-Methoxycoumarin)	Coumarins	BioDeep	776-86-3	867000	897000	834000	839000	0.0142±0.0076	163000	211000	231000	165000	0.0052±0.0093	136000	115000	104000	108000	0.0281±0.0316	238000	268000	271000	273000	0.0396±0.0264	42900	40300	52000	38700	0.009±0.0101	-	-
kz00	[M-	44	28	450.1	C21H22	Dunalianoside C	Phenoli	Bio	-	39	42	34	37	0.0636±0.0	5280	5470	5800	6050	0.1528±0.0	391000	34	3530	380000	0.089±0.0422	293000	292000	2870000	309000	0.445±0.02	99400	1100	8300	9460	0.0201±0.025	-	-

5186	H]-	9.11	7.06	2	O11		c acids	Deep		8000	7000	3000	3000	719	00	00	00	00	763		2000	00			0	0		0	99		00	0	0	7		
kz005187	[M-H]-	449.11	287.06	450.12	C21H22O11	Dunalianoside B	Phenolic acids	BioDeep	-	3070000	3430000	3500000	3420000	0.0554±0.0252	1350000	1280000	1410000	1390000	0.3671±0.0996	112000	1120000	96500	113000	0.0263±0.0183	1500000	1370000	1560000	1540000	0.225±0.1257	51000	49600	39200	39700	0.0093±0.014	-	-
kz005198	[M-H]-	593.19	285.00	594.19	C28H34O14	Poncirin(Isosakuranetin-7-neohesperidoside)	Flavonoids	BioDeep	-	2810000	2950000	2950000	3010000	0.484±0.1518	3250000	3170000	3300000	3450000	0.8905±0.2029	6620000	6310000	6180000	6210000	1.5387±0.3688	18700000	18300000	18100000	19200000	2.7999±0.1837	10000000	10200000	10500000	9910000	2.1101±0.0251	C09830	--
kz005200	[M-H]-	609.18	301.00	610.19	C28H34O15	Hesperetin 7-O-neohesperidoside(Neohesperidin)	Flavonoids	BioDeep	-	340000	357000	325000	336000	0.0561±0.0284	94600	102000	104000	88100	0.0263±0.0217	565000	566000	578000	635000	0.1425±0.0791	1060000	1040000	1040000	1130000	0.1609±0.018	798000	788000	762000	738000	0.1604±0.0684	C09806	ko00941
kz005202	[M-H]-	287.06	259.00	288.06	C15H12O6	Dihydrokaempferol	Flavonoids	BioDeep	-	394000	424000	380000	389000	0.0066±0.0033	16700	17700	16800	18500	0.0047±0.0023	576000	522000	573000	535000	0.1341±0.058	206000	196000	208000	215000	0.0311±0.0083	15200	19000	20900	20000	0.0039±0.0048	C00974	ko00941,ko01100,ko01110
kz005211	[M+H]+	343.12	313.08	342.11	C19H18O6	5,7,8,4'-Tetramethoxyflavone	Flavonoids	BioDeep	-	14000	16600	14700	14900	0.0025±0.0012	2270	1910	1300	1420	0.0005±0.0013	1850	2160	1960	2210	0.0005±0.0005	2460	2660	2540	2970	0.0004±0.0002	1270	1360	2250	1320	0.0003±0.0009	-	-
kz005225	[M+H]+	595.17	449.10	594.16	C27H30O15	Luteolin 7-O-neohesperidoside(Lonicerin)	Flavonoids	BioDeep	2739-72-2	6470	7470	9360	5570	0.0012±0.0033	7790	12500	11800	8580	0.0028±0.0065	105000	89500	120000	132000	0.0271±0.0438	11000	12600	10300	11600	0.0017±0.0015	24700	20300	25500	19000	0.0046±0.0061	C12630	ko00944
kz	[M	6	4	60	C28	Neodiosm	Flav	Bi	-	4	5	5	5	0.084	22	23	21	25	0.062	984	9	43	686	0.0018	574	220	2110	463	0.000	7450	62	67	59	0.0014	-	-

005226	+H]+	09.18	63.12	8.17	H32O15	in (Diosmetin-7-O-Neohesperidoside)	onoids	oDeep		610000	770000	150000	070000	9±0.0585	3000	1000	7000	8000	8±0.0466	0	000	60	0	±0.0059	0	0		0	5±0.0026		80	50	10	±0.0014		
kz005247	[M+H]+	471.20	425.00	470.19	C26H30O8	Limonin	Others	BioDeep	1180-71-8	1240000	1480000	1340000	1350000	0.0223±0.0097	157000	142000	160000	148000	0.041±0.0193	54700	63600	57700	65700	0.0147±0.0135	25000	21900	22600	22400	0.0035±0.0023	194000	185000	193000	190000	0.0396±0.013	C03514	--
kz005355	[M-H]-	331.10	153.02	332.11	C14H20O9	Koaburaside	Phenolic acids	BioDeep	-	2260000	2330000	2150000	2100000	0.0365±0.0259	149000	144000	135000	135000	0.0381±0.0234	412000	415000	423000	437000	0.1025±0.0309	517000	498000	492000	510000	0.076±0.0173	214000	209000	202000	212000	0.0435±0.022	-	-
kz005370	[M-H]-	359.15	180.08	360.16	C20H24O6	(+)-Isolariciresinol	Lignans	BioDeep	548-29-8	1070000	1030000	1010000	1020000	0.0171±0.0129	158000	191000	197000	189000	0.005±0.0047	56700	48000	54700	51100	0.0128±0.0082	213000	218000	186000	194000	0.0306±0.0261	9310	9100	8310	8720	0.0018±0.0013	-	-
kz005373	[M-H]-	419.17	404.15	420.18	C22H28O8	Lyoniresinol	Lignans	BioDeep	14464-90-5	1860000	1520000	2050000	1650000	0.0291±0.054	468000	457000	554000	624000	0.0142±0.0192	236000	211000	201000	260000	0.0551±0.0591	279000	197000	228000	226000	0.035±0.0511	16200	21000	8880	13900	0.0031±0.0107	-	-
kz005375	[M-H]-	521.20	359.15	522.21	C26H34O11	Isolariciresinol 9'-O-Glucoside	Lignans	BioDeep	63358-12-3	4870000	5250000	5130000	4910000	0.0832±0.0246	1880000	2090000	2170000	2090000	0.0557±0.0327	541000	534000	533000	531000	0.13±0.0158	1780000	1810000	1670000	1780000	0.2654±0.0968	545000	548000	586000	458000	0.1109±0.0907	-	-
kz005376	[M-H]-	521.20	371.15	522.21	C26H34O11	5'-methoxyisolariciresinol-9'-O-β-D-xylopyranoside	Lignans	BioDeep	-	66000	79800	80900	68800	0.0122±0.0123	14400	17800	19200	12700	0.0043±0.0083	-	-	-	-	-	-	-	-	-	-	-	-	-	-	-	-	-

kz005377	[M-H]-	551.21	419.17	552.22	C27H36O12	lyoniresinol-9'-O-β-D-xylopyranoside	Lignans	BioDeep	-	836000	872000	789000	957000	0.1426±0.1417	261000	249000	265000	263000	0.0702±0.0096	-	-	-	-	-	88900	83200	84600	89700	0.0131±0.0027	-	-	-	-	-	-	-
kz005378	[M-H]-	551.21	389.17	552.22	C27H36O12	5'-methoxyisolariciresinol-9'-O-β-D-glucopyranoside	Lignans	BioDeep	-	68500	69700	65500	58500	0.0108±0.0119	25300	33600	38000	30900	0.0086±0.0142	155000	129000	13300	154000	0.0347±0.0284	236000	229000	227000	232000	0.0348±0.0088	166000	19400	15800	14800	0.0346±0.0404	-	-
kz005385	[M-H]-	293.21	293.21	294.22	C18H30O3	Machilusolide D	Others	BioDeep	-	368000	384000	344000	348000	0.0596±0.0419	186000	198000	175000	187000	0.0505±0.0325	199000	187000	202000	226000	0.0495±0.0371	538000	519000	457000	500000	0.0759±0.0526	646000	63300	67400	64500	0.135±0.0278		
kz005389	[M+H]+	371.21	209.15	370.20	C19H30O7	Byzantionoside A	Others	BioDeep	-	1530000	1380000	1410000	1500000	0.2408±0.2528	503000	425000	465000	367000	0.1191±0.1614	86800	79600	9140	58800	0.0192±0.0354	269000	268000	290000	275000	0.0416±0.022	42000	4540	3400	3530	0.0082±0.0119		

Table S2. List of the differential metabolites (DEMs) compared between pairwise species based on OPLS-DA analysis.

RRT-F/RSS-F					RRT-F/RLM-F				
Index	Compounds	Class	VIP	Fold_Change	Index	Compounds	Class	VIP	Fold_Change
kz000047	Mandelic acid	Phenolic acids	1.10636	0.00035	kz004698	7S,8R-threo-3′,9,9′-trihydroxy-3-methoxy-4′,7-epoxy-neolignan-4-O-α-L-rhamnopyranoside	Lignans	1.08041	0.00001
kz004768	monogalloyl-diglucose	Phenolic acids	1.10627	0.00039	kz000530	Brevifolin carboxylic acid	Phenolic acids	1.08004	0.00003
km1437	4-Aminobenzoic acid	Phenolic acids	1.10603	0.00075	kz002211	Quercetin 3-O-β-D-xylopyranoside	Flavonols	1.08053	0.00005
kz001820	benzoylmalic acid	Phenolic acids	1.10619	0.00078	kz000881	Avicularin	Flavonols	1.08051	0.00006
kz0029	Patuletin-3-O-β-D-glucopyranoside	Flavones	1.105	0.00125	kz0005	5-O-Galloylshikimic acid	Phenolic acids	1.080	0.00009

83			98	
kz0000			1.106	
55	3,4,5-Trimethoxycinnamic acid	Phenolic acids	25	0.00173
kz0005			1.105	
96	β-D-Furanofructosyl-α-D-(6-mustard acyl)glucoside	Phenolic acids	92	0.00174
kz0045			1.105	
96	Methyl neochebulagate	Others	37	0.00204
kz0022			1.106	
95	(-)-Syringaresinol-4-O-β-D-monO-glucoside	Lignans	14	0.00232
kz0029			1.105	
81	Diosmetin-7-O-glucuronide	Flavones	98	0.00240
kz0045			1.103	
95	Geraniin	Phenolic acids	26	0.00247
kz0031			1.105	
04	Nobotanin D	Phenolic acids	46	0.00309
kz0017			1.104	
50	Tercatain	Phenolic acids	14	0.00314
kz0029			1.105	
75	Acacetin-7-O-glucuronide	Flavones	78	0.00374
kz0013			1.104	
07	Phthalic acid	Organic acids	19	0.00388
kz0005			1.105	
75	3,5-Di-O-galloylshikimic acid	Phenolic acids	31	0.00414
kz0035			1.103	
04	Geraniinic acid C	Phenolic acids	04	0.00446
kz0037			1.105	
97	3-Prenyl-4-O-β-D-glucopyranosyloxy-4-hydroxyl-benzoic acid	Phenolic acids	09	0.00506
kz0008			1.102	
86	Myricetin-3-O-α-L-arabinopyranoside	Flavonols	58	0.00598
kz0005			1.105	
02	Coniferaldehyde	Phenolic acids	55	0.00666
kz0009			1.104	
20	Quercetin-O-rutinoside-hexose	Flavonols	06	0.01014
kz0016			1.106	
84	Tetramethylluteolin (3',4',5,7-Tetramethoxyflavone)	Flavones	20	0.01259
kz0005			1.105	
36	5-O-Galloylshikimic acid	Phenolic acids	55	0.02368
kz0005			1.104	
37	3-O-Galloylshikimic acid	Phenolic acids	49	0.02590
kz0005			1.105	
05	Coniferyl alcohol	Phenolic acids	74	0.02829
kz0031	Clemaphenol A	Lignans	1.096	0.03618

36			45	
kz0053			1.080	
77	lyoniresinol-9'-O-β-D-xylopyranoside	Lignans	50	0.00012
kz0020			1.080	
63	Galloyl-HHDP(Hexahydroxydiphenoyl)-glueopy ranose	Phenolic acids	49	0.00015
kz0037			1.080	
17	Maplexin C (2,3-Di-O-Galloyl-1,5-Anhydro-D-Glucitol)	Phenolic acids	23	0.00016
kz0031			1.080	
03	Gemin D	Phenolic acids	48	0.00016
kz0005			1.080	
32	Protocatechuic acid-4-glucoside	Phenolic acids	46	0.00016
kz0008			1.080	1325.0000
72	Myricetin	Flavonols	52	0
kz0021			1.080	
44	Angelicin	Coumarins	26	645.75000
kz0037			1.080	
55	Quercetin-3-sambubioside	Flavonols	16	580.50000
kz0041			1.080	
72	Quercetin 3-O-β-D-xylopyranosyl(1→2)-β-D-galactopyranoside	Flavonols	49	518.25000
kz0005			1.080	
17	Sinapyl alcohol	Phenolic acids	18	483.50000
kz0012			1.079	
84	Oxaloacetic acid	Organic acids	48	372.75000
kz0018			1.079	
64	vnilloylcaffeoyltartaric acid	Phenolic acids	82	263.00000
kz0033			1.079	
62	Tubuloside C	Phenolic acids	98	174.42778
kz0038			1.074	
33	Quercetin-O-pentosyl-O-rhamnoside-O-glucoside	Flavonols	73	133.65000
kz0002			1.079	
78	Indole 3-acetic acid (IAA)	Alkaloids	48	127.50000
kz0010			1.077	
14	1-Methylhistamine	Others	50	109.82500
kz0031			1.075	
66	6-Hydroxy-7-methoxycoumarin	Coumarins	82	65.23669
kz0007			1.079	
97	Tetahydroxy-flavone-7-O-β-D-glucuronide	Flavones	11	58.75706
kz0017			1.080	
02	Kaempferol-3-O-β-D-glucuronide	Flavonols	18	51.27709
kz0007			1.079	
98	Luteolin-7-O-glucuronide	Flavones	88	43.19751
kz0029	Scutellarin(Scutellarein-7-O-glucuronide)	Flavones	1.079	43.04054

47			78		76			34	
kz0010			1.101		kz0030			1.077	
76	Ethyl gallate	Phenolic acids	39	0.03892	98	3-O-Digalloyl quinic acid	Phenolic acids	59	35.59633
kz0010			1.100		kz0009			1.080	
74	Gallic acid	Phenolic acids	48	0.07032	06	Kaempferol-3-O-robinobioside(Biorobin)	Flavonols	34	29.82635
kz0005			1.105		kz0013			1.067	
77	Hexahydroxydiphenoylglucose	Phenolic acids	29	0.08116	80	Quercetin-O-rutinoside-O-rhamnoside	Flavonols	34	29.42529
kz0020			1.093		kz0009			1.080	
63	Galloyl-HHDP(Hexahydroxydiphenoyl)-glueopy ranose	Phenolic acids	72	0.08921	05	Kaempferol-3-O-rutinoside(Nicotiflorin)	Flavonols	34	28.62360
kz0031			1.092		kz0038			1.080	
03	Gemin D	Phenolic acids	89	0.09405	50	(-)-secoisolariciresinol 4-O-β-D-giucopyranoside	Lignans	09	28.31001
kz0040			1.104		kz0036			1.074	
15	Cis-p-coumaric acid 4-O-glucoside	Others	95	0.09540	93	Quercetin-3-O-(2-O-α-L-rhamnopyranosyl)-β-D-galactopyranoside	Flavonols	51	22.94023
kz0025			1.102		kz0041			1.077	
48	p-Coumaric acid-O-glycoside	Phenolic acids	14	0.09823	71	Kaempferol 3-O-β-D-neohesperidoside	Flavonols	11	22.46246
km053			1.094		kz0047			1.079	
2	Delphinidin 3-glucoside (Mirtillin)	Anthocyanins	39	0.09850	03	secoisolariciresinol 9-O-β-D-glucopyranoside	Lignans	58	22.30068
kz0037			1.100		kz0029			1.073	
48	7'-O-Sinapoyljasminoside L	Terpenoids	89	0.10582	90	Luteolin-7-O-rutinoside	Flavones	96	18.86159
kz0005			1.092		kz0009			1.064	
07	Syringic Aldehyde	Phenolic acids	22	0.11032	07	Kaempferol-3-O-glucoside-7-O-rhamnoside	Flavonols	98	16.07401
kz0052			1.093		kz0052			1.072	
11	5,7,8,4'-Tetramethoxyflavone	Flavones	68	0.11462	25	Luteolin 7-O-neohesperidoside(Lonicerin)	Flavones	51	15.46588
kz0009			1.105		kz0025			1.061	
15	6-Hydroxykaempferol-7,6-O-Diglucoside	Flavonols	26	0.11748	60	6-O-feruloyl-α-glucose	Phenolic acids	01	14.36250
kz0013			1.099		kz0005			1.079	
12	Shikimic acid	Organic acids	24	0.12020	05	Coniferyl alcohol	Phenolic acids	73	14.20085
kz0031			1.095		kz0052			1.080	
06	Cuspinin	Phenolic acids	99	0.12741	02	Dihydrokaempferol	Flavanols	01	13.90044
kz0037			1.097		kz0031			1.078	
16	2,4-Di-O-Galloyl-1,5-Anhydro-D-Glucitol;(Maplexin D)	Phenolic acids	41	0.13066	51	Ailantinol E	Others	81	13.51201
kz0000			1.092		kz0038			1.072	
52	Ethyl 3,4-Dihydroxybenzoate (Ethyl protocatechuate)	Phenolic acids	67	0.13488	09	Sieboldin	Chalcones	36	12.44489
kz0007			1.098		kz0009			1.079	
03	Delphinidin-3-O-glucoside (Mirtillin)	Anthocyanins	65	0.13499	12	Isorhamnetin-3-O-rutinoside (Narcissin)	Flavonols	81	11.63443
kz0004			1.104		kz0037			1.073	
91	p-Coumaric acid	Phenolic acids	64	0.14848	97	3-Prenyl-4-O-β-D-glucopyranosyloxy-4-hydroxyl-benzoic acid	Phenolic acids	60	11.37975
kz0005			1.074		kz0005			1.070	
30	Brevifolin carboxylic acid	Phenolic acids	20	0.15421	92	Rosmarinyl Glucoside	Phenolic acids	59	11.18904
kz0025			1.088		kz0011			1.072	
54	p-Hydroxycinnamic acid	Phenolic acids	99	0.15795	13	Lumichrome	Alkaloids	57	11.05833
kz0000	2-(Formylamino)benzoic acid	Phenolic acids	1.103	0.15923	kz0027	Sexangularetin 3-glucoside-7-rhamnoside	Flavonols	1.067	11.04457

49			33	
kz0030			1.078	
98	3-O-Digalloyl quinic acid	Phenolic acids	03	0.16514
kz0000			1.064	
66	Feruloyl syringic acid	Phenolic acids	16	0.16592
kz0037			1.099	
19	Methyl 4,6-di-O-galloyl-D-glucoside	Phenolic acids	86	0.16675
kz0008			1.102	
41	Luteolin-O-sinapoylhexoside	Flavones	36	0.16868
kz0005			1.098	
04	Caffeic acid	Phenolic acids	85	0.17278
kz0053			1.103	
70	(+)-Isolariciresinol	Lignans	68	0.17797
kz0037			1.092	
17	Maplexin C (2,3-Di-O-Galloyl-1,5-Anhydro-D-Glucitol)	Phenolic acids	11	0.17833
kz0046	7S,8R-threo-3′,9,9′-trihydroxy-3-methoxy-4′,7-epoxy-neolignan-4-O-α-		1.099	
98	L-rhamnopyranoside	Lignans	77	0.18070
kz0010			1.103	
75	Methyl gallate	Phenolic acids	24	0.18342
kz0009			1.094	
14	6-Hydroxykaempferol-3,6-O-Diglucoside	Flavonols	35	0.18419
kz0047			1.102	
38	4,7,9,9′-Tetrahydroxy-3,3′-dimethoxy-8-O-4′-neolignan	Others	28	0.18652
kz0026			1.098	
78	Quercetin-3-O-(6''-O-acetyl)-galactoside	Flavonols	20	0.19369
kz0009			1.104	
95	Syringaresinol	Lignans	26	0.19482
kz0004			1.087	
84	Vanillin	Phenolic acids	64	0.20000
km111			1.095	
3	5-O-p-Coumaroyl shikimic acid	Phenolic acids	34	0.20752
kz0020			1.098	
08	3,5,7,4'--Tetrahydroxy-Coumaronochromone	Others	32	0.20808
kz0008			1.100	
64	Kaempferol	Flavonols	31	0.21028
kz0007			1.085	
00	Cyanidin-3-O-glucoside (Kuromanin)	Anthocyanins	45	0.21412
kz0053			1.090	
76	5'-methoxyisolariciresinol-9'-O-β-D-xylopyranoside	Lignans	68	0.21692
kz0051			1.095	
37	Isoscopoletin	Coumarins	04	0.22403
kz0046	5,7,3',4',5′-pentahydroxydihydroflavone	Flavanones	1.090	0.22663

88			65	
kz0007			1.078	
91	Apigenin 7-O-glucoside(Cosmosiin)	Flavones	83	10.39837
kz0021			1.072	
96	2'-Hydoxy,5-methoxy Genistein-O-rhamnosyl-glucoside	Isoflavones	41	9.87071
kz0007			1.076	
92	Apigenin 5-O-glucoside	Flavones	29	9.69072
km060			1.073	
2	Genistein 7-O-Glucoside (Genistin)	Isoflavones	55	9.38185
kz0050			1.074	
55	Galangin-7-glucoside	Flavones	58	9.24528
kz0009			1.077	
08	Quercetin-3-O-robinobioside	Flavonols	96	8.35443
kz0001			1.078	
90	Arbutin	Others	08	8.18991
kz0016			1.079	
37	Oresbiusin A	Phenolic acids	12	8.00731
kz0009			1.078	
09	Quercetin-3-O-rutinoside (Rutin)	Flavonols	64	7.66157
kz0005			1.080	
57	Glucosyringic Acid	Phenolic acids	25	7.63619
kz0026			1.078	
04	Kaempferol-3-O-(6''-acetyl)-glucoside	Flavonols	37	7.05004
kz0041			1.073	
73	Quercetin 3-O-neohesperidoside	Flavonols	90	6.88296
kz0011			1.077	
58	D-Pantothenic Acid	Vitamins	73	6.43132
kz0009			1.077	
11	Quercetin-7-O-rutinoside	Flavonols	55	6.37064
kz0005			1.079	
40	3,4,5-Trimethoxyphenyl-β-D-Glucopyranoside	Phenolic acids	71	5.98788
kz0022			1.071	
95	Syringaresinol-4'-O-β-D-monO-glucoside	Lignans	96	5.97214
kz0025			1.077	
46	Isosalicylic acid O-glycoside	Phenolic acids	69	5.56543
kz0040			1.065	
35	3-Indoleacrylic acid	Alkaloids	07	5.52196
kz0037			1.067	
94	Syringic Aldehyde-glucoside	Phenolic acids	16	5.13809
kz0025			1.075	
56	4-O-glucosyl-4-hydroxybenzoic acid	Phenolic acids	43	5.01519
kz0002	Citramalate	Organic acids	1.074	4.71366

65			71		96			03	
kz0037			1.104		kz0005			1.070	
14	6-O-Galloylglucose	Phenolic acids	43	0.23063	02	Coniferaldehyde	Phenolic acids	93	4.65391
kz0034			1.105		kz0004			1.078	
97	3-O-Galloyl-β-D-glucose	Phenolic acids	90	0.23135	12	Aucubin	Terpenoids	14	4.57824
kz0031			1.105		kz0024			1.058	
00	5-O-Galloylhamamelose	Phenolic acids	20	0.23149	85	3-methoxy-juglone	Quinones	75	4.44034
kz0006			1.066		kz0035			1.064	
98	Cyanidin-3-O-galactoside	Anthocyanins	56	0.23273	17	Glucosyloxybenzoic acid	Phenolic acids	19	4.19065
kz0004			1.104		kz0005			1.075	
24	Eriodictyol	Flavanones	14	0.23327	33	2,5-Dihydroxy benzoic acid O-hexside	Phenolic acids	60	4.04869
kz0005			1.102		kz0011			1.077	
73	p-Coumaroylferuloyltartaric acid	Phenolic acids	89	0.23624	63	Riboflavin	Vitamins	40	3.97904
kz0000			1.101		kz0004			1.075	
40	N,N'-Bis(Sinapoyl)Spermidine	Alkaloids	80	0.23754	66	4-Hydroxybenzaldehyde	Phenolic acids	08	3.93175
kz0009			1.104		kz0009			1.076	
92	Pinoresinol	Lignans	40	0.23880	73	Epigallocatechin (EGC)	Flavanols	12	3.60094
kz0022			1.104		kz0009			1.068	
19	Epipinoresinol	Lignans	44	0.24796	74	Gallocatechin	Flavanols	22	3.36138
kz0008			1.103		kz0038			1.046	
92	6-Hydroxykaempferol-7-O-glucoside	Flavonols	95	0.24889	15	Kaempferol 3-O-(6''-trans-p-Coumaroyl)-β-D-glucopyranoside	Flavonols	55	2.96432
kz0047			1.056		kz0034			1.076	
03	secoisolariciresinol 9-O-β-D-glucopyranoside	Lignans	03	0.24954	98	2-O-Galloyl-β-D-glucose	Phenolic acids	91	2.94830
kz0015			1.065		kz0010			1.076	
84	Quercetin-3′,4′-dimethyl ether	Flavonols	24	0.25600	20	4-Methyl-5-thiazoleethanol	Others	97	2.94695
kz0022			1.105		kz0013		Proanthocyani	1.075	
14	isohyperoside	Flavonols	19	0.25660	40	Procyanidin C2	dins	47	2.91212
kz0011			1.095		kz0005			1.075	
75	Esculetin	Coumarins	71	0.25774	42	1-O-Galloyl-β-D-glucose	Phenolic acids	49	2.86242
kz0031			1.097		kz0038			1.009	
08	mlyricetin 3-O-B-D-glucopyranoside	Flavonols	46	0.25832	12	Isorhamnetin O-malonylglucoside	Flavonols	20	2.81997
kz0004			1.102		kz0026			1.075	
96	Vanillic acid	Phenolic acids	79	0.25852	78	Quercetin-3-O-(6''-O-acetyl)-galactoside	Flavonols	90	2.61980
km007			1.103		kz0000			1.071	
1	Quercetin 3-O-galactoside (Hyperin)	Flavonols	60	0.25922	66	Feruloyl syringic acid	Phenolic acids	87	2.58701
kz0006			1.086		kz0005			1.022	
13	1,2,3,6-Tetra-O-Galloyl-β-D-Glucose	Phenolic acids	41	0.26009	00	Riboprine	Phenolic acids	73	2.53374
kz0005			1.015		kz0025			1.060	
52	4-O-Caffeoyl quinic acid (criptochlorogenic acid)	Phenolic acids	31	0.26067	52	Sinapic acid-glycoside	Phenolic acids	92	2.51813
km022			1.102		km054			1.079	
8	Quercetin 3-O-glucoside (Isotrifoliin)	Flavonols	75	0.26863	8	Dihydroquercetin (Taxifolin)	Flavonols	10	2.47179
km081	Morin	Flavonols	1.105	0.26893	kz0011	Esculin(6,7-DihydroxyCoumarin-6-glucoside)	Coumarins	1.023	2.40571

4			68	
km032			1.100	
9	3-(4-Hydroxyphenyl)propionic acid	Phenolic acids	13	0.26950
kz0004			1.099	
95	3-(4-Hydroxyphenyl)-propionic acid	Phenolic acids	66	0.26953
kz0017			1.063	
42	Methyl Brevifolincarboxylate	Others	41	0.27165
kz0025			1.098	
57	4-O-glucosyl-3,4-dihydroxybenzyl alcohol	Phenolic acids	66	0.27574
kz0012			1.104	
81	Citraconic acid	Organic acids	70	0.27993
km081			1.084	
8	Myricetin 3-O-rhamnoside (Myricitrin)	Flavonols	93	0.28405
kz0000			1.102	
53	Isoferulic Acid	Phenolic acids	24	0.28487
kz0030			1.103	
99	Di-O-Glucose-quinic acid	Phenolic acids	96	0.28567
kz0052			1.102	
00	Hesperetin 7-O-neohesperidoside(Neohesperidin)	Flavanones	39	0.28623
kz0016			1.096	
95	wogonoside	Flavones	26	0.29226
km126			1.104	
9	Di-O-methylquercetin	Flavonols	23	0.29287
kz0011			1.093	
84	Skimmin	Coumarins	58	0.29456
km054			1.100	
8	Dihydroquercetin (Taxifolin)	Flavonols	37	0.29535
kz0050			1.076	
46	Dihydrokaempferol-3-O-β-D-glucoside	Flavanols	06	0.29552
kz0047			1.100	
35	Ligraminol E	Others	45	0.29897
kz0053			1.087	
73	Lyoniresinol	Lignans	08	0.29915
kz0053			1.101	
77	lyoniresinol-9'-O-β-D-xylopyranoside	Lignans	51	0.30052
kz0012			1.094	
85	2-Methylsuccinic acid	Organic acids	31	0.30138
kz0053			1.094	
89	Byzantionoside A	Others	10	0.30241
kz0029			1.102	
33	Ixerin D	Terpenoids	07	0.30375
km064	Hesperetin 7-rutinoside (Hesperidin)	Flavanones	1.091	0.30467

88			08	
kz0000			1.078	
56	Salicylic acid glucoside	Phenolic acids	10	2.35668
kz0028			1.077	
62	Dihydrosedinine	Alkaloids	36	2.26505
km143			1.047	
7	p-Aminobenzoate	Phenolic acids	33	2.22180
kz0002			1.070	
40	Trans-Zeatin-9-N-Glucoside	Alkaloids	23	2.21678
kz0053			1.060	
78	5'-methoxyisolariciresinol-9'-O-β-D-glucopyranoside	Lignans	73	2.17773
kz0009			1.068	
04	Tiliroside	Flavonols	81	2.16624
kz0051			1.078	
98	Poncirin(Isosakuranetin-7-neohesperidoside)	Flavanones	10	2.16041
kz0011			1.053	
99	Indole-3-carboxaldehyde	Alkaloids	60	2.14719
kz0013			1.048	
08	D-Xylonic acid	Organic acids	95	2.12523
kz0051			1.071	
32	Luteolin-caffeoyl-O-rhamnoside	Flavones	54	2.11612
kz0031			1.056	
09	Mearnsitrin	Flavones	61	2.09911
kz0005			1.065	
15	Sinapinaldehyde	Phenolic acids	90	2.08977
kz0025			1.058	
57	4-O-glucosyl-3,4-dihydroxybenzyl alcohol	Phenolic acids	75	2.06623
kz0046			1.076	
78	Annuionone D	Others	08	2.06279
kz0013			1.055	
12	Shikimic acid	Organic acids	14	0.48580
km121			1.022	
1	Chrysoeriol 7-O-hexoside	Flavones	71	0.47626
kz0002			1.080	
01	D-galacitol	Others	02	0.47400
kz0008			1.075	
89	Spiraeoside	Flavonols	84	0.47048
kz0004			1.076	
41	Hesperetin 5-O-glucoside	Flavanols	49	0.46202
kz0008			1.070	
00	Chrysoeriol-5-O-hexoside	Flavones	56	0.45729
kz0015	Stachydrine	Alkaloids	1.070	0.45475

0			03	
kz0033			1.061	
70	Swertiamarin	Terpenoids	11	0.30468
kz0022			1.100	
21	Eucommin A	Lignans	02	0.30479
kz0005			1.101	
11	Ferulic acid	Phenolic acids	54	0.30875
km129			1.099	
3	Hesperetin O-malonylhexoside	Flavanones	61	0.31119
km130			1.093	
7	Isorhamnetin 5-O-hexoside	Flavonols	60	0.31510
kz0036			1.095	
90	Quercetin-3-O-α-L-rhamnopyranoside	Flavonols	11	0.32333
kz0038			1.100	
48	Cinchonain Ic	Flavanols	72	0.32438
kz0050			1.015	
37	Caffeoylferuloylshikimic?acid	Phenolic acids	54	0.33252
kz0004			1.076	
17	Phloretin	Chalcones	39	0.33271
kz0050			1.099	
88	Tricetin?4'-methyl?ether-3'-O-β-D-glucoside	Flavonols	63	0.33279
kz0008			1.100	
12	Tricin O-saccharic acid	Flavones	58	0.33559
kz0017			1.097	
39	Quercetin-3-O-(2''-galloyl)-β-D-glucoside	Flavonols	73	0.33727
kz0023			1.063	
67	Methyl dioxindole-3-acetate	Others	18	0.33733
kz0029			1.089	
76	Scutellarin(Scutellarein-7-O-glucuronide)	Flavones	01	0.33953
kz0003			1.091	
10	Pyruvic acid	Organic acids	78	0.34171
kz0028			1.068	
07	Dimethylmalonic acid	Organic acids	27	0.34427
kz0038			1.097	
29	Quercetin 3-O-(6’’-galloyl)-β-D-galactopyranoside	Flavonols	32	0.34535
kz0013		Proanthocyani	1.101	
35	Procyanidin B1	dins	16	0.34613
kz0041			1.104	
70	Rhamnetin 3-O-β-D-Glucoside	Flavonols	87	0.34947
kz0001			1.099	
47	Methylquercetin O-hexoside	Flavonols	10	0.35082
kz0005	3,4-Dimethoxycinnamic acid	Phenolic acids	1.099	0.35137

98			83	
kz0046			1.062	
97	Taxifolin-3'-O-β-D-glucoside	Flavones	19	0.45427
kz0002			1.032	
59	Thiamine	Vitamins	41	0.45184
kz0012			1.064	
85	2-Methylsuccinic acid	Organic acids	32	0.45123
kz0029			1.063	
77	Diosmetin-7-O-galactoside	Flavones	75	0.44965
kz0005			1.032	
96	β-D-Furanofructosyl-α-D-(6-mustard acyl)glucoside	Phenolic acids	79	0.44759
kz0052			1.064	
47	Limonin	Others	94	0.44677
km025			1.076	
8	(+)-cis,trans-Abscisic acid (ABA)	Others	78	0.43955
kz0017			1.038	
10	Stilbostemin B	Others	25	0.42844
km126			1.074	
9	3,7-Di-O-methylquercetin	Flavonols	49	0.42691
kz0012			1.063	
92	4-Acetamidobutyric acid	Organic acids	11	0.42113
kz0030			1.072	
38	3-O-(2-O-Acetyl-β-D-glucopyranosyl) oleanolic acid	Terpenoids	05	0.41982
kz0005			1.058	
39	3-Hydroxy-4-isopropylbenzylalcohol 3-glucoside	Phenolic acids	76	0.41242
kz0029			1.069	
10	Parthenolide	Terpenoids	96	0.38817
kz0010			1.046	
32	N-Acetyl-D-galactosamine	Others	64	0.38754
kz0008			1.070	
88	Quercetin-3-O-β-D-Galactoside (Hyperin)	Flavonols	94	0.38250
kz0012			1.070	
79	L-Homoserine	Organic acids	98	0.37917
kz0004			1.075	
79	Cinnamic acid	Phenolic acids	49	0.37014
kz0010			1.079	
91	Euscaphic acid	Terpenoids	62	0.36789
kz0003			1.065	
10	Pyruvic acid	Organic acids	39	0.36477
kz0005			1.040	
55	1-Caffeoylquinic acid	Phenolic acids	29	0.36258
kz0026	Eriodictyol-O-glucoside	Flavones	1.075	0.36000

14			32	
kz0029			1.102	
82	6-methoxykaempferol-3-O-glucoside	Flavonols	58	0.35328
kz0037			1.065	
18	2,3-Di-O-Galloyl-D-Glucose	Phenolic acids	28	0.35390
kz0038			1.079	
21	Kampferol 3-O-(6″-galloyl)-β-D-galactopyranoside	Flavonols	52	0.35449
kz0013			1.083	
08	D-Xylonic acid	Organic acids	82	0.35462
kz0004			1.097	
12	Aucubin	Terpenoids	02	0.35575
kz0028			1.082	
29	Isorhamnetin-7-O-glucoside	Flavonols	90	0.35596
kz0015			1.101	
81	Calceorioside B	Others	06	0.35807
kz0050			1.092	
79	Kaempferol-4'-O-β-D-glucopyranoside	Flavonols	11	0.35862
km140			1.089	
5	Nicotinic acid-hexoside	Vitamins	65	0.36025
kz0013		Proanthocyani	1.100	
32	Procyanidin B2	dins	45	0.36027
kz0008			1.100	
88	Quercetin-3-O-β-D-Galactoside (Hyperin)	Flavonols	78	0.36034
kz0013		Proanthocyani	1.101	
33	Procyanidin B3	dins	56	0.36107
kz0026			1.082	
04	Kaempferol-3-O-(6''-acetyl)-glucoside	Flavonols	76	0.36176
kz0038			1.090	
23	Kampferol 3-O-(2″-galloyl)-β-D-galactopyranoside	Flavonols	89	0.36201
kz0002			1.087	
93	Glutaric acid	Organic acids	97	0.36568
kz0009			1.010	
23	6-Hydroxykaempferol-3,6-O-Diglucoside-7-O-Glucuronic Acid	Flavonols	82	0.36753
kz0031			1.085	
51	Ailantinol E	Others	20	0.37079
kz0037			1.063	
21	Maplexin G	Phenolic acids	36	0.37100
kz0011			1.104	
56	Pyridoxine	Vitamins	00	0.37118
kz0007			1.068	
98	Luteolin-7-O-glucuronide	Flavones	35	0.37120
kz0005	3,4,5-Trimethoxyphenyl-β-D-Glucopyranoside	Phenolic acids	1.103	0.37131

73			47	
kz0005			1.046	
44	3-O-p-Coumaroyl quinic acid	Phenolic acids	15	0.35473
kz0035			1.070	
40	Dihydrokaempferol-7-O-glucoside	Flavonols	65	0.35232
kz0004			1.046	
17	Phloretin	Chalcones	33	0.34673
kz0010			1.052	
25	D-Glucurono-6,3-lactone	Others	24	0.34432
kz0047			1.077	
74	(2R)-Pinocembrin-7-neohesperidoside	Flavanones	02	0.33915
kz0000			1.071	
70	Anthranilate O-hexosyl-O-hexoside	Phenolic acids	41	0.33691
kz0037			1.036	
21	Maplexin G	Phenolic acids	49	0.33227
kz0002			1.063	
94	2-Hydroxyisocaproic acid	Organic acids	05	0.32836
kz0051			1.074	
87	Dunalianoside B	Phenolic acids	45	0.32303
kz0028			1.075	
53	Dihydroisopelletierine	Alkaloids	93	0.32101
kz0012			1.076	
68	2-Furanoic acid	Organic acids	69	0.31792
kz0006			1.023	
98	Cyanidin-3-O-galactoside	Anthocyanins	34	0.31296
kz0037			1.025	
93	Dihydromyricetin-O-glucoside	Flavanols	85	0.31035
kz0041			1.076	
33	L-Ascorbic acid	Vitamins	52	0.30850
kz0009			1.073	
15	6-Hydroxykaempferol-7,6-O-Diglucoside	Flavonols	67	0.30565
km022			1.080	
8	Quercetin 3-O-glucoside (Isotrifoliin)	Flavonols	05	0.30417
kz0022			1.079	
14	isohyperoside	Flavonols	83	0.29816
km068			1.074	
6	Kaempferol 3-O-rhamnoside (Kaempferin)	Flavonols	13	0.29358
kz0009			1.000	
23	6-Hydroxykaempferol-3,6-O-Diglucoside-7-O-Glucuronic Acid	Flavonols	44	0.29276
kz0004			1.073	
87	2,5-Dihydroxybenzoic acid	Phenolic acids	40	0.28956
kz0008	6-Hydroxykaempferol-7-O-glucoside	Flavonols	1.077	0.28914

40			09	
kz0012			1.103	
97	(Rs)-Mevalonic acid	Organic acids	81	0.37167
kz0041			1.086	
69	Isorhamnetin 3-O-β-D-Glucoside	Flavonols	88	0.37252
kz0017			1.075	
02	Kaempferol-3-O-β-D-glucuronide	Flavonols	07	0.37539
kz0038			1.060	
61	3-O-Trans-feruloyl euscaphic acid	Terpenoids	84	0.38225
kz0008			1.097	
00	Chrysoeriol-5-O-hexoside	Flavones	57	0.38358
kz0008			1.096	
84	Kaempferol-3-O-glucoside (Astragalin)	Flavonols	36	0.38464
kz0036			1.077	
36	Plantainoside A	Phenolic acids	43	0.38692
kz0009			1.103	
78	Catechin-(7,8-bc)-4β-(3,4-dihydroxyphenyl)-dihydro-2-(3H)-pyranone	Flavanols	10	0.38800
kz0002			1.097	
58	Nicotinate D-ribonucleoside	Vitamins	54	0.38914
kz0008			1.087	
66	Quercetin	Flavonols	10	0.39025
kz0010			1.102	
96	Choline	Alkaloids	16	0.39030
kz0004			1.105	
41	Hesperetin 5-O-glucoside	Flavanols	00	0.39111
kz0005			1.095	
55	1-Caffeoylquinic acid	Phenolic acids	02	0.39318
kz0000			1.065	
32	Caffeoylagmatine	Alkaloids	44	0.39482
kz0019			1.085	
73	Kaempferol-3-O-(cinnamoyl)-sophoroside-7-O-glucose	Flavonols	53	0.39576
kz0005			1.066	
15	Sinapinaldehyde	Phenolic acids	57	0.39937
kz0008			1.103	
89	Spiraeoside	Flavonols	29	0.40120
kz0009			1.101	
79	Catechin-(7,8-bc)-4α-(3,4-dihydroxyphenyl)-dihydro-2-(3H)-pyranone	Flavanols	74	0.40159
kz0007			1.077	
95	Luteolin-7-O-glucoside(Cynaroside)	Flavones	90	0.40453
kz0031			1.024	
58	Ailanindole	Alkaloids	34	0.40462
kz0005	Neochlorogenic acid(5-O-Caffeoylquinic acid)	Phenolic acids	1.075	0.40726

92			69	
kz0042			1.073	
33	Terminolic acid	Terpenoids	07	0.28813
kz0038			1.076	
63	Sanguisorbigenin	Terpenoids	05	0.28589
kz0031			1.074	
08	mlyricetin 3-O-B-D-glucopyranoside	Flavonols	62	0.28580
km007			1.077	
1	Quercetin 3-O-galactoside (Hyperin)	Flavonols	64	0.28473
kz0011			1.072	
84	Skimmin	Coumarins	96	0.28132
kz0007			1.020	
00	Cyanidin-3-O-glucoside (Kuromanin)	Anthocyanins	94	0.27875
km081			1.078	
4	Morin dihydrate	Flavonols	65	0.27494
kz0031			1.028	
16	2,4,6-trihydroxy benzoic acid	Organic acids	01	0.27070
kz0004			1.075	
89	Protocatechuic acid	Phenolic acids	60	0.26909
kz0013			1.076	
02	2,3-Dihydroxybenzoic Acid	Organic acids	89	0.26813
kz0000			1.078	
49	2-(Formylamino)benzoic acid	Phenolic acids	69	0.26803
kz0025			1.075	
54	p-Hydroxycinnamic acid	Phenolic acids	51	0.26639
kz0004			1.069	
40	Astilbin	Flavanols	12	0.26620
kz0011			1.067	
75	Esculetin	Coumarins	56	0.26272
kz0030			1.077	
99	Di-O-Glucose-quinic acid	Phenolic acids	95	0.25974
kz0050			1.066	
37	Caffeoylferuloylshikimic?acid	Phenolic acids	28	0.25718
kz0020			1.051	
08	3,5,7,4'--Tetrahydroxy-Coumaronochromone	Others	41	0.25682
kz0005			1.067	
48	Trans-3-O-p-coumaric quinic acid	Phenolic acids	15	0.25560
kz0012			1.070	
87	(S)-(-)-2-Hydroxyisocaproic acid	Organic acids	75	0.24349
kz0004			1.078	
24	Eriodictyol	Flavanones	24	0.24164
kz0011	Trigonelline	Alkaloids	1.078	0.24102

53			85		01			56	
kz0053			1.101		kz0004			1.066	
75	Isolariciresinol 9'-O-Glucoside	Lignans	50	0.40823	10	Sweroside	Terpenoids	47	0.24090
kz0050			1.101		kz0004			1.077	
78	Kaempferol-7-O-β-D-glucopyranoside	Flavonols	55	0.40937	91	p-Coumaric acid	Phenolic acids	10	0.23148
kz0046			1.088		kz0046			1.078	
97	Taxifolin-3'-O-β-D-glucoside	Flavones	59	0.40968	36	2α-hydroxypyracrenic acid	Terpenoids	15	0.22649
kz0009			1.056		kz0005			1.077	
74	Gallocatechin	Flavanols	50	0.41000	77	Hexahydroxydiphenoylglucose	Phenolic acids	56	0.21978
km068			1.093		kz0012			1.070	
6	Kaempferol 3-O-rhamnoside (Kaempferin)	Flavonols	33	0.41027	98	L-(+)-Tartaric acid	Organic acids	31	0.21813
kz0010			1.097		kz0026			1.067	
97	Betaine	Alkaloids	20	0.41856	87	Nortrachelogenin 4-O-β-D-glucoside	Lignans	04	0.21745
km068			1.102		kz0011			1.075	
3	Kaempferol 3-O-galactoside (Trifolin)	Flavonols	11	0.42220	56	Pyridoxine	Vitamins	41	0.21375
kz0007			1.059		kz0036			1.069	
97	Tetahydroxy-flavone-7-O-β-D-glucuronide	Flavones	13	0.42286	36	Plantainoside A	Phenolic acids	96	0.20950
km008			1.066		kz0017			1.070	
1	Genistein (4',5,7-Trihydroxyisoflavone)	Isoflavones	41	0.42395	06	Isololiolide	Others	91	0.20931
kz0047			1.102		kz0022			1.075	
71	Epicatechin glucoside	Flavanols	26	0.43593	20	Olivil-4’-O-β-D-glucoside	Lignans	66	0.20784
kz0009			1.081		kz0001			1.077	
97	Terpineol monO-glucoside	Lignans	55	0.43719	45	Formononetin	Isoflavones	91	0.20713
kz0005			1.099		kz0012			1.066	
01	Methyl p-coumarate	Phenolic acids	61	0.43878	89	Malic acid,D	Organic acids	09	0.19833
kz0052			1.100		kz0011			1.079	
02	Dihydrokaempferol	Flavanols	78	0.43919	54	Nicotinamide	Vitamins	61	0.19735
km023			1.070		kz0008			1.076	
5	Apigenin	Flavones	66	0.44050	64	Kaempferol	Flavonols	09	0.19324
km077			1.078		kz0047	2-Hydroxy-5,8,11,14,17-icosapentaenoyloxy]propyl-2-		1.079	
9	Luteolin 7-O-glucoside	Flavones	29	0.44130	62	(trimethylammonio)ethyl phosphate	Alkaloids	11	0.19282
kz0038			1.103		kz0000			1.062	
49	dihydrodehydrodiconiferyl alcohol 4-O-β-D-glucopyranosides	Lignans	13	0.44269	40	N,N'-Bis(Sinapoyl)Spermidine	Alkaloids	11	0.18492
kz0012			1.093		kz0017			1.055	
93	4-Guanidinobutyric acid	Organic acids	66	0.44386	42	Methyl Brevifolincarboxylate	Others	67	0.18314
kz0026			1.081		kz0047			1.079	
87	Nortrachelogenin 4-O-β-D-glucoside	Lignans	51	0.44481	38	4,7,9,9’-Tetrahydroxy-3,3’-dimethoxy-8-O-4’-neolignan	Others	29	0.18172
kz0013		Proanthocyani	1.068		kz0038			1.076	
40	Procyanidin C2	dins	09	0.44676	54	Roseoside	Others	58	0.18128
kz0026			1.087		kz0047			1.057	
07	Lariciresinol glucopyranoside	Others	34	0.44802	35	Ligraminol E	Others	65	0.17814
kz0052	Neodiosmin (Diosmetin-7-O-Neohesperidoside)	Flavones	1.088	0.45097	kz0010	Oleanolic acid 2-O-β-D-glucopyranoside	Terpenoids	1.076	0.17808

26			17		93			16	
kz0005			1.088		kz0040			1.077	
32	Protocatechuic acid-4-glucoside	Phenolic acids	22	0.45271	15	Cis-p-coumaric acid 4-O-glucoside	Others	11	0.17325
kz0031			1.077		kz0031			1.079	
09	Mearnsitrin	Flavones	31	0.45788	00	5-O-Galloylhamamelose	Phenolic acids	52	0.16981
kz0037			1.055		kz0042			1.076	
28	Geniposide	Terpenoids	29	0.46158	31	Isothankunic acid	Terpenoids	05	0.16889
kz0011			1.099		kz0034			1.079	
55	Nicotinic acid	Vitamins	80	0.46408	97	3-O-Galloyl-β-D-glucose	Phenolic acids	75	0.16566
kz0009			1.100		kz0037			1.079	
71	Catechin	Flavanols	57	0.47079	14	6-O-Galloylglucose	Phenolic acids	18	0.16176
kz0005			1.089		kz0029			1.079	
12	Syringic acid	Phenolic acids	83	0.47510	33	Ixerin D	Terpenoids	20	0.16125
kz0008			1.096		kz0025			1.077	
31	Diosmin	Flavones	26	0.47734	48	p-Coumaric acid-O-glycoside	Phenolic acids	97	0.15936
kz0038			1.009		kz0046			1.024	
50	(-)-secoisolariciresinol 4-O-β-D-giucopyranoside	Lignans	39	0.48009	30	2α-hydroxyursolic acid	Terpenoids	79	0.15640
kz0018			1.048		kz0010			1.075	
80	feruloylsinapoyltartaric acid	Phenolic acids	87	0.48454	75	Methyl gallate	Phenolic acids	96	0.15308
kz0053			1.055		kz0005			1.077	
78	5'-methoxyisolariciresinol-9'-O-β-D-glucopyranoside	Lignans	67	0.48741	73	p-Coumaroylferuloyltartaric acid	Phenolic acids	82	0.15084
kz0004			1.080		kz0000			1.070	
14	2-Picolylamine	Alkaloids	22	0.48826	47	Mandelic acid	Phenolic acids	22	0.15004
kz0011			1.098		kz0038			1.075	
77	Scoparone	Coumarins	16	0.49726	60	Caffeoyl hawthorn acid	Terpenoids	19	0.14956
kz0025			1.050		kz0038			1.076	
52	Sinapic acid-glycoside	Phenolic acids	57	0.50000	61	3-O-Trans-feruloyl euscaphic acid	Terpenoids	12	0.14647
kz0012			1.102		kz0037			1.059	
87	(S)-(-)-2-Hydroxyisocaproic acid	Organic acids	01	2.01836	18	2,3-Di-O-Galloyl-D-Glucose	Phenolic acids	57	0.14229
kz0050			1.001		kz0005			1.077	
55	Galangin-7-glucoside	Flavones	99	2.02191	54	Chlorogenic acid	Phenolic acids	10	0.14008
kz0024			1.102		kz0011			1.073	
19	Ergotamine	Alkaloids	62	2.11346	77	Scoparone	Coumarins	00	0.13825
kz0009			1.096		kz0010			1.077	
09	Quercetin-3-O-rutinoside (Rutin)	Flavonols	23	2.11908	97	Betaine	Alkaloids	24	0.13799
kz0046			1.087		kz0005			1.075	
78	Annuionone D	Others	72	2.11921	04	Caffeic acid	Phenolic acids	73	0.13758
kz0021			1.071		kz0052			1.077	
96	2'-Hydoxy,5-methoxy Genistein-O-rhamnosyl-glucoside	Isoflavones	37	2.12483	11	5,7,8,4'-Tetramethoxyflavone	Flavones	94	0.13588
kz0001			1.104		km111			1.070	
45	Formononetin	Isoflavones	45	2.16086	3	5-O-p-Coumaroyl shikimic acid	Phenolic acids	54	0.13398
kz0001	Naringenin 7-O-glucoside (Prunin)	Flavanols	1.099	2.19231	kz0051	Camaldulenic acid	Terpenoids	1.077	0.13382

59			39		36			86	
kz0003			1.101		kz0009			1.078	
08	N-[(-)-Jasmonoyl]-(L)-Isoleucine (JA-L-Ile)	Organic acids	76	2.22345	92	Pinoresinol	Lignans	79	0.13110
kz0029			1.095		kz0022			1.078	
10	Parthenolide	Terpenoids	93	2.25247	19	Epipinoresinol	Lignans	33	0.13095
kz0009			1.093		kz0003			1.077	
08	Quercetin-3-O-robinobioside	Flavonols	27	2.25738	04	(±)-jasmonic acid	Organic acids	61	0.12608
kz0009			1.099		kz0010			1.079	
05	Kaempferol-3-O-rutinoside(Nicotiflorin)	Flavonols	86	2.27341	90	Methoxyursolic acid	Terpenoids	41	0.12368
kz0009			1.098		kz0040			1.079	
06	Kaempferol-3-O-robinobioside(Biorobin)	Flavonols	83	2.27477	13	Solatuberenol A	Others	80	0.11026
kz0046			1.104		kz0037			1.071	
32	isoceanothic acid	Terpenoids	83	2.27504	67	Obtusilin	Terpenoids	57	0.10413
kz0027			1.059		km081			1.074	
88	Sexangularetin 3-glucoside-7-rhamnoside	Flavonols	82	2.29944	8	Myricetin 3-O-rhamnoside (Myricitrin)	Flavonols	40	0.09480
kz0007			1.075		kz0000			1.078	
92	Apigenin 5-O-glucoside	Flavones	41	2.40388	52	Ethyl 3,4-Dihydroxybenzoate (Ethyl protocatechuate)	Phenolic acids	15	0.09287
kz0029			1.077		kz0003			1.079	
60	Eupatilin	Flavones	31	2.45423	08	N-[(-)-Jasmonoyl]-(L)-Isoleucine (JA-L-Ile)	Organic acids	10	0.08780
kz0038			1.091		kz0005			1.075	
62	3β-Hydroxy-28-norurs-17,19,21-trien	Terpenoids	52	2.46244	53	Neochlorogenic acid(5-O-Caffeoylquinic acid)	Phenolic acids	12	0.07352
kz0040			1.083		kz0037			1.073	
21	6-Deoxyfagomine	Alkaloids	77	2.49283	20	Maplexin H	Phenolic acids	39	0.07217
km087			1.079		kz0031			1.078	
9	Orotic acid	Vitamins	48	2.52743	47	Clemaphenol A	Lignans	42	0.06825
kz0009			1.088		kz0007			1.053	
12	Isorhamnetin-3-O-rutinoside (Narcissin)	Flavonols	45	2.56798	03	Delphinidin-3-O-glucoside (Mirtillin)	Anthocyanins	86	0.06728
kz0002			1.102		kz0038			1.070	
31	5-Aminolevulinate	Alkaloids	69	2.57224	21	Kampferol 3-O-(6”-galloyl)-β-D-galactopyranoside	Flavonols	31	0.05968
kz0042			1.103		kz0006			1.074	
33	Terminolic acid	Terpenoids	11	2.66464	13	1,2,3,6-Tetra-O-Galloyl-D-Glucose	Phenolic acids	03	0.05750
kz0047			1.087		kz0010			1.077	
56	N-Benzylmethylene isomethylamine	Alkaloids	88	2.66884	76	Ethyl gallate	Phenolic acids	89	0.05539
kz0005			1.105		kz0037			1.071	
84	5-O-p-Coumaroyl quinic acid O-hexoside	Phenolic acids	31	2.73214	19	Methyl 4,6-di-O-galloyl-D-glucoside	Phenolic acids	55	0.05475
kz0012			1.103		kz0053			1.076	
83	6-Aminocaproic acid	Organic acids	79	2.81845	89	Byzantionoside A	Others	86	0.05440
kz0029			1.097		kz0000			1.079	
62	Penduletin (5,4'-Dihydroxy-3,6,7-trimethoxyflavone)	Flavones	24	2.86255	55	3,4,5-Trimethoxycinnamic acid	Phenolic acids	89	0.04896
kz0042			1.091		km032			1.079	
63	Osthole	Coumarins	70	2.89337	9	3-(4-Hydroxyphenyl)propionic acid	Phenolic acids	87	0.04767
kz0000	3-O-p-Coumaroylshikimic acid	Phenolic acids	1.097	2.94171	kz0004	3-(4-Hydroxyphenyl)-propionic acid	Phenolic acids	1.077	0.04740

59			52	
kz0011			1.058	
03	6-Hydroxynicotinic acid	Alkaloids	06	2.95274
kz0007			1.096	
91	Apigenin 7-O-glucoside(Cosmosiin)	Flavones	05	3.08682
kz0038			1.104	
72	Lup-12-en-15α,19β-diol-3,11-dioxo-28-oic acid	Terpenoids	40	3.13494
kz0000			1.090	
64	1-O-Feruloyl quinic acid	Phenolic acids	24	3.20162
kz0042			1.099	
31	Isothankunic acid	Terpenoids	16	3.28889
kz0002			1.105	
32	O-Phosphocholine	Alkaloids	49	3.30817
km008			1.080	
0	Biochanin A	Isoflavones	37	3.32088
kz0011			1.070	
13	Lumichrome	Alkaloids	03	3.32532
kz0050			1.101	
43	Scopoletin?Beta-D-Glucuronide	Coumarins	29	3.32968
kz0011			1.097	
93	Prunetin	Isoflavones	55	3.68953
kz0026			1.104	
73	Eriodictyol-O-glucoside	Flavones	05	4.02963
kz0051			1.104	
87	Dunalianoside B	Phenolic acids	12	4.04620
kz0021			1.098	
97	2'-Hydoxy,5-methoxy Genistein-4',7-O-diglucoside	Isoflavones	45	4.18234
kz0035			1.101	
40	Dihydrokaempferol-7-O-glucoside	Flavonols	08	4.33561
kz0004			1.104	
40	Astilbin	Flavanols	27	4.36173
kz0004			1.105	
79	Cinnamic acid	Phenolic acids	09	4.75933
kz0031			1.073	
66	6-Hydroxy-7-methoxycoumarin	Coumarins	25	4.86834
kz0012			1.100	
61	γ-Aminobutyric acid	Organic acids	95	5.53141
kz0005			1.096	
48	Trans-3-O-p-coumaric quinic acid	Phenolic acids	30	6.45799
kz0029			1.085	
78	Pratensein 7-O-glucopyranoside	Flavones	37	7.47812
kz0022	Farrerol 7-O-glucoside	Flavanones	1.104	7.58551

95			81	
kz0046			1.080	
32	isoceanothic acid	Terpenoids	22	0.04736
kz0010			1.076	
74	Gallic acid	Phenolic acids	34	0.04474
kz0009			1.080	
79	Catechin-(7,8-bc)-4α-(3,4-dihydroxyphenyl)-dihydro-2-(3H)-pyranone	Flavanols	14	0.04322
kz0009			1.080	
78	Catechin-(7,8-bc)-4β-(3,4-dihydroxyphenyl)-dihydro-2-(3H)-pyranone	Flavanols	29	0.03990
km053			1.067	
2	Delphinidin 3-O-glucoside (Mirtillin)	Anthocyanins	90	0.03570
kz0012			1.078	
97	(Rs)-Mevalonic acid lithium salt	Organic acids	80	0.03552
kz0001			1.078	
56	7-O-Methyleriodictyol	Flavanols	70	0.03247
kz0012			1.079	
81	Citraconic acid	Organic acids	55	0.03035
kz0029			1.079	
62	Penduletin (5,4'-Dihydroxy-3,6,7-trimethoxyflavone)	Flavones	81	0.02909
kz0001			1.079	
18	Sakuranetin	Flavones	64	0.02817
km129			1.079	
3	Hesperetin O-malonylhexoside	Flavanones	79	0.02731
kz0038			1.079	
48	Cinchonain Ic	Flavanols	30	0.02639
kz0008			1.076	
30	Chrysoeriol-7-O-rutinoside	Flavones	88	0.02408
kz0009			1.079	
97	Terpineol mon-O-glucoside	Lignans	35	0.02299
kz0022			1.072	
21	Eucommin A	Lignans	55	0.02296
kz0001			1.078	
97	E-3,4,5'-Trihydroxy-3'-glucopyranosylstilbene	Others	64	0.02156
kz0029			1.079	
60	Eupatilin	Flavones	12	0.01979
kz0003			1.077	
06	Methyl jasmonate	Organic acids	58	0.01938
kz0050			1.079	
49	Salireposide	Phenolic acids	73	0.01919
kz0008			1.078	
31	Diosmin	Flavones	96	0.01520
kz0052	Neodiosmin (Diosmetin-7-O-Neohesperidoside)	Flavones	1.074	0.01459

02			34	
kz0003			1.105	
04	(±)-jasmonic acid	Organic acids	51	7.75797
kz0003			1.095	
06	Methyl jasmonate	Organic acids	81	8.51744
kz0005			1.103	
44	3-O-p-Coumaroyl quinic acid	Phenolic acids	76	9.88176
kz0001			1.104	
18	Sakuranetin	Flavones	00	10.11972
kz0001			1.103	
56	7-O-Methyleriodictyol	Flavanols	05	15.89286
kz0030			1.104	
83	5,2'-Dihydroxy-7,8-dimethoxyflavone glycosides	Flavones	38	29.80000
kz0037			1.105	
55	Quercetin-3-sambubioside	Flavonols	00	71.77500
kz0038			1.106	
33	Quercetin-O-pentosyl-O-rhamnoside-O-glucoside	Flavonols	07	72.52500
kz0041			1.102	
72	Quercetin 3-O-β-D-xylopyranosyl-(1→2)-β-D-galactopyranoside	Flavonols	91	97.07500
kz0012			1.100	214.7500
84	Oxaloacetic acid	Organic acids	96	0
kz0005			1.105	530.2500
80	1,6-di-O-Galloyl-β-D-Glucose	Phenolic acids	80	0
kz0045			1.106	541.5000
98	1,4-di-O-galloyl-β-D-glcose	Phenolic acids	10	0
kz0005			1.106	1082.500
51	methyl 6-O-galloyl-β-D-glucopyranoside	Phenolic acids	29	00
kz0028			1.106	1103.500
20	Limocitrin 7-glucoside	Flavonols	11	00
kz0036			1.106	2495.000
34	Sinapaldehyde Glucoside	Phenolic acids	37	00

26			07	
kz0042			1.079	
63	Osthole	Coumarins	77	0.01389
kz0038			1.077	
29	Quercetin 3-O-(6′′-galloyl)-β-D-galactopyranoside	Flavonols	03	0.01271
kz0016			1.080	
84	Tetramethylluteolin (3',4',5,7-Tetramethoxyflavone)	Flavones	43	0.01259
kz0017			1.071	
39	Quercetin-3-O-(2''-galloyl)-β-D-glucoside	Flavonols	21	0.01231
kz0029			1.076	
78	Pratensein 7-O-glucopyranoside	Flavones	25	0.01061
kz0019			1.080	
73	Kaempferol-3-O-(cinnamoyl)-sophoroside-7-O-glucose	Flavonols	19	0.01046
kz0022			1.080	
02	Farrerol 7-O-glucoside	Flavanones	22	0.00894
kz0021			1.080	
97	2'-Hydoxy,5-methoxy Genistein-4',7-O-diglucoside	Isoflavones	08	0.00768
kz0008			1.076	
86	Myricetin-3-O-arabinoside	Flavonols	60	0.00598
kz0026			1.080	
68	Ovalifoliolides B	Terpenoids	25	0.00569
kz0023			1.079	
37	Luteolin-7-O-β-D-gentiobioside	Flavones	58	0.00566
kz0016			1.080	
95	wogonoside	Flavones	31	0.00469
kz0035			1.077	
04	Geraniinic acid C	Phenolic acids	04	0.00446
kz0005			1.079	
75	3,5-Di-O-galloylshikimic acid	Phenolic acids	23	0.00414
kz0008			1.080	
41	Luteolin-O-sinapoylhexoside	Flavones	27	0.00409
kz0015			1.078	
84	Quercetin-3′,4′-dimethyl ether	Flavonols	79	0.00400
kz0050			1.080	
43	Scopoletin?Beta-D-Glucuronide	Coumarins	32	0.00398
kz0013			1.078	
07	Phthalic acid	Organic acids	19	0.00388
kz0029			1.080	
75	Acacetin-7-O-glucuronide	Flavones	19	0.00374
kz0017			1.077	
40	Quercetin-3-O-(2'',3''-digalloyl)-β-D-glucopyranoside	Flavonols	61	0.00350
kz0000	Caffeoylagmatine	Alkaloids	1.080	0.00329

32			39	
kz0038			1.080	
72	Lup-12-en-15α,19β-diol-3,11-dioxo-28-oic acid	Terpenoids	46	0.00321
kz0017			1.078	
50	Tercatain	Phenolic acids	40	0.00314
kz0031			1.079	
04	Nobotanin D	Phenolic acids	60	0.00309
kz0006			1.079	
12	1,3,4,6-Tetra-O-Galloyl-D-Glucose	Phenolic acids	91	0.00285
kz0045			1.077	
95	Geraniin	Phenolic acids	64	0.00247
kz0029			1.080	
81	Diosmetin-7-O-glucuronide	Flavones	19	0.00240
kz0017			1.080	
26	Prunellin A	Others	41	0.00236
kz0001			1.080	
86	2-Deoxyribose 1-phosphate	Others	29	0.00225
km130			1.080	
0	Hydroxy-methoxycinnamate	Phenolic acids	22	0.00210
kz0022			1.078	
12	Azalein (Azaleatin-3-O-rhamnoside)	Flavonols	85	0.00207
kz0045			1.079	
96	Methyl neochebulagate	Others	44	0.00204
kz0000			1.080	
64	1-O-Feruloyl quinic acid	Phenolic acids	04	0.00203
kz0005			1.080	
52	4-O-Caffeoyl quinic acid (criptochlorogenic acid)	Phenolic acids	04	0.00202
kz0000			1.080	
59	3-O-p-Coumaroylshikimic acid	Phenolic acids	33	0.00182
kz0037			1.080	
66	Uncargenin D	Terpenoids	16	0.00174
kz0046			1.080	
65	5,7,3',4',5’-pentahydroxydihydroflavone	Flavanones	33	0.00172
kz0010			1.080	
79	Galloyl Methyl gallate	Phenolic acids	17	0.00169
kz0002			1.079	
76	4-Aminoindole	Alkaloids	91	0.00136
kz0053			1.080	
76	5'-methoxyisolariciresinol-9'-O-β-D-xylopyranoside	Lignans	32	0.00135
kz0029			1.080	
83	Patuletin-3-O-β-D-glucopyranoside	Flavones	31	0.00125
kz0005	1-O-p-Coumaroyl quinic acid	Phenolic acids	1.080	0.00111

46			50	
kz0031<br>06	Cuspinin	Phenolic acids	1.080<br>08	0.00100
kz0018<br>24	cinnamoyltartaric acid	Phenolic acids	1.080<br>32	0.00099
kz0038<br>62	3β-Hydroxy-28-norurs-17,19,21-trien	Terpenoids	1.080<br>46	0.00094
kz0018<br>20	benzoylmalic acid	Phenolic acids	1.080<br>40	0.00078
kz0010<br>86	2,3-Dihydroxy 5(6),12(13)diene ursolic acid	Terpenoids	1.080<br>42	0.00072
kz0038<br>23	Kampferol 3-O-(2″-galloyl)-β-D-galactopyranoside	Flavonols	1.080<br>52	0.00054
kz0026<br>07	Lariciresinol glucopyranoside	Others	1.080<br>50	0.00053
kz0011<br>91	4-hydroxycoumarin di-glucoside	Coumarins	1.080<br>56	0.00048
kz0047<br>68	monogalloyl-diglucose	Phenolic acids	1.080<br>42	0.00039
kz0005<br>37	3-Galloylshikimic acid	Phenolic acids	1.080<br>41	0.00038
km064<br>0	Hesperetin 7-rutinoside (Hesperidin)	Flavanones	1.080<br>24	0.00033
kz0037<br>48	7'-O-Sinapoyljasminoside L	Terpenoids	1.080<br>36	0.00029
kz0037<br>16	Maplexin D (2,4-Di-O-Galloyl-1,5-Anhydro-D-Glucitol)	Phenolic acids	1.080<br>45	0.00020
kz0051<br>33	Luteolin-O-glucuronate-O-rhamnoside	Flavones	1.080<br>32	1340.0000<br>0
kz0009<br>19	Robinin(Kaempferol-3-O-gal-rham-7-O-rham)	Flavonols	1.080<br>54	1877.5000<br>0
kz0044<br>25	Luteolin-O-rutinoside-O-rhamnoside	Flavones	1.080<br>53	1907.5000<br>0
kz0045<br>98	1,4-di-O-galloyl-β-D-glcose	Phenolic acids	1.080<br>55	5277.5000<br>0
kz0005<br>80	1,6-Di-O-Galloyl-D-Glucose	Phenolic acids	1.080<br>50	5362.5000<br>0
kz0029<br>12	Santamarin	Terpenoids	1.080<br>47	5390.0000<br>0
kz0029<br>36	Silibinin	Flavanols	1.080<br>55	7915.0000<br>0
kz0000	1'-O-β-D-(3,4-Dihydroxyphenethyl)-O-caffeoyl-glucoside	Phenolic acids	1.080	9030.0000

Index	Compounds	Class	VIP	Fold_Change
78			53	0
kz004260	Corosolic acid	Terpenoids	1.08056	18800.00000
km0936	Quercetin 7-O-β-D-Glucuronide	Flavonols	1.08056	155750.00000

Table S2 Continued

RRT-F/RSL-F					RSS-F/RLM-F					RSS-F/RDP-F	
Index	Compounds	Class	VIP	Fold_Change	Index	Compounds	Class	VIP	Fold_Change	Index	Con
kz004698	7S,8R-threo-3′,9,9′-trihydroxy-3-methoxy-4′,7-epoxy-neolignan-4-O-α-L-rhamnopyranoside	Lignans	1.09	0.00001	kz004698	7S,8R-threo-3′,9,9′-trihydroxy-3-methoxy-4′,7-epoxy-neolignan-4-O-α-L-rhamnopyranoside	Lignans	1.08501	0.00006	kz005049	Sali
kz000530	Brevifolin carboxylic acid	Phenolic acids	1.09	0.00003	kz002211	Quercetin 3-O-β-D-xylopyranoside	Flavonols	1.08500	0.00006	kz004698	7S,8 rha
kz005049	Salireposide	Phenolic acids	1.09	0.00004	kz000881	Avicularin	Flavonols	1.08498	0.00007	kz003862	3β-
kz000703	Delphinidin-3-O-glucoside (Mirtillin)	Anthocyanins	1.09	0.00008	kz000530	Brevifolin carboxylic acid	Phenolic acids	1.08418	0.00021	kz003634	Sina
kz001332	Procyanidin B2	Proanthocyanidins	1.09	0.00008	kz000532	Protocatechuic acid-4-glucoside	Phenolic acids	1.08491	0.00036	kz002820	Lim
kz001335	Procyanidin B1	Proanthocyanidins	1.09	0.00010	kz003862	3β-Hydroxy-28-norurs-17,19,21-trien	Terpenoids	1.08488	0.00038	kz000551	Met
kz001333	Procyanidin B3	Proanthocyanidins	1.09	0.00011	kz005377	lyoniresinol-9'-O-β-D-xylopyranoside	Lignans	1.08500	0.00039	kz001086	2,3-
kz005377	lyoniresinol-9'-O-β-D-xylopyranoside	Lignans	1.09	0.00012	kz003634	Sinapaldehyde Glucoside	Phenolic acids	1.08499	0.00040	km0640	Hes
kz003717	Maplexin C (2,3-Di-O-Galloyl-1,5-Anhydro-D-Glucitol)	Phenolic acids	1.09	0.00016	kz000059	3-O-p-Coumaroylshikimic acid	Phenolic acids	1.08495	0.00062	kz002202	Farr
kz003716	Maplexin D (2,4-Di-O-Galloyl-1,5-Anhydro-D-Glucitol)	Phenolic acids	1.09	0.00020	kz000064	1-O-Feruloyl quinic acid	Phenolic acids	1.08492	0.00063	kz001177	Sco
kz000704	Cyanidin-O-syringic acid	Anthocyanins	1.09	0.00025	kz003766	Uncargenin D	Terpenoids	1.08498	0.00088	km0818	Myr
km0640	Hesperetin 7-rutinoside (Hesperidin)	Flavanones	1.09	0.00033	kz003717	Maplexin C (2,3-Di-O-Galloyl-1,5-Anhydro-D-Glucitol)	Phenolic acids	1.08463	0.00089	kz001824	cinr
kz004768	monogalloyl-diglucose	Phenolic acids	1.09	0.00039	kz002820	Limocitrin 7-glucoside	Flavonols	1.08479	0.00091	kz003794	Syri
km0818	Myricetin 3-O-rhamnoside (Myricitrin)	Flavonols	1.09	0.00044	kz001191	4-hydroxycoumarin di-glucoside	Coumarins	1.08495	0.00092	kz000306	Met
kz001191	4-hydroxycoumarin di-glucoside	Coumarins	1.09	0.00048	kz000551	Methyl 6-O-galloyl-β-D-glucopyranoside	Phenolic acids	1.08493	0.00092	kz002212	Aza
kz000553	Neochlorogenic acid(5-O-Caffeoylquinic acid)	Phenolic acids	1.09	0.00048	kz001086	2,3-Dihydroxy 5(6),12(13)diene ursolic acid	Terpenoids	1.08496	0.00101	kz000276	4-A
kz0031	Clemaphenol A	Lignans	1.0	0.00053	kz0038	Lup-12-en-15α,19β-diol-3,11-dioxo-28-oic acid	Terpenoids	1.085	0.00102	kz0017	Pru

47			9		72			00		26	
kz0038			1.0		km064			1.084		kz0053	
23	Kampferol 3-O-(2″-galloyl)-β-D-galactopyranoside	Flavonols	9	0.00054	0	Hesperetin 7-rutinoside (Hesperidin)	Flavanones	95	0.00110	76	5'-m
kz0005			1.0		kz0005			1.084		kz0041	
55	1-Caffeoylquinic acid	Phenolic acids	9	0.00061	46	1-O-p-Coumaroyl quinic acid	Phenolic acids	85	0.00110	72	Que
kz0038			1.0		kz0026			1.084		kz0003	
21	Kampferol 3-O-(6″-galloyl)-β-D-galactopyranoside	Flavonols	9	0.00061	07	Lariciresinol glucopyranoside	Others	81	0.00118	04	(±)-
kz0036			1.0		kz0022			1.084		kz0038	
36	Plantainoside A	Phenolic acids	9	0.00072	02	Farrerol 7-O-glucoside	Flavanones	99	0.00118	33	Que
kz0018			1.0		kz0050			1.084		kz0004	
20	benzoylmalic acid	Phenolic acids	9	0.00078	43	Scopoletin?Beta-D-Glucuronide	Coumarins	98	0.00120	79	Cinr
kz0038			1.0		kz0029			1.084		kz0052	
62	3β-Hydroxy-28-norurs-17,19,21-trien	Terpenoids	9	0.00094	78	Pratensein 7-O-glucopyranoside	Flavones	97	0.00142	26	Nec
kz0018			1.0		kz0001			1.084		kz0008	
24	cinnamoyltartaric acid	Phenolic acids	9	0.00099	86	2-Deoxyribose 1-phosphate	Others	93	0.00143	30	Chr
kz0031			1.0		kz0038			1.084		kz0008	
06	Cuspinin	Phenolic acids	9	0.00100	23	Kampferol 3-O-(2″-galloyl)-β-D-galactopyranoside	Flavonols	68	0.00150	31	Dios
kz0005			1.0		kz0037			1.084		kz0047	
46	1-O-p-Coumaroyl quinic acid	Phenolic acids	9	0.00111	16	Maplexin D (2,4-Di-O-Galloyl-1,5-Anhydro-D-Glucitol)	Phenolic acids	31	0.00156	74	(2R)
kz0029			1.0		kz0020			1.083		kz0003	
83	Patuletin-3-O-β-D-glucopyranoside	Flavones	9	0.00125	63	Galloyl-HHDP(Hexahydroxydiphenoyl)-glueopy ranose	Phenolic acids	39	0.00165	08	N-[(
kz0013		Proanthocyani	1.0		kz0018			1.084		kz0029	
40	Procyanidin C2	dins	9	0.00128	24	cinnamoyltartaric acid	Phenolic acids	90	0.00168	78	Prat
kz0047			1.0		kz0031			1.083		kz0011	
74	(2R)-Pinocembrin-7-neohesperidoside	Flavanones	9	0.00134	03	Gemin D	Phenolic acids	25	0.00170	91	4-hy
kz0053			1.0		kz0010			1.084		kz0042	
76	5'-methoxyisolariciresinol-9'-O-β-D-xylopyranoside	Lignans	9	0.00135	79	Galloyl Methyl gallate	Phenolic acids	99	0.00178	31	Isot
kz0002			1.0		kz0021			1.084		kz0042	
76	4-Aminoindole	Alkaloids	9	0.00136	97	2'-Hydoxy,5-methoxy Genistein-4',7-O-diglucoside	Isoflavones	84	0.00184	33	Terr
kz0025			1.0		kz0001			1.084		kz0038	
57	4-O-glucosyl-3,4-dihydroxybenzyl alcohol	Phenolic acids	9	0.00144	56	7-O-Methyleriodictyol	Flavanols	94	0.00204	72	Lup
kz0025			1.0		km130			1.084		kz0001	
51	Isosinapic acid-hexoside	Phenolic acids	9	0.00163	0	Hydroxy-methoxycinnamate	Phenolic acids	98	0.00213	56	7-O
kz0023			1.0		kz0003			1.084		kz0000	
67	Methyl dioxindole-3-acetate	Others	9	0.00166	06	Methyl jasmonate	Organic acids	99	0.00228	64	1-O
kz0046			1.0		kz0022			1.084		kz0050	
65	5,7,3',4',5’-pentahydroxydihydroflavone	Flavanones	9	0.00172	12	Azalein (Azaleatin-3-O-rhamnoside)	Flavonols	38	0.00261	43	Sco
kz0009			1.0		kz0037			1.084		kz0052	
74	Gallocatechin	Flavanols	9	0.00187	48	7'-O-Sinapoyljasminoside L	Terpenoids	70	0.00275	47	Lim
kz0033			1.0		kz0002			1.084		kz0046	
70	Swertiamarin	Terpenoids	9	0.00191	76	4-Aminoindole	Alkaloids	13	0.00276	32	isoc
kz0005	4-O-Caffeoyl quinic acid (criptochlorogenic acid)	Phenolic acids	1.0	0.00202	kz0001	Sakuranetin	Flavones	1.084	0.00278	kz0012	γ-Ar

52			9		18			99		61	
kz0045			1.0		kz0006			1.084		kz0031	
96	Methyl neochebulagate	Others	9	0.00204	12	1,3,4,6-Tetra-O-Galloyl-D-Glucose	Phenolic acids	12	0.00321	66	6-H
kz0022			1.0		kz0017			1.084		kz0012	
12	Azalein (Azaleatin-3-O-rhamnoside)	Flavonols	9	0.00207	26	Prunellin A	Others	12	0.00370	97	(Rs)
kz0037			1.0		kz0005			1.084		kz0013	
94	Syringic Aldehyde-glucoside	Phenolic acids	9	0.00208	36	5-Galloylshikimic acid	Phenolic acids	84	0.00372	18	Qui
kz0000			1.0		kz0042			1.084		kz0029	
50	Homogentisic acid	Phenolic acids	9	0.00214	63	Osthole	Coumarins	88	0.00480	10	Part
kz0001			1.0		kz0026			1.084		kz0000	
86	2-Deoxyribose 1-phosphate	Others	9	0.00225	68	Ovalifoliolides B	Terpenoids	94	0.00597	59	3-O
kz0017			1.0		kz0053			1.083		kz0026	
26	Prunellin A	Others	9	0.00236	76	5'-methoxyisolariciresinol-9'-O-β-D-xylopyranoside	Lignans	83	0.00624	68	Ova
kz0029			1.0		kz0017			1.082		kz0042	
81	Diosmetin-7-O-glucuronide	Flavones	9	0.00240	40	Quercetin-3-O-(2'',3''-digalloyl)-β-D-glucopyranoside	Flavonols	97	0.00730	63	Ost
kz0005			1.0		kz0046			1.083		kz0012	
92	Rosmarinyl Glucoside	Phenolic acids	9	0.00267	65	5,7,3',4',5′-pentahydroxydihydroflavone	Flavanones	98	0.00760	81	Citr
kz0006			1.0		kz0005			1.076		kz0002	
12	1,3,4,6-Tetra-O-Galloyl-D-Glucose	Phenolic acids	9	0.00285	52	4-O-Caffeoyl quinic acid (criptochlorogenic acid)	Phenolic acids	88	0.00774	32	O-P
kz0031			1.0		kz0031			1.083		kz0037	
04	Nobotanin D	Phenolic acids	9	0.00309	06	Cuspinin	Phenolic acids	92	0.00787	19	Met
kz0013		Proanthocyani	1.0		kz0029			1.084		kz0053	
31	Procyanidin A1	dins	9	0.00311	60	Eupatilin	Flavones	90	0.00806	77	lyon
kz0009			1.0		kz0000			1.083		kz0001	
73	Epigallocatechin (EGC)	Flavanols	9	0.00312	32	Caffeoylagmatine	Alkaloids	56	0.00833	18	Sak
kz0017			1.0		kz0029			1.084		kz0030	
50	Tercatain	Phenolic acids	9	0.00314	62	Penduletin (5,4'-Dihydroxy-3,6,7-trimethoxyflavone)	Flavones	99	0.01016	99	Di-C
kz0000			1.0		kz0023			1.077		kz0024	
32	Caffeoylagmatine	Alkaloids	9	0.00329	37	Luteolin-7-O-β-D-gentiobioside	Flavones	24	0.01253	19	Ergo
kz0029			1.0		kz0005			1.084		kz0002	
75	Acacetin-7-O-glucuronide	Flavones	9	0.00374	37	3-Galloylshikimic acid	Phenolic acids	09	0.01461	74	N-si
kz0015			1.0		kz0015			1.083		kz0001	
84	Quercetin-3′,4′-dimethyl ether	Flavonols	9	0.00400	84	Quercetin-3′,4′-dimethyl ether	Flavonols	30	0.01563	45	Forr
kz0005			1.0		kz0016			1.084		kz0040	
75	3,5-Di-O-galloylshikimic acid	Phenolic acids	9	0.00414	95	wogonoside	Flavones	56	0.01604	13	Sola
kz0047			1.0		kz0003			1.084		kz0038	
03	secoisolariciresinol 9-O-β-D-glucopyranoside	Lignans	9	0.00456	04	(±)-jasmonic acid	Organic acids	48	0.01625	54	Ros
kz0016			1.0		kz0046			1.084		kz0047	2-H
95	wogonoside	Flavones	9	0.00469	32	isoceanothic acid	Terpenoids	91	0.02082	62	(trir
kz0026			1.0		kz0008			1.084		kz0025	
68	Ovalifoliolides B	Terpenoids	9	0.00569	41	Luteolin-O-sinapoylhexoside	Flavones	66	0.02427	51	Isos
kz0038	Sieboldin	Chalcones	1.0	0.00802	kz0019	Kaempferol-3-O-(cinnamoyl)-sophoroside-7-O-glucose	Flavonols	1.084	0.02644	kz0010	4-M

09			9		73			12		20	
kz0009			1.0		kz0050			1.083		kz0010	
71	Catechin	Flavanols	9	0.00859	49	Salireposide	Phenolic acids	94	0.02783	93	Ole
kz0022			1.0		kz0001			1.078		kz0009	
02	Farrerol 7-O-glucoside	Flavanones	9	0.00894	97	E-3,4,5'-Trihydroxy-3'-glucopyranosylstilbene	Others	77	0.02899	78	Cat
kz0038			1.0		kz0008			1.083		kz0010	
48	Cinchonain Ic	Flavanols	9	0.00946	31	Diosmin	Flavones	19	0.03183	91	Eus
kz0047			1.0		kz0052			1.075		kz0009	
71	Epicatechin glucoside	Flavanols	9	0.01045	26	Neodiosmin (Diosmetin-7-O-Neohesperidoside)	Flavones	86	0.03236	79	Cat
kz0029			1.0		kz0030			1.083		kz0038	
78	Pratensein 7-O-glucopyranoside	Flavones	9	0.01061	83	5,2'-Dihydroxy-7,8-dimethoxyflavone glycosides	Flavones	32	0.03356	63	San
kz0004			1.0		kz0008			1.080		kz0004	
19	Phlorizin	Chalcones	9	0.01107	30	Chrysoeriol-7-O-rutinoside	Flavones	56	0.03441	14	2-Pi
kz0009			1.0		kz0005			1.082		kz0009	
79	Catechin-(7,8-bc)-4α-(3,4-dihydroxyphenyl)-dihydro-2-(3H)-pyranone	Flavanols	9	0.01247	44	3-O-p-Coumaroyl quinic acid	Phenolic acids	43	0.03590	11	Que
kz0016			1.0		kz0017			1.069		kz0005	
84	Tetramethylluteolin (3',4',5,7-Tetramethoxyflavone)	Flavones	9	0.01259	39	Quercetin-3-O-(2''-galloyl)-β-D-glucoside	Flavonols	17	0.03650	22	Met
kz0009			1.0		kz0038			1.078		kz0005	
78	Catechin-(7,8-bc)-4β-(3,4-dihydroxyphenyl)-dihydro-2-(3H)-pyranone	Flavanols	9	0.01272	29	Quercetin 3-O-(6’’-galloyl)-β-D-galactopyranoside	Flavonols	97	0.03681	39	3-H
kz0052			1.0		kz0003			1.084		kz0009	
26	Neodiosmin (Diosmetin-7-O-Neohesperidoside)	Flavones	9	0.01281	08	N-[(-)-Jasmonoyl]-(L)-Isoleucine (JA-L-Ile)	Organic acids	55	0.03949	08	Que
kz0009			1.0		kz0005			1.083		kz0005	
69	Afzelechin(3,5,7,4'-Tetrahydroxyflavan)	Flavanols	9	0.01380	48	Trans-3-O-p-coumaric quinic acid	Phenolic acids	67	0.03958	67	Dita
kz0008			1.0		kz0042			1.083		kz0016	
31	Diosmin	Flavones	9	0.01422	31	Isothankunic acid	Terpenoids	60	0.05135	95	wog
km043			1.0		kz0009			1.083		kz0034	
6	Afzelechin (3,5,7,4'-Tetrahydroxyflavan)	Flavanones	9	0.01662	97	Terpineol monO-glucoside	Lignans	44	0.05259	98	2-O
kz0003			1.0		kz0037			1.078		kz0050	
06	Methyl jasmonate	Organic acids	9	0.01938	67	Obtusilin	Terpenoids	29	0.05416	54	Chr
kz0029			1.0		kz0004			1.082		kz0013	
60	Eupatilin	Flavones	9	0.01979	40	Astilbin	Flavanols	68	0.06103	12	Shik
kz0038			1.0		kz0010			1.084		kz0003	
29	Quercetin 3-O-(6’’-galloyl)-β-D-galactopyranoside	Flavonols	9	0.02233	90	Methoxyursolic acid	Terpenoids	55	0.06384	12	A-K
kz0017			1.0		kz0051			1.083		kz0005	
39	Quercetin-3-O-(2''-galloyl)-β-D-glucoside	Flavonols	8	0.02245	36	Camaldulenic acid	Terpenoids	92	0.06751	42	1-O
kz0037			1.0		kz0022			1.068		kz0001	
19	Methyl 4,6-di-O-galloyl-D-glucoside	Phenolic acids	8	0.02424	21	Eucommin A	Lignans	60	0.07532	21	Chr
kz0000			1.0		kz0004			1.084		kz0010	
40	N,N'-Bis(Sinapoyl)Spermidine	Alkaloids	9	0.02434	79	Cinnamic acid	Phenolic acids	56	0.07777	41	N-A
kz0008			1.0		kz0051			1.084		kz0005	
30	Chrysoeriol-7-O-rutinoside	Flavones	9	0.02440	87	Dunalianoside B	Phenolic acids	06	0.07983	20	Sina
kz0037	7'-O-Sinapoyljasminoside L	Terpenoids	1.0	0.02606	kz0035	Dihydrokaempferol-7-O-glucoside	Flavonols	1.083	0.08126	kz0004	But

48			9		40			68		22	
kz0038			1.0		kz0038			1.082		kz0004	
12	Isorhamnetin O-malonylglucoside	Flavonols	9	0.02628	48	Cinchonain Ic	Flavanols	88	0.08136	34	Pinc
kz0053			1.0		km129			1.083		km084	
89	Byzantionoside A	Others	9	0.02692	3	Hesperetin O-malonylhexoside	Flavanones	13	0.08775	9	Nar
kz0029			1.0		kz0026			1.084		kz0011	
62	Penduletin (5,4'-Dihydroxy-3,6,7-trimethoxyflavone)	Flavones	9	0.02909	73	Eriodictyol-O-glucoside	Flavones	32	0.08934	03	6-H
kz0001			1.0		kz0046			1.050		kz0051	
56	7-O-Methyleriodictyol	Flavanols	9	0.03247	30	2α-hydroxyursolic acid	Terpenoids	91	0.09062	36	Can
kz0042			1.0		kz0012			1.082		kz0046	
31	Isothankunic acid	Terpenoids	9	0.03259	97	(Rs)-Mevalonic acid	Organic acids	07	0.09556	97	Taxi
kz0005			1.0		kz0001			1.083		kz0010	
36	5-Galloylshikimic acid	Phenolic acids	9	0.03507	45	Formononetin	Isoflavones	97	0.09586	32	N-A
kz0005			1.0		kz0009			1.084		kz0038	
37	3-Galloylshikimic acid	Phenolic acids	9	0.04409	78	Catechin-(7,8-bc)-4β-(3,4-dihydroxyphenyl)-dihydro-2-(3H)-pyranone	Flavanols	93	0.10283	12	Isor
kz0035			1.0		kz0009			1.084		kz0008	
19	Cimidahurinine	Phenolic acids	9	0.04974	79	Catechin-(7,8-bc)-4α-(3,4-dihydroxyphenyl)-dihydro-2-(3H)-pyranone	Flavanols	39	0.10763	89	Spir
kz0047			1.0		kz0042			1.082		kz0008	
38	4,7,9,9′-Tetrahydroxy-3,3′-dimethoxy-8-O-4′-neolignan	Others	9	0.05055	33	Terminolic acid	Terpenoids	17	0.10813	88	Que
kz0000			1.0		kz0012			1.083		kz0005	
58	Protocatechuic acid O-glucoside	Phenolic acids	9	0.05189	81	Citraconic acid	Organic acids	12	0.10841	84	5-O
kz0010			1.0		kz0012			1.080		km084	
74	Gallic acid	Phenolic acids	9	0.05321	87	(S)-(-)-2-Hydroxyisocaproic acid	Organic acids	20	0.12064	3	Nar
km053			1.0		kz0010			1.081		kz0005	
2	Delphinidin 3-O-glucoside (Mirtillin)	Anthocyanins	7	0.05463	93	Oleanolic acid 2-O-β-D-glucopyranoside	Terpenoids	99	0.12643	14	3,4-
kz0030			1.0		kz0047	2-Hydroxy-5,8,11,14,17-icosapentaenoyloxy]propyl-2-		1.084		kz0030	
99	Di-O-Glucose-quinic acid	Phenolic acids	9	0.05597	62	(trimethylammonio)ethyl phosphate	Alkaloids	27	0.12904	38	3-O
kz0029			1.0		kz0037			1.072		kz0023	
10	Parthenolide	Terpenoids	9	0.06178	20	Maplexin H	Phenolic acids	95	0.14063	67	Met
kz0035			1.0		kz0011			1.084		kz0038	
18	5-(2-Hydroxyethyl)-2-O-glucosylohenol	Phenolic acids	9	0.06180	01	Trigonelline	Alkaloids	35	0.14334	11	Kae
kz0010			1.0		kz0012			1.081		kz0008	
86	2,3-Dihydroxy 5(6),12(13)diene ursolic acid	Terpenoids	9	0.07097	61	γ-Aminobutyric acid	Organic acids	15	0.14469	00	Chr
km111			1.0		kz0004			1.083		km130	
3	5-O-p-Coumaroyl shikimic acid	Phenolic acids	8	0.07658	89	Protocatechuic acid	Phenolic acids	93	0.16372	0	Hyd
kz0015			1.0		kz0005			1.082		kz0004	
81	Calceorioside B	Others	8	0.08463	54	Chlorogenic acid	Phenolic acids	81	0.16377	41	Hes
kz0053			1.0		kz0013			1.083		kz0047	
73	Lyoniresinol	Lignans	8	0.08532	02	2,3-Dihydroxybenzoic Acid	Organic acids	85	0.16790	35	Lign
kz0050			1.0		kz0038			1.064		kz0013	
88	Tricetin-4'-methyl-ether-3'-O-β-D-glucoside	Flavonols	9	0.08551	21	Kampferol 3-O-(6″-galloyl)-β-D-galactopyranoside	Flavonols	30	0.16836	32	Proc
kz0053	(+)-Isolariciresinol	Lignans	1.0	0.08581	kz0029	Parthenolide	Terpenoids	1.083	0.17233	kz0031	mly

70			9		10			97		08	
kz0004			1.0		kz0002			1.082		kz0053	
40	Astilbin	Flavanols	9	0.08687	94	2-Hydroxyisocaproic acid	Organic acids	69	0.17338	85	Mac
kz0011			1.0		kz0004			1.076		km022	
00	Nicotinic Acid Methyl Ester(Methyl Nicotinate)	Alkaloids	9	0.08790	95	3-(4-Hydroxyphenyl)-propionic acid	Phenolic acids	23	0.17585	8	Que
kz0041			1.0		kz0040			1.073		kz0022	
70	Rhamnetin 3-O-β-D-Glucoside	Flavonols	9	0.09070	13	Solatuberenol A	Others	22	0.17597	14	isoh
km130			1.0		km032			1.079		kz0008	
7	Isorhamnetin 5-O-hexoside	Flavonols	9	0.09278	9	3-(4-Hydroxyphenyl)propionic acid	Phenolic acids	89	0.17687	92	6-H
kz0041			1.0		kz0004			1.084		km007	
69	Isorhamnetin 3-O-β-D-Glucoside	Flavonols	9	0.09292	87	2,5-Dihydroxybenzoic acid	Phenolic acids	03	0.17787	1	Que
kz0001			1.0		kz0053			1.069		kz0005	
47	Methylquercetin O-hexoside	Flavonols	9	0.09517	89	Byzantionoside A	Others	58	0.17989	92	Ros
kz0028			1.0		kz0005			1.071		kz0013	
29	Isorhamnetin-7-O-glucoside	Flavonols	9	0.09566	53	Neochlorogenic acid(5-O-Caffeoylquinic acid)	Phenolic acids	73	0.18052	35	Proc
kz0037			1.0		kz0041			1.082		kz0013	
14	6-O-Galloylglucose	Phenolic acids	9	0.09570	33	L-Ascorbic acid	Vitamins	44	0.19856	33	Proc
kz0034			1.0		kz0038			1.083		kz0004	
97	3-O-Galloyl-β-D-glucose	Phenolic acids	9	0.09722	63	Sanguisorbigenin	Terpenoids	13	0.20173	77	Tyro
kz0029			1.0		kz0026			1.040		kz0010	
82	6-methoxykaempferol-3-O-glucoside	Flavonols	9	0.09795	65	Betulinic acid	Terpenoids	31	0.20622	90	Met
kz0041			1.0		kz0047			1.084		kz0006	
74	Benzyl-O-β-D-glucopyranose-β-D-xyranoside	Others	2	0.09931	74	(2R)-Pinocembrin-7-neohesperidoside	Flavanones	07	0.21239	98	Cya
kz0031			1.0		kz0006			1.055		kz0041	
00	5-O-Galloylhamamelose	Phenolic acids	9	0.10011	13	1,2,3,6-Tetra-O-Galloyl-D-Glucose	Phenolic acids	87	0.22107	74	Ben
kz0005			1.0		kz0038			1.074		kz0011	
77	Hexahydroxydiphenoylglucose	Phenolic acids	9	0.10074	60	Caffeoyl hawthorn acid	Terpenoids	95	0.22572	75	Escu
kz0052			1.0		kz0031			1.062		kz0016	
11	5,7,8,4'-Tetramethoxyflavone	Flavones	8	0.10299	16	2,4,6-trihydroxy benzoic acid	Organic acids	99	0.22793	71	5,7,
kz0005			1.0		kz0038			1.079		kz0005	
96	β-D-Furanofructosyl-α-D-(6-mustard acyl)glucoside	Phenolic acids	9	0.10548	54	Roseoside	Others	76	0.24325	04	Caff
kz0008			1.0		kz0000			1.076		kz0041	
41	Luteolin-O-sinapoylhexoside	Flavones	8	0.11433	70	Anthranilate O-hexosyl-O-hexoside	Phenolic acids	07	0.25133	69	Isor
kz0005			1.0		kz0017			1.077		kz0006	
04	Caffeic acid	Phenolic acids	9	0.11486	06	Isololiolide	Others	11	0.25276	12	1,3,
kz0008			1.0		kz0010			1.084		kz0037	
81	Avicularin	Flavonols	9	0.11730	91	Euscaphic acid	Terpenoids	49	0.25788	20	Map
kz0009			1.0		kz0011			1.084		kz0002	
23	6-Hydroxykaempferol-3,6-O-Diglucoside-7-O-Glucuronic Acid	Flavonols	7	0.11925	54	Nicotinamide	Vitamins	30	0.26314	90	Ami
kz0004			1.0		kz0030			1.082		kz0004	
24	Eriodictyol	Flavanones	9	0.12258	38	3-O-(2-O-Acetyl-β-D-glucopyranosyl) oleanolic acid	Terpenoids	26	0.26364	91	p-Co
kz0026	Eriodictyol-O-glucoside	Flavones	1.0	0.12993	kz0004	Sweroside	Terpenoids	1.074	0.26514	kz0010	Cho

73			9	
km054			1.0	
8	Dihydroquercetin (Taxifolin)	Flavonols	9	0.13011
kz0051			1.0	
87	Dunalianoside B	Phenolic acids	9	0.13376
kz0040			1.0	
13	Solatuberenol A	Others	8	0.13542
kz0031			1.0	
09	Mearnsitrin	Flavones	9	0.13568
kz0035			1.0	
40	Dihydrokaempferol-7-O-glucoside	Flavonols	9	0.13591
kz0003			1.0	
08	N-[(-)-Jasmonoyl]-(L)-Isoleucine (JA-L-Ile)	Organic acids	9	0.13629
kz0010			1.0	
20	4-Methyl-5-thiazoleethanol	Others	9	0.14018
kz0010			1.0	
76	Ethyl gallate	Phenolic acids	9	0.14267
kz0047			1.0	
35	Ligraminol E	Others	8	0.14464
kz0022			1.0	
11	Quercetin 3-O-β-D-xylopyranoside	Flavonols	9	0.14493
kz0006			1.0	
13	1,2,3,6-Tetra-O-Galloyl-D-Glucose	Phenolic acids	8	0.15028
kz0003			1.0	
04	(±)-jasmonic acid	Organic acids	9	0.15322
kz0026			1.0	
07	Lariciresinol glucopyranoside	Others	9	0.15396
kz0038			1.0	
54	Roseoside	Others	9	0.15467
kz0009			1.0	
97	Terpineol monO-glucoside	Lignans	9	0.15469
kz0011			1.0	
77	Scoparone	Coumarins	9	0.15969
km140			1.0	
5	Nicotinic acid-hexoside	Vitamins	8	0.16522
kz0013			1.0	
12	Shikimic acid	Organic acids	9	0.16935
kz0012			1.0	
97	(Rs)-Mevalonic acid lithium salt	Organic acids	9	0.16956
kz0040			1.0	
15	Cis-p-coumaric acid 4-O-glucoside	Others	9	0.18049
kz0029	Diosmetin-7-O-galactoside	Flavones	1.0	0.18110

10			52	
kz0012			1.081	
79	L-Homoserine	Organic acids	19	0.27305
kz0011			1.067	
77	Scoparone	Coumarins	71	0.27803
kz0012			1.080	
98	L-(+)-Tartaric acid	Organic acids	33	0.30047
kz0005			1.071	
39	3-Hydroxy-4-isopropylbenzylalcohol 3-glucoside	Phenolic acids	28	0.31549
kz0012			1.069	
89	Malic acid	Organic acids	72	0.31886
kz0011			1.033	
03	6-Hydroxynicotinic acid	Alkaloids	20	0.32235
kz0028			1.081	
53	Dihydroisopelletierine	Alkaloids	68	0.32303
kz0012			1.084	
83	6-Aminocaproic acid	Organic acids	03	0.32788
kz0037			1.018	
19	Methyl 4,6-di-O-galloyl-D-glucoside	Phenolic acids	06	0.32836
kz0010			1.073	
97	Betaine	Alkaloids	35	0.32968
km081			1.060	
8	Myricetin 3-O-rhamnoside (Myricitrin)	Flavonols	30	0.33372
km008			1.079	
0	Biochanin A	Isoflavones	94	0.33640
kz0005			1.035	
84	5-O-p-Coumaroyl quinic acid O-hexoside	Phenolic acids	96	0.34771
kz0013		Proanthocyani	1.014	
31	Procyanidin A1	dins	27	0.34892
kz0011			1.077	
93	Prunetin	Isoflavones	32	0.36155
kz0012			1.081	
68	2-Furanoic acid	Organic acids	47	0.37093
kz0046			1.076	
36	2α-hydroxypyracrenic acid	Terpenoids	10	0.38159
kz0038			1.028	
61	3-O-Trans-feruloyl euscaphic acid	Terpenoids	30	0.38317
km087			1.064	
9	Orotic acid	Vitamins	22	0.38500
kz0022			1.073	
20	Olivil-4′-O-β-D-glucoside	Lignans	53	0.38927
kz0010	D-Glucurono-6,3-lactone	Others	1.055	0.39459

96	
kz0012	
89	Mal
kz0025	
56	4-O
kz0033	
70	Swe
kz0015	
81	Calc
kz0012	
75	Met
kz0028	
29	Isor
kz0047	
56	N-B
kz0000	
51	4-M
kz0010	
79	Gall
kz0012	
76	Suc
kz0050	
88	Tric
kz0007	
79	Dios
kz0041	
70	Rha
kz0008	
84	Kae
km023	
9	Chr
kz0001	
47	Met
km043	
6	Afze
kz0025	
54	p-H
kz0000	
49	2-(F
kz0038	
60	Caff
kz0028	Dim

77			7		25			75		07	
kz0004			1.0		kz0015			1.079		kz0002	
17	Phloretin	Chalcones	8	0.18178	98	Stachydrine	Alkaloids	90	0.39570	59	Thia
kz0026			1.0		kz0052			1.075		kz0005	
87	Nortrachelogenin 4-O-β-D-glucoside	Lignans	7	0.18286	47	Limonin	Others	14	0.39819	57	Glu
kz0025			1.0		kz0037			1.023		kz0002	
48	p-Coumaric acid-O-glycoside	Phenolic acids	9	0.18647	18	2,3-Di-O-Galloyl-D-Glucose	Phenolic acids	88	0.40206	01	D-ga
kz0037			1.0		kz0002			1.079		kz0046	
20	Maplexin H	Phenolic acids	8	0.19104	31	5-Aminolevulinate	Alkaloids	40	0.40391	78	Ann
kz0024			1.0		kz0010			1.082		kz0029	
86	1,4,8-trihydroxy naphthalene-1-O-β-D-glucopyranoside	Quinones	9	0.19536	88	3,24-Dihydroxy-17,21-semiacetal-12(13)oleanolic fruit	Terpenoids	74	0.40587	82	6-m
kz0000			1.0		kz0002			1.080		kz0005	
59	3-O-p-Coumaroylshikimic acid	Phenolic acids	7	0.19613	32	O-Phosphocholine	Alkaloids	97	0.40975	12	Syri
kz0038			1.0		kz0047			1.075		kz0011	
61	3-O-Trans-feruloyl euscaphic acid	Terpenoids	8	0.19874	56	N-Benzylmethylene isomethylamine	Alkaloids	90	0.41132	05	Ace
kz0004			1.0		kz0010			1.083		kz0025	
31	Eriodictyol 7-O-glucoside	Flavanones	7	0.19903	87	Maslinic acid	Terpenoids	09	0.41353	46	Isos
kz0005			1.0		kz0046			1.081		kz0035	
54	Chlorogenic acid	Phenolic acids	9	0.20145	27	alphitolic acid	Terpenoids	39	0.41686	17	Glu
kz0005			1.0		kz0035			1.081		kz0022	
22	Methyl sinapate	Phenolic acids	6	0.20451	49	Hederagenin	Others	83	0.41845	19	Epi
kz0012			1.0		kz0010			1.082		kz0053	
81	Citraconic acid	Organic acids	9	0.22024	89	2-Hydroxyoleanolic acid	Terpenoids	24	0.41881	55	Koa
kz0031			1.0		kz0040			1.059		kz0010	
03	Gemin D	Phenolic acids	9	0.22244	21	6-Deoxyfagomine	Alkaloids	74	0.42253	97	Beta
kz0029			1.0		kz0046			1.082		kz0009	
95	Quercetin-7-O-(6'-O-malonyl)-β-D-glucoside	Flavonols	7	0.23150	26	pomolic acid	Terpenoids	94	0.42260	69	Afze
km121			1.0		kz0000			1.017		kz0005	
1	Chrysoeriol 7-O-hexoside	Flavones	4	0.23276	50	Homogentisic acid	Phenolic acids	86	0.48112	37	3-G
kz0020			1.0		kz0026			1.018		kz0002	
63	Galloyl-HHDP(Hexahydroxydiphenoyl)-glueopy ranose	Phenolic acids	8	0.24006	87	Nortrachelogenin 4-O-β-D-glucoside	Lignans	70	0.48887	58	Nico
kz0051			1.0		kz0016			1.046		kz0050	
86	Dunalianoside C	Phenolic acids	8	0.25114	42	5'-Glucopyranosyloxyjasmanic acid	Phenolic acids	87	0.48946	84	(Kae
kz0000			1.0		kz0002			1.083		kz0009	
52	Ethyl 3,4-Dihydroxybenzoate (Ethyl protocatechuate)	Phenolic acids	8	0.25241	01	D-galacitol	Others	76	0.49021	92	Pino
kz0029			1.0		kz0002			1.072		km081	
49	Hispidulin	Flavones	7	0.25402	40	Trans-Zeatin-9-N-Glucoside	Alkaloids	77	2.01911	4	Mor
kz0009			1.0		kz0005			1.077		kz0020	
95	Syringaresinol	Lignans	9	0.25586	01	Methyl p-coumarate	Phenolic acids	76	2.05142	08	3,5,
km068			1.0		km023			1.047		kz0029	
6	Kaempferol 3-O-rhamnoside (Kaempferin)	Flavonols	9	0.25778	5	Apigenin	Flavones	22	2.09381	49	Hisp
kz0028	Dihydroisopelletierine	Alkaloids	1.0	0.25997	kz0007	Luteolin-7-O-glucoside(Cynaroside)	Flavones	1.073	2.10816	kz0005	Met

53			9		95			71		01	
kz0013			1.0		kz0003			1.071		km130	
18	Quinic Acid	Organic acids	9	0.26365	12	A-Ketoglutaric acid	Organic acids	71	2.17849	7	Isor
kz0002			1.0		kz0005			1.080		kz0038	
84	3-Hydroxypropanoic acid	Organic acids	8	0.26557	14	3,4-Dimethoxycinnamic acid	Phenolic acids	04	2.21638	23	Kam
kz0026			1.0		kz0005			1.075		km068	
78	Quercetin-3-O-(6''-O-acetyl)-galactoside	Flavonols	8	0.26571	11	Ferulic acid	Phenolic acids	12	2.24935	3	Kae
km023			1.0		kz0050			1.063		kz0005	
9	Chrysoeriol	Flavones	7	0.26644	79	Kaempferol-4'-O-β-D-glucopyranoside	Flavonols	03	2.25118	36	5-G
kz0005			1.0		kz0028			1.078		kz0008	
60	Trihydroxycinnamoylquinic acid	Phenolic acids	8	0.29274	62	Dihydrosedinine	Alkaloids	85	2.29400	66	Que
kz0016			1.0		km043			1.083		kz0006	
71	5,7,2'-Trhiyroxy-8-methoxyflavone	Flavones	8	0.30671	6	Afzelechin (3,5,7,4'-Tetrahydroxyflavan)	Flavanones	69	2.31188	13	1,2,
kz0009			1.0		kz0038			1.054		kz0012	
92	Pinoresinol	Lignans	9	0.31102	11	Kaempferol-malonyl-3-O-glucoside	Flavonols	93	2.36829	85	2-M
kz0022			1.0		kz0038			1.079		kz0046	
21	Eucommin A	Lignans	8	0.31565	49	dihydrodehydrodiconiferyl alcohol 4-O-β-D-glucopyranosides	Lignans	02	2.41183	36	2α-
km129			1.0		kz0021			1.007		kz0050	
3	Hesperetin O-malonylhexoside	Flavanones	9	0.31611	64	Isoluteolin (Orobol)(5,7,3',4'-tetrahydroxyisoflavone)	Isoflavones	26	2.44226	78	Kae
kz0007			1.0		kz0005			1.073		kz0034	
79	Diosmetin	Flavones	8	0.31841	12	Syringic acid	Phenolic acids	29	2.44294	97	3-O
kz0038			1.0		kz0010			1.081		kz0031	
11	Kaempferol-malonyl-3-O-glucoside	Flavonols	5	0.32473	96	Choline	Alkaloids	12	2.47368	00	5-O
kz0022			1.0		kz0000			1.082		kz0031	
19	(+)-Epipinoresinol	Lignans	9	0.32698	53	Isoferulic Acid	Phenolic acids	17	2.55076	09	Mea
kz0000			1.0		kz0000			1.082		kz0037	
68	Catechin gallate	Phenolic acids	6	0.33213	56	Salicylic acid glucoside	Phenolic acids	65	2.55197	14	6-O
km007			1.0		kz0009			1.082		kz0005	
1	Quercetin 3-O-galactoside (Hyperin)	Flavonols	9	0.33575	69	Afzelechin(3,5,7,4'-Tetrahydroxyflavan)	Flavanols	90	2.56019	32	Prot
kz0022			1.0		km057			1.015		kz0022	
14	isohyperoside	Flavonols	9	0.33900	3	Epicatechin gallate (ECG)	Flavanols	52	2.58712	20	Oliv
kz0030			1.0		kz0053			1.081		kz0009	
98	3-O-Digalloyl quinic acid	Phenolic acids	6	0.34151	75	Isolariciresinol 9'-O-Glucoside	Lignans	58	2.59903	71	Cat
kz0008			1.0		kz0009			1.077		km077	
92	6-Hydroxykaempferol-7-O-glucoside	Flavonols	9	0.34564	15	6-Hydroxykaempferol-7,6-O-Diglucoside	Flavonols	60	2.60181	9	Lut
kz0005			1.0		kz0050			1.067		kz0029	
73	p-Coumaroylferuloyltartaric acid	Phenolic acids	9	0.35000	46	Dihydrokaempferol-3-O-β-D-glucoside	Flavanols	84	2.61257	95	Que
kz0004			1.0		kz0002			1.084		kz0036	
79	Cinnamic acid	Phenolic acids	9	0.35135	91	2-Picolinic acid	Organic acids	05	2.63652	90	Que
kz0031			1.0		km008			1.056		kz0005	
16	2,4,6-trihydroxy benzoic acid	Organic acids	3	0.35629	1	Genistein (4',5,7-Trihydroxyisoflavone)	Isoflavones	75	2.63936	60	Trih
km022	Quercetin 3-O-glucoside (Isotrifoliin)	Flavonols	1.0	0.35728	kz0011	Indole-3-carboxaldehyde	Alkaloids	1.037	2.66086	kz0007	Lut

8			9	
kz0000			1.0	
56	Salicylic acid glucoside	Phenolic acids	9	0.36239
kz0005			1.0	
57	Glucosyringic Acid	Phenolic acids	8	0.36495
kz0010			1.0	
75	Methyl gallate	Phenolic acids	8	0.36712
kz0004			1.0	
95	3-(4-Hydroxyphenyl)-propionic acid	Phenolic acids	9	0.36834
kz0023			1.0	
37	Luteolin-7-O-β-D-gentiobioside	Flavones	5	0.37482
kz0011			1.0	
54	Nicotinamide	Vitamins	9	0.38506
kz0025			1.0	
58	6-O-caffeoyl-β-glucose	Phenolic acids	8	0.38880
kz0031			1.0	
08	mlyricetin 3-O-B-D-glucopyranoside	Flavonols	6	0.39196
kz0050			1.0	
54	Chrysin-7-glucoside	Flavones	8	0.39706
km032			1.0	
9	3-(4-Hydroxyphenyl)propionic acid	Phenolic acids	9	0.40153
kz0020			1.0	
08	3,5,7,4'--Tetrahydroxy-Coumaronochromone	Others	5	0.40591
kz0004			1.0	
14	2-Picolylamine	Alkaloids	7	0.43198
kz0017			1.0	
10	Stilbostemin B	Others	6	0.43279
kz0010			1.0	
41	N-Acetyl-D-glucosamine 1-phosphate	Others	9	0.43855
kz0009			1.0	
15	6-Hydroxykaempferol-7,6-O-Diglucoside	Flavonols	9	0.44120
kz0029			1.0	
33	Ixerin D	Terpenoids	8	0.44150
kz0000			1.0	
47	Mandelic acid	Phenolic acids	9	0.44222
kz0011			1.0	
75	Esculetin	Coumarins	6	0.44751
kz0005			1.0	
49	1-O-[(E)-Caffeoyl]-β-D-glucopyranose	Phenolic acids	8	0.44969
kz0001			1.0	
21	Chrysin 5-O-glucoside (Toringin)	Flavones	8	0.45566
kz0005	Coniferin	Phenolic acids	1.0	0.45730

99			26	
kz0002			1.079	
58	Nicotinate D-ribonucleoside	Vitamins	01	2.68000
kz0005			1.081	
77	Hexahydroxydiphenoylglucose	Phenolic acids	42	2.70811
kz0053			1.073	
70	(+)-Isolariciresinol	Lignans	73	2.86395
kz0012			1.083	
93	4-Guanidinobutyric acid	Organic acids	18	2.90886
kz0002			1.081	
96	Citramalate	Organic acids	02	2.95580
kz0053			1.082	
55	Koaburaside	Phenolic acids	91	2.99645
kz0041			1.072	
69	Isorhamnetin 3-O-β-D-Glucoside	Flavonols	28	3.08092
kz0011			1.082	
63	Riboflavin	Vitamins	34	3.12951
kz0015			1.081	
81	Calceorioside B	Others	20	3.24356
kz0028			1.075	
29	Isorhamnetin-7-O-glucoside	Flavonols	44	3.26793
kz0011			1.073	
13	Lumichrome	Alkaloids	67	3.32550
kz0007			1.076	
91	Apigenin 7-O-glucoside(Cosmosiin)	Flavones	73	3.36863
kz0033			1.040	
70	Swertiamarin	Terpenoids	66	3.38871
kz0009			1.078	
11	Quercetin-7-O-rutinoside	Flavonols	83	3.40781
kz0001			1.079	
47	Methylquercetin O-hexoside	Flavonols	27	3.41294
kz0010			1.083	
20	4-Methyl-5-thiazoleethanol	Others	88	3.42270
kz0050			1.080	
88	Tricetin?4'-methyl?ether-3'-O-β-D-glucoside	Flavonols	49	3.45499
kz0041			1.084	
70	Rhamnetin 3-O-β-D-Glucoside	Flavonols	02	3.49603
kz0013			1.083	
18	Quinic Acid	Organic acids	24	3.49885
kz0029			1.082	
82	6-methoxykaempferol-3-O-glucoside	Flavonols	01	3.50433
kz0041	Quercetin 3-O-β-D-neohesperidoside	Flavonols	1.081	3.55085

95	
kz0050	
46	Dihy
kz0053	
73	Lyo
kz0050	
79	Kae
kz0010	
18	3-A
kz0038	
21	Kam
kz0026	
04	Kae
kz0025	
60	6-O
kz0005	
40	3,4,
kz0013	
08	D-X
kz0025	
57	4-O
kz0004	
75	4-H
kz0027	
83	Kae
kz0009	
95	Syri
kz0008	
12	Tric
kz0002	
40	Tran
kz0015	
84	Que
kz0051	
86	Dur
kz0004	
31	Erio
kz0025	
48	p-Co
kz0004	
66	4-H
km009	Oro

50			9		73			45		8	
kz0011			1.0		kz0038			1.037		kz0038	
55	Nicotinic acid	Vitamins	8	0.46114	59	p-Coumaroyleuscaphic acid	Terpenoids	23	3.60244	29	Que
kz0052			1.0		kz0009			1.083		kz0051	
02	Dihydrokaempferol	Flavanols	6	0.47322	09	Quercetin-3-O-rutinoside (Rutin)	Flavonols	08	3.61553	98	Pon
kz0046			1.0		kz0005			1.079		kz0035	
97	Taxifolin-3'-O-β-D-glucoside	Flavones	5	0.49424	00	Riboprine	Phenolic acids	71	3.65002	72	Bior
kz0002			1.0		km130			1.078		kz0033	
91	2-Picolinic acid	Organic acids	8	0.49728	7	Isorhamnetin 5-O-hexoside	Flavonols	24	3.69444	62	Tub
kz0008			1.0		kz0009			1.081		kz0046	
88	Quercetin-3-O-β-D-Galactoside (Hyperin)	Flavonols	8	0.49761	08	Quercetin-3-O-robinobioside	Flavonols	41	3.70093	67	Myr
kz0008			1.0		kz0038			1.055		kz0009	
89	Spiraeoside	Flavonols	9	0.49960	12	Isorhamnetin O-malonylglucoside	Flavonols	48	3.74847	04	Tilir
kz0030			1.0		kz0009			1.082		kz0051	
97	3,4-Di-O-galloyl-shikimic acid	Phenolic acids	9	2.01010	71	Catechin	Flavanols	78	3.83453	32	Lute
kz0010			1.0		km140			1.080		kz0017	
93	Oleanolic acid 2-O-β-D-glucopyranoside	Terpenoids	9	2.16236	5	Nicotinic acid-hexoside	Vitamins	27	4.01995	39	Que
km126			1.0		kz0007			1.082		km140	
9	3,7-Di-O-methylquercetin	Flavonols	9	2.21042	92	Apigenin 5-O-glucoside	Flavones	44	4.03128	5	Nico
kz0051			1.0		kz0013			1.069		kz0017	
36	Camaldulenic acid	Terpenoids	9	2.22589	12	Shikimic acid	Organic acids	94	4.04146	42	Met
kz0009			1.0		kz0024			1.063		kz0040	
11	Quercetin-7-O-rutinoside	Flavonols	8	2.25872	85	3-methoxy-juglone	Quinones	90	4.23004	15	Cis-
kz0037			1.0		kz0040			1.018		km023	
28	Geniposide	Terpenoids	1	2.26921	35	3-Indoleacrylic acid	Alkaloids	73	4.26822	5	Apig
kz0052			1.0		kz0009			1.083		kz0037	
00	Hesperetin 7-O-neohesperidoside(Neohesperidin)	Flavanones	9	2.27246	95	Syringaresinol	Lignans	30	4.31214	63	Betu
kz0009			1.0		kz0053			1.071		kz0011	
20	Quercetin-O-rutinoside-hexose	Flavonols	0	2.27826	73	Lyoniresinol	Lignans	84	4.31764	93	Prur
kz0041			1.0		km060			1.064		km008	
73	Quercetin 3-O-neohesperidoside	Flavonols	5	2.27926	2	Genistein 7-O-Glucoside (Genistin)	Isoflavones	79	4.32852	0	Bioc
kz0002			1.0		kz0053			1.071		kz0005	
74	N-sinapoylhydroxycoumarin	Coumarins	6	2.29496	78	5'-methoxyisolariciresinol-9'-O-β-D-glucopyranoside	Lignans	30	4.46792	73	p-Co
kz0009			1.0		kz0037			1.061		kz0037	
09	Quercetin-3-O-rutinoside (Rutin)	Flavonols	8	2.36976	94	Syringic Aldehyde-glucoside	Phenolic acids	44	4.49612	21	Map
kz0005			1.0		kz0009			1.079		kz0004	
42	1-O-Galloyl-β-D-glucose	Phenolic acids	9	2.43943	12	Isorhamnetin-3-O-rutinoside (Narcissin)	Flavonols	99	4.53057	19	Phlo
km023			1.0		kz0050			1.068		km023	
5	Apigenin	Flavones	6	2.46446	55	Galangin-7-glucoside	Flavones	52	4.57255	1	2'-H
km130			1.0		kz0031			1.081		km008	
0	Hydroxy-methoxycinnamate	Phenolic acids	8	2.48952	09	Mearnsitrin	Flavones	67	4.58442	1	Ger
kz0034	2-O-Galloyl-β-D-glucose	Phenolic acids	1.0	2.50889	kz0004	Vanillic acid	Phenolic acids	1.081	4.62008	kz0015	Isot

98			9		96			41		86	
kz0053			1.0		kz0021			1.061		kz0053	
78	5'-methoxyisolariciresinol-9'-O-β-D-glucopyranoside	Lignans	8	2.54005	96	2'-Hydoxy,5-methoxy Genistein-O-rhamnosyl-glucoside	Isoflavones	53	4.64540	78	5'-m
kz0027			1.0		kz0035			1.082		kz0021	
83	Kaempferol 3-O-β-d-(6''-O-(E)-p-coumaroyl) glucopyranoside	Flavonols	9	2.59149	17	Glucosyloxybenzoic acid	Phenolic acids	23	4.65532	64	Isol
kz0012			1.0		kz0013		Proanthocyani	1.083		kz0038	
75	Methylmalonic acid	Organic acids	9	2.62644	32	Procyanidin B2	dins	67	4.76062	50	(-)-s
kz0013			1.0		kz0027			1.053		kz0001	
15	Anchoic Acid	Organic acids	7	2.63482	88	Sexangularetin 3-glucoside-7-rhamnoside	Flavonols	73	4.80315	90	Arb
kz0011			1.0		kz0004			1.066		kz0020	
01	Trigonelline	Alkaloids	9	2.70794	66	4-Hydroxybenzaldehyde	Phenolic acids	42	4.83881	65	4-α-
kz0019			1.0		kz0038			1.079		kz0025	
73	Kaempferol-3-O-(cinnamoyl)-sophoroside-7-O-glucose	Flavonols	8	2.73346	15	Kaempferol 3-O-(6''-trans-p-Coumaroyl)-β-D-glucopyranoside	Flavonols	61	4.94193	49	Van
kz0004			1.0		kz0013		Proanthocyani	1.082		kz0010	
96	Vanillic acid	Phenolic acids	9	2.73791	35	Procyanidin B1	dins	02	4.96513	75	Met
km008			1.0		kz0025			1.082		kz0005	
1	Genistein (4',5,7-Trihydroxyisoflavone)	Isoflavones	7	2.75563	56	4-O-glucosyl-4-hydroxybenzoic acid	Phenolic acids	42	4.98115	00	Ribo
kz0002			1.0		kz0025			1.078		kz0005	
90	Aminomalonic acid	Organic acids	8	2.76608	52	Sinapic acid-glycoside	Phenolic acids	11	5.03627	15	Sina
kz0012			1.0		kz0013		Proanthocyani	1.082		kz0038	
76	Succinic acid	Organic acids	9	2.76660	33	Procyanidin B3	dins	58	5.05686	49	dihy
kz0032			1.0		kz0005			1.069		kz0047	
62	Ayapin	Coumarins	7	2.79587	15	Sinapinaldehyde	Phenolic acids	99	5.23262	38	4,7,
km023			1.0		kz0041			1.058		kz0004	
1	2'-Hydroxygenistein	Isoflavones	1	2.81098	72	Quercetin 3-O-β-D-xylopyranosyl(1→2)-β-D-galactopyranoside	Flavonols	43	5.33866	96	Van
kz0005			1.0		kz0025			1.081		kz0025	
02	Coniferaldehyde	Phenolic acids	7	2.85857	46	Isosalicylic acid O-glycoside	Phenolic acids	65	5.46608	52	Sina
kz0004			1.0		kz0009			1.072		km093	
77	Tyrosol	Phenolic acids	5	2.86277	14	6-Hydroxykaempferol-3,6-O-Diglucoside	Flavonols	92	5.61705	4	Que
kz0009			1.0		kz0013			1.082		kz0030	
08	Quercetin-3-O-robinobioside	Flavonols	9	2.91491	08	D-Xylonic acid	Organic acids	83	5.99301	97	3,4-
kz0018			1.0		kz0051			1.073		kz0008	
19	Salicylic acid	Phenolic acids	7	2.94087	37	Isoscopoletin (6-Hydroxy-7-Methoxycoumarin)	Coumarins	84	6.01299	90	Que
kz0013			1.0		kz0052			1.082		kz0053	
13	SubericAcid	Organic acids	1	2.97894	00	Hesperetin 7-O-neohesperidoside(Neohesperidin)	Flavanones	82	6.03036	75	Isola
kz0002			1.0		kz0016			1.078		kz0012	
01	D-galacitol	Others	9	2.99669	37	Oresbiusin A	Phenolic acids	57	6.04555	93	4-G
kz0004			1.0		kz0004			1.071		kz0000	
84	Vanillin	Phenolic acids	9	3.02751	84	Vanillin	Phenolic acids	48	6.34304	32	Caff
kz0035			1.0		kz0013		Proanthocyani	1.079		kz0009	
72	Biondnoid I	Flavonols	9	3.04365	40	Procyanidin C2	dins	07	6.51831	15	6-H
kz0052	Luteolin 7-O-neohesperidoside(Lonicerin)	Flavones	1.0	3.10010	kz0009	Epigallocatechin (EGC)	Flavanols	1.078	7.02128	kz0012	4-Ac

25			5		73			70		92	
kz0005			1.0		kz0025			1.083		km054	
07	Syringic Aldehyde	Phenolic acids	9	3.20996	57	4-O-glucosyl-3,4-dihydroxybenzyl alcohol	Phenolic acids	49	7.49347	8	Dihy
km009			1.0		kz0005			1.075		kz0004	
8	Orobol (5,7,3',4'-tetrahydroxyisoflavone)	Isoflavones	2	3.33158	92	Rosmarinyl Glucoside	Phenolic acids	42	7.87865	12	Auc
kz0012			1.0		kz0037			1.075		kz0010	
83	6-Aminocaproic acid	Organic acids	3	3.34598	55	Quercetin-3-sambubioside	Flavonols	53	8.08777	25	D-G
kz0003			1.0		kz0009			1.078		kz0030	
02	2-Isopropylmalate	Organic acids	7	3.35369	74	Gallocatechin	Flavanols	48	8.19840	98	3-O
kz0030			1.0		km054			1.083		kz0038	
38	3-O-(2-O-Acetyl-β-D-glucopyranosyl) oleanolic acid	Terpenoids	9	3.36272	8	Dihydroquercetin (Taxifolin)	Flavonols	07	8.36915	09	Sieb
kz0009			1.0		kz0045			1.082		km126	
04	Tiliroside	Flavonols	9	3.37255	98	1,4-di-O-galloyl-β-D-glcose	Phenolic acids	97	9.74608	9	Di-
kz0021			1.0		kz0009			1.066		kz0000	
97	2'-Hydoxy,5-methoxy Genistein-4',7-O-diglucoside	Isoflavones	5	3.43186	07	Kaempferol-3-O-glucoside-7-O-rhamnoside	Flavonols	50	10.02257	68	Cate
kz0051			1.0		kz0005			1.079		kz0023	
32	Luteolin-caffeoyl-O-rhamnoside	Flavones	9	3.43414	80	1,6-Di-O-Galloyl-D-Glucose	Phenolic acids	75	10.11315	37	Lute
kz0021			1.0		kz0011			1.082		kz0003	
64	Isoluteolin (Orobol)(5,7,3',4'-tetrahydroxyisoflavone)	Isoflavones	6	3.45178	58	D-Pantothenic Acid	Vitamins	55	10.49764	02	2-Is
kz0051			1.0		kz0025			1.081		kz0031	
98	Poncirin(Isosakuranetin-7-neohesperidoside)	Flavanones	9	3.46502	60	6-O-feruloyl-α-glucose	Phenolic acids	82	10.58986	58	Aila
kz0022			1.0		kz0052			1.073		kz0004	
23	Eucommia	Others	8	3.52995	25	Luteolin 7-O-neohesperidoside(Lonicerin)	Flavones	51	10.97861	17	Phlo
kz0016			1.0		kz0029			1.074		kz0037	
42	5'-Glucopyranosyloxyjasmanic acid	Phenolic acids	9	3.65824	90	Luteolin-7-O-rutinoside	Flavones	35	11.26988	55	Que
kz0038			1.0		kz0038			1.075		kz0052	
72	Lup-12-en-15α,19β-diol-3,11-dioxo-28-oic acid	Terpenoids	9	3.68675	09	Sieboldin	Chalcones	00	11.50213	00	Hes
kz0037			1.0		kz0001			1.083		kz0001	
67	Obtusilin	Terpenoids	9	3.75492	90	Arbutin	Others	51	11.89655	97	E-3,
kz0005			1.0		kz0041			1.080		kz0053	
33	2,5-Dihydroxy benzoic acid O-hexside	Phenolic acids	8	3.77998	71	Kaempferol 3-O-β-D-neohesperidoside	Flavonols	66	12.40464	70	(+)-
kz0001			1.0		kz0005			1.084		kz0018	
97	E-3,4,5'-Trihydroxy-3'-glucopyranosylstilbene	Others	5	3.81671	57	Glucosyringic Acid	Phenolic acids	53	12.48132	80	feru
kz0011			1.0		kz0009			1.083		kz0031	
88	Esculin(6,7-DihydroxyCoumarin-6-glucoside)	Coumarins	7	3.92966	05	Kaempferol-3-O-rutinoside(Nicotiflorin)	Flavonols	99	12.59061	47	Clem
kz0017			1.0		kz0004			1.083		kz0016	
06	Isololiolide	Others	9	4.02706	12	Aucubin	Terpenoids	98	12.86942	37	Ore
km143			1.0		kz0009			1.084		kz0052	
7	p-Aminobenzoate	Phenolic acids	8	4.05827	06	Kaempferol-3-O-robinobioside(Biorobin)	Flavonols	53	13.11181	02	Dihy
kz0004			1.0		kz0031			1.084		kz0037	
75	4-Hydroxybenzoic acid	Phenolic acids	9	4.06836	66	6-Hydroxy-7-methoxycoumarin	Coumarins	51	13.40018	48	7'-C
kz0002	5-Aminolevulinate	Alkaloids	1.0	4.19263	kz0026	Quercetin-3-O-(6''-O-acetyl)-galactoside	Flavonols	1.082	13.52593	km057	Epic

31			3		78			37		3	
kz0009			1.0		kz0000			1.066		kz0011	
07	Kaempferol-3-O-glucoside-7-O-rhamnoside	Flavonols	4	4.46098	66	Feruloyl syringic acid	Phenolic acids	42	15.59173	58	D-P
kz0033			1.0		kz0005			1.084		kz0038	
62	Tubuloside C	Phenolic acids	8	4.53828	40	3,4,5-Trimethoxyphenyl-β-D-Glucopyranoside	Phenolic acids	75	16.12622	15	Kae
kz0041			1.0		kz0005			1.076		kz0031	
71	Kaempferol 3-O-β-D-neohesperidoside	Flavonols	8	4.75676	07	Syringic Aldehyde	Phenolic acids	93	16.87097	51	Aila
kz0016			1.0		kz0026			1.082		kz0004	
37	Oresbiusin A	Phenolic acids	9	4.91773	04	Kaempferol-3-O-(6''-acetyl)-glucoside	Flavonols	18	19.48803	24	Eri
kz0010			1.0		kz0036			1.081		kz0012	
84	Ursolic acid	Terpenoids	8	4.95469	93	Quercetin-3-O-(2-O-α-L-rhamnopyranosyl)-β-D-galactopyranoside	Flavonols	99	21.51981	98	L-(+
kz0029			1.0		kz0000			1.084		kz0009	
90	Luteolin-7-O-rutinoside	Flavones	8	5.01229	55	3,4,5-Trimethoxycinnamic acid	Phenolic acids	74	28.30000	73	Epig
kz0008			1.0		kz0013			1.079		kz0046	
86	Myricetin-3-O-arabinoside	Flavonols	5	5.13683	80	Quercetin-O-rutinoside-O-rhamnoside	Flavonols	72	30.39177	30	2α-
km087			1.0		kz0052			1.084		kz0051	
9	Orotic acid	Vitamins	9	5.56304	02	Dihydrokaempferol	Flavanols	65	31.64993	37	Isos
kz0005			1.0		kz0031			1.083		kz0030	
05	Coniferyl alcohol	Phenolic acids	7	5.63649	51	Ailantinol E	Others	78	36.44068	83	5,2'
kz0047			1.0		kz0038			1.081		kz0005	
56	N-Benzylmethylene isomethylamine	Alkaloids	6	5.99230	50	(-)-secoisolariciresinol 4-O-β-D-giucopyranoside	Lignans	58	58.96861	33	2,5-
kz0010			1.0		kz0047			1.079		kz0012	
25	D-Glucurono-6,3-lactone	Others	9	6.15586	03	secoisolariciresinol 9-O-β-D-glucopyranoside	Lignans	67	89.36559	03	Ind
kz0046			1.0		kz0009			1.083		kz0024	
78	Annuionone D	Others	9	6.24479	20	Quercetin-O-rutinoside-hexose	Flavonols	82	90.30000	85	3-m
kz0037			1.0		kz0010			1.081		kz0004	
63	Betulin	Terpenoids	9	6.82881	14	1-Methylhistamine	Others	72	109.82500	84	Van
kz0009			1.0		kz0007			1.083		kz0009	
06	Kaempferol-3-O-robinobioside(Biorobin)	Flavonols	9	6.83350	98	Luteolin-7-O-glucuronide	Flavones	73	116.37209	74	Gall
kz0009			1.0		kz0029			1.084		kz0037	
05	Kaempferol-3-O-rutinoside(Nicotiflorin)	Flavonols	9	6.87266	76	Scutellarin(Scutellarein-7-O-glucuronide)	Flavones	55	126.76617	18	2,3-
kz0015			1.0		kz0002			1.083		kz0040	
86	Isotamarixin	Flavonols	8	6.96179	78	Indole 3-acetic acid (IAA)	Alkaloids	78	127.50000	35	3-In
kz0011			1.0		kz0017			1.083		kz0011	
03	6-Hydroxynicotinic acid	Alkaloids	8	7.14787	02	Kaempferol-3-O-β-D-glucuronide	Flavonols	55	136.59794	99	Ind
kz0026			1.0		kz0007			1.084		kz0013	
65	Betulinic acid	Terpenoids	3	8.36538	97	Tetahydroxy-flavone-7-O-β-D-glucuronide	Flavones	35	138.95170	19	D-G
kz0011			1.0		kz0030			1.083		kz0005	
13	Lumichrome	Alkaloids	8	8.55081	98	3-O-Digalloyl quinic acid	Phenolic acids	05	215.55556	07	Syri
kz0005			1.0		kz0005			1.084		kz0010	
44	3-O-p-Coumaroyl quinic acid	Phenolic acids	9	10.46030	96	β-D-Furanofructosyl-α-D-(6-mustard acyl)glucoside	Phenolic acids	37	257.25000	84	Urs
kz0029	Scutellarin(Scutellarein-7-O-glucuronide)	Flavones	1.0	11.30743	kz0018	vnilloylcaffeoyltartaric acid	Phenolic acids	1.084	263.00000	kz0010	Gall

76			9	
kz0007			1.0	
98	Luteolin-7-O-glucuronide	Flavones	9	11.79213
kz0013			1.0	
19	D-Galacturonic acid(Gal A)	Organic acids	9	11.97323
kz0050			1.0	
37	Caffeoylferuloylshikimic?acid	Phenolic acids	9	12.41192
kz0017			1.0	
02	Kaempferol-3-O-β-D-glucuronide	Flavonols	9	13.84675
kz0036			1.0	
93	Quercetin-3-O-(2-O-α-L-rhamnopyranosyl)-β-D-galactopyranoside	Flavonols	9	14.14724
kz0011			1.0	
99	Indole-3-carboxaldehyde	Alkaloids	9	14.82988
kz0020			1.0	
65	4-α-L-Rhamnopyranosyl-ellagic acid	Phenolic acids	9	15.09756
kz0007			1.0	
97	Tetahydroxy-flavone-7-O-β-D-glucuronide	Flavones	9	16.12777
kz0012			1.0	
03	Indole-3-carboxylic acid	Alkaloids	9	17.49424
kz0005			1.0	
15	Sinapinaldehyde	Phenolic acids	9	17.95407
kz0001			1.0	
45	Formononetin	Isoflavones	9	18.39138
kz0004			1.0	
66	4-Hydroxybenzaldehyde	Phenolic acids	9	18.50517
kz0046			1.0	
67	Myricetin-O-rhamnoside	Flavonols	9	22.67361
kz0010			1.0	
90	Methoxyursolic acid	Terpenoids	9	24.69359
kz0013			1.0	
80	Quercetin-O-rutinoside-O-rhamnoside	Flavonols	8	24.71264
km093			1.0	
4	Quercetin 4'-O-glucoside (Spiraeoside)	Flavonols	9	30.06167
kz0029			1.0	
36	Silibinin	Flavanols	9	32.20000
kz0008			1.0	
90	Quercetin-7-O-Glucoside	Flavonols	9	32.38600
kz0031			1.0	
66	6-Hydroxy-7-methoxycoumarin	Coumarins	9	58.66864
kz0005			1.0	
00	Riboprine	Phenolic acids	9	63.19018
kz0044	Luteolin-O-rutinoside-O-rhamnoside	Flavones	1.0	78.87500

64			28	
kz0033			1.083	
62	Tubuloside C	Phenolic acids	37	285.89909
kz0000			1.083	
47	Mandelic acid	Phenolic acids	96	431.75000
kz0005			1.084	
17	Sinapyl alcohol	Phenolic acids	58	483.50000
kz0005			1.085	
05	Coniferyl alcohol	Phenolic acids	01	502.00000
kz0021			1.084	
44	Angelicin	Coumarins	74	645.75000
kz0005			1.084	
02	Coniferaldehyde	Phenolic acids	75	699.25000
kz0008			1.084	1325.0000
72	Myricetin	Flavonols	97	0
kz0051			1.084	1340.0000
33	Luteolin-O-glucuronate-O-rhamnoside	Flavones	82	0
kz0009			1.084	1877.5000
19	Robinin(Kaempferol-3-O-gal-rham-7-O-rham)	Flavonols	97	0
kz0044			1.084	1907.5000
25	Luteolin-O-rutinoside-O-rhamnoside	Flavones	96	0
kz0037			1.084	2247.5000
97	3-Prenyl-4-O-β-D-glucopyranosyloxy-4-hydroxyl-benzoic acid	Phenolic acids	92	0
kz0022			1.084	2572.5000
95	Syringaresinol-4'-O-β-D-monO-glucoside	Lignans	70	0
km143			1.084	2955.0000
7	p-Aminobenzoate	Phenolic acids	99	0
kz0029			1.084	5390.0000
12	Santamarin	Terpenoids	92	0
kz0029			1.084	7915.0000
36	Silibinin	Flavanols	99	0
kz0000			1.084	9030.0000
78	1'-O-β-D-(3,4-Dihydroxyphenethyl)-O-caffeoyl-glucoside	Phenolic acids	99	0
kz0042			1.085	18800.000
60	Corosolic acid	Terpenoids	00	00
km093			1.085	155750.00
6	Quercetin 7-O-β-D-Glucuronide	Flavonols	01	000

74	
kz0007	
98	Lute
kz0029	
76	Scu
kz0007	
97	Teta
kz0017	
02	Kae
kz0037	
96	1'-C
kz0009	
14	6-H
kz0000	
52	Ethy
kz0000	
55	3,4,
kz0047	
03	seco
kz0037	
28	Ger
kz0009	
20	Que
kz0029	
75	Aca
kz0010	
14	1-M
kz0008	
72	Myr
kz0007	
33	Delp
kz0005	
75	3,5-
kz0047	
68	mor
kz0045	
95	Ger
kz0035	
04	Ger
kz0010	
76	Ethy
kz0029	San

25			9	
kz000919	Robinin(Kaempferol-3-O-gal-rham-7-O-rham)	Flavonols	1.09	90.35000
kz000733	Delphinidin-3,5-O-diglucoside	Anthocyanins	1.09	92.20000
kz000278	Indole 3-acetic acid (IAA)	Alkaloids	1.09	125.75000
kz003634	Sinapaldehyde Glucoside	Phenolic acids	1.09	197.25000
kz002912	Santamarin	Terpenoids	1.09	264.50000
kz000580	1,6-Di-O-Galloyl-D-Glucose	Phenolic acids	1.09	330.00000
kz004598	1,4-di-O-galloyl-β-D-glcose	Phenolic acids	1.09	343.00000
kz000173	3-Hydroxypyridine	Others	1.09	389.50000
kz001284	Oxaloacetic acid	Organic acids	1.09	601.50000
kz001834	feruloylmalic acid	Phenolic acids	1.09	733.00000
kz003833	Quercetin-O-pentosyl-O-rhamnoside-O-glucoside	Flavonols	1.09	934.25000
kz000078	1'-O-β-D-(3,4-Dihydroxyphenethyl)-O-caffeoyl-glucoside	Phenolic acids	1.09	1307.50000
kz001014	1-Methylhistamine	Others	1.09	1512.50000
kz000551	methyl 6-O-galloyl-β-D-glucopyranoside	Phenolic acids	1.09	5045.00000
kz004172	Quercetin 3-O-β-D-xylopyranosyl-(1→2)-β-D-galactopyranoside	Flavonols	1.09	11337.50000
kz003755	Quercetin-3-sambubioside	Flavonols	1.09	11525.00000
km0936	Quercetin 7-O-β-D-Glucuronide	Flavonols	1.09	21775.00000

12	
kz000278	Indo
kz001680	6-hy
kz000596	β-D
kz000173	3-H
kz000886	Myr
kz001307	Phtl
kz002936	Silib
kz000505	Con
kz000502	Con
kz001864	vnil
kz000207	Can
kz000517	Sina
kz000047	Mar
kz000078	1'-O
kz004260	Cor
kz002295	Syri
km1437	p-A
km0936	Que

Table S2 Continued

RSS-F/RSL-F					RLM-F/RDP-F					RLM-F/RSL-F					RDP-F/RSL-F				
Index	Compounds	Class	VIP	Fold_Change	Index	Compounds	Class	VIP	Fold_Change	Index	Compounds	Class	VIP	Fold_Change	Index	Compounds	Class	VIP	Fold_Change

				e					e					e					e
kz0050449	Salireposide	Phenolic acids	1.07387	0.00005	kz0037794	Syringic Aldehyde-glucoside	Phenolic acids	1.07116	0.00041	kz0013332	Procyanidin B2	Proanthocyanidins	1.08189	0.00005	kz0013332	Procyanidin B2	Proanthocyanidins	1.09247	0.00008
kz0046698	7S,8R-threo-3′,9,9′-trihydroxy-3-methoxy-4′,7-epoxy-neolignan-4-O-α-L-rhamnopyranoside	Lignans	1.07400	0.00006	kz0037997	3-Prenyl-4-O-β-D-glucopyranosyloxy-4-hydroxyl-benzoic acid	Phenolic acids	1.07131	0.00044	kz0042600	Corosolic acid	Terpenoids	1.08192	0.00005	kz0013335	Procyanidin B1	Proanthocyanidins	1.09248	0.00010
kz0005300	Brevifolin carboxylic acid	Phenolic acids	1.07333	0.00021	kz0044425	Luteolin-O-rutinoside-O-rhamnoside	Flavones	1.07144	0.00052	kz0013335	Procyanidin B1	Proanthocyanidins	1.08187	0.00006	kz0013333	Procyanidin B3	Proanthocyanidins	1.09247	0.00011
kz0013332	Procyanidin B2	Proanthocyanidins	1.07399	0.00022	kz0009919	Robinin(Kaempferol-3-O-gal-rham-7-O-rham)	Flavonols	1.07147	0.00053	kz0013333	Procyanidin B3	Proanthocyanidins	1.08188	0.00006	kz0005300	Brevifolin carboxylic acid	Phenolic acids	1.09194	0.00019
kz0007004	Cyanidin-O-syringic acid	Anthocyanins	1.07393	0.00025	kz0051313	Luteolin-O-glucuronate-O-rhamnoside	Flavones	1.07118	0.00075	kz0047003	secoisolariciresinol 9-O-β-D-glucopyranoside	Lignans	1.08184	0.00020	kz0007004	Cyanidin-O-syringic acid	Anthocyanins	1.09249	0.00020
kz0013335	Procyanidin B1	Proanthocyanidins	1.07397	0.00028	kz0021444	Angelicin	Coumarins	1.07109	0.00155	kz0005592	Rosmarinyl Glucoside	Phenolic acids	1.08194	0.00024	kz0009974	Gallocatechin	Flavanols	1.09253	0.00027
kz0013333	Procyanidin B3	Proanthocyanidins	1.07395	0.00030	kz0050449	Salireposide	Phenolic acids	1.07122	0.00185	kz0007004	Cyanidin-O-syringic acid	Anthocyanins	1.08194	0.00024	kz0042600	Corosolic acid	Terpenoids	1.09236	0.00028
kz0038862	3β-Hydroxy-28-norurs-17,19,21-trien	Terpenoids	1.07376	0.00038	kz0041172	Quercetin 3-O-β-D-xylopyranosyl(1→2)-β-D-galactopyranoside	Flavonols	1.07144	0.00193	kz0037794	Syringic Aldehyde-glucoside	Phenolic acids	1.08159	0.00041	kz0007003	Delphinidin-3-O-glucoside (Mirtillin)	Anthocyanins	1.09073	0.00029
kz0053777	lyoniresinol-9'-O-β-D-xylopyranoside	Lignans	1.07396	0.00039	km0818	Myricetin 3-O-rhamnoside (Myricitrin)	Flavonols	1.07094	0.00467	kz0013400	Procyanidin C2	Proanthocyanidins	1.08193	0.00044	kz0047003	secoisolariciresinol 9-O-β-D-glucopyranoside	Lignans	1.09238	0.00032
kz0007003	Delphinidin-3-O-glucoside (Mirtillin)	Anthocyanins	1.07392	0.00057	kz0001177	Scoparone	Coumarins	1.07057	0.00496	kz0009974	Gallocatechin	Flavanols	1.08189	0.00056	kz0038821	Kampferol 3-O-(6″-galloyl)-β-D-galactopyranoside	Flavonols	1.09221	0.00038
kz0	Homogentisic acid	Pheno	1.	0.00	kz0	Quercetin-O-pentosyl-O-	Flavon	1.	0.00	kz0	Sieboldin	Chalco	1.	0.00	kz0	Kampferol 3-O-(2″-	Flavon	1.	0.00

00050		lic acids	07379	083	03833	rhamnoside-O-glucoside	ols	06589	748	03809		nes	08167	064	03823	galloyl)-β-D-galactopyranoside	ols	09245	040
kz0004774	(2R)-Pinocembrin-7-neohesperidoside	Flavanones	1.07391	0.00084	kz0003166	6-Hydroxy-7-methoxycoumarin	Coumarins	1.07144	0.01828	kz0002557	4-O-glucosyl-3,4-dihydroxybenzyl alcohol	Phenolic acids	1.08186	0.00070	kz0000973	Epigallocatechin (EGC)	Flavanols	1.09241	0.00046
kz0003717	Maplexin C (2,3-Di-O-Galloyl-1,5-Anhydro-D-Glucitol)	Phenolic acids	1.07378	0.00089	kz0003362	Tubuloside C	Phenolic acids	1.07123	0.01984	kz0005133	Luteolin-O-glucuronate-O-rhamnoside	Flavones	1.08172	0.00075	kz0000592	Rosmarinyl Glucoside	Phenolic acids	1.09211	0.00068
kz0002820	Limocitrin 7-glucoside	Flavonols	1.07376	0.00091	kz0003693	Quercetin-3-O-(2-O-α-L-rhamnopyranosyl)-β-D-galactopyranoside	Flavonols	1.06887	0.03826	kz0000872	Myricetin	Flavonols	1.08191	0.00075	kz0000186	2-Deoxyribose 1-phosphate	Others	1.09246	0.00079
kz0001191	4-hydroxycoumarin di-glucoside	Coumarins	1.07388	0.00092	kz0001380	Quercetin-O-rutinoside-O-rhamnoside	Flavonols	1.06650	0.04176	kz0000973	Epigallocatechin (EGC)	Flavanols	1.08192	0.00087	kz0003809	Sieboldin	Chalcones	1.09240	0.00079
kz0002551	Isosinapic acid-hexoside	Phenolic acids	1.07346	0.00103	kz0003098	3-O-Digalloyl quinic acid	Phenolic acids	1.06973	0.04336	kz0000703	Delphinidin-3-O-glucoside (Mirtillin)	Anthocyanins	1.07738	0.00114	kz0003717	Maplexin C (2,3-Di-O-Galloyl-1,5-Anhydro-D-Glucitol)	Phenolic acids	1.09180	0.00084
km0640	Hesperetin 7-rutinoside (Hesperidin)	Flavanones	1.07387	0.00110	kz0002912	Santamarin	Terpenoids	1.06710	0.05798	kz0002551	Isosinapic acid-hexoside	Phenolic acids	1.08190	0.00135	kz0000546	1-O-p-Coumaroyl quinic acid	Phenolic acids	1.09246	0.00085
kz0000546	1-O-p-Coumaroyl quinic acid	Phenolic acids	1.07386	0.00110	kz0002936	Silibinin	Flavanols	1.07101	0.07037	kz0002144	Angelicin	Coumarins	1.08160	0.00155	kz0000517	Sinapyl alcohol	Phenolic acids	1.09243	0.00085
kz0002202	Farrerol 7-O-glucoside	Flavanones	1.07397	0.00118	kz0001318	Quinic Acid	Organic acids	1.07121	0.07125	kz0000555	1-Caffeoylquinic acid	Phenolic acids	1.08096	0.00167	kz0000032	Caffeoylagmatine	Alkaloids	1.09253	0.00094
kz0000553	Neochlorogenic acid(5-O-Caffeoylquinic acid)	Phenolic acids	1.07355	0.00119	kz0000907	Kaempferol-3-O-glucoside-7-O-rhamnoside	Flavonols	1.06225	0.08636	kz0000050	Homogentisic acid	Phenolic acids	1.08093	0.00172	kz0000419	Phlorizin	Chalcones	1.09234	0.00094
kz00297	Pratensein 7-O-glucopyranoside	Flavones	1.0740	0.00142	kz00459	1,4-di-O-galloyl-β-D-glcose	Phenolic acids	1.0648	0.08697	kz00337	Swertiamarin	Terpenoids	1.0815	0.00185	kz00055	Neochlorogenic acid(5-O-Caffeoylquinic acid)	Phenolic acids	1.0922	0.00096

8			2	
kz000186	2-Deoxyribose 1-phosphate	Others	1.07393	0.00143
kz003823	Kampferol 3-O-(2″-galloyl)-β-D-galactopyranoside	Flavonols	1.07387	0.00150
kz000555	1-Caffeoylquinic acid	Phenolic acids	1.07389	0.00154
km0818	Myricetin 3-O-rhamnoside (Myricitrin)	Flavonols	1.07385	0.00156
kz003716	Maplexin D (2,4-Di-O-Galloyl-1,5-Anhydro-D-Glucitol)	Phenolic acids	1.07318	0.00156
kz001824	cinnamoyltartaric acid	Phenolic acids	1.07388	0.00168
kz003821	Kampferol 3-O-(6″-galloyl)-β-D-galactopyranoside	Flavonols	1.07395	0.00172
kz003794	Syringic Aldehyde-glucoside	Phenolic acids	1.07306	0.00182
kz003636	Plantainoside A	Phenolic acids	1.07359	0.00185
kz000592	Rosmarinyl Glucoside	Phenolic acids	1.07294	0.00188
kz0	Procyanidin A1	Proant	1.	0.00

8			9	
kz002678	Quercetin-3-O-(6''-O-acetyl)-galactoside	Flavonols	1.07121	0.09343
kz002990	Luteolin-7-O-rutinoside	Flavones	1.06490	0.09770
kz000905	Kaempferol-3-O-rutinoside(Nicotiflorin)	Flavonols	1.07045	0.10020
kz000906	Kaempferol-3-O-robinobioside(Biorobin)	Flavonols	1.07070	0.10134
kz005225	Luteolin 7-O-neohesperidoside(Lonicerin)	Flavones	1.06545	0.10190
kz004171	Kaempferol 3-O-β-D-neohesperidoside	Flavonols	1.06595	0.10541
kz000872	Myricetin	Flavonols	1.06845	0.11547
kz000580	1,6-Di-O-Galloyl-D-Glucose	Phenolic acids	1.06483	0.11953
kz003850	(-)-secoisolariciresinol 4-O-β-D-giucopyranoside	Lignans	1.07040	0.12586
kz001020	4-Methyl-5-thiazoleethanol	Others	1.07078	0.13333
kz0	(2R)-Pinocembrin-7-	Flavan	1.	0.18

0			1	
kz005049	Salireposide	Phenolic acids	1.08164	0.00185
kz000517	Sinapyl alcohol	Phenolic acids	1.08152	0.00207
kz002367	Methyl dioxindole-3-acetate	Others	1.08168	0.00316
kz003636	Plantainoside A	Phenolic acids	1.08161	0.00342
kz001864	vnilloylcaffeoyltartaric acid	Phenolic acids	1.08114	0.00380
kz004774	(2R)-Pinocembrin-7-neohesperidoside	Flavanones	1.08190	0.00396
kz002936	Silibinin	Flavanols	1.08177	0.00407
km0818	Myricetin 3-O-rhamnoside (Myricitrin)	Flavonols	1.08124	0.00467
kz000971	Catechin	Flavanols	1.07964	0.00476
kz001331	Procyanidin A1	Proanthocyanidins	1.07814	0.00564
kz0	Neochlorogenic acid(5-O-	Pheno	1.	0.00

3			9	
kz000050	Homogentisic acid	Phenolic acids	1.09253	0.00102
kz000612	1,3,4,6-Tetra-O-Galloyl-D-Glucose	Phenolic acids	1.09096	0.00103
kz000207	Cannabiscitrin	Others	1.09241	0.00105
kz003636	Plantainoside A	Phenolic acids	1.09233	0.00106
kz002557	4-O-glucosyl-3,4-dihydroxybenzyl alcohol	Phenolic acids	1.09248	0.00110
kz005377	lyoniresinol-9'-O-β-D-xylopyranoside	Lignans	1.09245	0.00115
kz003716	Maplexin D (2,4-Di-O-Galloyl-1,5-Anhydro-D-Glucitol)	Phenolic acids	1.09039	0.00125
kz000555	1-Caffeoylquinic acid	Phenolic acids	1.09252	0.00128
kz003147	Clemaphenol A	Lignans	1.09224	0.00131
kz001864	vnilloylcaffeoyltartaric acid	Phenolic acids	1.09227	0.00150
kz0	Procyanidin C2	Proant	1.	0.00

01331		hocyanidins	07399	197
kz000156	7-O-Methyleriodictyol	Flavanols	1.07386	0.00204
kz000306	Methyl jasmonate	Organic acids	1.07401	0.00228
kz002212	Azalein (Azaleatin-3-O-rhamnoside)	Flavonols	1.07353	0.00261
kz000276	4-Aminoindole	Alkaloids	1.07299	0.00276
kz001340	Procyanidin C2	Proanthocyanidins	1.07349	0.00287
kz000612	1,3,4,6-Tetra-O-Galloyl-D-Glucose	Phenolic acids	1.07335	0.00321
kz001726	Prunellin A	Others	1.07278	0.00370
kz000974	Gallocatechin	Flavanols	1.07327	0.00456
kz002367	Methyl dioxindole-3-acetate	Others	1.07273	0.00491
kz00255	4-O-glucosyl-3,4-dihydroxybenzyl alcohol	Phenolic acids	1.0738	0.00522
04774	neohesperidoside	ones	04546	042
kz004260	Corosolic acid	Terpenoids	1.06967	0.19229
kz000479	Cinnamic acid	Phenolic acids	1.06824	0.19374
kz001163	Riboflavin	Vitamins	1.07015	0.20868
kz005055	Galangin-7-glucoside	Flavones	1.06407	0.23417
kz002604	Kaempferol-3-O-(6''-acetyl)-glucoside	Flavonols	1.06815	0.23570
kz000792	Apigenin 5-O-glucoside	Flavones	1.06298	0.23867
km060602	Genistein 7-O-Glucoside (Genistin)	Isoflavones	1.06728	0.23878
kz002788	Sexangularetin 3-glucoside-7-rhamnoside	Flavonols	1.02976	0.24926
kz000066	Feruloyl syringic acid	Phenolic acids	1.03108	0.27028
kz000091	Isorhamnetin-3-O-rutinoside (Narcissin)	Flavonols	1.0683	0.27205
00553	Caffeoylquinic acid)	lic acids	08073	658
kz000419	Phlorizin	Chalcones	1.07969	0.00760
kz003147	Clemaphenol A	Lignans	1.08188	0.00772
kz003812	Isorhamnetin O-malonylglucoside	Flavonols	1.07985	0.00932
kz000969	Afzelechin(3,5,7,4'-Tetrahydroxyflavan)	Flavanols	1.07798	0.00943
kz003098	3-O-Digalloyl quinic acid	Phenolic acids	1.08173	0.00959
kz003821	Kampferol 3-O-(6″-galloyl)-β-D-galactopyranoside	Flavonols	1.07885	0.01020
km0436	Afzelechin (3,5,7,4'-Tetrahydroxyflavan)	Flavanones	1.08124	0.01166
kz004771	Epicatechin glucoside	Flavanols	1.08002	0.01996
kz003362	Tubuloside C	Phenolic acids	1.08164	0.02602
kz00520	Dihydrokaempferol	Flavanols	1.0802	0.03404
01340		hocyanidins	09183	150
kz002367	Methyl dioxindole-3-acetate	Others	1.09237	0.00189
kz003370	Swertiamarin	Terpenoids	1.09243	0.00192
kz002551	Isosinapic acid-hexoside	Phenolic acids	1.09191	0.00227
kz003083	5,2'-Dihydroxy-7,8-dimethoxyflavone glycosides	Flavones	1.09233	0.00244
kz001331	Procyanidin A1	Proanthocyanidins	1.09215	0.00249
kz001680	6-hydroxy-5,7,4'-trimethoxyflavone	Flavones	1.09246	0.00283
kz001584	Quercetin-3′,4′-dimethyl ether	Flavonols	1.09243	0.00302
kz000552	4-O-Caffeoyl quinic acid (criptochlorogenic acid)	Phenolic acids	1.09222	0.00319
kz000971	Catechin	Flavanols	1.09031	0.00445
kz00296	Eupatilin	Flavones	1.0923	0.00499

7			8	
kz002668	Ovalifoliolides B	Terpenoids	1.07399	0.00597
kz000973	Epigallocatechin (EGC)	Flavanols	1.07309	0.00608
kz000419	Phlorizin	Chalcones	1.07289	0.00617
kz005376	5'-methoxyisolariciresinol-9'-O-β-D-xylopyranoside	Lignans	1.07295	0.00624
kz003370	Swertiamarin	Terpenoids	1.07214	0.00627
kz003809	Sieboldin	Chalcones	1.07185	0.00741
kz004665	5,7,3',4',5’-pentahydroxydihydroflavone	Flavanones	1.07327	0.00760
kz000552	4-O-Caffeoyl quinic acid (criptochlorogenic acid)	Phenolic acids	1.06558	0.00774
kz003106	Cuspinin	Phenolic acids	1.07291	0.00787
kz002960	Eupatilin	Flavones	1.07375	0.00806
kz0	Caffeoylagmatine	Alkaloi	1.	0.00

2			1	
kz002196	2'-Hydoxy,5-methoxy Genistein-O-rhamnosyl-glucoside	Isoflavones	1.03423	0.27416
kz000557	Glucosyringic Acid	Phenolic acids	1.07058	0.27987
kz000540	3,4,5-Trimethoxyphenyl-β-D-Glucopyranoside	Phenolic acids	1.07018	0.28576
kz000797	Tetahydroxy-flavone-7-O-β-D-glucuronide	Flavones	1.06955	0.28735
kz001340	Procyanidin C2	Proanthocyanidins	1.04500	0.29284
kz000791	Apigenin 7-O-glucoside(Cosmosiin)	Flavones	1.05024	0.29371
kz002976	Scutellarin(Scutellarein-7-O-glucuronide)	Flavones	1.06621	0.29482
kz001113	Lumichrome	Alkaloids	1.06352	0.30343
kz000296	Citramalate	Organic acids	1.06341	0.30467
kz000798	Luteolin-7-O-glucuronide	Flavones	1.06979	0.30871
kz0	Kaempferol-3-O-β-D-	Flavon	1.	0.31

2			8	
kz003850	(-)-secoisolariciresinol 4-O-β-D-giucopyranoside	Lignans	1.08154	0.03856
kz004425	Luteolin-O-rutinoside-O-rhamnoside	Flavones	1.07455	0.04135
kz001020	4-Methyl-5-thiazoleethanol	Others	1.08170	0.04757
kz000557	Glucosyringic Acid	Phenolic acids	1.08006	0.04779
kz000919	Robinin(Kaempferol-3-O-gal-rham-7-O-rham)	Flavonols	1.07293	0.04812
kz002912	Santamarin	Terpenoids	1.07915	0.04907
km0548	Dihydroquercetin (Taxifolin)	Flavonols	1.08054	0.05264
kz002560	6-O-feruloyl-α-glucose	Phenolic acids	1.07672	0.05527
kz003519	Cimidahurinine	Phenolic acids	1.08093	0.05670
kz000058	Protocatechuic acid O-glucoside	Phenolic acids	1.07895	0.05954
kz0	1,6-Di-O-Galloyl-D-Glucose	Pheno	1.	0.06

0			6	
kz004768	monogalloyl-diglucose	Phenolic acids	1.09222	0.00507
kz000575	3,5-Di-O-galloylshikimic acid	Phenolic acids	1.09213	0.00559
kz003106	Cuspinin	Phenolic acids	1.08812	0.00573
kz000872	Myricetin	Flavonols	1.09233	0.00654
kz000969	Afzelechin(3,5,7,4'-Tetrahydroxyflavan)	Flavanols	1.08878	0.00666
kz004665	5,7,3',4',5’-pentahydroxydihydroflavone	Flavanones	1.09047	0.00722
kz002962	Penduletin (5,4'-Dihydroxy-3,6,7-trimethoxyflavone)	Flavones	1.09229	0.00746
kz001695	wogonoside	Flavones	1.09209	0.00759
km0436	Afzelechin (3,5,7,4'-Tetrahydroxyflavan)	Flavanones	1.09200	0.00791
kz002975	Acacetin-7-O-glucuronide	Flavones	1.09211	0.00828
kz0	Quercetin-3-O-(2''-	Flavon	1.	0.01

00032		ds	07273	833
kz004231	Isothankunic acid	Terpenoids	1.07289	0.00991
kz002962	Penduletin (5,4'-Dihydroxy-3,6,7-trimethoxyflavone)	Flavones	1.07392	0.01016
kz003147	Clemaphenol A	Lignans	1.06898	0.01457
kz001584	Quercetin-3′,4′-dimethyl ether	Flavonols	1.07193	0.01563
kz001695	wogonoside	Flavones	1.07374	0.01604
kz000971	Catechin	Flavanols	1.06992	0.01826
kz004703	secoisolariciresinol 9-O-β-D-glucopyranoside	Lignans	1.06758	0.01826
kz000304	(±)-jasmonic acid	Organic acids	1.07393	0.01975
kz000440	Astilbin	Flavanols	1.07087	0.01992
kz00477	Epicatechin glucoside	Flavanols	1.0721	0.02396
01702	glucuronide	ols	06742	004
kz000078	1'-O-β-D-(3,4-Dihydroxyphenethyl)-O-caffeoyl-glucoside	Phenolic acids	1.06211	0.31894
km0936	Quercetin 7-O-β-D-Glucuronide	Flavonols	1.06883	0.32472
kz003151	Ailantinol E	Others	1.06810	0.35089
kz000592	Rosmarinyl Glucoside	Phenolic acids	1.05922	0.35104
kz005202	Dihydrokaempferol	Flavanols	1.06832	0.37398
kz002862	Dihydrosedinine	Alkaloids	1.06749	0.37848
kz005247	Limonin	Others	1.06257	0.38022
kz000291	2-Picolinic acid	Organic acids	1.06764	0.38437
kz001188	Esculin(6,7-DihydroxyCoumarin-6-glucoside)	Coumarins	1.05084	0.39682
kz00309	Di-O-Glucose-quinic acid	Phenolic acids	1.0631	0.39689
00580		lic acids	06486	154
kz003109	Mearnsitrin	Flavones	1.07931	0.06464
kz004598	1,4-di-O-galloyl-β-D-glcose	Phenolic acids	1.07986	0.06499
kz002788	Sexangularetin 3-glucoside-7-rhamnoside	Flavonols	1.06962	0.06570
kz005373	Lyoniresinol	Lignans	1.06684	0.06606
kz003518	5-(2-Hydroxyethyl)-2-O-glucosylohenol	Phenolic acids	1.07470	0.07080
kz004174	Benzyl-O-β-D-glucopyranose-β-D-xyranoside	Others	1.01867	0.07264
kz001581	Calceorioside B	Others	1.07403	0.07287
kz004170	Rhamnetin 3-O-β-D-Glucoside	Flavonols	1.08138	0.07424
kz005088	Tricetin?4'-methyl?ether-3'-O-β-D-glucoside	Flavonols	1.07866	0.07437
km0602	Genistein 7-O-Glucoside (Genistin)	Isoflavones	1.0708	0.07556
01739	galloyl)-β-D-glucoside	ols	08710	140
kz003829	Quercetin 3-O-(6′′-galloyl)-β-D-galactopyranoside	Flavonols	1.09016	0.01150
kz001191	4-hydroxycoumarin di-glucoside	Coumarins	1.09039	0.01278
kz001076	Ethyl gallate	Phenolic acids	1.09226	0.01310
kz003812	Isorhamnetin O-malonylglucoside	Flavonols	1.09195	0.01410
kz000440	Astilbin	Flavanols	1.08947	0.01976
kz003748	7'-O-Sinapoyljasminoside L	Terpenoids	1.08935	0.02075
kz000156	7-O-Methyleriodictyol	Flavanols	1.09043	0.02083
kz004774	(2R)-Pinocembrin-7-neohesperidoside	Flavanones	1.08772	0.02193
kz002668	Ovalifoliolides B	Terpenoids	1.08687	0.02247
kz00297	Pratensein 7-O-glucopyranoside	Flavones	1.0917	0.02251

1			6	
kz000969	Afzelechin(3,5,7,4'-Tetrahydroxyflavan)	Flavanols	1.06766	0.02414
km0436	Afzelechin (3,5,7,4'-Tetrahydroxyflavan)	Flavanones	1.07282	0.02696
kz002910	Parthenolide	Terpenoids	1.07314	0.02743
kz005226	Neodiosmin (Diosmetin-7-O-Neohesperidoside)	Flavones	1.07284	0.02841
kz003848	Cinchonain Ic	Flavanols	1.07190	0.02915
kz000831	Diosmin	Flavones	1.07343	0.02979
kz000979	Catechin-(7,8-bc)-4α-(3,4-dihydroxyphenyl)-dihydro-2-(3H)-pyranone	Flavanols	1.07225	0.03106
kz003540	Dihydrokaempferol-7-O-glucoside	Flavonols	1.07289	0.03135
kz002673	Eriodictyol-O-glucoside	Flavones	1.07269	0.03224
kz000978	Catechin-(7,8-bc)-4β-(3,4-dihydroxyphenyl)-dihydro-2-(3H)-pyranone	Flavanols	1.07367	0.03278
kz0	Dunalianoside B	Pheno	1.	0.03

9			7	
kz002560	6-O-feruloyl-α-glucose	Phenolic acids	1.05973	0.43429
kz000577	Hexahydroxydiphenoylglucose	Phenolic acids	1.06808	0.44099
kz000274	N-sinapoylhydroxycoumarin	Coumarins	1.03171	0.49631
kz000290	Aminomalonic acid	Organic acids	1.05749	2.01965
kz002554	p-Hydroxycinnamic acid	Phenolic acids	1.05192	2.03115
kz000049	2-(Formylamino)benzoic acid	Phenolic acids	1.06927	2.03683
kz000795	Luteolin-7-O-glucoside(Cynaroside)	Flavones	1.05986	2.03825
kz000491	p-Coumaric acid	Phenolic acids	1.05288	2.04320
kz000884	Kaempferol-3-O-glucoside (Astragalin)	Flavonols	1.05788	2.04671
kz000051	4-Methoxycinnamic acid	Phenolic acids	1.06846	2.06000
kz0	Ditartaroyl-hydroxylcoumarin	Pheno	1.	2.07

			2	
kz000792	Apigenin 5-O-glucoside	Flavones	1.08113	0.07685
kz002982	6-methoxykaempferol-3-O-glucoside	Flavonols	1.07963	0.07912
kz000147	Methylquercetin O-hexoside	Flavonols	1.07926	0.07949
km1307	Isorhamnetin 5-O-hexoside	Flavonols	1.08123	0.07970
kz005055	Galangin-7-glucoside	Flavones	1.08040	0.08005
kz004169	Isorhamnetin 3-O-β-D-Glucoside	Flavonols	1.07639	0.08096
kz002829	Isorhamnetin-7-O-glucoside	Flavonols	1.07937	0.08223
kz002196	2'-Hydoxy,5-methoxy Genistein-O-rhamnosyl-glucoside	Isoflavones	1.06825	0.08419
kz000912	Isorhamnetin-3-O-rutinoside (Narcissin)	Flavonols	1.08191	0.08827
kz000540	3,4,5-Trimethoxyphenyl-β-D-Glucopyranoside	Phenolic acids	1.08157	0.09345
kz0	Apigenin 7-O-	Flavon	1.	0.10

8			9	
kz002673	Eriodictyol-O-glucoside	Flavones	1.09141	0.02780
kz002485	3-methoxy-juglone	Quinones	1.07474	0.02798
kz003540	Dihydrokaempferol-7-O-glucoside	Flavonols	1.09205	0.02968
kz005187	Dunalianoside B	Phenolic acids	1.09116	0.03007
kz001074	Gallic acid	Phenolic acids	1.09204	0.03041
kz005186	Dunalianoside C	Phenolic acids	1.09171	0.03277
kz004738	4,7,9,9′-Tetrahydroxy-3,3′-dimethoxy-8-O-4′-neolignan	Others	1.08984	0.03320
kz000431	Eriodictyol 7-O-glucoside	Flavanones	1.08778	0.03561
kz004771	Epicatechin glucoside	Flavanols	1.08853	0.03681
kz000052	Ethyl 3,4-Dihydroxybenzoate (Ethyl protocatechuate)	Phenolic acids	1.08940	0.03772
kz0	Catechin gallate	Pheno	1.	0.03

05187		lic acids	07229	306
kz0003083	5,2'-Dihydroxy-7,8-dimethoxyflavone glycosides	Flavones	1.07214	0.03356
kz0000830	Chrysoeriol-7-O-rutinoside	Flavones	1.07298	0.03487
kz0003812	Isorhamnetin O-malonylglucoside	Flavonols	1.07273	0.03493
kz0003519	Cimidahurinine	Phenolic acids	1.07316	0.03954
kz0000058	Protocatechuic acid O-glucoside	Phenolic acids	1.07166	0.04346
kz0003518	5-(2-Hydroxyethyl)-2-O-glucosylohenol	Phenolic acids	1.06842	0.05039
kz0000308	N-[(-)-Jasmonoyl]-(L)-Isoleucine (JA-L-Ile)	Organic acids	1.07384	0.06130
kz0003829	Quercetin 3-O-(6′′-galloyl)-β-D-galactopyranoside	Flavonols	1.06786	0.06466
kz0001739	Quercetin-3-O-(2''-galloyl)-β-D-glucoside	Flavonols	1.05814	0.06657
kz00005	3-O-p-Coumaroylshikimic acid	Phenolic acids	1.0666	0.06667
00567		lic acids	06005	119
kz0000974	Gallocatechin	Flavanols	1.06499	2.07510
kz0000889	Spiraeoside	Flavonols	1.06946	2.12548
kz0002558	6-O-caffeoyl-β-glucose	Phenolic acids	1.06306	2.13245
kz0001156	Pyridoxine	Vitamins	1.04371	2.14730
kz0003690	Quercetin-3-O-α-L-rhamnopyranoside	Flavonols	1.05889	2.14768
kz0000500	Riboprine	Phenolic acids	1.06537	2.15375
kz0005078	Kaempferol-7-O-β-D-glucopyranoside	Flavonols	1.03427	2.16486
km0779	Luteolin 7-O-glucoside	Flavones	1.05556	2.16808
kz0000800	Chrysoeriol-5-O-hexoside	Flavones	1.06893	2.19780
kz00159	Stachydrine	Alkaloids	1.0560	2.22126
00791	glucoside(Cosmosiin)	es	07529	059
kz0002678	Quercetin-3-O-(6''-O-acetyl)-galactoside	Flavonols	1.04885	0.10142
kz0002485	3-methoxy-juglone	Quinones	1.08073	0.10186
kz0003151	Ailantinol E	Others	1.08013	0.10848
km1405	Nicotinic acid-hexoside	Vitamins	1.07613	0.11409
kz0001100	Nicotinic Acid Methyl Ester(Methyl Nicotinate)	Alkaloids	1.08115	0.11518
kz0000190	Arbutin	Others	1.07726	0.13062
kz0000040	N,N'-Bis(Sinapoyl)Spermidine	Alkaloids	1.04422	0.13160
km0936	Quercetin 7-O-β-D-Glucuronide	Flavonols	1.08139	0.13981
kz0003797	3-Prenyl-4-O-β-D-glucopyranosyloxy-4-hydroxyl-benzoic acid	Phenolic acids	1.06814	0.14093
kz00007	1'-O-β-D-(3,4-Dihydroxyphenethyl)-O-caffeoyl-glucoside	Phenolic acids	1.0806	0.14480
00068		lic acids	08960	893
kz0000424	Eriodictyol	Flavanones	1.09186	0.04056
km0573	Epicatechin gallate (ECG)	Flavanols	1.08657	0.04352
kz0005370	(+)-Isolariciresinol	Lignans	1.09186	0.04370
kz004174	Benzyl-O-β-D-glucopyranose-β-D-xyranoside	Others	1.04309	0.04729
km0548	Dihydroquercetin (Taxifolin)	Flavonols	1.09106	0.04890
kz0003848	Cinchonain Ic	Flavanols	1.08902	0.05050
kz0000417	Phloretin	Chalcones	1.09028	0.05061
kz0000040	N,N'-Bis(Sinapoyl)Spermidine	Alkaloids	1.06481	0.05368
kz0000584	5-O-p-Coumaroyl quinic acid O-hexoside	Phenolic acids	1.06862	0.05660
kz00293	Silibinin	Flavanols	1.0919	0.05781

9			4	
kz000479	Cinnamic acid	Phenolic acids	1.07308	0.07382
kz003634	Sinapaldehyde Glucoside	Phenolic acids	1.06855	0.07906
kz005389	Byzantionoside A	Others	1.06913	0.08903
kz001086	2,3-Dihydroxy 5(6),12(13)diene ursolic acid	Terpenoids	1.07279	0.09914
kz000040	N,N'-Bis(Sinapoyl)Spermidine	Alkaloids	1.05276	0.10245
kz000881	Avicularin	Flavonols	1.07087	0.13828
kz000584	5-O-p-Coumaroyl quinic acid O-hexoside	Phenolic acids	1.02934	0.14321
kz003719	Methyl 4,6-di-O-galloyl-D-glucoside	Phenolic acids	1.03236	0.14540
kz001100	Nicotinic Acid Methyl Ester(Methyl Nicotinate)	Alkaloids	1.07225	0.14586
kz002211	Quercetin 3-O-β-D-xylopyranoside	Flavonols	1.07018	0.15869
kz0	4-Methyl-5-thiazoleethanol	Other	1.	0.16
8			6	
kz004697	Taxifolin-3'-O-β-D-glucoside	Flavones	1.06420	2.22673
kz002807	Dimethylmalonic acid	Organic acids	1.01996	2.23954
kz000441	Hesperetin 5-O-glucoside	Flavanols	1.06838	2.24311
kz005137	Isoscopoletin (6-Hydroxy-7-Methoxycoumarin)	Coumarins	1.05048	2.26782
kz002995	Quercetin-7-O-(6'-O-malonyl)-β-D-glucoside	Flavonols	1.02214	2.30000
kz001154	Nicotinamide	Vitamins	1.06878	2.30028
kz000550	Coniferin	Phenolic acids	1.06899	2.30891
kz004013	Solatuberenol A	Others	1.06720	2.31033
kz002214	isohyperoside	Flavonols	1.06752	2.36242
kz000549	1-O-[(E)-Caffeoyl]-β-D-glucopyranose	Phenolic acids	1.06040	2.37167
kz0	Quercetin-3-O-β-D-Galactoside	Flavon	1.	2.37
8			0	
kz001318	Quinic Acid	Organic acids	1.08063	0.14949
kz002604	Kaempferol-3-O-(6''-acetyl)-glucoside	Flavonols	1.07328	0.15180
kz000056	Salicylic acid glucoside	Phenolic acids	1.08165	0.15377
kz002910	Parthenolide	Terpenoids	1.07784	0.15915
kz002295	Syringaresinol-4'-O-β-D-monO-glucoside	Lignans	1.06564	0.16336
kz000412	Aucubin	Terpenoids	1.07864	0.16342
kz002546	Isosalicylic acid O-glycoside	Phenolic acids	1.07639	0.16357
kz005370	(+)-Isolariciresinol	Lignans	1.07963	0.16836
kz001158	D-Pantothenic Acid	Vitamins	1.05808	0.17754
kz002556	4-O-glucosyl-4-hydroxybenzoic acid	Phenolic acids	1.07199	0.18463
kz0	Citramalate	Organi	1.	0.18
6			1	
km0080	Biochanin A	Isoflavones	1.09034	0.06083
kz003796	1'-O-vanilloyl-β-D-glucoside	Phenolic acids	1.08999	0.06111
kz000560	Trihydroxycinnamoylquinic acid	Phenolic acids	1.09145	0.06239
kz000979	Catechin-(7,8-bc)-4α-(3,4-dihydroxyphenyl)-dihydro-2-(3H)-pyranone	Flavanols	1.09026	0.06240
kz001193	Prunetin	Isoflavones	1.08826	0.06411
kz005373	Lyoniresinol	Lignans	1.07714	0.06449
kz000978	Catechin-(7,8-bc)-4β-(3,4-dihydroxyphenyl)-dihydro-2-(3H)-pyranone	Flavanols	1.09170	0.06978
kz001581	Calceorioside B	Others	1.08315	0.07248
kz003519	Cimidahurinine	Phenolic acids	1.08801	0.07347
kz003109	Mearnsitrin	Flavones	1.08941	0.07352
kz0	Protocatechuic acid O-	Pheno	1.	0.07

01020		s	07274	281
kz0 05186	Dunalianoside C	Phenolic acids	1.06901	0.17124
kz0 00118	Sakuranetin	Flavones	1.07182	0.17850
kz0 01261	γ-Aminobutyric acid	Organic acids	1.05266	0.18110
kz0 00431	Eriodictyol 7-O-glucoside	Flavanones	1.04930	0.18671
kz0 03099	Di-O-Glucose-quinic acid	Phenolic acids	1.07332	0.19591
kz0 03854	Roseoside	Others	1.07158	0.20754
kz0 04013	Solatuberenol A	Others	1.04495	0.21612
kz0 00554	Chlorogenic acid	Phenolic acids	1.07072	0.23551
kz0 01581	Calceorioside B	Others	1.04958	0.23636
kz0 0241	Ergotamine	Alkaloids	1.0719	0.24006

00888	(Hyperin)	ols	06340	374
kz0 00892	6-Hydroxykaempferol-7-O-glucoside	Flavonols	1.06308	2.37420
km 0228	Quercetin 3-O-glucoside (Isotrifoliin)	Flavonols	1.06811	2.41332
kz0 03108	mlyricetin 3-O-B-D-glucopyranoside	Flavonols	1.05542	2.42409
kz0 00517	Sinapyl alcohol	Phenolic acids	1.04338	2.43020
kz0 04626	pomolic acid	Terpenoids	1.06984	2.45103
kz0 01671	5,7,2'-Trhiyroxy-8-methoxyflavone	Flavones	1.05376	2.46787
kz0 05385	Machilusolide D	Others	1.06099	2.47420
km 1437	p-Aminobenzoate	Phenolic acids	1.06874	2.50761
km 0071	Quercetin 3-O-galactoside (Hyperin)	Flavonols	1.06890	2.51332
kz0 0108	Maslinic acid	Terpenoids	1.0694	2.51475

00296		c acids	06871	847
kz0 04231	Isothankunic acid	Terpenoids	1.07192	0.19298
kz0 00066	Feruloyl syringic acid	Phenolic acids	1.07014	0.19618
kz0 00068	Catechin gallate	Phenolic acids	1.07214	0.19793
kz0 05225	Luteolin 7-O-neohesperidoside(Lonicerin)	Flavones	1.06639	0.20045
km 0573	Epicatechin gallate (ECG)	Flavanols	1.05298	0.20534
kz0 04171	Kaempferol 3-O-β-D-neohesperidoside	Flavonols	1.07908	0.21176
kz0 03099	Di-O-Glucose-quinic acid	Phenolic acids	1.08089	0.21547
kz0 03517	Glucosyloxybenzoic acid	Phenolic acids	1.07923	0.21615
kz0 02995	Quercetin-7-O-(6'-O-malonyl)-β-D-glucoside	Flavonols	1.05745	0.22241
kz0 0090	Kaempferol-3-O-robinobioside(Biorobin)	Flavonols	1.0789	0.22911

00058	glucoside	lic acids	08853	458
kz0 04630	2α-hydroxyursolic acid	Terpenoids	1.09222	0.07649
kz0 05088	Tricetin?4'-methyl?ether-3'-O-β-D-glucoside	Flavonols	1.08786	0.07652
kz0 04170	Rhamnetin 3-O-β-D-Glucoside	Flavonols	1.09067	0.07665
kz0 03728	Geniposide	Terpenoids	1.08274	0.07788
km 1405	Nicotinic acid-hexoside	Vitamins	1.08852	0.07827
km 1307	Isorhamnetin 5-O-hexoside	Flavonols	1.09176	0.07837
kz0 02982	6-methoxykaempferol-3-O-glucoside	Flavonols	1.08991	0.07856
kz0 00147	Methylquercetin O-hexoside	Flavonols	1.08936	0.07979
kz0 04169	Isorhamnetin 3-O-β-D-Glucoside	Flavonols	1.08731	0.08081
kz0 0091	6-Hydroxykaempferol-3,6-O-Diglucoside	Flavonols	1.0885	0.08083

9			4	
kz003748	7'-O-Sinapoyljasminoside L	Terpenoids	1.06065	0.24629
kz004169	Isorhamnetin 3-O-β-D-Glucoside	Flavonols	1.05538	0.24942
kz005088	Tricetin?4'-methyl?ether-3'-O-β-D-glucoside	Flavonols	1.06095	0.25693
kz004170	Rhamnetin 3-O-β-D-Glucoside	Flavonols	1.07252	0.25955
kz000548	Trans-3-O-p-coumaric quinic acid	Phenolic acids	1.06618	0.25995
kz002853	Dihydroisopelletierine	Alkaloids	1.07308	0.26160
kz000560	Trihydroxycinnamoylquinic acid	Phenolic acids	1.06835	0.26399
kz002829	Isorhamnetin-7-O-glucoside	Flavonols	1.07008	0.26873
kz004738	4,7,9,9'-Tetrahydroxy-3,3'-dimethoxy-8-O-4'-neolignan	Others	1.05026	0.27100
kz000147	Methylquercetin O-hexoside	Flavonols	1.06374	0.27129
kz0	6-methoxykaempferol-3-O-	Flavon	1.	0.27

7			6	
kz000484	Vanillin	Phenolic acids	1.06939	2.51658
km0081	Genistein (4',5,7-Trihydroxyisoflavone)	Isoflavones	1.03996	2.51868
kz002933	Ixerin D	Terpenoids	1.04122	2.53411
kz001864	vnilloylcaffeoyltartaric acid	Phenolic acids	1.03829	2.53707
kz003815	Kaempferol 3-O-(6''-trans-p-Coumaroyl)-β-D-glucopyranoside	Flavonols	1.04499	2.53941
kz001089	2-Hydroxyoleanolic acid	Terpenoids	1.06818	2.54107
kz000240	Trans-Zeatin-9-N-Glucoside	Alkaloids	1.05985	2.54890
kz004627	alphitolic acid	Terpenoids	1.06902	2.55738
kz001297	(Rs)-Mevalonic acid	Organic acids	1.05236	2.56548
kz001088	3,24-Dihydroxy-17,21-semiacetal-12(13)oleanolic fruit	Terpenoids	1.06836	2.58065
kz0	2-Methylsuccinic acid	Organi	1.	2.59

6			0	
kz000522	Methyl sinapate	Phenolic acids	1.05604	0.23465
kz000596	β-D-Furanofructosyl-α-D-(6-mustard acyl)glucoside	Phenolic acids	1.06442	0.23567
kz000905	Kaempferol-3-O-rutinoside(Nicotiflorin)	Flavonols	1.08041	0.24010
kz002976	Scutellarin(Scutellarein-7-O-glucuronide)	Flavones	1.07873	0.26272
kz003811	Kaempferol-malonyl-3-O-glucoside	Flavonols	1.06351	0.26371
kz005186	Dunalianoside C	Phenolic acids	1.07383	0.26398
kz002486	1,4,8-trihydroxy naphthalene-1-O-β-D-glucopyranoside	Quinones	1.05776	0.26486
kz002990	Luteolin-7-O-rutinoside	Flavones	1.06826	0.26574
kz001702	Kaempferol-3-O-β-D-glucuronide	Flavonols	1.07877	0.27004
kz000798	Luteolin-7-O-glucuronide	Flavones	1.08068	0.27298
kz0	Tetahydroxy-flavone-7-O-β-D-	Flavon	1.	0.27

4			4	
kz002829	Isorhamnetin-7-O-glucoside	Flavonols	1.09068	0.08147
kz002337	Luteolin-7-O-β-D-gentiobioside	Flavones	1.08587	0.08217
kz004035	3-Indoleacrylic acid	Alkaloids	1.02579	0.08678
kz002295	Syringaresinol-4'-O-β-D-monO-glucoside	Lignans	1.08601	0.08773
kz005202	Dihydrokaempferol	Flavanols	1.08923	0.09103
kz003518	5-(2-Hydroxyethyl)-2-O-glucosylohenol	Phenolic acids	1.08130	0.09542
kz002995	Quercetin-7-O-(6'-O-malonyl)-β-D-glucoside	Flavonols	1.08492	0.09670
kz003714	6-O-Galloyl-β-D-glucose	Phenolic acids	1.09203	0.10246
kz003497	3-O-Galloyl-β-D-glucose	Phenolic acids	1.09206	0.10604
kz003100	5-O-Galloylhamamelose	Phenolic acids	1.09232	0.10879
kz0	Parthenolide	Terpe	1.	0.10

02982	glucoside	ols	06538	726
kz002977	Diosmetin-7-O-galactoside	Flavones	1.03563	0.27967
kz000232	O-Phosphocholine	Alkaloids	1.07208	0.28465
kz005373	Lyoniresinol	Lignans	1.00565	0.28521
km1307	Isorhamnetin 5-O-hexoside	Flavonols	1.06648	0.29444
kz005054	Chrysin-7-glucoside	Flavones	1.07009	0.29513
kz000522	Methyl sinapate	Phenolic acids	1.03105	0.29616
kz003109	Mearnsitrin	Flavones	1.05771	0.29632
kz002486	1,4,8-trihydroxy naphthalene-1-O-β-D-glucopyranoside	Quinones	1.06038	0.29763
kz003116	2,4,6-trihydroxy benzoic acid	Organic acids	1.05538	0.30000
kz00015	Naringenin 7-O-glucoside (Prunin)	Flavanols	1.0721	0.30281
01285		c acids	06357	091
kz003549	Hederagenin	Others	1.07073	2.59662
kz000779	Diosmetin	Flavones	1.06747	2.63377
kz002977	Diosmetin-7-O-galactoside	Flavones	1.05952	2.63654
km1211	Chrysoeriol 7-O-hexoside	Flavones	1.04421	2.64192
kz001157	4-Pyridoxic acid	Vitamins	1.06042	2.65940
kz000812	Tricin O-saccharic acid	Flavones	1.06893	2.70166
kz000278	Indole 3-acetic acid (IAA)	Alkaloids	1.04247	2.76863
kz000300	DL-Glyceraldehyde 3-phosphate	Organic acids	1.05967	2.78026
kz004021	6-Deoxyfagomine	Alkaloids	1.05729	2.79555
kz00126	2-Furanoic acid	Organic acids	1.0674	2.81628
00797	glucuronide	es	08053	448
kz000907	Kaempferol-3-O-glucoside-7-O-rhamnoside	Flavonols	1.05982	0.27753
kz004738	4,7,9,9′-Tetrahydroxy-3,3′-dimethoxy-8-O-4′-neolignan	Others	1.06282	0.27816
kz000979	Catechin-(7,8-bc)-4α-(3,4-dihydroxyphenyl)-dihydro-2-(3H)-pyranone	Flavanols	1.06866	0.28858
kz000291	2-Picolinic acid	Organic acids	1.08023	0.29034
kz000995	Syringaresinol	Lignans	1.07905	0.30456
kz000909	Quercetin-3-O-rutinoside (Rutin)	Flavonols	1.07713	0.30930
kz000240	Trans-Zeatin-9-N-Glucoside	Alkaloids	1.08001	0.31399
kz000978	Catechin-(7,8-bc)-4β-(3,4-dihydroxyphenyl)-dihydro-2-(3H)-pyranone	Flavanols	1.07882	0.31877
kz000284	3-Hydroxypropanoic acid	Organic acids	1.07252	0.32536
kz00044	Astilbin	Flavanols	1.0346	0.32634
02910		noids	08877	970
kz004231	Isothankunic acid	Terpenoids	1.08738	0.11855
kz003720	Maplexin H	Phenolic acids	1.08694	0.11930
kz001298	L-(+)-Tartaric acid	Organic acids	1.09144	0.11931
kz002949	Hispidulin	Flavones	1.07975	0.12037
kz000881	Avicularin	Flavonols	1.09039	0.12135
kz000240	Trans-Zeatin-9-N-Glucoside	Alkaloids	1.09055	0.12318
kz001880	feruloylsinapoyltartaric acid	Phenolic acids	1.08437	0.12480
kz002560	6-O-feruloyl-α-glucose	Phenolic acids	1.08243	0.12725
kz003158	Ailanindole	Alkaloids	1.01025	0.13540
kz00505	Chrysin-7-glucoside	Flavones	1.0913	0.13856

9			5	
kz000792	Apigenin 5-O-glucoside	Flavones	1.06848	0.30979
kz000064	1-O-Feruloyl quinic acid	Phenolic acids	1.06213	0.31440
kz003766	Uncargenin D	Terpenoids	1.06914	0.31523
kz002788	Sexangularetin 3-glucoside-7-rhamnoside	Flavonols	1.03940	0.31557
kz005043	Scopoletin?Beta-D-Glucuronide	Coumarins	1.05732	0.32067
kz001177	Scoparone	Coumarins	1.06796	0.32115
kz000791	Apigenin 7-O-glucoside(Cosmosiin)	Flavones	1.04091	0.33885
kz002607	Lariciresinol glucopyranoside	Others	1.04802	0.34364
kz000997	Terpineol monO-glucoside	Lignans	1.06604	0.35384
kz000284	3-Hydroxypropanoic acid	Organic acids	1.06122	0.36151
kz0	Chrysin 5-O-glucoside (Toringin)	Flavon	1.	0.36

8			4	
kz001281	Citraconic acid	Organic acids	1.06184	2.83352
kz002853	Dihydroisopelletierine	Alkaloids	1.06586	2.84347
km0235	Apigenin	Flavones	1.05767	2.85484
kz000904	Tiliroside	Flavonols	1.06960	2.89256
kz001175	Esculetin	Coumarins	1.04583	2.93033
kz005198	Poncirin(Isosakuranetin-7-neohesperidoside)	Flavanones	1.07022	2.93444
kz001105	Acetylcholine	Alkaloids	1.06828	3.00000
kz001293	4-Guanidinobutyric acid	Organic acids	1.06958	3.00146
kz005132	Luteolin-caffeoyl-O-rhamnoside	Flavones	1.06987	3.00983
km0098	Orobol (5,7,3',4'-tetrahydroxyisoflavone)	Isoflavones	1.02259	3.11535
kz0	2,5-Dihydroxy benzoic acid O-	Pheno	1.	3.12

0			6	
kz000431	Eriodictyol 7-O-glucoside	Flavanones	1.04219	0.33034
kz004173	Quercetin 3-O-β-D-neohesperidoside	Flavonols	1.06950	0.33115
kz000560	Trihydroxycinnamoylquinic acid	Phenolic acids	1.07570	0.33157
kz001163	Riboflavin	Vitamins	1.07375	0.33409
kz001312	Shikimic acid	Organic acids	1.07556	0.34859
kz000908	Quercetin-3-O-robinobioside	Flavonols	1.07627	0.34891
kz003859	p-Coumaroyleuscaphic acid	Terpenoids	1.01130	0.35369
kz000911	Quercetin-7-O-rutinoside	Flavonols	1.07523	0.35455
kz003848	Cinchonain Ic	Flavanols	1.04995	0.35826
kz002673	Eriodictyol-O-glucoside	Flavones	1.06829	0.36091
kz0	Dihydrosedinine	Alkaloi	1.	0.36

4			6	
kz005389	Byzantionoside A	Others	1.08870	0.14220
kz000522	Methyl sinapate	Phenolic acids	1.07677	0.14529
km0239	Chrysoeriol	Flavones	1.08427	0.14778
kz002211	Quercetin 3-O-β-D-xylopyranoside	Flavonols	1.08647	0.14911
kz001158	D-Pantothenic Acid	Vitamins	1.07120	0.15000
kz000613	1,2,3,6-Tetra-O-Galloyl-D-Glucose	Phenolic acids	1.07739	0.15028
kz002977	Diosmetin-7-O-galactoside	Flavones	1.07244	0.15276
kz000159	Naringenin 7-O-glucoside (Prunin)	Flavanols	1.09182	0.15748
kz000121	Chrysin 5-O-glucoside (Toringin)	Flavones	1.09155	0.15829
kz000100	Nicotinic Acid Methyl Ester(Methyl Nicotinate)	Alkaloids	1.09088	0.15965
kz0	4-Acetamidobutyric acid	Organi	1.	0.16

001211		es	06882	543	00533	hexside	lic acids	04742	918	02862		ds	07142	305	01292		c acids	08808	016
kz005055	Galangin-7-glucoside	Flavones	1.03733	0.36604	km0231	2'-Hydroxygenistein	Isoflavones	1.00854	3.15574	kz005137	Isoscopoletin (6-Hydroxy-7-Methoxycoumarin)	Coumarins	1.05685	0.37559	kz003718	2,3-Di-O-Galloyl-D-Glucose	Phenolic acids	1.08731	0.16249
kz003720	Maplexin H	Phenolic acids	1.04178	0.37225	kz002783	Kaempferol 3-O-β-d-(6''-O-(E)-p-coumaroyl) glucopyranoside	Flavonols	1.06865	3.16172	kz003540	Dihydrokaempferol-7-O-glucoside	Flavonols	1.07080	0.38578	kz005137	Isoscopoletin (6-Hydroxy-7-Methoxycoumarin)	Coumarins	1.08728	0.16562
kz004632	isoceanothic acid	Terpenoids	1.07328	0.37273	kz003636	Plantainoside A	Phenolic acids	1.05915	3.21386	kz000505	Coniferyl alcohol	Phenolic acids	1.02072	0.39691	kz003815	Kaempferol 3-O-(6''-trans-p-Coumaroyl)-β-D-glucopyranoside	Flavonols	1.07506	0.16580
kz000068	Catechin gallate	Phenolic acids	1.01300	0.38255	kz004015	Cis-p-coumaric acid 4-O-glucoside	Others	1.06484	3.23737	kz002977	Diosmetin-7-O-galactoside	Flavones	1.00557	0.40275	kz000779	Diosmetin	Flavones	1.08615	0.16627
km0080	Biochanin A	Isoflavones	1.05323	0.38344	km0239	Chrysoeriol	Flavones	1.06846	3.25105	kz005187	Dunalianoside B	Phenolic acids	1.05681	0.41407	kz000557	Glucosyringic Acid	Phenolic acids	1.08702	0.17077
kz002196	2'-Hydoxy,5-methoxy Genistein-O-rhamnosyl-glucoside	Isoflavones	1.00839	0.39110	kz005084	(Kaempferol-3-O-β-D-galactoside-4'O-β-D-glucoside)	Flavonols	1.05234	3.25372	kz003815	Kaempferol 3-O-(6''-trans-p-Coumaroyl)-β-D-glucopyranoside	Flavonols	1.01942	0.42104	kz000596	β-D-Furanofructosyl-α-D-(6-mustard acyl)glucoside	Phenolic acids	1.08673	0.17089
kz000056	Salicylic acid glucoside	Phenolic acids	1.07220	0.39242	kz002548	p-Coumaric acid-O-glycoside	Phenolic acids	1.06867	3.27444	kz001671	5,7,2'-Trhiyroxy-8-methoxyflavone	Flavones	1.05251	0.42301	kz001671	5,7,2'-Trhiyroxy-8-methoxyflavone	Flavones	1.08756	0.17141
kz000912	Isorhamnetin-3-O-rutinoside (Narcissin)	Flavonols	1.05960	0.39989	kz005375	Isolariciresinol 9'-O-Glucoside	Lignans	1.07072	3.29126	kz000779	Diosmetin	Flavones	1.05236	0.43792	kz003766	Uncargenin D	Terpenoids	1.08938	0.17940
kz001193	Prunetin	Isoflavones	1.03896	0.40117	kz003849	dihydrodehydrodiconiferyl alcohol 4-O-β-D-glucopyranosides	Lignans	1.06749	3.32254	kz002419	Ergotamine	Alkaloids	1.07804	0.44473	kz004735	Ligraminol E	Others	1.07103	0.18244
kz00299	Quercetin-7-O-(6'-O-malonyl)-β-D-glucoside	Flavonols	1.0231	0.40496	kz00127	L-Homoserine	Organic acids	1.0657	3.33586	kz00057	Hexahydroxydiphenoylglucose	Phenolic acids	1.0770	0.45837	km1211	Chrysoeriol 7-O-hexoside	Flavones	1.0569	0.18499

5			9	
kz003714	6-O-Galloyl-β-D-glucose	Phenolic acids	1.068888	0.41493
kz003497	3-O-Galloyl-β-D-glucose	Phenolic acids	1.07316	0.42022
kz000070	Anthranilate O-hexosyl-O-hexoside	Phenolic acids	1.04707	0.42906
kz003100	5-O-Galloylhamamelose	Phenolic acids	1.07286	0.43245
km0548	Dihydroquercetin (Taxifolin)	Flavonols	1.04553	0.44054
kz001041	N-Acetyl-D-glucosamine 1-phosphate	Others	1.07135	0.44141
kz001287	(S)-(-)-2-Hydroxyisocaproic acid	Organic acids	1.05357	0.44665
kz001297	(Rs)-Mevalonic acid	Organic acids	1.07268	0.45620
km1405	Nicotinic acid-hexoside	Vitamins	1.02556	0.45863
kz004133	L-Ascorbic acid	Vitamins	1.05364	0.46904
kz0	6-O-caffeoyl-β-glucose	Pheno	1.	0.47

9			8	
kz004762	2-Hydroxy-5,8,11,14,17-icosapentaenoyloxy]propyl-2-(trimethylammonio)ethyl phosphate	Alkaloids	1.07076	3.36971
kz003861	3-O-Trans-feruloyl euscaphic acid	Terpenoids	1.06075	3.39804
kz003572	Biondnoid I	Flavonols	1.06881	3.40340
kz000915	6-Hydroxykaempferol-7,6-O-Diglucoside	Flavonols	1.06775	3.41739
kz001032	N-Acetyl-D-galactosamine	Others	1.04983	3.47216
kz005389	Byzantionoside A	Others	1.05100	3.48073
kz000422	Butin	Flavanones	1.07017	3.51646
km0814	Morin	Flavonols	1.06903	3.59070
kz004678	Annuionone D	Others	1.05800	3.59453
kz000159	Naringenin 7-O-glucoside (Prunin)	Flavanols	1.06908	3.60882
kz0	5-Aminolevulinate	Alkaloi	1.	3.61

7			4	
km0239	Chrysoeriol	Flavones	1.02687	0.48043
kz005355	Koaburaside	Phenolic acids	1.07965	0.49615
kz002807	Dimethylmalonic acid	Organic acids	1.00986	2.06844
kz001292	4-Acetamidobutyric acid	Organic acids	1.05107	2.08901
kz003721	Maplexin G	Phenolic acids	1.02258	2.20535
kz001315	Anchoic Acid	Organic acids	1.05392	2.24652
kz000698	Cyanidin-3-O-galactoside	Anthocyanins	1.00690	2.28688
kz002554	p-Hydroxycinnamic acid	Phenolic acids	1.06993	2.28972
kz000496	Vanillic acid	Phenolic acids	1.07905	2.29229
kz000573	p-Coumaroylferuloyltartaric acid	Phenolic acids	1.06118	2.32036
kz0	Ayapin	Coum	1.	2.34

			3	
kz002549	Vanillic acid glycoside	Phenolic acids	1.090800	0.18635
kz001041	N-Acetyl-D-glucosamine 1-phosphate	Others	1.09199	0.19004
kz000056	Salicylic acid glucoside	Phenolic acids	1.09089	0.19816
kz000190	Arbutin	Others	1.08313	0.20892
kz001084	Ursolic acid	Terpenoids	1.08711	0.21260
kz002486	1,4,8-trihydroxy naphthalene-1-O-β-D-glucopyranoside	Quinones	1.08293	0.21612
kz000504	Caffeic acid	Phenolic acids	1.08884	0.21647
kz000284	3-Hydroxypropanoic acid	Organic acids	1.08578	0.21794
kz003098	3-O-Digalloyl quinic acid	Phenolic acids	1.08647	0.22125
kz000573	p-Coumaroylferuloyltartaric acid	Phenolic acids	1.08902	0.23075
kz0	Aucubin	Terpe	1.	0.23

02558		lic acids	07033	716
kz000549	1-O-[(E)-Caffeoyl]-β-D-glucopyranose	Phenolic acids	1.06973	0.47892
kz001598	Stachydrine	Alkaloids	1.07050	0.48105
kz005370	(+)-Isolariciresinol	Lignans	1.05437	0.48218
kz000294	2-Hydroxyisocaproic acid	Organic acids	1.05281	0.48891
kz000550	Coniferin	Phenolic acids	1.06977	0.49157
kz001075	Methyl gallate	Phenolic acids	1.05566	2.00149
kz001032	N-Acetyl-D-galactosamine	Others	1.05257	2.02646
kz000412	Aucubin	Terpenoids	1.04852	2.10309
kz000501	Methyl p-coumarate	Phenolic acids	1.06921	2.10498
kz00303	3-O-(2-O-Acetyl-β-D-glucopyranosyl) oleanolic acid	Terpenoids	1.0671	2.11178
00231		ds	06905	282
kz002485	3-methoxy-juglone	Quinones	1.05776	3.64044
kz000434	Pinobanksin	Flavanols	1.07108	3.64092
kz001093	Oleanolic acid 2-O-β-D-glucopyranoside	Terpenoids	1.06245	3.66434
kz003116	2,4,6-trihydroxy benzoic acid	Organic acids	1.03762	3.72965
km0849	Naringenin chalcone	Flavanones	1.07046	3.75267
km0843	Naringenin	Flavanones	1.07062	3.76856
kz005054	Chrysin-7-glucoside	Flavones	1.06899	3.77004
kz003793	Dihydromyricetin-O-glucoside	Flavanols	1.03801	3.83247
kz005370	(+)-Isolariciresinol	Lignans	1.06643	3.85273
kz00050	Caffeic acid	Phenolic acids	1.0635	3.85662
03262		arins	04879	091
kz001156	Pyridoxine	Vitamins	1.06146	2.34336
kz004133	L-Ascorbic acid	Vitamins	1.06876	2.36217
kz000992	Pinoresinol	Lignans	1.07431	2.37234
kz003796	1'-O-vanilloyl-β-D-glucoside	Phenolic acids	1.03215	2.38230
kz000484	Vanillin	Phenolic acids	1.08041	2.38648
kz001075	Methyl gallate	Phenolic acids	1.06123	2.39821
kz001285	2-Methylsuccinic acid	Organic acids	1.06770	2.41187
kz001276	Succinic acid	Organic acids	1.07987	2.43794
km0081	Genistein (4',5,7-Trihydroxyisoflavone)	Isoflavones	1.04891	2.46264
kz00462	pomolic acid	Terpenoids	1.0804	2.47750
00412		noids	08717	226
kz002220	Olivil-4′-O-β-D-glucoside	Lignans	1.09058	0.23878
kz003811	Kaempferol-malonyl-3-O-glucoside	Flavonols	1.08061	0.23941
kz002546	Isosalicylic acid O-glycoside	Phenolic acids	1.08290	0.25156
kz001075	Methyl gallate	Phenolic acids	1.08729	0.25658
kz000059	3-O-p-Coumaroylshikimic acid	Phenolic acids	1.06099	0.25698
kz000995	Syringaresinol	Lignans	1.09021	0.26236
kz002788	Sexangularetin 3-glucoside-7-rhamnoside	Flavonols	1.06493	0.26358
km1113	5-O-p-Coumaroyl shikimic acid	Phenolic acids	1.01821	0.26951
kz000539	3-Hydroxy-4-isopropylbenzylalcohol 3-glucoside	Phenolic acids	1.08900	0.27193
km0843	Naringenin	Flavanones	1.0918	0.27462

8			2	
kz000410	Sweroside	Terpenoids	1.06827	2.13944
km0879	Orotic acid	Vitamins	1.03316	2.20107
kz001819	Salicylic acid	Phenolic acids	1.05761	2.20623
kz005137	Isoscopoletin (6-Hydroxy-7-Methoxycoumarin)	Coumarins	1.02057	2.25844
kz000532	Protocatechuic acid-4-glucoside	Phenolic acids	1.06474	2.26794
kz003850	(-)-secoisolariciresinol 4-O-β-D-giucopyranoside	Lignans	1.00089	2.27410
kz001105	Acetylcholine	Alkaloids	1.05928	2.28872
kz001157	4-Pyridoxic acid	Vitamins	1.06350	2.37074
kz001103	6-Hydroxynicotinic acid	Alkaloids	1.05445	2.42076
kz000884	Kaempferol-3-O-glucoside (Astragalin)	Flavonols	1.06557	2.42124
kz0	dihydrodehydrodiconiferyl	Lignan	1.	2.43

4			3	
kz002949	Hispidulin	Flavones	1.06558	3.92014
kz001283	6-Aminocaproic acid	Organic acids	1.06941	3.94525
kz000554	Chlorogenic acid	Phenolic acids	1.06790	3.96755
kz000410	Sweroside	Terpenoids	1.06285	4.05710
kz000121	Chrysin 5-O-glucoside (Toringin)	Flavones	1.06742	4.11841
kz000145	Formononetin	Isoflavones	1.06813	4.12330
kz000475	4-Hydroxybenzoic acid	Phenolic acids	1.06424	4.19149
kz000047	Mandelic acid	Phenolic acids	1.04735	4.23277
kz004035	3-Indoleacrylic acid	Alkaloids	1.06762	4.35854
kz001018	3-Amino-1-propionic sulfonic acid	Others	1.04547	4.35860
kz0	N-Acetyl-D-glucosamine 1-	Other	1.	4.38

6			2	
kz000920	Quercetin-O-rutinoside-hexose	Flavonols	1.03040	2.48893
kz002219	Epipinoresinol	Lignans	1.07510	2.49688
kz002220	Olivil-4′-O-β-D-glucoside	Lignans	1.06680	2.50731
kz001087	Maslinic acid	Terpenoids	1.08016	2.50938
kz000049	2-(Formylamino)benzoic acid	Phenolic acids	1.07964	2.51801
kz001275	Methylmalonic acid	Organic acids	1.08004	2.52254
kz001089	2-Hydroxyoleanolic acid	Terpenoids	1.07911	2.54107
kz003549	Hederagenin	Others	1.08129	2.56396
kz004627	alphitolic acid	Terpenoids	1.07942	2.56831
kz001076	Ethyl gallate	Phenolic acids	1.05592	2.57566
kz0	p-Coumaric acid	Pheno	1.	2.58

			5	
km0849	Naringenin chalcone	Flavanones	1.09115	0.28073
kz003517	Glucosyloxybenzoic acid	Phenolic acids	1.08787	0.28273
kz002556	4-O-glucosyl-4-hydroxybenzoic acid	Phenolic acids	1.07050	0.28460
kz002853	Dihydroisopelletierine	Alkaloids	1.08991	0.28480
kz000422	Butin	Flavanones	1.09107	0.28847
kz000434	Pinobanksin	Flavanols	1.09184	0.29289
kz000533	2,5-Dihydroxy benzoic acid O-hexside	Phenolic acids	1.05909	0.29836
kz005375	Isolariciresinol 9'-O-Glucoside	Lignans	1.08554	0.30355
kz003849	dihydrodehydrodiconiferyl alcohol 4-O-β-D-glucopyranosides	Lignans	1.08837	0.30404
kz003721	Maplexin G	Phenolic acids	1.08339	0.30413
kz0	(-)-secoisolariciresinol 4-	Lignan	1.	0.30

038 49	alcohol 4-O-β-D-glucopyranosides	s	06837	638
kz0002258	Nicotinate D-ribonucleoside	Vitamins	1.06761	2.45854
kz0000051	4-Methoxycinnamic acid	Phenolic acids	1.07060	2.49319
km1300	Hydroxy-methoxycinnamate	Phenolic acids	1.07116	2.53063
kz0027 83	Kaempferol 3-O-β-d-(6''-O-(E)-p-coumaroyl) glucopyranoside	Flavonols	1.06005	2.53750
kz0005 12	Syringic acid	Phenolic acids	1.05716	2.54720
kz0011 13	Lumichrome	Alkaloids	1.04383	2.57143
kz0053 75	Isolariciresinol 9'-O-Glucoside	Lignans	1.06148	2.59660
kz0002 59	Thiamine	Vitamins	1.05072	2.59858
kz0041 71	Kaempferol 3-O-β-D-neohesperidoside	Flavonols	1.04893	2.62687
km0683	Kaempferol 3-O-galactoside (Trifolin)	Flavonols	1.0664	2.65608
01041	phosphate	s	07010	410
kz0047 35	Ligraminol E	Others	1.04383	4.45023
kz0020 65	4-α-L-Rhamnopyranosyl-ellagic acid	Phenolic acids	1.06717	4.48941
kz0004 87	2,5-Dihydroxybenzoic acid	Phenolic acids	1.06933	4.51498
kz0009 78	Catechin-(7,8-bc)-4β-(3,4-dihydroxyphenyl)-dihydro-2-(3H)-pyranone	Flavanols	1.07067	4.56812
kz0009 79	Catechin-(7,8-bc)-4α-(3,4-dihydroxyphenyl)-dihydro-2-(3H)-pyranone	Flavanols	1.07053	4.62460
km0573	Epicatechin gallate (ECG)	Flavanols	1.06474	4.71816
kz0013 02	2,3-Dihydroxybenzoic Acid	Organic acids	1.06974	4.73342
kz0004 89	Protocatechuic acid	Phenolic acids	1.06922	4.74595
kz0002 94	2-Hydroxyisocaproic acid	Organic acids	1.06783	4.98653
kz0007	Anthranilate O-hexosyl-O-hexoside	Phenolic acids	1.0706	5.00455
00491		lic acids	07170	240
kz0010 88	3,24-Dihydroxy-17,21-semiacetal-12(13)oleanolic fruit	Terpenoids	1.07955	2.58621
kz0002 90	Aminomalonic acid	Organic acids	1.07751	2.61736
kz0030 97	3,4-Di-O-galloyl-shikimic acid	Phenolic acids	1.07553	2.62830
kz0037 20	Maplexin H	Phenolic acids	1.02744	2.64700
km0235	Apigenin	Flavones	1.05569	2.67204
kz0010 91	Euscaphic acid	Terpenoids	1.08143	2.71197
kz0000 52	Ethyl 3,4-Dihydroxybenzoate (Ethyl protocatechuate)	Phenolic acids	1.04454	2.71783
kz0029 33	Ixerin D	Terpenoids	1.07033	2.73798
kz0002 94	2-Hydroxyisocaproic acid	Organic acids	1.06812	2.81987
kz0103	N-Acetyl-D-galactosamine	Others	1.0494	2.84292
03850	O-β-D-giucopyranoside	s	08755	642
kz0021 96	2'-Hydoxy,5-methoxy Genistein-O-rhamnosyl-glucoside	Isoflavones	1.04148	0.30708
kz0005 20	Sinapic acid	Phenolic acids	1.07123	0.30805
kz0031 51	Ailantinol E	Others	1.08852	0.30916
km0602	Genistein 7-O-Glucoside (Genistin)	Isoflavones	1.03963	0.31645
kz0040 15	Cis-p-coumaric acid 4-O-glucoside	Others	1.07704	0.32180
kz0007 92	Apigenin 5-O-glucoside	Flavones	1.07828	0.32197
kz0009 12	Isorhamnetin-3-O-rutinoside (Narcissin)	Flavonols	1.08889	0.32445
kz0005 40	3,4,5-Trimethoxyphenyl-β-D-Glucopyranoside	Phenolic acids	1.09088	0.32704
kz0016 37	Oresbiusin A	Phenolic acids	1.08868	0.33067
kz0255	6-O-caffeoyl-β-glucose	Phenolic acids	1.0909	0.33256

			0	
kz002063	Galloyl-HHDP(Hexahydroxydiphenoyl)-glueopy ranose	Phenolic acids	1.00994	2.69088
kz000514	3,4-Dimethoxycinnamic acid	Phenolic acids	1.06943	2.71760
km0779	Luteolin 7-O-glucoside	Flavones	1.06957	2.77523
kz003572	Biondnoid I	Flavonols	1.06544	2.77758
kz003718	2,3-Di-O-Galloyl-D-Glucose	Phenolic acids	1.05107	2.79138
kz003690	Quercetin-3-O-α-L-rhamnopyranoside	Flavonols	1.06305	2.79813
kz005078	Kaempferol-7-O-β-D-glucopyranoside	Flavonols	1.06866	2.83539
kz002552	Sinapic acid-glycoside	Phenolic acids	1.05914	2.87392
kz003097	3,4-Di-O-galloyl-shikimic acid	Phenolic acids	1.05239	2.91301
kz002223	Eucommia	Others	1.05326	2.92261
kz0	Annuionone D	Other	1.	2.94

0			9	
kz000068	Catechin gallate	Phenolic acids	1.06943	5.08402
kz000560	Trihydroxycinnamoylquinic acid	Phenolic acids	1.06992	5.31420
kz003100	5-O-Galloylhamamelose	Phenolic acids	1.07073	5.41880
kz003497	3-O-Galloyl-β-D-glucose	Phenolic acids	1.07058	5.53448
kz000259	Thiamine	Vitamins	1.06108	5.67838
kz004667	Myricetin-O-rhamnoside	Flavonols	1.06741	5.70000
kz003714	6-O-Galloyl-β-D-glucose	Phenolic acids	1.07084	5.77384
km0532	Delphinidin 3-O-glucoside (Mirtillin)	Anthocyanins	1.01801	5.79622
kz004133	L-Ascorbic acid	Vitamins	1.06760	5.84708
kz003147	Clemaphenol A	Lignans	1.06761	5.88031
kz0	Quercetin-7-O-Glucoside	Flavon	1.	5.98

2			3	
kz001268	2-Furanoic acid	Organic acids	1.07896	2.86430
kz001313	SubericAcid	Organic acids	1.00389	2.91019
kz000047	Mandelic acid	Phenolic acids	1.05122	2.94731
kz004678	Annuionone D	Others	1.07756	3.02735
kz005247	Limonin	Others	1.07693	3.15267
kz005385	Machilusolide D	Others	1.07790	3.19165
kz001157	4-Pyridoxic acid	Vitamins	1.08086	3.22343
kz004636	2α-hydroxypyracrenic acid	Terpenoids	1.07096	3.44325
km0814	Morin	Flavonols	1.07979	3.51628
kz001283	6-Aminocaproic acid	Organic acids	1.02873	3.62077
kz0	(S)-(-)-2-Hydroxyisocaproic acid	Organi	1.	3.70

8			2	
kz003860	Caffeoyl hawthorn acid	Terpenoids	1.07709	0.33300
kz000505	Coniferyl alcohol	Phenolic acids	1.03237	0.33757
kz004636	2α-hydroxypyracrenic acid	Terpenoids	1.07895	0.33764
kz000549	1-O-[(E)-Caffeoyl]-β-D-glucopyranose	Phenolic acids	1.08777	0.33918
kz000070	Anthranilate O-hexosyl-O-hexoside	Phenolic acids	1.08805	0.34112
kz005055	Galangin-7-glucoside	Flavones	1.07689	0.34186
kz000791	Apigenin 7-O-glucoside(Cosmosiin)	Flavones	1.04324	0.34247
kz003097	3,4-Di-O-galloyl-shikimic acid	Phenolic acids	1.08887	0.34370
kz000302	2-Isopropylmalate	Organic acids	1.06581	0.34531
kz002552	Sinapic acid-glycoside	Phenolic acids	1.08715	0.34595
kz0	2,4,6-trihydroxy benzoic	Organi	1.	0.35

04678		s	06242	676	00890		ols	07055	684	01287		c acids	06399	243	03116	acid	c acids	05914	290
kz002604	Kaempferol-3-O-(6''-acetyl)-glucoside	Flavonols	1.03794	2.95830	kz001586	Isotamarixin	Flavonols	1.05827	6.00153	kz001642	5'-Glucopyranosyloxyjasmanic acid	Phenolic acids	1.07304	3.75554	kz000992	Pinoresinol	Lignans	1.08730	0.35542
kz000866	Quercetin	Flavonols	1.06308	2.96931	km0934	Quercetin 4'-O-glucoside (Spiraeoside)	Flavonols	1.07007	6.09726	kz000487	2,5-Dihydroxybenzoic acid	Phenolic acids	1.08078	3.82936	kz000278	Indole 3-acetic acid (IAA)	Alkaloids	1.07662	0.35623
kz001096	Choline	Alkaloids	1.07165	2.97053	kz003158	Ailanindole	Alkaloids	1.03943	6.13493	kz003728	Geniposide	Terpenoids	1.04866	3.96040	kz001020	4-Methyl-5-thiazoleethanol	Others	1.08838	0.35675
kz002990	Luteolin-7-O-rutinoside	Flavones	1.04300	2.99486	kz000539	3-Hydroxy-4-isopropylbenzylalcohol 3-glucoside	Phenolic acids	1.06759	6.54618	kz000231	5-Aminolevulinate	Alkaloids	1.01434	4.03545	kz002548	p-Coumaric acid-O-glycoside	Phenolic acids	1.08661	0.35735
kz000906	Kaempferol-3-O-robinobioside(Biorobin)	Flavonols	1.06657	3.00404	km1269	Di-O-methylquercetin	Flavonols	1.07100	6.67062	kz001097	Betaine	Alkaloids	1.07534	4.09177	kz000550	Coniferin	Phenolic acids	1.09147	0.35896
kz000905	Kaempferol-3-O-rutinoside(Nicotiflorin)	Flavonols	1.06961	3.02306	kz000992	Pinoresinol	Lignans	1.06899	6.67471	kz001302	2,3-Dihydroxybenzoic Acid	Organic acids	1.07963	4.09883	kz000554	Chlorogenic acid	Phenolic acids	1.08701	0.36245
kz000812	Tricin O-saccharic acid	Flavones	1.06725	3.02732	kz002219	Epipinoresinol	Lignans	1.06828	6.77903	kz001084	Ursolic acid	Terpenoids	1.07232	4.23613	kz002219	Epipinoresinol	Lignans	1.08445	0.36832
kz001188	Esculin(6,7-DihydroxyCoumarin-6-glucoside)	Coumarins	1.05349	3.04864	kz000553	Neochlorogenic acid(5-O-Caffeoylquinic acid)	Phenolic acids	1.06299	6.84868	kz000259	Thiamine	Vitamins	1.06562	4.26344	kz001097	Betaine	Alkaloids	1.08493	0.37370
kz000795	Luteolin-7-O-glucoside(Cynaroside)	Flavones	1.07261	3.06588	kz001287	(S)-(-)-2-Hydroxyisocaproic acid	Organic acids	1.06516	7.06891	kz000489	Protocatechuic acid	Phenolic acids	1.07961	4.27432	kz001079	Galloyl Methyl gallate	Phenolic acids	1.06758	0.38080
kz00069	Cyanidin-3-O-galactoside	Anthocyanins	1.0295	3.07524	kz00119	Indole-3-carboxaldehyde	Alkaloids	1.0708	7.07544	kz00046	4-Hydroxybenzaldehyde	Phenolic acids	1.0797	4.70660	kz00030	DL-Glyceraldehyde 3-phosphate	Organic acids	1.0748	0.38436

8			3		9			0		6			6		0			9	
kz005198	Poncirin(Isosakuranetin-7-neohesperidoside)	Flavanones	1.07304	3.08352	kz003848	Cinchonain Ic	Flavanols	1.06793	7.09376	kz001297	(Rs)-Mevalonic acid	Organic acids	1.07511	4.77381	kz000536	5-Galloylshikimic acid	Phenolic acids	1.07902	0.38560
kz000904	Tiliroside	Flavonols	1.07259	3.09789	kz000201	D-galacitol	Others	1.07142	7.12746	kz000864	Kaempferol	Flavonols	1.07755	4.80867	kz003861	3-O-Trans-feruloyl euscaphic acid	Terpenoids	1.06752	0.39931
kz000201	D-galacitol	Others	1.07357	3.09916	kz003721	Maplexin G	Phenolic acids	1.06211	7.25134	kz001184	Skimmin	Coumarins	1.07804	4.81691	kz005084	(Kaempferol-3-O-β-D-galactoside-4'O-β-D-glucoside)	Flavonols	1.02946	0.40163
kz005132	Luteolin-caffeoyl-O-rhamnoside	Flavones	1.07300	3.14774	kz000584	5-O-p-Coumaroyl quinic acid O-hexoside	Phenolic acids	1.05351	7.27652	kz003860	Caffeoyl hawthorn acid	Terpenoids	1.07752	5.06530	kz004133	L-Ascorbic acid	Vitamins	1.07212	0.40399
kz002807	Dimethylmalonic acid	Organic acids	1.03466	3.19062	kz001103	6-Hydroxynicotinic acid	Alkaloids	1.05583	7.59341	kz002223	Eucommia	Others	1.06862	5.15232	kz005355	Koaburaside	Phenolic acids	1.09118	0.41497
kz000700	Cyanidin-3-O-glucoside (Kuromanin)	Anthocyanins	1.01999	3.19969	kz001706	Isololiolide	Others	1.06773	7.59886	kz000302	2-Isopropylmalate	Organic acids	1.07942	5.17149	kz001018	3-Amino-1-propionic sulfonic acid	Others	1.05709	0.42076
kz001313	SubericAcid	Organic acids	1.00460	3.21686	kz003097	3,4-Di-O-galloyl-shikimic acid	Phenolic acids	1.06974	7.64717	km1269	Di-O-methylquercetin	Flavonols	1.08122	5.17773	kz000915	6-Hydroxykaempferol-7,6-O-Diglucoside	Flavonols	1.09035	0.42239
kz005079	Kaempferol-4'-O-β-D-glucopyranoside	Flavonols	1.06339	3.23418	kz004756	N-Benzylmethylene isomethylamine	Alkaloids	1.07006	8.03292	kz001298	L-(+)-Tartaric acid	Organic acids	1.07897	5.25556	kz002197	2'-Hydoxy,5-methoxy Genistein-4',7-O-diglucoside	Isoflavones	1.00751	0.42683
kz001308	D-Xylonic acid	Organic acids	1.07017	3.45455	kz000419	Phlorizin	Chalcones	1.05942	8.05133	kz004756	N-Benzylmethylene isomethylamine	Alkaloids	1.05150	5.45872	km0936	Quercetin 7-O-β-D-Glucuronide	Flavonols	1.08884	0.43055
kz005385	Machilusolide D	Others	1.07208	3.48257	kz005186	Dunalianoside C	Phenolic acids	1.07066	8.05593	kz004233	Terminolic acid	Terpenoids	1.07781	5.49624	kz001319	D-Galacturonic acid(GalA)	Organic acids	1.08459	0.43750
km	Morin	Flavon	1.	3.59	km	3-(4-Hydroxyphenyl)propionic	Pheno	1.	8.28	kz0	4-Hydroxybenzoic acid	Pheno	1.	5.53	kz0	2-Picolylamine	Alkaloi	1.	0.43

0814		ols	07348	486
kz000302	2-Isopropylmalate	Organic acids	1.00289	3.60187
kz001285	2-Methylsuccinic acid	Organic acids	1.06254	3.61111
kz001076	Ethyl gallate	Phenolic acids	1.04478	3.66573
kz001637	Oresbiusin A	Phenolic acids	1.06278	3.71291
kz000915	6-Hydroxykaempferol-7,6-O-Diglucoside	Flavonols	1.07247	3.75566
kz001315	Anchoic Acid	Organic acids	1.06342	3.77658
kz003262	Ayapin	Coumarins	1.06078	3.82615
kz002554	p-Hydroxycinnamic acid	Phenolic acids	1.04499	3.86165
kz000914	6-Hydroxykaempferol-3,6-O-Diglucoside	Flavonols	1.05532	3.94798
kz00315	Ailantinol E	Others	1.0654	3.95314
0329	acid	lic acids	06962	974
kz004495	3-(4-Hydroxyphenyl)-propionic acid	Phenolic acids	1.06490	8.32085
kz004738	4,7,9,9′-Tetrahydroxy-3,3′-dimethoxy-8-O-4′-neolignan	Others	1.07038	8.37738
kz004632	isoceanothic acid	Terpenoids	1.07100	8.49722
kz001880	feruloylsinapoyltartaric acid	Phenolic acids	1.06865	8.50133
kz000914	6-Hydroxykaempferol-3,6-O-Diglucoside	Flavonols	1.06639	8.69565
kz001742	Methyl Brevifolincarboxylate	Others	1.06570	8.70990
kz002665	Betulinic acid	Terpenoids	1.04203	8.89112
kz002549	Vanillic acid glycoside	Phenolic acids	1.05260	9.12409
kz000431	Eriodictyol 7-O-glucoside	Flavanones	1.07118	9.27738
kz00107	Methyl gallate	Phenolic acids	1.0683	9.34676
00475		lic acids	07457	989
km08792	Orotic acid	Vitamins	1.07842	5.71711
kz003863	Sanguisorbigenin	Terpenoids	1.08109	5.72805
kz001289	Malic acid	Organic acids	1.07684	6.11345
kz004762	2-Hydroxy-5,8,11,14,17-icosapentaenoyloxy]propyl-2-(trimethylammonio)ethyl phosphate	Alkaloids	1.08174	6.16751
kz000201	D-galacitol	Others	1.08186	6.32213
kz000548	Trans-3-O-p-coumaric quinic acid	Phenolic acids	1.08080	6.56784
kz000997	Terpineol monO-glucoside	Lignans	1.07971	6.72781
kz001199	Indole-3-carboxaldehyde	Alkaloids	1.08088	6.90665
kz003718	2,3-Di-O-Galloyl-D-Glucose	Phenolic acids	1.06599	6.94275
kz00383	Quercetin-O-pentosyl-O-rhamnoside-O-glucoside	Flavonols	1.0476	6.99027
00414		ds	05900	785
kz000078	1'-O-β-D-(3,4-Dihydroxyphenethyl)-O-caffeoyl-glucoside	Phenolic acids	1.07047	0.45399
kz005136	Camaldulenic acid	Terpenoids	1.09167	0.45862
km0231	2'-Hydroxygenistein	Isoflavones	1.00050	0.46076
km0071	Quercetin 3-O-galactoside (Hyperin)	Flavonols	1.08492	0.46917
kz000537	3-Galloylshikimic acid	Phenolic acids	1.05963	0.46976
kz002607	Lariciresinol glucopyranoside	Others	1.02261	0.48104
kz002214	isohyperoside	Flavonols	1.08681	0.48127
km0228	Quercetin 3-O-glucoside (Isotrifoliin)	Flavonols	1.08542	0.48673
kz004697	Taxifolin-3'-O-β-D-glucoside	Flavones	1.06320	0.48860
kz00044	Hesperetin 5-O-glucoside	Flavanols	1.0898	0.48869

1			9	
kz000491	p-Coumaric acid	Phenolic acids	1.07000	4.02594
kz005046	Dihydrokaempferol-3-O-β-D-glucoside	Flavanols	1.05589	4.04815
kz000290	Aminomalonic acid	Organic acids	1.07145	4.07392
kz002164	Isoluteolin (Orobol)(5,7,3',4'-tetrahydroxyisoflavone)	Isoflavones	1.05725	4.13277
kz001275	Methylmalonic acid	Organic acids	1.07332	4.17352
kz001276	Succinic acid	Organic acids	1.07319	4.19207
kz000049	2-(Formylamino)benzoic acid	Phenolic acids	1.07044	4.23854
kz000511	Ferulic acid	Phenolic acids	1.07223	4.32898
kz001293	4-Guanidinobutyric acid	Organic acids	1.07340	4.39097
kz000864	Kaempferol	Flavonols	1.06808	4.41896
kz0	Isoferulic Acid	Pheno	1.	4.53

5			9	
kz003763	Betulin	Terpenoids	1.05741	9.65690
kz003038	3-O-(2-O-Acetyl-β-D-glucopyranosyl) oleanolic acid	Terpenoids	1.07073	9.84000
kz003863	Sanguisorbigenin	Terpenoids	1.07136	9.91435
kz000573	p-Coumaroylferuloyltartaric acid	Phenolic acids	1.06962	10.05562
kz001289	Malic acid	Organic acids	1.06948	10.09724
kz004636	2α-hydroxypyracrenic acid	Terpenoids	1.07072	10.19785
kz001101	Trigonelline	Alkaloids	1.07104	10.24706
kz000417	Phloretin	Chalcones	1.06554	10.35939
kz002220	Olivil-4′-O-β-D-glucoside	Lignans	1.06942	10.50038
kz001097	Betaine	Alkaloids	1.06914	10.94937
kz0	Indole-3-carboxylic acid	Alkaloi	1.	12.3

3			1	
kz001281	Citraconic acid	Organic acids	1.07901	7.25770
kz001103	6-Hydroxynicotinic acid	Alkaloids	1.06580	7.50983
kz000495	3-(4-Hydroxyphenyl)-propionic acid	Phenolic acids	1.07700	7.77154
kz003038	3-O-(2-O-Acetyl-β-D-glucopyranosyl) oleanolic acid	Terpenoids	1.08094	8.01000
kz000410	Sweroside	Terpenoids	1.07851	8.06912
km0329	3-(4-Hydroxyphenyl)propionic acid	Phenolic acids	1.07981	8.42388
kz000515	Sinapinaldehyde	Phenolic acids	1.07973	8.59141
kz001742	Methyl Brevifolincarboxylate	Others	1.07569	9.49791
kz001101	Trigonelline	Alkaloids	1.08155	11.23529
kz004630	2α-hydroxyursolic acid	Terpenoids	1.04841	11.35773
kz0	Isotamarixin	Flavon	1.	11.4

1			9	
kz003572	Biondnoid I	Flavonols	1.08065	0.49158
kz003854	Roseoside	Others	1.06131	0.49164
kz000889	Spiraeoside	Flavonols	1.09049	0.49960
kz004171	Kaempferol 3-O-β-D-neohesperidoside	Flavonols	1.04328	2.00888
kz000886	Myricetin-3-O-arabinoside	Flavonols	1.04493	2.02655
kz003767	Obtusilin	Terpenoids	1.08924	2.05052
kz001318	Quinic Acid	Organic acids	1.08817	2.09797
kz004632	isoceanothic acid	Terpenoids	1.09183	2.10699
kz000920	Quercetin-O-rutinoside-hexose	Flavonols	1.00846	2.10884
kz001642	5'-Glucopyranosyloxyjasmanic acid	Phenolic acids	1.07976	2.16423
kz0	Kaempferol-3-O-	Flavon	1.	2.26

00053		lic acids	07291	964
kz001184	Skimmin	Coumarins	1.06735	4.60051
kz000551	Methyl 6-O-galloyl-β-D-glucopyranoside	Phenolic acids	1.07186	4.66051
kz001706	Isololiolide	Others	1.07305	4.86305
kz003728	Geniposide	Terpenoids	1.04674	4.91620
kz001084	Ursolic acid	Terpenoids	1.06634	5.10456
kz000197	E-3,4,5'-Trihydroxy-3'-glucopyranosylstilbene	Others	1.02914	5.13043
kz005378	5'-methoxyisolariciresinol-9'-O-β-D-glucopyranoside	Lignans	1.06240	5.21127
km0235	Apigenin	Flavones	1.06452	5.59475
kz001742	Methyl Brevifolincarboxylate	Others	1.06448	6.40339
kz00047	4-Hydroxybenzoic acid	Phenolic acids	1.0705	6.48910
01203		ds	06964	5811
kz000424	Eriodictyol	Flavanones	1.07138	12.50769
kz000997	Terpineol monO-glucoside	Lignans	1.07035	12.72189
kz000548	Trans-3-O-p-coumaric quinic acid	Phenolic acids	1.06922	12.81341
kz002673	Eriodictyol-O-glucoside	Flavones	1.07119	12.98354
kz003540	Dihydrokaempferol-7-O-glucoside	Flavonols	1.07064	12.99569
kz001292	4-Acetamidobutyric acid	Organic acids	1.07026	13.04348
kz005187	Dunalianoside B	Phenolic acids	1.07036	13.77163
kz000302	2-Isopropylmalate	Organic acids	1.06799	14.97646
kz003860	Caffeoyl hawthorn acid	Terpenoids	1.06849	15.21127
kz00044	Astilbin	Flavanols	1.0690	16.51626
01586		ols	07621	2202
kz002665	Betulinic acid	Terpenoids	1.06610	11.49670
kz00055	3,4,5-Trimethoxycinnamic acid	Phenolic acids	1.08142	11.53710
km1293	Hesperetin O-malonylhexoside	Flavanones	1.08047	11.57658
kz002065	4-α-L-Rhamnopyranosyl-ellagic acid	Phenolic acids	1.08053	11.65176
kz001093	Oleanolic acid 2-O-β-D-glucopyranoside	Terpenoids	1.07989	12.14286
kz001319	D-Galacturonic acid(Gal A)	Organic acids	1.08176	12.36559
kz002221	Eucommin A	Lignans	1.06612	13.74896
kz001014	1-Methylhistamine	Others	1.07167	13.77191
kz003763	Betulin	Terpenoids	1.07154	14.39957
kz00120	Indole-3-carboxylic acid	Alkaloids	1.0804	15.37838
00906	robinobioside(Biorobin)	ols	07854	090
kz000905	Kaempferol-3-O-rutinoside(Nicotiflorin)	Flavonols	1.08705	2.39634
kz000553	Isoferulic Acid	Phenolic acids	1.08995	2.40177
kz000511	Ferulic acid	Phenolic acids	1.08797	2.40988
kz004595	Geraniin	Phenolic acids	1.03483	2.41707
kz000064	1-O-Feruloyl quinic acid	Phenolic acids	1.03412	2.47756
kz003504	Geraniinic acid C	Phenolic acids	1.04495	2.50161
kz001706	Isololiolide	Others	1.09085	2.53190
kz001113	Lumichrome	Alkaloids	1.06721	2.54839
kz001281	Citraconic acid	Organic acids	1.08721	2.56137
kz00206	4-α-L-Rhamnopyranosyl-ellagic acid	Phenolic acids	1.0861	2.59539

5			0	
km0081	Genistein (4',5,7-Trihydroxyisoflavone)	Isoflavones	1.06191	6.49981
kz001973	Kaempferol-3-O-(cinnamoyl)-sophoroside-7-O-glucose	Flavonols	1.06993	6.90681
kz001025	D-Glucurono-6,3-lactone	Others	1.07082	7.05453
kz003362	Tubuloside C	Phenolic acids	1.06149	7.43855
km1269	Di-O-methylquercetin	Flavonols	1.07379	7.54750
kz005200	Hesperetin 7-O-neohesperidoside(Neohesperidin)	Flavanones	1.07258	7.93928
kz000145	Formononetin	Isoflavones	1.07389	8.51113
kz003763	Betulin	Terpenoids	1.06961	8.92952
kz001319	D-Galacturonic acid(Gal A)	Organic acids	1.07305	9.51537
kz000496	Vanillic acid	Phenolic acids	1.07315	10.59055
kz0	6-Hydroxy-7-methoxycoumarin	Coum	1.	12.0
0			7	
kz001193	Prunetin	Isoflavones	1.07071	17.30717
kz000613	1,2,3,6-Tetra-O-Galloyl-D-Glucose	Phenolic acids	1.06516	17.39216
kz003767	Obtusilin	Terpenoids	1.06572	17.58507
km1293	Hesperetin O-malonylhexoside	Flavanones	1.07066	17.63514
km0080	Biochanin A	Isoflavones	1.07118	18.73860
kz002221	Eucommin A	Lignans	1.05910	19.51016
kz001084	Ursolic acid	Terpenoids	1.06988	19.92563
kz003720	Maplexin H	Phenolic acids	1.06739	22.18718
kz001025	D-Glucurono-6,3-lactone	Others	1.07034	23.40588
kz003821	Kampferol 3-O-(6″-galloyl)-β-D-galactopyranoside	Flavonols	1.06411	27.08493
kz0	D-Galacturonic acid(Gal A)	Organi	1.	28.2
3			2	
kz005136	Camaldulenic acid	Terpenoids	1.08104	16.63321
kz001025	D-Glucurono-6,3-lactone	Others	1.08027	17.87813
kz004632	isoceanothic acid	Terpenoids	1.08165	17.90353
kz001706	Isololiolide	Others	1.08041	19.23954
kz003755	Quercetin-3-sambubioside	Flavonols	1.07938	19.85357
kz004172	Quercetin 3-O-β-D-xylopyranosyl(1→2)-β-D-galactopyranoside	Flavonols	1.08019	21.87651
kz000500	Riboprine	Phenolic acids	1.08135	24.93947
kz000841	Luteolin-O-sinapoylhexoside	Flavones	1.07877	27.92500
kz000544	3-O-p-Coumaroyl quinic acid	Phenolic acids	1.07909	29.48810
kz003767	Obtusilin	Terpenoids	1.07855	36.05860
kz0	Myricetin-O-rhamnoside	Flavon	1.	38.4
5			1	
kz002990	Luteolin-7-O-rutinoside	Flavones	1.08255	2.72000
kz000864	Kaempferol	Flavonols	1.08244	2.79011
kz003262	Ayapin	Coumarins	1.06670	2.81421
kz001315	Anchoic Acid	Organic acids	1.07582	2.82670
kz001313	SubericAcid	Organic acids	1.00567	2.99278
kz000907	Kaempferol-3-O-glucoside-7-O-rhamnoside	Flavonols	1.04844	3.21356
km0879	Orotic acid	Vitamins	1.08425	3.26001
kz001093	Oleanolic acid 2-O-β-D-glucopyranoside	Terpenoids	1.09096	3.31379
kz000274	N-sinapoylhydroxycoumarin	Coumarins	1.07800	3.94312
kz001188	Esculin(6,7-DihydroxyCoumarin-6-glucoside)	Coumarins	1.08094	4.11639
kz0	4-Hydroxybenzaldehyde	Pheno	1.	4.25

03166		arins	07329	5105
kz0010090	Methoxyursolic acid	Terpenoids	1.07376	12.74623
kz003833	Quercetin-O-pentosyl-O-rhamnoside-O-glucoside	Flavonols	1.07089	12.88176
kz001586	Isotamarixin	Flavonols	1.05218	13.21189
kz003693	Quercetin-3-O-(2-O-α-L-rhamnopyranosyl)-β-D-galactopyranoside	Flavonols	1.06979	13.27127
kz000484	Vanillin	Phenolic acids	1.06713	15.13754
kz001199	Indole-3-carboxaldehyde	Alkaloids	1.06817	18.37764
kz001203	Indole-3-carboxylic acid	Alkaloids	1.07309	18.47403
kz002065	4-α-L-Rhamnopyranosyl-ellagic acid	Phenolic acids	1.07319	19.57312
kz000466	4-Hydroxybenzaldehyde	Phenolic acids	1.06860	22.77433
kz00138	Quercetin-O-rutinoside-O-rhamnoside	Flavonols	1.0706	25.52434

01319		c acids	07106	6421
kz005136	Camaldulenic acid	Terpenoids	1.07088	36.26825
kz003796	1'-O-vanilloyl-β-D-glucoside	Phenolic acids	1.06876	38.98305
kz001074	Gallic acid	Phenolic acids	1.07065	39.10359
kz000544	3-O-p-Coumaroyl quinic acid	Phenolic acids	1.06948	42.08333
kz003718	2,3-Di-O-Galloyl-D-Glucose	Phenolic acids	1.06761	42.72838
kz001298	L-(+)-Tartaric acid	Organic acids	1.07096	44.04994
kz002978	Pratensein 7-O-glucopyranoside	Flavones	1.07064	44.42500
kz002668	Ovalifoliolides B	Terpenoids	1.06611	44.50000
kz001090	Methoxyursolic acid	Terpenoids	1.07123	44.81982
kz00084	Luteolin-O-sinapoylhexoside	Flavones	1.0683	45.10000

04667		ols	08123	1176
kz000890	Quercetin-7-O-Glucoside	Flavonols	1.08166	40.18421
km0934	Quercetin 4'-O-glucoside (Spiraeoside)	Flavonols	1.08138	42.54364
kz005037	Caffeoylferuloylshikimic?acid	Phenolic acids	1.08131	48.26133
kz000118	Sakuranetin	Flavones	1.08157	64.12500
kz002337	Luteolin-7-O-β-D-gentiobioside	Flavones	1.08103	66.25000
kz000145	Formononetin	Isoflavones	1.08158	88.79103
kz003748	7'-O-Sinapoyljasminoside L	Terpenoids	1.08118	89.52500
kz000733	Delphinidin-3,5-O-diglucoside	Anthocyanins	1.07885	92.20000
kz001086	2,3-Dihydroxy 5(6),12(13)diene ursolic acid	Terpenoids	1.08177	98.47502
kz00005	3-O-p-Coumaroylshikimic acid	Phenolic acids	1.0794	107.67500

00466		lic acids	08965	650
kz001090	Methoxyursolic acid	Terpenoids	1.09173	4.45477
kz000479	Cinnamic acid	Phenolic acids	1.08804	4.89950
kz001184	Skimmin	Coumarins	1.09025	5.26008
kz002223	Eucommia	Others	1.08557	5.67883
kz001973	Kaempferol-3-O-(cinnamoyl)-sophoroside-7-O-glucose	Flavonols	1.08465	5.71350
kz000515	Sinapinaldehyde	Phenolic acids	1.09073	5.71429
kz000055	3,4,5-Trimethoxycinnamic acid	Phenolic acids	1.08938	6.13434
kz000890	Quercetin-7-O-Glucoside	Flavonols	1.09157	6.71209
kz004667	Myricetin-O-rhamnoside	Flavonols	1.09035	6.73891
km0934	Quercetin 4'-O-glucoside (Spiraeoside)	Flavonols	1.0908	6.97751

0			1	
kz0005007	Syringic Aldehyde	Phenolic acids	1.068222	29.09677
kz0007998	Luteolin-7-O-glucuronide	Flavones	1.071766	31.76744
kz0029366	Silibinin	Flavanols	1.073688	32.20000
kz0029766	Scutellarin(Scutellarein-7-O-glucuronide)	Flavones	1.073566	33.30348
kz0017022	Kaempferol-3-O-β-D-glucuronide	Flavonols	1.071553	36.88660
kz0050377	Caffeoylferuloylshikimic?acid	Phenolic acids	1.064855	37.32681
kz0007977	Tetahydroxy-flavone-7-O-β-D-glucuronide	Flavones	1.073099	38.13977
kz0046677	Myricetin-O-rhamnoside	Flavonols	1.062288	38.63905
kz0005155	Sinapinaldehyde	Phenolic acids	1.071833	44.95557
kz0008900	Quercetin-7-O-Glucoside	Flavonols	1.072266	57.08411
km	Quercetin 4'-O-glucoside	Flavon	1.	58.3

1			9	
kz0019733	Kaempferol-3-O-(cinnamoyl)-sophoroside-7-O-glucose	Flavonols	1.07055	45.72500
kz0001566	7-O-Methyleriodictyol	Flavanols	1.069422	48.00000
kz0037288	Geniposide	Terpenoids	1.069233	50.85509
kz0042633	Osthole	Coumarins	1.071055	58.55000
kz0000522	Ethyl 3,4-Dihydroxybenzoate (Ethyl protocatechuate)	Phenolic acids	1.071066	72.05418
kz0011911	4-hydroxycoumarin di-glucoside	Coumarins	1.069555	78.25000
kz0038722	Lup-12-en-15α,19β-diol-3,11-dioxo-28-oic acid	Terpenoids	1.070711	84.80000
kz0050433	Scopoletin?Beta-D-Glucuronide	Coumarins	1.069133	117.55000
kz0001188	Sakuranetin	Flavones	1.071266	120.00000
kz0029755	Acacetin-7-O-glucuronide	Flavones	1.071000	120.75000
kz0	wogonoside	Flavon	1.	131.

9			2	
kz0042633	Osthole	Coumarins	1.080000	116.25000
kz0005377	3-Galloylshikimic acid	Phenolic acids	1.081299	116.50000
kz0017400	Quercetin-3-O-(2'',3''-digalloyl)-β-D-glucopyranoside	Flavonols	1.081377	160.25000
kz0001977	E-3,4,5'-Trihydroxy-3'-glucopyranosylstilbene	Others	1.080011	177.00000
kz0036344	Sinapaldehyde Glucoside	Phenolic acids	1.080633	197.25000
kz0010900	Methoxyursolic acid	Terpenoids	1.081811	199.66216
kz0013077	Phthalic acid	Organic acids	1.079699	260.75000
kz0019733	Kaempferol-3-O-(cinnamoyl)-sophoroside-7-O-glucose	Flavonols	1.081766	261.25000
kz0050433	Scopoletin?Beta-D-Glucuronide	Coumarins	1.081255	268.00000
kz0026077	Lariciresinol glucopyranoside	Others	1.081211	291.75000
kz0	Uncargenin D	Terpe	1.	357.

			8	
kz0042333	Terminolic acid	Terpenoids	1.09087	7.06963
kz0052477	Limonin	Others	1.092144	8.29162
kz0010144	1-Methylhistamine	Others	1.087511	11.16236
kz0005000	Riboprine	Phenolic acids	1.091844	11.57954
kz0038722	Lup-12-en-15α,19β-diol-3,11-dioxo-28-oic acid	Terpenoids	1.090833	13.53184
kz0037555	Quercetin-3-sambubioside	Flavonols	1.089833	14.76618
kz0036933	Quercetin-3-O-(2-O-α-L-rhamnopyranosyl)-β-D-galactopyranoside	Flavonols	1.089355	16.11885
kz0013800	Quercetin-O-rutinoside-O-rhamnoside	Flavonols	1.088922	20.11225
kz0001455	Formononetin	Isoflavones	1.092355	21.53398
kz0050377	Caffeoylferuloylshikimic? acid	Phenolic acids	1.089822	25.97845
kz0	6-Hydroxy-7-	Coum	1.	49.2

0934	(Spiraeoside)	ols	07259	2479
kz0000596	β-D-Furanofructosyl-α-D-(6-mustard acyl)glucoside	Phenolic acids	1.07305	60.62500
kz0004425	Luteolin-O-rutinoside-O-rhamnoside	Flavones	1.07024	78.87500
kz0000919	Robinin(Kaempferol-3-O-gal-rham-7-O-rham)	Flavonols	1.06965	90.35000
kz0000500	Riboprine	Phenolic acids	1.07367	91.02961
kz0000733	Delphinidin-3,5-O-diglucoside	Anthocyanins	1.07080	92.20000
kz0004172	Quercetin 3-O-β-D-xylopyranosyl(1→2)-β-D-galactopyranoside	Flavonols	1.06992	116.79114
kz0000278	Indole 3-acetic acid (IAA)	Alkaloids	1.07353	125.75000
kz0003755	Quercetin-3-sambubioside	Flavonols	1.07264	160.57123
kz0000505	Coniferyl alcohol	Phenolic acids	1.07179	199.25000
kz00092	Quercetin-O-rutinoside-hexose	Flavonols	1.0733	224.75000

01695		es	07110	75000
kz0002962	Penduletin (5,4'-Dihydroxy-3,6,7-trimethoxyflavone)	Flavones	1.07119	134.00000
kz0004665	5,7,3',4',5'-pentahydroxydihydroflavone	Flavanones	1.06951	138.50000
kz0004630	2α-hydroxyursolic acid	Terpenoids	1.06256	148.48339
kz0003829	Quercetin 3-O-(6''-galloyl)-β-D-galactopyranoside	Flavonols	1.06919	152.73342
kz0001739	Quercetin-3-O-(2''-galloyl)-β-D-glucoside	Flavonols	1.06418	160.00000
kz0000733	Delphinidin-3,5-O-diglucoside	Anthocyanins	1.06998	163.50000
kz0003106	Cuspinin	Phenolic acids	1.06740	174.50000
kz0000575	3,5-Di-O-galloylshikimic acid	Phenolic acids	1.07100	179.00000
kz0001076	Ethyl gallate	Phenolic acids	1.07067	196.64474
kz00476	monogalloyl-diglucose	Phenolic acids	1.0711	197.25000

03766		noids	08180	00000
kz0000173	3-Hydroxypyridine	Others	1.08163	389.50000
kz0000536	5-Galloylshikimic acid	Phenolic acids	1.08168	397.75000
kz0002197	2'-Hydoxy,5-methoxy Genistein-4',7-O-diglucoside	Isoflavones	1.08054	447.00000
kz0000064	1-O-Feruloyl quinic acid	Phenolic acids	1.08163	496.75000
kz0004595	Geraniin	Phenolic acids	1.08137	502.75000
kz0003504	Geraniinic acid C	Phenolic acids	1.08136	582.25000
kz0001079	Galloyl Methyl gallate	Phenolic acids	1.08160	714.00000
kz0001834	feruloylmalic acid	Phenolic acids	1.08180	733.00000
kz0000886	Myricetin-3-O-arabinoside	Flavonols	1.08149	858.75000
kz00387	Lup-12-en-15α,19β-diol-3,11-dioxo-28-oic acid	Terpenoids	1.0818	1147.50000

03166	methoxycoumarin	arins	09244	0596
kz0004425	Luteolin-O-rutinoside-O-rhamnoside	Flavones	1.08812	78.87500
kz0000919	Robinin(Kaempferol-3-O-gal-rham-7-O-rham)	Flavonols	1.08830	90.35000
kz0001086	2,3-Dihydroxy 5(6),12(13)diene ursolic acid	Terpenoids	1.09230	98.47500
kz0003634	Sinapaldehyde Glucoside	Phenolic acids	1.09139	197.25000
kz0001177	Scoparone	Coumarins	1.09228	232.75000
kz0003797	3-Prenyl-4-O-β-D-glucopyranosyloxy-4-hydroxyl-benzoic acid	Phenolic acids	1.09098	316.75000
kz0001834	feruloylmalic acid	Phenolic acids	1.09236	733.00000
kz0003833	Quercetin-O-pentosyl-O-rhamnoside-O-glucoside	Flavonols	1.09220	934.25000
kz0000551	Methyl 6-O-galloyl-β-D-glucopyranoside	Phenolic acids	1.09245	5045.00000
kz00417	Quercetin 3-O-β-D-xylopyranosyl(1→2)-β-D-galactopyranoside	Flavonols	1.0924	11337.5000

0			2	
kz001307	Phthalic acid	Organic acids	1.07184	260.75000
kz002912	Santamarin	Terpenoids	1.07337	264.50000
kz003797	3-Prenyl-4-O-β-D-glucopyranosyloxy-4-hydroxyl-benzoic acid	Phenolic acids	1.07263	316.75000
kz000055	3,4,5-Trimethoxycinnamic acid	Phenolic acids	1.07396	326.50000
kz000173	3-Hydroxypyridine	Others	1.07369	389.50000
kz002295	Syringaresinol-4'-O-β-D-monO-glucoside	Lignans	1.07310	420.25000
kz000502	Coniferaldehyde	Phenolic acids	1.07358	429.50000
kz004595	Geraniin	Phenolic acids	1.07349	502.75000
kz003504	Geraniinic acid C	Phenolic acids	1.07348	582.25000
kz001834	feruloylmalic acid	Phenolic acids	1.07388	733.00000
kz0	Myricetin-3-O-arabinoside	Flavon	1.	858.
8			0	
kz000064	1-O-Feruloyl quinic acid	Phenolic acids	1.07003	200.50000
kz002960	Eupatilin	Flavones	1.07137	200.50000
kz004595	Geraniin	Phenolic acids	1.07035	208.00000
kz003504	Geraniinic acid C	Phenolic acids	1.07084	232.75000
kz000537	3-Galloylshikimic acid	Phenolic acids	1.07133	248.00000
kz001740	Quercetin-3-O-(2'',3''-digalloyl)-β-D-glucopyranoside	Flavonols	1.06930	307.25000
kz000552	4-O-Caffeoyl quinic acid (criptochlorogenic acid)	Phenolic acids	1.07114	313.25000
kz001584	Quercetin-3′,4′-dimethyl ether	Flavonols	1.07134	331.00000
kz001680	6-hydroxy-5,7,4'-trimethoxyflavone	Flavones	1.07145	353.75000
kz000173	3-Hydroxypyridine	Others	1.07134	379.75000
kz0	E-3,4,5'-Trihydroxy-3'-	Other	1.	380.
2			9	
km1300	Hydroxy-methoxycinnamate	Phenolic acids	1.08190	1187.50000
kz003103	Gemin D	Phenolic acids	1.08162	1392.50000
kz002063	Galloyl-HHDP(Hexahydroxydiphenoyl)-glueopy ranose	Phenolic acids	1.08175	1630.00000
kz000881	Avicularin	Flavonols	1.08180	1932.50000
kz002211	Quercetin 3-O-β-D-xylopyranoside	Flavonols	1.08175	2717.50000
kz000551	Methyl 6-O-galloyl-β-D-glucopyranoside	Phenolic acids	1.08190	5045.00000
kz000532	Protocatechuic acid-4-glucoside	Phenolic acids	1.08192	6242.50000
2			4	0

00886		ols	07360	75000
kz000047	Mandelic acid	Phenolic acids	1.07394	1272.50000
kz000078	1'-O-β-D-(3,4-Dihydroxyphenethyl)-O-caffeoyl-glucoside	Phenolic acids	1.07390	1307.50000
kz001014	1-Methylhistamine	Others	1.07398	1512.50000
km1437	p-Aminobenzoate	Phenolic acids	1.07396	5397.50000
km0936	Quercetin 7-O-β-D-Glucuronide	Flavonols	1.07398	21775.0000

00197	glucopyranosylstilbene	s	07107	25000
kz003083	5,2'-Dihydroxy-7,8-dimethoxyflavone glycosides	Flavones	1.07134	409.50000
kz000059	3-O-p-Coumaroylshikimic acid	Phenolic acids	1.07133	419.00000
kz000886	Myricetin-3-O-arabinoside	Flavonols	1.07125	423.75000
kz001307	Phthalic acid	Organic acids	1.07100	482.25000
kz002607	Lariciresinol glucopyranoside	Others	1.07096	606.50000
kz003103	Gemin D	Phenolic acids	1.07055	745.75000
kz003716	Maplexin D (2,4-Di-O-Galloyl-1,5-Anhydro-D-Glucitol)	Phenolic acids	1.06948	797.00000
kz002063	Galloyl-HHDP(Hexahydroxydiphenoyl)-glueopy ranose	Phenolic acids	1.06890	800.50000
kz002337	Luteolin-7-O-β-D-gentiobioside	Flavones	1.07096	806.25000
kz00537	lyoniresinol-9'-O-β-D-xylopyranoside	Lignans	1.0714	866.00000

7			4	
kz000207	Cannabiscitrin	Others	1.07137	950.75000
kz000612	1,3,4,6-Tetra-O-Galloyl-D-Glucose	Phenolic acids	1.07008	970.00000
kz000536	5-Galloylshikimic acid	Phenolic acids	1.07144	1031.50000
kz002197	2'-Hydoxy,5-methoxy Genistein-4',7-O-diglucoside	Isoflavones	1.07106	1047.25000
kz000032	Caffeoylagmatine	Alkaloids	1.07147	1060.50000
kz000546	1-O-p-Coumaroyl quinic acid	Phenolic acids	1.07142	1182.50000
kz003717	Maplexin C (2,3-Di-O-Galloyl-1,5-Anhydro-D-Glucitol)	Phenolic acids	1.07072	1197.50000
km1300	Hydroxy-methoxycinnamate	Phenolic acids	1.07134	1235.00000
kz000186	2-Deoxyribose 1-phosphate	Others	1.07144	1270.00000
kz001079	Galloyl Methyl gallate	Phenolic acids	1.07131	1875.00000
kz0	Uncargenin D	Terpe	1.	199

03766		noids	07138	0.00000		
kz0038823	Kampferol 3-O-(2”-galloyl)-β-D-galactopyranoside	Flavonols	1.07144	2525.00000		
kz0037748	7'-O-Sinapoyljasminoside L	Terpenoids	1.07099	4315.00000		
kz000530	Brevifolin carboxylic acid	Phenolic acids	1.07098	5310.00000		
kz000532	Protocatechuic acid-4-glucoside	Phenolic acids	1.07140	11250.00000		
kz000881	Avicularin	Flavonols	1.07146	15925.00000		
kz002211	Quercetin 3-O-β-D-xylopyranoside	Flavonols	1.07142	18225.00000		

Table S3. The proportion of classified metabolites of five *Rosa* fruits respectively.

Class	RRT-F	RSS-F	RLM-F	RDP-F	RSL-F
Flavonoids	34.04%	22.35%	45.14%	41.26%	35.13%
Phenolic acids	18.08%	14.33%	16.05%	16.06%	12.35%
Organic acids	16.17%	15.95%	14.59%	13.32%	16.33%
Terpenoids	11.38%	22.62%	7.57%	13.21%	17.18%
Alkaloids	4.89%	9.37%	5.10%	3.96%	5.10%
Lignans	4.06%	1.50%	1.29%	1.30%	0.59%
Vitamins	0.82%	0.87%	0.99%	0.68%	0.79%
Quinones	0.02%	0.01%	0.02%	0.03%	0.00%
Coumarins	0.27%	0.26%	0.46%	0.13%	0.53%
Others	9.43%	10.62%	8.61%	7.04%	10.12%

Table S4. List of relative content of differential metabolites (DEMs) among five *Rosa* fruits.

Compounds	Class	RRT-F		RSS-F		RLM-F		RDP-F		RSL-F	
		mean value	Proportion of peak area(%)	mean value	Proportion of peak area(%)	mean value	Proportion of peak area(%)	mean value	Proportion of peak area(%)	mean value	Proportion of peak area(%)
Quercetin 3-O-galactoside (Hyperin)	Flavonoids	7252500	1.6994±0.8623	1880000	0.6004±0.3025	2065000	0.5946±0.3488	5190000	0.8572±0.216	2435000	0.5761±0.3093
Biochanin A	Flavonoids	1473	0.0003±0.0006	4890	0.0016±0.001	1645	0.0005±0.0001	30825	0.0051±0.0009	1875	0.0004±0.0007
Genistein (4',5,7-Trihydroxyisoflavone)	Flavonoids	15550	0.0036±0.0024	6593	0.0021±0.0037	17400	0.005±0.0051	43825	0.0072±0.0113	42850	0.0101±0.014
Orobol (5,7,3',4'-tetrahydroxyisoflavone)	Flavonoids	47575	0.0111±0.0335	54525	0.0175±0.0405	95575	0.0275±0.0442	297750	0.0491±0.1117	158500	0.0375±0.0884
Quercetin 3-O-glucoside (Isotrifoliin)	Flavonoids	6542500	1.534±0.5425	1757500	0.561±0.3926	1990000	0.5727±0.0298	4802500	0.7927±0.2945	2337500	0.5534±0.1776
2'-Hydroxygenistein	Flavonoids	44175	0.0103±0.0249	40625	0.013±0.0409	85400	0.0246±0.0817	269500	0.0446±0.0698	124175	0.0295±0.0749
Apigenin	Flavonoids	15125	0.0036±0.0041	6663	0.0021±0.0026	13950	0.004±0.0048	39825	0.0066±0.0067	37275	0.0088±0.0127
Chrysoeriol	Flavonoids	14975	0.0035±0.0034	7948	0.0025±0.003	8305	0.0024±0.0021	27000	0.0045±0.0009	3990	0.0009±0.0016
(+)-cis,trans-Abscisic acid (ABA)	Others	1340000	0.314±0.1224	926250	0.2958±0.0727	589000	0.1695±0.0196	576500	0.0952±0.0341	684750	0.1621±0.0414
3-(4-Hydroxyphenyl)propionic acid	Phenolic acids	782000	0.1833±0.0449	210750	0.0673±0.062	37275	0.0107±0.0097	309000	0.051±0.0228	314000	0.0743±0.0435
Afzelechin (3,5,7,4'-Tetrahydroxyflavan)	Flavonoids	409500	0.0961±0.0685	252500	0.0806±0.0054	583750	0.1681±0.0653	860750	0.1421±0.0489	6808	0.0016±0.0022
Delphinidin 3-O-glucoside (Mirtillin)	Flavonoids	1147750	0.2679±0.3194	113050	0.0361±0.0781	40975	0.0118±0.0436	237500	0.0392±0.0978	62700	0.0149±0.0628
Dihydroquercetin (Taxifolin)	Flavonoids	354500	0.0831±0.0359	104700	0.0335±0.0389	876250	0.2522±0.0598	943250	0.1558±0.0586	46125	0.0109±0.0132
Epicatechin gallate (ECG)	Flavonoids	29600	0.0069±0.0178	26400	0.0085±0.0231	68300	0.0196±0.0249	322250	0.0532±0.0069	14025	0.0033±0.0093
Genistein 7-O-Glucoside (Genistin)	Flavonoids	31950	0.0075±0.0158	69250	0.0222±0.0505	299750	0.0863±0.0529	71575	0.0118±0.0104	22650	0.0054±0.0164
Hesperetin 7-rutinoside (Hesperidin)	Flavonoids	299500	0.0699±0.077	91250	0.0291±0.0185	-	-	-	-	-	-
Kaempferol 3-O-galactoside (Trifolin)	Flavonoids	7455000	1.7476±0.7386	3147500	1.0051±0.3998	6052500	1.7407±0.9784	12025000	1.9852±0.6144	8360000	1.9785±1.4768
Kaempferol 3-O-rhamnoside (Kaempferin)	Flavonoids	155750	0.0366±0.0294	63900	0.0204±0.0195	45725	0.0132±0.0113	46925	0.0078±0.0031	40150	0.0095±0.0102

Luteolin 7-O-glucoside	Flavonoids	4940000	1.1554±1.2535	2180000	0.6957±0.4241	4135000	1.189±0.9631	8965000	1.4798±0.5194	6050000	1.4327±0.5167
Morin	Flavonoids	1955000	0.4583±0.1648	525750	0.1679±0.0195	537500	0.1547±0.0693	1930000	0.3186±0.0998	1890000	0.4477±0.2085
Myricetin 3-O-rhamnoside (Myricitrin)	Flavonoids	225750	0.0532±0.108	64125	0.0205±0.031	21400	0.0062±0.0081	-	-	-	-
Naringenin	Flavonoids	295750	0.0693±0.03	254250	0.0812±0.0311	171750	0.0494±0.0095	647250	0.1069±0.0168	177750	0.0421±0.0141
Naringenin chalcone	Flavonoids	324250	0.0761±0.0461	287750	0.0919±0.0153	187000	0.0538±0.0161	701750	0.1158±0.0355	197000	0.0467±0.0177
Orotic acid	Vitamins	259750	0.0609±0.0464	656500	0.2094±0.2755	252750	0.0728±0.024	443250	0.0732±0.0209	1445000	0.3421±0.3686
Quercetin 4'-O-glucoside (Spiraeoside)	Flavonoids	283750	0.0664±0.0356	146250	0.0466±0.0786	200500	0.0577±0.0332	1222500	0.2019±0.0482	8530000	2.019±1.3585
Quercetin 7-O-β-D-Glucuronide	Flavonoids	-	-	-	-	15575000	4.4845±2.4246	5057500	0.835±0.3103	2177500	0.5156±0.1911
5-O-p-Coumaroyl shikimic acid	Phenolic acids	105050	0.0246±0.0146	21800	0.007±0.0117	14075	0.0041±0.008	29850	0.0049±0.0091	8045	0.0019±0.0077
Chrysoeriol 7-O-hexoside	Flavonoids	144250	0.0339±0.0497	98925	0.0316±0.0301	68700	0.0198±0.0328	181500	0.03±0.021	33575	0.0079±0.0282
Di-O-methylquercetin	Flavonoids	494250	0.1159±0.0792	144750	0.0462±0.0077	211000	0.0607±0.0139	1407500	0.2324±0.032	1092500	0.2586±0.0356
Hesperetin O-malonylhexoside	Flavonoids	406500	0.0952±0.0391	126500	0.0404±0.0265	11100	0.0032±0.0031	195750	0.0323±0.0169	128500	0.0304±0.0188
Hydroxy-methoxycinnamate	Phenolic acids	47700	0.0112±0.0113	46925	0.015±0.0058	-	-	123500	0.0204±0.0139	118750	0.0281±0.0072
Isorhamnetin 5-O-hexoside	Flavonoids	1142500	0.2672±0.162	360000	0.115±0.1226	1330000	0.3829±0.2262	1352500	0.2233±0.1205	106000	0.0251±0.0114
Nicotinic acid-hexoside	Vitamins	236500	0.0553±0.0375	85200	0.0272±0.0278	342500	0.0985±0.0277	499250	0.0824±0.0387	39075	0.0093±0.017
p-Aminobenzoate	Phenolic acids	133000	0.0312±0.0475	-	-	295500	0.0851±0.0359	741000	0.1223±0.0209	539750	0.1278±0.0909
Caffeoylagmatine	Alkaloids	30400	0.0071±0.0037	12003	0.0038±0.0076	-	-	106050	0.0175±0.011	-	-
N,N'-Bis(Sinapoyl)Spermidine	Alkaloids	223750	0.0526±0.058	53150	0.017±0.0144	41375	0.0119±0.0256	101425	0.0167±0.0561	5445	0.0013±0.0043
Mandelic acid	Phenolic acids	287750	0.0675±0.0363	-	-	43175	0.0124±0.0265	182750	0.0302±0.0417	127250	0.0302±0.0219
2-(Formylamino)benzoic acid	Phenolic acids	1165000	0.2729±0.0719	185500	0.0592±0.0512	312250	0.0899±0.0304	636000	0.1051±0.0424	786250	0.1861±0.0262
Homogentisic acid	Phenolic acids	46675	0.0108±0.0446	120500	0.0385±0.0393	57975	0.0167±0.0289	98200	0.0162±0.0098	-	-

4-Methoxycinnamic acid	Phenolic acids	189250	0.0443±0.0053	100975	0.0323±0.017	162500	0.0468±0.0057	334750	0.0553±0.0077	251750	0.0596±0.0204
Ethyl 3,4-Dihydroxybenzoate (Ethyl protocatechuate)	Phenolic acids	59625	0.014±0.0065	8043	0.0026±0.007	5538	0.0016±0.0017	399000	0.0659±0.0192	15050	0.0036±0.0062
Isoferulic Acid	Phenolic acids	631000	0.1477±0.0691	179750	0.0574±0.0244	458500	0.1319±0.0422	339750	0.0561±0.0167	816000	0.1932±0.0626
3,4,5-Trimethoxycinnamic acid	Phenolic acids	57800	0.0135±0.0052	-	-	2830	0.0008±0.0005	5323	0.0009±0.0011	32650	0.0077±0.002
Salicylic acid glucoside	Phenolic acids	192750	0.0452±0.0207	178000	0.0569±0.021	454250	0.1308±0.0476	352500	0.0582±0.0341	69850	0.0165±0.006
Protocatechuic acid O-glucoside	Phenolic acids	944250	0.2218±0.2184	1127500	0.3601±0.1358	823000	0.2369±0.1441	657000	0.1085±0.0851	49000	0.0116±0.0179
3-O-p-Coumaroylshikimic acid	Phenolic acids	54900	0.0129±0.012	161500	0.0516±0.0295	-	-	41900	0.0069±0.0074	10768	0.0025±0.0057
1-O-Feruloyl quinic acid	Phenolic acids	49350	0.0115±0.0133	158000	0.0505±0.0379	-	-	20050	0.0033±0.0078	49675	0.0118±0.0147
Feruloyl syringic acid	Phenolic acids	48125	0.0113±0.0105	7985	0.0025±0.0093	124500	0.0359±0.0276	33650	0.0056±0.0175	24425	0.0058±0.0108
Catechin gallate	Phenolic acids	38125	0.0089±0.0133	33100	0.0106±0.0225	63975	0.0184±0.0142	325250	0.0537±0.019	12663	0.003±0.0049
Anthranilate O-hexosyl-O-hexoside	Phenolic acids	489000	0.1147±0.1281	655500	0.2095±0.2932	164750	0.0474±0.0187	824500	0.1362±0.0291	281250	0.0665±0.0414
1'-O-β-D-(3,4-Dihydroxyphenethyl)-O-caffeoyl-glucoside	Phenolic acids	-	-	-	-	903000	0.26±0.1397	288000	0.0476±0.057	130750	0.031±0.0182
Sakuranetin	Flavonoids	3550	0.0008±0.0008	35925	0.0115±0.0056	-	-	12000	0.002±0.0014	6413	0.0015±0.0009
Chrysin 5-O-glucoside (Toringin)	Flavonoids	54975	0.0129±0.0089	68550	0.0219±0.0142	38425	0.0111±0.0107	158250	0.0261±0.0096	25050	0.0059±0.0008
Formononetin	Flavonoids	150750	0.0353±0.0091	325750	0.104±0.0131	31225	0.009±0.0071	128750	0.0213±0.009	2772500	0.6565±0.187
Methylquercetin O-hexoside	Flavonoids	781750	0.1831±0.0754	274250	0.0875±0.0494	936000	0.2692±0.1601	932500	0.1539±0.1287	74400	0.0176±0.0228
7-O-Methyleriodictyol	Flavonoids	3080	0.0007±0.0011	48950	0.0156±0.0073	-	-	4800	0.0008±0.0015	-	-
Naringenin 7-O-glucoside (Prunin)	Flavonoids	650000	0.1524±0.1058	1425000	0.4551±0.1628	759250	0.2184±0.111	2740000	0.4528±0.2107	431500	0.1021±0.0284
3-Hydroxypyridine	Others	-	-	-	-	-	-	37975	0.0063±0.0039	38950	0.0092±0.0124
2-Deoxyribose 1-phosphate	Others	44375	0.0104±0.0097	69900	0.0223±0.0157	-	-	127000	0.021±0.0126	-	-
Arbutin	Others	16850	0.004±0.0046	11600	0.0037±0.0034	138000	0.0397±0.007	86275	0.0143±0.0084	18025	0.0043±0.006

E-3,4,5'-Trihydroxy-3'-glucopyranosylstilbene	Others	4638	0.0011±0.002	3450	0.0011±0.004	-	-	38025	0.0063±0.007	17700	0.0042±0.0101
D-galacitol	Others	831750	0.195±0.0576	804250	0.2568±0.0428	394250	0.1135±0.0231	2810000	0.464±0.0366	2492500	0.5902±0.1105
Cannabiscitrin	Others	-	-	-	-	-	-	95075	0.0157±0.0129	-	-
5-Aminolevulinate	Alkaloids	353000	0.0827±0.0312	908000	0.2901±0.1628	366750	0.1055±0.0543	1325000	0.2188±0.0486	1480000	0.3486±1.549
O-Phosphocholine	Alkaloids	364250	0.0854±0.0408	1205000	0.385±0.1699	493750	0.1422±0.0949	371000	0.0612±0.0349	343000	0.0813±0.0531
Trans-Zeatin-9-N-Glucoside	Alkaloids	107250	0.0251±0.0116	117750	0.0376±0.0242	237750	0.0684±0.0254	606000	0.1±0.0844	74650	0.0177±0.0015
Nicotinate D-ribonucleoside	Vitamins	658500	0.1544±0.1009	256250	0.0818±0.0529	686750	0.1977±0.0896	934750	0.1543±0.0615	630000	0.1491±0.0354
Thiamine	Vitamins	23775	0.0056±0.0053	17625	0.0056±0.0055	10743	0.0031±0.0042	61000	0.0101±0.0108	45800	0.0108±0.0139
N-sinapoylhydroxycoumarin	Coumarins	66450	0.0155±0.0126	98750	0.0315±0.0342	77925	0.0224±0.0182	38675	0.0064±0.0074	152500	0.0361±0.0429
4-Aminoindole	Alkaloids	73800	0.0173±0.033	36275	0.0116±0.0204	-	-	-	-	-	-
Indole 3-acetic acid (IAA)	Alkaloids	-	-	-	-	12750	0.0037±0.0058	35300	0.0058±0.0049	12575	0.003±0.0028
3-Hydroxypropanoic acid	Organic acids	129250	0.0303±0.0295	94950	0.0303±0.0125	105500	0.0304±0.0071	157500	0.026±0.0116	34325	0.0081±0.0093
Aminomalonic acid	Organic acids	86675	0.0203±0.0199	58850	0.0188±0.0123	91600	0.0264±0.0116	185000	0.0305±0.016	239750	0.0568±0.019
2-Picolinic acid	Organic acids	229750	0.054±0.0508	149250	0.0477±0.0053	393500	0.1132±0.0194	151250	0.025±0.0112	114250	0.027±0.0101
Glutaric acid	Organic acids	456000	0.1068±0.0359	166750	0.0532±0.0727	305750	0.0881±0.0906	321750	0.0532±0.0387	781750	0.182±2.6587
2-Hydroxyisocaproic acid	Organic acids	45225	0.0106±0.0159	85650	0.0274±0.0111	14850	0.0043±0.003	74050	0.0122±0.0081	41875	0.0099±0.0089
Citramalate	Organic acids	51075	0.012±0.0174	81450	0.026±0.0144	240750	0.0693±0.0113	73350	0.0121±0.0137	45375	0.0107±0.0212
DL-Glyceraldehyde 3-phosphate	Organic acids	47750	0.0112±0.015	64300	0.0205±0.0248	32425	0.0093±0.0071	90150	0.0149±0.0156	34650	0.0082±0.0089
2-Isopropylmalate	Organic acids	57325	0.0134±0.0244	53375	0.017±0.0668	37175	0.0107±0.0038	556750	0.0918±0.1513	192250	0.0456±0.0438
(±)-jasmonic acid	Organic acids	79950	0.0187±0.0054	620250	0.1981±0.0351	10080	0.0029±0.0035	6478	0.0011±0.0009	12250	0.0029±0.0005
Methyl jasmonate	Organic acids	5160	0.0012±0.0027	43950	0.014±0.0071	-	-	-	-	-	-

N-[(-)-Jasmonoyl]-(L)-Isoleucine (JA-L-Ile)	Organic acids	15775	0.0037±0.0017	35075	0.0112±0.001	1385	0.0004±0.0003	2075	0.0003±0.0003	2150	0.0005±0.0001
Pyruvic acid	Organic acids	277500	0.065±0.0499	94825	0.0303±0.0301	101225	0.0291±0.0287	93875	0.0155±0.0176	186500	0.0442±0.0339
A-Ketoglutaric acid	Organic acids	534500	0.1252±0.0261	341750	0.1092±0.0514	744500	0.2142±0.1774	749000	0.1236±0.0283	450500	0.1068±0.1137
Sweroside	Terpenoids	276250	0.0647±0.0677	251000	0.0802±0.0518	66550	0.0191±0.0246	270000	0.0446±0.023	537000	0.1272±0.0316
Aucubin	Terpenoids	40900	0.0096±0.0066	14550	0.0046±0.0032	187250	0.0539±0.0108	131750	0.0217±0.0042	30600	0.0072±0.0068
2-Picolylamine	Alkaloids	67075	0.0157±0.0107	32750	0.0105±0.0149	46050	0.0133±0.0266	66175	0.011±0.0143	28975	0.0069±0.0076
Phloretin	Flavonoids	16050	0.0038±0.0039	5340	0.0017±0.0033	5565	0.0016±0.0037	57650	0.0095±0.0019	2918	0.0007±0.0012
Phlorizin	Flavonoids	9030	0.0021±0.0043	16200	0.0052±0.0077	13150	0.0038±0.0102	105875	0.0175±0.0125	-	-
Butin	Flavonoids	216750	0.0508±0.0187	180750	0.0577±0.0133	121500	0.035±0.0089	427250	0.0705±0.0161	123250	0.0292±0.006
Eriodictyol	Flavonoids	269000	0.063±0.0421	62750	0.02±0.0043	65000	0.0187±0.0043	813000	0.1343±0.0256	32975	0.0078±0.0076
Eriodictyol 7-O-glucoside	Flavonoids	92450	0.0216±0.0157	98550	0.0315±0.0459	55700	0.016±0.0023	516750	0.0854±0.0552	18400	0.0043±0.0094
Pinobanksin	Flavonoids	312500	0.0733±0.0509	282000	0.0901±0.0152	184500	0.0531±0.0056	671750	0.1109±0.0226	196750	0.0466±0.0122
Astilbin	Flavonoids	179000	0.0419±0.0112	780750	0.2493±0.1092	47650	0.0137±0.0193	787000	0.1299±0.0193	15550	0.0037±0.008
Hesperetin 5-O-glucoside	Flavonoids	12375000	2.901±1.004	4840000	1.5455±0.2133	5717500	1.6465±0.9934	12825000	2.1185±0.6449	6267500	1.4844±0.6799
4-Hydroxybenzaldehyde	Phenolic acids	104025	0.0243±0.017	84525	0.0271±0.0656	409000	0.1178±0.0811	452250	0.0746±0.0382	1925000	0.4557±0.1785
4-Hydroxybenzoic acid	Phenolic acids	1280000	0.3±0.0804	802500	0.2562±0.2033	940000	0.2708±0.3608	3940000	0.6503±0.1867	5207500	1.2321±0.9969
Tyrosol	Phenolic acids	6978	0.0016±0.0024	7798	0.0025±0.0069	10523	0.003±0.0101	22100	0.0036±0.0056	19975	0.0047±0.0115
Cinnamic acid	Phenolic acids	97125	0.0228±0.0072	462250	0.1476±0.0274	35950	0.0104±0.0058	6965	0.0012±0.0013	34125	0.0081±0.0063
Vanillin	Phenolic acids	1545000	0.3619±0.0955	309000	0.0989±0.2314	1960000	0.5644±0.2418	4932500	0.8143±0.1783	4677500	1.1078±0.3783
2,5-Dihydroxybenzoic acid	Phenolic acids	1700000	0.3976±0.2691	2767500	0.8836±0.1239	492250	0.1417±0.0765	2222500	0.3667±0.1233	1885000	0.4465±0.1361
Protocatechuic acid	Phenolic acids	6875000	1.6091±0.7324	11300000	3.6087±0.8069	1850000	0.5323±0.3054	8780000	1.4489±0.3573	7907500	1.8729±0.99

p-Coumaric acid	Phenolic acids	675000	0.1584±0.1321	100225	0.032±0.0187	156250	0.045±0.0312	319250	0.0527±0.0368	403500	0.0955±0.0605
3-(4-Hydroxyphenyl)-propionic acid	Phenolic acids	845000	0.1977±0.0761	227750	0.0728±0.0701	40050	0.0115±0.0167	333250	0.0549±0.0454	311250	0.0737±0.0343
Vanillic acid	Phenolic acids	491250	0.1152±0.0323	127000	0.0405±0.0324	586750	0.1688±0.059	1041500	0.172±0.0649	1345000	0.3185±0.0746
Riboprine	Phenolic acids	8150	0.0019±0.0042	5658	0.0018±0.0013	20650	0.0059±0.0027	44475	0.0074±0.0034	515000	0.1219±0.062
Methyl p-coumarate	Phenolic acids	212750	0.0498±0.0162	93350	0.0298±0.0151	191500	0.0551±0.0256	349500	0.0578±0.037	196500	0.0465±0.0126
Coniferaldehyde	Phenolic acids	15025	0.0035±0.0039	-	-	69925	0.0201±0.0175	60700	0.01±0.0111	42950	0.0102±0.013
Caffeic acid	Phenolic acids	1001250	0.2342±0.1029	173000	0.0553±0.086	137750	0.0396±0.0429	531250	0.0877±0.0358	115000	0.0272±0.0223
Coniferyl alcohol	Phenolic acids	3535	0.0008±0.0006	-	-	50200	0.0145±0.0042	59025	0.0097±0.012	19925	0.0047±0.0122
Syringic Aldehyde	Phenolic acids	140500	0.0329±0.0128	15500	0.005±0.0146	261500	0.0752±0.0353	367500	0.0607±0.0547	451000	0.1068±0.0569
Ferulic acid	Phenolic acids	620250	0.1453±0.0721	191500	0.0612±0.0427	430750	0.124±0.0746	344000	0.0568±0.0367	829000	0.1963±0.0367
Syringic acid	Phenolic acids	186750	0.0438±0.0112	88725	0.0284±0.0322	216750	0.0624±0.0084	313500	0.0518±0.0251	226000	0.0535±0.0433
3,4-Dimethoxycinnamic acid	Phenolic acids	58200	0.0136±0.006	20450	0.0065±0.0033	45325	0.0131±0.0049	52100	0.0086±0.0026	55575	0.0132±0.0066
Sinapinaldehyde	Phenolic acids	11975	0.0028±0.0018	4783	0.0015±0.003	25025	0.0072±0.0069	37625	0.0062±0.002	215000	0.0509±0.0312
Sinapyl alcohol	Phenolic acids	-	-	-	-	48350	0.0139±0.0181	117500	0.0194±0.0173	-	-
Sinapic acid	Phenolic acids	36925	0.0087±0.0158	27500	0.0088±0.0121	36450	0.0105±0.0213	63950	0.0105±0.014	19700	0.0047±0.0048
Methyl sinapate	Phenolic acids	10745	0.0025±0.0039	7420	0.0024±0.0036	9365	0.0027±0.0023	15125	0.0025±0.0026	2198	0.0005±0.0014
Brevifolin carboxylic acid	Phenolic acids	3030000	0.7059±1.4229	467250	0.1495±0.4567	-	-	531000	0.0875±0.1568	-	-
Protocatechuic acid-4-glucoside	Phenolic acids	608000	0.1423±0.0696	275250	0.0879±0.0699	-	-	1125000	0.1858±0.1253	624250	0.1478±0.0589
2,5-Dihydroxy benzoic acid O-hexside	Phenolic acids	27725	0.0065±0.0056	24525	0.0079±0.0427	112250	0.0323±0.014	351250	0.0581±0.1179	104800	0.0248±0.0405
5-Galloylshikimic acid	Phenolic acids	1134000	0.2654±0.1955	26850	0.0086±0.0073	-	-	103150	0.017±0.0096	39775	0.0094±0.0074
3-Galloylshikimic acid	Phenolic acids	264250	0.0618±0.0369	6845	0.0022±0.0035	-	-	24800	0.0041±0.0023	11650	0.0028±0.0044

3-Hydroxy-4-isopropylbenzylalcohol 3-glucoside	Phenolic acids	60375	0.0142±0.0117	78925	0.0252±0.0217	24900	0.0072±0.0073	163000	0.0269±0.0138	44325	0.0105±0.0064
3,4,5-Trimethoxyphenyl-β-D-Glucopyranoside	Phenolic acids	1237500	0.2904±0.2192	459500	0.1468±0.061	7410000	2.1325±0.5203	2117500	0.3496±0.0704	692500	0.1641±0.0822
1-O-Galloyl-β-D-glucose	Phenolic acids	1217500	0.285±0.1452	1922500	0.614±0.0549	3485000	1.0027±0.2093	4240000	0.7±0.1678	2970000	0.7033±0.1678
3-O-p-Coumaroyl quinic acid	Phenolic acids	59200	0.0139±0.016	585000	0.1869±0.1194	21000	0.0061±0.0116	883750	0.1458±0.0816	619250	0.1465±0.0802
1-O-p-Coumaroyl quinic acid	Phenolic acids	90375	0.0212±0.016	90500	0.0289±0.029	-	-	118250	0.0195±0.0155	-	-
Trans-3-O-p-coumaric quinic acid	Phenolic acids	303500	0.0709±0.0906	1960000	0.6267±0.8144	77575	0.0223±0.0054	994000	0.1644±0.2241	509500	0.1207±0.0906
1-O-[(E)-Caffeoyl]-β-D-glucopyranose	Phenolic acids	688250	0.1611±0.117	646250	0.2064±0.0805	384750	0.1107±0.0734	912500	0.1509±0.1313	309500	0.0733±0.0443
Coniferin	Phenolic acids	717250	0.1683±0.1479	667250	0.2132±0.1223	395750	0.1139±0.0418	913750	0.1509±0.0468	328000	0.0777±0.0313
Methyl 6-O-galloyl-β-D-glucopyranoside	Phenolic acids	-	-	108250	0.0346±0.0199	-	-	-	-	504500	0.1194±0.0595
4-O-Caffeoyl quinic acid (criptochlorogenic acid)	Phenolic acids	49575	0.0116±0.012	12923	0.0041±0.0194	-	-	31325	0.0052±0.0064	-	-
Neochlorogenic acid(5-O-Caffeoylquinic acid)	Phenolic acids	206750	0.0484±0.0557	84200	0.0269±0.0307	15200	0.0044±0.0087	104100	0.0172±0.0123	-	-
Chlorogenic acid	Phenolic acids	363000	0.0851±0.09	310500	0.0991±0.0647	50850	0.0146±0.0091	201750	0.0333±0.0184	73125	0.0173±0.0123
1-Caffeoylquinic acid	Phenolic acids	165000	0.0386±0.0283	64875	0.0207±0.0096	59825	0.0172±0.033	78300	0.0129±0.01	-	-
Glucosyringic Acid	Phenolic acids	131250	0.0308±0.0125	80300	0.0256±0.0115	1002250	0.2885±0.1027	280500	0.0463±0.0119	47900	0.0113±0.0171
Trihydroxycinnamoylquinic acid	Phenolic acids	365000	0.0854±0.053	404750	0.1293±0.0956	322250	0.0927±0.0392	1712500	0.2828±0.1359	106850	0.0253±0.0193
Ditartaroyl-hydroxylcoumarin	Phenolic acids	160250	0.0375±0.0129	103800	0.0332±0.026	104650	0.0301±0.0223	216750	0.0358±0.0222	133500	0.0316±0.025
p-Coumaroylferuloyltartaric acid	Phenolic acids	149000	0.0349±0.0088	35200	0.0113±0.0115	22475	0.0065±0.0057	226000	0.0373±0.0069	52150	0.0123±0.0093
3,5-Di-O-galloylshikimic acid	Phenolic acids	24150	0.0057±0.0118	-	-	-	-	17900	0.003±0.0046	-	-
Hexahydroxydiphenoylglucose	Phenolic acids	8770000	2.0557±1.7551	711750	0.2272±0.0946	1927500	0.5546±0.1493	850000	0.1403±0.0162	883500	0.2091±0.0544
1,6-Di-O-Galloyl-D-Glucose	Phenolic acids	-	-	53025	0.0169±0.028	536250	0.1544±0.1186	64100	0.0106±0.0192	33000	0.0078±0.0268
5-O-p-Coumaroyl quinic acid O-hexoside	Phenolic acids	12600	0.003±0.002	34425	0.011±0.0034	11970	0.0035±0.0087	87100	0.0144±0.0146	4930	0.0012±0.0046

Rosmarinyl Glucoside	Phenolic acids	37425	0.0088±0.0225	53150	0.017±0.0393	418750	0.1206±0.0685	147000	0.0243±0.0334	-	-
β-D-Furanofructosyl-α-D-(6-mustard acyl)glucoside	Phenolic acids	57475	0.0134±0.0127	-	-	25725	0.0074±0.0119	35475	0.0059±0.0023	6063	0.0014±0.0022
1,3,4,6-Tetra-O-Galloyl-D-Glucose	Phenolic acids	35075	0.0082±0.01	31125	0.01±0.0178	-	-	97000	0.0159±0.0469	-	-
1,2,3,6-Tetra-O-Galloyl-D-Glucose	Phenolic acids	221750	0.0519±0.0477	57675	0.0184±0.033	12750	0.0037±0.0086	221750	0.0366±0.0243	33325	0.0079±0.0213
Cyanidin-3-O-galactoside	Flavonoids	78525	0.0184±0.0353	18275	0.0058±0.0146	24575	0.0071±0.0154	54125	0.009±0.0175	56200	0.0133±0.0128
Cyanidin-3-O-glucoside (Kuromanin)	Flavonoids	75425	0.0176±0.0216	16150	0.0052±0.0098	21025	0.006±0.0188	33600	0.0056±0.0193	51675	0.0123±0.0298
Delphinidin-3-O-glucoside (Mirtillin)	Flavonoids	1307500	0.3053±0.4134	176500	0.0564±0.0418	87975	0.0252±0.097	341500	0.0563±0.1866	-	-
Cyanidin-O-syringic acid	Flavonoids	401750	0.0941±0.026	393750	0.1257±0.0389	408750	0.1177±0.0398	492500	0.0813±0.0249	-	-
Delphinidin-3,5-O-diglucoside	Flavonoids	-	-	-	-	-	-	16350	0.0027±0.0055	9220	0.0022±0.0072
Diosmetin	Flavonoids	26475	0.0062±0.0043	15050	0.0048±0.0063	19250	0.0055±0.0018	50700	0.0084±0.0047	8430	0.002±0.0026
Apigenin 7-O-glucoside(Cosmosiin)	Flavonoids	24475	0.0058±0.0063	75550	0.0241±0.0204	254500	0.0732±0.043	74750	0.0123±0.0195	25600	0.0061±0.0118
Apigenin 5-O-glucoside	Flavonoids	41225	0.0097±0.0148	99100	0.0317±0.0193	399500	0.115±0.0544	95350	0.0157±0.0183	30700	0.0073±0.0044
Luteolin-7-O-glucoside(Cynaroside)	Flavonoids	6285000	1.4714±2.0458	2542500	0.8117±0.2	5360000	1.5431±1.3243	10925000	1.8036±0.368	7795000	1.8452±0.354
Tetahydroxy-flavone-7-O-β-D-glucuronide	Flavonoids	57525	0.0134±0.0173	24325	0.0078±0.0098	3380000	0.9724±0.4129	971250	0.1604±0.0449	927750	0.2197±0.0203
Luteolin-7-O-glucuronide	Flavonoids	72400	0.0169±0.0118	26875	0.0086±0.0155	3127500	0.9004±0.3345	965500	0.1594±0.0336	853750	0.2022±0.0993
Chrysoeriol-5-O-hexoside	Flavonoids	2985000	0.7017±0.8853	1145000	0.3656±0.1654	1365000	0.3928±0.0212	3000000	0.4954±0.1914	2130000	0.5045±0.1964
Tricin O-saccharic acid	Flavonoids	627250	0.1469±0.0717	210500	0.0673±0.0514	390500	0.1124±0.0397	1055000	0.1742±0.0538	637250	0.151±0.1393
Chrysoeriol-7-O-rutinoside	Flavonoids	622000	0.1458±0.0891	435250	0.1391±0.0702	14975	0.0043±0.0102	15675	0.0026±0.0043	15175	0.0036±0.0049
Diosmin	Flavonoids	1280000	0.2996±0.1235	611000	0.1951±0.04	19450	0.0056±0.0094	23225	0.0038±0.006	18200	0.0043±0.0043
Luteolin-O-sinapoylhexoside	Flavonoids	24425	0.0057±0.0039	4120	0.0013±0.001	-	-	4510	0.0008±0.0017	2793	0.0007±0.0013
Kaempferol	Flavonoids	86550	0.0203±0.0032	18200	0.0058±0.0071	16725	0.0048±0.0048	28825	0.0048±0.005	80425	0.0191±0.0069

Quercetin	Flavonoids	179500	0.042±0.0146	70050	0.0224±0.026	136250	0.0393±0.0773	269250	0.0444±0.0568	208000	0.0493±0.0215
Myricetin	Flavonoids	-	-	-	-	132500	0.0382±0.0243	15300	0.0025±0.0024	-	-
Avicularin	Flavonoids	1647500	0.3858±0.1438	1397500	0.4461±0.2096	-	-	1592500	0.2628±0.0809	193250	0.0458±0.0487
Kaempferol-3-O-glucoside (Astragalin)	Flavonoids	9242500	2.1617±1.2651	3555000	1.1356±0.5663	5887500	1.6945±1.2457	12050000	1.9898±1.0731	8607500	2.0363±1.2819
Myricetin-3-O-arabinoside	Flavonoids	16718	0.0039±0.0138	-	-	-	-	42375	0.007±0.0074	85875	0.0203±0.0303
Quercetin-3-O-β-D-Galactoside (Hyperin)	Flavonoids	2200000	0.5152±0.2179	792750	0.2531±0.1144	841500	0.2422±0.1865	1997500	0.3298±0.0711	1094750	0.259±0.1119
Spiraeoside	Flavonoids	12450000	2.921±1.9763	4995000	1.5948±0.4708	5857500	1.6862±0.7176	12450000	2.0562±0.3932	6220000	1.4734±0.6764
Quercetin-7-O-Glucoside	Flavonoids	235750	0.0553±0.0746	133750	0.0427±0.0816	190000	0.0547±0.0242	1137500	0.1877±0.0409	7635000	1.8075±0.6279
6-Hydroxykaempferol-7-O-glucoside	Flavonoids	6770000	1.5855±0.4275	1685000	0.538±0.233	1957500	0.5636±0.3057	4647500	0.7667±0.4116	2340000	0.5536±0.4187
Tiliroside	Flavonoids	2932500	0.6866±0.4492	3192500	1.0191±0.3729	6352500	1.8279±0.5866	18375000	3.0341±0.8871	9890000	2.3417±0.3655
Kaempferol-3-O-rutinoside(Nicotiflorin)	Flavonoids	267000	0.0626±0.032	607000	0.194±0.1513	7642500	2.1985±0.7128	765750	0.1264±0.0554	1835000	0.4345±0.1481
Kaempferol-3-O-robinobioside(Biorobin)	Flavonoids	244750	0.0574±0.045	556750	0.1779±0.1191	7300000	2.1006±0.1679	739750	0.1221±0.0537	1672500	0.3958±0.302
Kaempferol-3-O-glucoside-7-O-rhamnoside	Flavonoids	6215	0.0015±0.0048	9968	0.0032±0.0102	99900	0.0288±0.0387	8628	0.0014±0.0028	27725	0.0066±0.0096
Quercetin-3-O-robinobioside	Flavonoids	711000	0.1665±0.1332	1605000	0.5125±0.1987	5940000	1.7086±1.0136	3340000	0.5514±0.2356	2072500	0.4907±0.1899
Quercetin-3-O-rutinoside (Rutin)	Flavonoids	638250	0.1494±0.0909	1352500	0.432±0.1013	4890000	1.4065±0.5674	2687500	0.4438±0.138	1512500	0.3578±0.1708
Quercetin-7-O-rutinoside	Flavonoids	616500	0.1443±0.1083	1152500	0.3683±0.3542	3927500	1.1305±0.7021	2340000	0.3868±0.2606	1392500	0.3297±0.2277
Isorhamnetin-3-O-rutinoside (Narcissin)	Flavonoids	89175	0.0209±0.0199	229000	0.0731±0.0751	1037500	0.2987±0.1143	282250	0.0466±0.0321	91575	0.0217±0.0039
6-Hydroxykaempferol-3,6-O-Diglucoside	Flavonoids	93925	0.022±0.018	17300	0.0055±0.0102	97175	0.028±0.0373	845000	0.1396±0.162	68300	0.0162±0.0178
6-Hydroxykaempferol-7,6-O-Diglucoside	Flavonoids	376250	0.0881±0.0316	44200	0.0141±0.0072	115000	0.0331±0.0285	393000	0.0649±0.0315	166000	0.0393±0.0215
Robinin(Kaempferol-3-O-gal-rham-7-O-rham)	Flavonoids	-	-	-	-	187750	0.054±0.0211	-	-	9035	0.0021±0.0063
Quercetin-O-rutinoside-hexose	Flavonoids	9865	0.0023±0.0071	-	-	9030	0.0026±0.004	10658	0.0018±0.0037	22475	0.0053±0.009

6-Hydroxykaempferol-3,6-O-Diglucoside-7-O-Glucuronic Acid	Flavonoids	45575	0.0106±0.0228	16750	0.0054±0.013	13343	0.0039±0.0142	10963	0.0018±0.0036	5435	0.0013±0.0033
Afzelechin(3,5,7,4'-Tetrahydroxyflavan)	Flavonoids	450250	0.1057±0.0933	257500	0.0822±0.0166	659250	0.1897±0.0744	932750	0.1539±0.1125	6215	0.0015±0.0041
Catechin	Flavonoids	2952500	0.6927±0.4837	1390000	0.4441±0.206	5330000	1.5331±0.6092	5697500	0.941±0.2077	25375	0.006±0.0142
Epigallocatechin (EGC)	Flavonoids	32075	0.0075±0.0094	16450	0.0053±0.0084	115500	0.0332±0.0111	218000	0.036±0.0223	-	-
Gallocatechin	Flavonoids	53475	0.0125±0.0155	21925	0.007±0.0137	179750	0.0517±0.0273	373000	0.0616±0.0117	-	-
Catechin-(7,8-bc)-4β-(3,4-dihydroxyphenyl)-dihydro-2-(3H)-pyranone	Flavonoids	2437500	0.5708±0.1396	945750	0.3021±0.042	97250	0.028±0.0087	444250	0.0734±0.0487	31000	0.0073±0.0045
Catechin-(7,8-bc)-4α-(3,4-dihydroxyphenyl)-dihydro-2-(3H)-pyranone	Flavonoids	2365000	0.5539±0.1028	949750	0.3033±0.1303	102225	0.0294±0.01	472750	0.0781±0.0217	29500	0.007±0.0111
Pinoresinol	Lignans	474250	0.1111±0.0298	113250	0.0362±0.0142	62175	0.0179±0.0133	415000	0.0685±0.0437	147500	0.0349±0.015
Syringaresinol	Lignans	111000	0.026±0.0075	21625	0.0069±0.0034	93250	0.0268±0.0047	108250	0.0179±0.0038	28400	0.0067±0.0053
Terpineol monO-glucoside	Lignans	5512500	1.292±1.4298	2410000	0.7691±0.5893	126750	0.0365±0.0328	1612500	0.2661±0.117	852750	0.2019±0.0789
1-Methylhistamine	Others	-	-	-	-	10983	0.0032±0.0084	13550	0.0022±0.0046	151250	0.0358±0.0037
3-Amino-1-propionic sulfonic acid	Others	10905	0.0026±0.004	9953	0.0032±0.0046	10388	0.003±0.0074	45275	0.0075±0.0018	19050	0.0045±0.0074
4-Methyl-5-thiazoleethanol	Others	509000	0.1191±0.039	438250	0.1399±0.0437	1500000	0.4317±0.1057	200000	0.033±0.0133	71350	0.0169±0.0062
D-Glucurono-6,3-lactone	Others	66725	0.0156±0.0179	58225	0.0186±0.0209	22975	0.0066±0.0084	537750	0.0888±0.0175	410750	0.0972±0.0087
N-Acetyl-D-galactosamine	Others	142500	0.0334±0.0271	77475	0.0248±0.0195	55225	0.0159±0.027	191750	0.0316±0.022	157000	0.0372±0.0331
N-Acetyl-D-glucosamine 1-phosphate	Others	84825	0.0199±0.0038	84275	0.0269±0.0067	44650	0.0129±0.0075	195750	0.0324±0.0135	37200	0.0088±0.0046
Gallic acid	Phenolic acids	2805000	0.6542±0.9786	197250	0.0631±0.0699	125500	0.0361±0.0299	4907500	0.8095±0.3994	149250	0.0353±0.0099
Methyl gallate	Phenolic acids	365000	0.0858±0.1006	66950	0.0214±0.0114	55875	0.0161±0.0183	522250	0.0863±0.0761	134000	0.0317±0.0231
Ethyl gallate	Phenolic acids	686000	0.1608±0.0958	26700	0.0085±0.0221	38000	0.0109±0.0166	7472500	1.2334±0.2124	97875	0.0232±0.0198
Galloyl Methyl gallate	Phenolic acids	59125	0.0138±0.0122	56075	0.0179±0.0081	-	-	187500	0.0309±0.0288	71400	0.0169±0.0195
Ursolic acid	Terpenoids	68975	0.0161±0.0323	66950	0.0214±0.0249	80675	0.0232±0.0251	1607500	0.2655±0.177	341750	0.0809±0.0751

2,3-Dihydroxy 5(6),12(13)diene ursolic acid	Terpenoids	138750	0.0325±0.0227	99325	0.0317±0.0169	-	-	-	-	9848	0.0023±0.0017
Maslinic acid	Terpenoids	7292500	1.7074±1.0329	11275000	3.6007±0.7075	4662500	1.3416±0.3256	11725000	1.9369±0.844	11700000	2.7708±0.8915
3,24-Dihydroxy-17,21-semiacetal-12(13)oleanolic acid	Terpenoids	7170000	1.6787±0.8064	11075000	3.5365±0.802	4495000	1.2934±0.3292	11600000	1.9151±0.7004	11625000	2.7538±1.3664
2-Hydroxyoleanolic acid	Terpenoids	7250000	1.6977±0.656	10900000	3.481±0.3164	4565000	1.3133±0.481	11600000	1.9157±0.6903	11600000	2.7478±1.1323
Methoxyursolic acid	Terpenoids	179500	0.0421±0.0307	347750	0.111±0.0409	22200	0.0064±0.0031	995000	0.1643±0.0577	4432500	1.0495±0.289
Euscaphic acid	Terpenoids	10900000	2.5557±0.965	15550000	4.9661±1.283	4010000	1.154±0.1212	7530000	1.2434±0.1069	10875000	2.5754±0.5942
Oleanolic acid 2-O-β-D-glucopyranoside	Terpenoids	24175	0.0057±0.0012	34050	0.0109±0.003	4305	0.0012±0.0014	15775	0.0026±0.0015	52275	0.0124±0.0034
Choline	Alkaloids	304250	0.0713±0.0224	118750	0.0379±0.0216	293750	0.0846±0.0381	378500	0.0625±0.0373	352750	0.0835±0.0278
Betaine	Alkaloids	114500	0.0268±0.0099	47925	0.0153±0.0096	15800	0.0046±0.0051	173000	0.0286±0.019	64650	0.0153±0.0084
Nicotinic Acid Methyl Ester(Methyl Nicotinate)	Alkaloids	341000	0.0798±0.0648	205500	0.0657±0.0589	260250	0.0749±0.0192	187750	0.031±0.0079	29975	0.0071±0.0031
Trigonelline	Alkaloids	2645000	0.6195±0.2039	4447500	1.4208±0.7388	637500	0.1835±0.0554	6532500	1.0788±0.3342	7162500	1.6961±0.6289
6-Hydroxynicotinic acid	Alkaloids	10685	0.0025±0.0056	31550	0.0101±0.012	10170	0.0029±0.0089	77225	0.0127±0.0027	76375	0.0181±0.0127
Acetylcholine	Alkaloids	173000	0.0404±0.0459	130750	0.0417±0.0423	154750	0.0446±0.0196	464250	0.0766±0.0309	299250	0.0709±0.0236
Lumichrome	Alkaloids	4158	0.001±0.0024	13825	0.0044±0.005	45975	0.0132±0.0065	13950	0.0023±0.0021	35550	0.0084±0.0128
Nicotinamide	Vitamins	907000	0.2125±0.0144	680250	0.2172±0.0528	179000	0.0515±0.0168	411750	0.068±0.0098	349250	0.0826±0.0416
Nicotinic acid	Vitamins	1023000	0.2396±0.061	474750	0.1516±0.0619	915750	0.2636±0.078	550500	0.0909±0.0098	471750	0.1116±0.0697
Pyridoxine	Vitamins	112750	0.0264±0.0045	41850	0.0134±0.006	24100	0.0069±0.0079	51750	0.0085±0.006	56475	0.0134±0.0088
4-Pyridoxic acid	Vitamins	16900	0.004±0.0054	12475	0.004±0.0036	9175	0.0026±0.0005	24400	0.004±0.0037	29575	0.007±0.0016
D-Pantothenic Acid	Vitamins	77900	0.0182±0.0116	47725	0.0153±0.0184	501000	0.1441±0.0581	593000	0.0979±0.0344	88950	0.021±0.0687
Riboflavin	Vitamins	250500	0.0587±0.039	318500	0.1018±0.0569	996750	0.287±0.1542	208000	0.0344±0.0215	333000	0.0788±0.0727
Esculetin	Coumarins	185750	0.0435±0.0164	47875	0.0153±0.0244	48800	0.0141±0.0222	143000	0.0236±0.0279	83125	0.0197±0.0278

Scoparone	Coumarins	145750	0.0342±0.0188	72475	0.0232±0.0152	20150	0.0058±0.0099	-	-	23275	0.0055±0.0056
Skimmin	Coumarins	298500	0.0699±0.0255	87925	0.028±0.0379	83975	0.0242±0.024	76900	0.0127±0.0096	404500	0.0959±0.0618
Esculin(6,7-DihydroxyCoumarin-6-glucoside)	Coumarins	49050	0.0116±0.0316	63225	0.0202±0.0335	118000	0.034±0.0204	46825	0.0077±0.0103	192750	0.0456±0.0325
4-hydroxycoumarin di-glucoside	Coumarins	210250	0.0493±0.0239	108500	0.0347±0.0235	-	-	7825	0.0013±0.0023	-	-
Prunetin	Flavonoids	1385	0.0003±0.0003	5110	0.0016±0.0007	1848	0.0005±0.0005	31975	0.0053±0.0015	2050	0.0005±0.0009
Indole-3-carboxaldehyde	Alkaloids	33800	0.0079±0.0093	27275	0.0087±0.0205	72575	0.0209±0.0084	513500	0.0848±0.0245	501250	0.1187±0.0762
Indole-3-carboxylic acid	Alkaloids	32525	0.0076±0.0056	30800	0.0098±0.0091	37000	0.0107±0.0133	457250	0.0755±0.0282	569000	0.1347±0.0346
γ-Aminobutyric acid	Organic acids	11620	0.0027±0.0024	64275	0.0205±0.0137	9300	0.0027±0.0028	15250	0.0025±0.002	11640	0.0028±0.0079
2-Furanoic acid	Organic acids	1130000	0.2649±0.1543	968500	0.3093±0.1227	359250	0.1034±0.054	1011750	0.167±0.0426	1029000	0.2436±0.0609
Methylmalonic acid	Organic acids	2610000	0.6114±0.1825	1642500	0.5247±0.1574	2717500	0.782±0.119	5390000	0.89±0.1095	6855000	1.6228±0.4046
Succinic acid	Organic acids	2485000	0.5823±0.4018	1640000	0.5238±0.1253	2820000	0.8119±0.3	5500000	0.9081±0.068	6875000	1.6277±0.585
L-Homoserine	Organic acids	260500	0.061±0.0302	361750	0.1156±0.0535	98775	0.0285±0.0259	329500	0.0544±0.0203	308000	0.0725±0.3624
Citraconic acid	Organic acids	14450000	3.3837±0.8065	4045000	1.2919±0.1119	438500	0.1261±0.1228	1242500	0.2054±0.1357	3182500	0.7531±0.3335
6-Aminocaproic acid	Organic acids	33600	0.0079±0.0057	94700	0.0303±0.012	31050	0.0089±0.0013	122500	0.0202±0.01	112425	0.0265±0.0939
Oxaloacetic acid	Organic acids	-	-	21475	0.0069±0.0312	37275	0.0107±0.0231	37400	0.0062±0.0053	60150	0.0142±0.0051
2-Methylsuccinic acid	Organic acids	597250	0.1398±0.0517	180000	0.0576±0.0788	269500	0.0776±0.0652	698250	0.1153±0.0324	650000	0.1538±0.1041
(S)-(-)-2-Hydroxyisocaproic acid	Organic acids	299500	0.0702±0.0239	604500	0.193±0.084	72925	0.021±0.0322	515500	0.0851±0.0447	270000	0.0639±0.0712
Malic acid	Organic acids	777750	0.1815±0.2765	483750	0.1544±0.1433	154250	0.0444±0.048	1557500	0.2571±0.0336	943000	0.2231±0.1784
4-Acetamidobutyric acid	Organic acids	230750	0.054±0.0355	142000	0.0454±0.0157	97175	0.028±0.0288	1267500	0.2092±0.0615	203000	0.0481±0.0557
4-Guanidinobutyric acid	Organic acids	1322500	0.3099±0.2531	587000	0.1874±0.0616	1707500	0.4915±0.1931	5125000	0.8459±0.1994	2577500	0.6102±0.018
(Rs)-Mevalonic acid	Organic acids	11825000	2.7695±0.7426	4395000	1.4036±0.1914	420000	0.1207±0.1664	1077500	0.178±0.0607	2005000	0.4748±0.1488

L-(+)-Tartaric acid	Organic acids	293750	0.0686±0.0752	213250	0.0681±0.0308	64075	0.0184±0.0127	2822500	0.4658±0.2276	336750	0.0797±0.0407
2,3-Dihydroxybenzoic Acid	Organic acids	7170000	1.6784±0.3712	11450000	3.656±1.0689	1922500	0.5531±0.2704	9100000	1.502±0.2698	7880000	1.8661±0.9662
Phthalic acid	Organic acids	25800	0.006±0.015	-	-	-	-	48225	0.008±0.0142	26075	0.0062±0.0176
D-Xylonic acid	Organic acids	4032500	0.9415±1.0004	1430000	0.4564±0.3339	8570000	2.4663±0.8674	6755000	1.1152±0.3653	4940000	1.1696±0.3948
Shikimic acid	Organic acids	933000	0.2193±0.2948	112150	0.0359±0.0607	453250	0.1304±0.099	244500	0.0404±0.0298	158000	0.0374±0.0092
SubericAcid	Organic acids	97350	0.0228±0.0215	90150	0.0288±0.0347	99650	0.0287±0.0233	96900	0.016±0.0308	290000	0.0684±0.2382
Anchoic Acid	Organic acids	3977500	0.9319±0.2004	2775000	0.8862±0.129	4665000	1.3427±0.2034	3707500	0.6123±0.1414	10480000	2.4775±3.5832
Quinic Acid	Organic acids	4305000	1.0087±0.4297	2170000	0.693±0.1304	7592500	2.1856±1.2285	541000	0.0894±0.0242	1135000	0.2687±0.1309
D-Galacturonic acid(Gal A)	Organic acids	504250	0.118±0.157	634500	0.2027±0.1508	488250	0.1406±0.0555	13800000	2.2761±1.2092	6037500	1.4296±0.3274
Procyanidin A1	Flavonoids	32175	0.0075±0.0067	50800	0.0162±0.0053	17725	0.0051±0.0164	40150	0.0066±0.0084	-	-
Procyanidin B2	Flavonoids	1290000	0.3021±0.1666	464750	0.1485±0.0622	2212500	0.6365±0.2017	1237500	0.2041±0.1044	-	-
Procyanidin B3	Flavonoids	913250	0.2141±0.0774	329750	0.1053±0.0676	1667500	0.4796±0.2034	916750	0.1513±0.0466	-	-
Procyanidin B1	Flavonoids	1035750	0.2427±0.0684	358500	0.1145±0.0891	1780000	0.5119±0.2305	995000	0.1642±0.0418	-	-
Procyanidin C2	Flavonoids	77950	0.0183±0.0092	34825	0.0111±0.0176	227000	0.0654±0.0332	66475	0.011±0.0216	-	-
Quercetin-O-rutinoside-O-rhamnoside	Flavonoids	6525	0.0015±0.0056	6318	0.002±0.004	192000	0.0552±0.0818	8018	0.0013±0.0029	161250	0.0382±0.0264
Calceorioside B	Others	1192500	0.2793±0.1041	427000	0.1364±0.0923	1385000	0.3987±0.2283	1392500	0.2298±0.1908	100925	0.0239±0.0544
Quercetin-3′,4′-dimethyl ether	Flavonoids	25000	0.0058±0.0118	6400	0.002±0.0037	-	-	33100	0.0055±0.0059	-	-
Isotamarixin	Flavonoids	26825	0.0063±0.0117	14135	0.0045±0.0199	16350	0.0047±0.0078	98125	0.0163±0.0255	186750	0.0442±0.0489
Stachydrine	Alkaloids	889500	0.2085±0.1161	1022250	0.3265±0.0713	404500	0.1164±0.0783	898500	0.1484±0.1309	491750	0.1164±0.0417
Oresbiusin A	Phenolic acids	27350	0.0064±0.0036	36225	0.0116±0.0188	219000	0.063±0.0408	406750	0.0673±0.0564	134500	0.0319±0.0132
5'-Glucopyranosyloxyjasmanic acid	Phenolic acids	40525	0.0095±0.0066	80650	0.0257±0.0219	39475	0.0113±0.0124	68500	0.0113±0.0103	148250	0.0351±0.0246

5,7,2'-Trhiyroxy-8-methoxyflavone	Flavonoids	26825	0.0063±0.0042	16025	0.0051±0.0044	19450	0.0056±0.0054	48000	0.0079±0.0036	8228	0.002±0.0022
6-hydroxy-5,7,4'-trimethoxyflavone	Flavonoids	-	-	-	-	-	-	35375	0.0058±0.0014	-	-
Tetramethylluteolin (3',4',5,7-Tetramethoxyflavone)	Flavonoids	7945	0.0019±0.0003	-	-	-	-	-	-	-	-
wogonoside	Flavonoids	21325	0.005±0.0039	6233	0.002±0.0023	-	-	13175	0.0022±0.0021	-	-
Kaempferol-3-O-β-D-glucuronide	Flavonoids	64600	0.0151±0.0076	24250	0.0078±0.0138	3312500	0.9526±0.5091	1027000	0.1696±0.036	894500	0.2118±0.0724
Isololiolide	Others	62825	0.0147±0.0122	52025	0.0166±0.0088	13150	0.0038±0.0048	99925	0.0165±0.0003	253000	0.0599±0.0272
Stilbostemin B	Others	13800	0.0032±0.0025	9023	0.0029±0.0057	5913	0.0017±0.0031	6073	0.001±0.002	5973	0.0014±0.0019
Prunellin A	Others	42350	0.0099±0.0076	27000	0.0086±0.0143	-	-	-	-	-	-
Quercetin-3-O-(2''-galloyl)-β-D-glucoside	Flavonoids	1015500	0.2376±0.1687	342500	0.1094±0.0332	12500	0.0036±0.0139	2000000	0.3303±0.0785	22800	0.0054±0.0194
Quercetin-3-O-(2'',3''-digalloyl)-β-D-glucopyranoside	Flavonoids	28575	0.0067±0.019	13690	0.0044±0.0102	-	-	30725	0.0051±0.0147	16025	0.0038±0.0054
Methyl Brevifolincarboxylate	Others	1957500	0.4565±0.8954	531750	0.1699±0.292	358500	0.1032±0.1706	3122500	0.5155±0.295	3405000	0.8062±0.7481
Tercatain	Phenolic acids	31850	0.0075±0.0219	-	-	-	-	-	-	-	-
Salicylic acid	Phenolic acids	48200	0.0113±0.0202	64250	0.0205±0.0163	74775	0.0215±0.0299	96300	0.0159±0.0125	141750	0.0335±0.0218
benzoylmalic acid	Phenolic acids	128000	0.03±0.0292	-	-	-	-	-	-	-	-
cinnamoyltartaric acid	Phenolic acids	101375	0.0238±0.0251	59350	0.019±0.0165	-	-	-	-	-	-
feruloylmalic acid	Phenolic acids	-	-	-	-	-	-	-	-	73300	0.0173±0.012
vnilloylcaffeoyltartaric acid	Phenolic acids	-	-	-	-	26300	0.0076±0.0119	66725	0.011±0.0089	-	-
feruloylsinapoyltartaric acid	Phenolic acids	29925	0.007±0.0071	14500	0.0046±0.0076	18850	0.0054±0.0044	160250	0.0264±0.018	20000	0.0047±0.0081
Kaempferol-3-O-(cinnamoyl)-sophoroside-7-O-glucose	Flavonoids	9558	0.0022±0.0014	3783	0.0012±0.0014	-	-	4573	0.0008±0.0009	26125	0.0062±0.0056
3,5,7,4'--Tetrahydroxy-Coumaronochromone	Others	207250	0.0486±0.0769	43125	0.0138±0.0074	53225	0.0153±0.0336	161000	0.0266±0.0913	84125	0.0199±0.0269
Galloyl-HHDP(Hexahydroxydiphenoyl)-glueopy ranose	Phenolic acids	679000	0.1593±0.1643	60575	0.0193±0.0459	-	-	80050	0.0133±0.0596	163000	0.0386±0.0469

4-α-L-Rhamnopyranosyl-ellagic acid	Phenolic acids	410000	0.0961±0.0422	316250	0.101±0.0859	531250	0.153±0.1591	2385000	0.3944±0.3357	6190000	1.4665±0.9306
Angelicin	Coumarins	-	-	-	-	64575	0.0186±0.0225	-	-	-	-
Isoluteolin (Orobol)(5,7,3',4'-tetrahydroxyisoflavone)	Flavonoids	6403	0.0015±0.0018	5348	0.0017±0.0011	13060	0.0038±0.0099	39575	0.0065±0.0188	22100	0.0053±0.0108
2'-Hydoxy,5-methoxy Genistein-O-rhamnosyl-glucoside	Flavonoids	56075	0.0131±0.0057	119150	0.038±0.0509	553500	0.1597±0.3941	151750	0.0251±0.0492	46600	0.011±0.0268
2'-Hydoxy,5-methoxy Genistein-4',7-O-diglucoside	Flavonoids	13025	0.0031±0.0027	54475	0.0174±0.0167	-	-	104725	0.0173±0.0253	44700	0.0106±0.0265
Farrerol 7-O-glucoside	Flavonoids	11183	0.0026±0.0022	84825	0.0271±0.0097	-	-	-	-	-	-
Quercetin 3-O-β-D-xylopyranoside	Flavonoids	1875000	0.4398±0.2929	1712500	0.5469±0.1905	-	-	1822500	0.3005±0.2295	271750	0.0645±0.0937
Azalein (Azaleatin-3-O-rhamnoside)	Flavonoids	48225	0.0112±0.0281	38375	0.0123±0.0216	-	-	-	-	-	-
isohyperoside	Flavonoids	6917500	1.6214±0.3834	1775000	0.5669±0.2313	2062500	0.5937±0.1986	4872500	0.8041±0.3291	2345000	0.5555±0.2415
Epipinoresinol	Lignans	458750	0.1075±0.0689	113750	0.0363±0.0033	60075	0.0173±0.0111	407250	0.0672±0.0539	150000	0.0355±0.0045
Olivil-4'-O-β-D-glucoside	Lignans	156250	0.0366±0.0058	83425	0.0267±0.0092	32475	0.0093±0.0103	341000	0.0563±0.0172	81425	0.0193±0.0126
Eucommin A	Lignans	391250	0.0919±0.1141	119250	0.0381±0.0146	8983	0.0026±0.0112	175250	0.029±0.0225	123500	0.0292±0.018
Eucommia	Others	27550	0.0065±0.0062	33275	0.0106±0.0144	18875	0.0054±0.0105	17125	0.0028±0.0039	97250	0.023±0.0212
Syringaresinol-4'-O-β-D-monO-glucoside	Lignans	43075	0.0101±0.0062	-	-	257250	0.074±0.1145	479000	0.0791±0.0513	42025	0.0099±0.0193
Luteolin-7-O-β-D-gentiobioside	Flavonoids	17675	0.0042±0.0077	7980	0.0025±0.0094	-	-	80625	0.0133±0.0216	6625	0.0016±0.0021
Methyl dioxindole-3-acetate	Others	60400	0.0141±0.0169	20375	0.0065±0.0152	31600	0.0091±0.0105	52950	0.0087±0.0068	-	-
Ergotamine	Alkaloids	8505000	1.9929±0.2377	17975000	5.7392±1.858	9702500	2.7922±0.1557	6555000	1.0822±0.2789	4315000	1.0214±0.4359
3-methoxy-juglone	Quinones	8800	0.0021±0.004	9238	0.003±0.0056	39075	0.0112±0.0162	142250	0.0235±0.0194	3980	0.0009±0.0046
1,4,8-trihydroxy naphthalene-1-O-β-D-glucopyranoside	Quinones	68975	0.0162±0.0037	45275	0.0145±0.0148	50875	0.0147±0.03	62350	0.0103±0.0077	13475	0.0032±0.0046
Isosalicylic acid O-glycoside	Phenolic acids	61900	0.0145±0.0093	63025	0.0201±0.0166	344500	0.0991±0.0502	224000	0.037±0.0163	56350	0.0133±0.0161
p-Coumaric acid-O-glycoside	Phenolic acids	550500	0.1292±0.1377	54075	0.0172±0.0222	87725	0.0252±0.0095	287250	0.0475±0.0258	102650	0.0243±0.0225

Vanillic acid glycoside	Phenolic acids	15300	0.0036±0.0093	13950	0.0045±0.0076	11645	0.0034±0.0117	106250	0.0176±0.0095	19800	0.0047±0.0033
Isosinapic acid-hexoside	Phenolic acids	61450	0.0145±0.0219	97150	0.031±0.041	74225	0.0214±0.011	44050	0.0073±0.0105	-	-
Sinapic acid-glycoside	Phenolic acids	28950	0.0068±0.0064	14475	0.0046±0.006	72900	0.021±0.0145	120250	0.0199±0.0144	41600	0.0099±0.0075
p-Hydroxycinnamic acid	Phenolic acids	180750	0.0423±0.0202	28550	0.0091±0.0228	48150	0.0139±0.0102	97800	0.0162±0.0129	110250	0.0261±0.0097
4-O-glucosyl-4-hydroxybenzoic acid	Phenolic acids	65850	0.0154±0.0154	66300	0.0212±0.0087	330250	0.095±0.0543	214250	0.0354±0.0431	60975	0.0144±0.0226
4-O-glucosyl-3,4-dihydroxybenzyl alcohol	Phenolic acids	69450	0.0163±0.0193	19150	0.0061±0.0042	143500	0.0413±0.0161	91275	0.0151±0.0115	-	-
6-O-caffeoyl-β-glucose	Phenolic acids	826250	0.1938±0.1935	673250	0.2151±0.0888	453000	0.1304±0.1008	966000	0.1596±0.0724	321250	0.0761±0.0371
6-O-feruloyl-α-glucose	Phenolic acids	20000	0.0047±0.0182	27125	0.0087±0.0103	287250	0.0827±0.062	124750	0.0206±0.0092	15875	0.0038±0.0075
Kaempferol-3-O-(6''-acetyl)-glucoside	Flavonoids	33475	0.0078±0.0039	12110	0.0039±0.0053	236000	0.0679±0.0377	55625	0.0092±0.0044	35825	0.0085±0.015
Lariciresinol glucopyranoside	Others	189500	0.0444±0.0098	84900	0.0271±0.0243	-	-	60650	0.0101±0.0169	29175	0.0069±0.0105
Betulinic acid	Terpenoids	1196	0.0003±0.0016	4220	0.0014±0.0029	870	0.0003±0.0008	7738	0.0013±0.0036	10005	0.0024±0.0055
Ovalifoliolides B	Terpenoids	17575	0.0041±0.0037	16750	0.0054±0.003	-	-	4450	0.0007±0.0021	-	-
Eriodictyol-O-glucoside	Flavonoids	337500	0.079±0.0259	1360000	0.4342±0.144	121500	0.035±0.0153	1577500	0.2605±0.0356	43850	0.0104±0.0111
Quercetin-3-O-(6''-O-acetyl)-galactoside	Flavonoids	87125	0.0205±0.0216	16875	0.0054±0.0078	228250	0.0657±0.016	21325	0.0035±0.0024	23150	0.0055±0.0072
Nortrachelogenin 4-O-β-D-glucoside	Lignans	159000	0.0373±0.0282	70725	0.0226±0.0247	34575	0.0099±0.0161	47050	0.0078±0.0069	29075	0.0069±0.0208
Kaempferol 3-O-β-d-(6''-O-(E)-p-coumaroyl) glucopyranoside	Flavonoids	2350000	0.5508±0.2219	2400000	0.7658±0.7921	3787500	1.0904±0.5668	11975000	1.9773±0.7828	6090000	1.4412±0.6307
Sexangularetin 3-glucoside-7-rhamnoside	Flavonoids	53850	0.0126±0.019	123825	0.0395±0.0517	594750	0.171±0.4793	148250	0.0245±0.0413	39075	0.0093±0.0183
Dimethylmalonic acid	Organic acids	49525	0.0116±0.0062	17050	0.0054±0.0128	26300	0.0076±0.0131	58900	0.0097±0.0131	54400	0.0129±0.0185
Limocitrin 7-glucoside	Flavonoids	-	-	110350	0.0352±0.0391	-	-	-	-	-	-
Isorhamnetin-7-O-glucoside	Flavonoids	1145500	0.2675±0.3499	407750	0.1303±0.0675	1332500	0.3833±0.4129	1345000	0.2219±0.1472	109575	0.0259±0.0205
Dihydroisopelletierine	Alkaloids	602000	0.141±0.0633	598250	0.1911±0.0971	193250	0.0556±0.0343	549500	0.0907±0.0481	156500	0.0371±0.0077

Dihydrosedinine	Alkaloids	257500	0.0604±0.0262	254250	0.0812±0.059	583250	0.1678±0.0437	220750	0.0365±0.0284	211750	0.0501±0.0483
Parthenolide	Terpenoids	126750	0.0297±0.0205	285500	0.0912±0.0374	49200	0.0142±0.0082	71375	0.0118±0.0077	7830	0.0019±0.0019
Santamarin	Terpenoids	-	-	-	-	539000	0.1553±0.1754	31250	0.0052±0.0085	26450	0.0063±0.0082
Ixerin D	Terpenoids	200000	0.0469±0.0173	60750	0.0194±0.0185	32250	0.0093±0.005	81725	0.0135±0.0194	88300	0.0209±0.0179
Silibinin	Flavonoids	-	-	-	-	791500	0.2277±0.0584	55700	0.0092±0.0028	3220	0.0008±0.0004
Hispidulin	Flavonoids	13375	0.0031±0.0027	7553	0.0024±0.0009	7200	0.0021±0.0022	28225	0.0047±0.0016	3398	0.0008±0.0019
Eupatilin	Flavonoids	5053	0.0012±0.0018	12400	0.004±0.0017	-	-	20050	0.0033±0.0015	-	-
Penduletin (5,4'-Dihydroxy-3,6,7-trimethoxyflavone)	Flavonoids	3438	0.0008±0.0009	9840	0.0031±0.0004	-	-	13400	0.0022±0.0025	-	-
Acacetin-7-O-glucuronide	Flavonoids	26750	0.0063±0.0087	-	-	-	-	12075	0.002±0.0029	-	-
Scutellarin(Scutellarein-7-O-glucuronide)	Flavonoids	74000	0.0173±0.0171	25125	0.008±0.0063	3185000	0.916±0.4592	939000	0.155±0.084	836750	0.1981±0.0881
Diosmetin-7-O-galactoside	Flavonoids	141500	0.0332±0.0343	91625	0.0293±0.02	63625	0.0183±0.0147	167750	0.0277±0.0172	25625	0.0061±0.0181
Pratensein 7-O-glucopyranoside	Flavonoids	9428	0.0022±0.0076	70500	0.0225±0.0143	-	-	4443	0.0007±0.0011	-	-
Diosmetin-7-O-glucuronide	Flavonoids	41725	0.0098±0.0178	-	-	-	-	-	-	-	-
6-methoxykaempferol-3-O-glucoside	Flavonoids	1145000	0.2684±0.1587	404500	0.1291±0.0504	1417500	0.4077±0.1551	1427500	0.2359±0.1796	112150	0.0265±0.0291
Patuletin-3-O-β-D-glucopyranoside	Flavonoids	80025	0.0188±0.0252	-	-	-	-	-	-	-	-
Luteolin-7-O-rutinoside	Flavonoids	61050	0.0143±0.0256	102175	0.0327±0.073	1151500	0.3312±0.5773	112500	0.0186±0.0236	306000	0.0725±0.022
Quercetin-7-O-(6'-O-malonyl)-β-D-glucoside	Flavonoids	45875	0.0107±0.0181	26225	0.0084±0.0133	47750	0.0137±0.0271	109825	0.0181±0.0155	10620	0.0025±0.0045
3-O-(2-O-Acetyl-β-D-glucopyranosyl) oleanolic acid	Terpenoids	595500	0.1396±0.0991	948250	0.3029±0.1809	250000	0.072±0.0472	2460000	0.4061±0.1372	2002500	0.4742±0.2214
5,2'-Dihydroxy-7,8-dimethoxyflavone glycosides	Flavonoids	-	-	2980	0.001±0.0015	-	-	40950	0.0068±0.0046	-	-
3,4-Di-O-galloyl-shikimic acid	Phenolic acids	173250	0.0406±0.0189	119550	0.0382±0.0659	132500	0.0381±0.0268	1013250	0.1674±0.1002	348250	0.0825±0.0453
3-O-Digalloyl quinic acid	Phenolic acids	109000	0.0254±0.0431	18000	0.0058±0.0159	3880000	1.1163±0.2646	168250	0.0277±0.03	37225	0.0088±0.0062

Di-O-Glucose-quinic acid	Phenolic acids	2912500	0.6845±0.732	832000	0.2657±0.0759	756500	0.2179±0.1237	300250	0.0497±0.0554	163000	0.0386±0.0193
5-O-Galloylhamamelose	Phenolic acids	6890000	1.6138±0.3208	1595000	0.5095±0.1804	1170000	0.3367±0.0987	6340000	1.0467±0.2702	689750	0.1634±0.0551
Gemin D	Phenolic acids	626000	0.1467±0.1159	58875	0.0188±0.0507	-	-	74575	0.0123±0.0249	139250	0.0329±0.0389
Nobotanin D	Phenolic acids	32375	0.0076±0.0141	-	-	-	-	-	-	-	-
Cuspinin	Phenolic acids	99675	0.0233±0.0333	12700	0.0041±0.0075	-	-	17450	0.0029±0.0092	-	-
mlyricetin 3-O-B-D-glucopyranoside	Flavonoids	864250	0.2028±0.1976	223250	0.0712±0.081	247000	0.0711±0.0411	598750	0.0987±0.0857	338750	0.0802±0.146
Mearnsitrin	Flavonoids	252250	0.059±0.0583	115500	0.0369±0.032	529500	0.1524±0.0508	465500	0.0769±0.0482	34225	0.0081±0.0103
2,4,6-trihydroxy benzoic acid	Organic acids	98725	0.0233±0.0614	117250	0.0374±0.0247	26725	0.0077±0.0175	99675	0.0164±0.02	35175	0.0083±0.0138
Clemaphenol A	Lignans	189750	0.0443±0.0426	6865	0.0022±0.0072	12950	0.0037±0.0023	76150	0.0126±0.0128	-	-
Ailantinol E	Others	27050	0.0063±0.0054	10030	0.0032±0.0034	365500	0.1051±0.0562	128250	0.0212±0.0033	39650	0.0094±0.0064
Ailanindole	Alkaloids	50850	0.0119±0.0096	20575	0.0066±0.0165	35575	0.0102±0.021	218250	0.0361±0.1168	29550	0.0069±0.0533
6-Hydroxy-7-methoxycoumarin	Coumarins	16900	0.0039±0.0117	82275	0.0263±0.0163	1102500	0.3173±0.0962	20150	0.0033±0.0022	991500	0.2347±0.0785
Ayapin	Coumarins	230250	0.054±0.0147	168250	0.0537±0.0151	275000	0.0791±0.0172	228750	0.0377±0.0248	643750	0.1521±0.2513
Tubuloside C	Phenolic acids	63350	0.0149±0.0222	38650	0.0123±0.0296	11050000	3.1802±0.598	219250	0.0362±0.0268	287500	0.0681±0.0554
Swertiamarin	Terpenoids	52350	0.0123±0.0121	15950	0.0051±0.0141	54050	0.0156±0.0211	51950	0.0086±0.0102	-	-
3-O-Galloyl-β-D-glucose	Phenolic acids	7002500	1.6421±0.8339	1620000	0.5174±0.1033	1160000	0.3339±0.1498	6420000	1.0596±0.2322	680750	0.1612±0.0565
2-O-Galloyl-β-D-glucose	Phenolic acids	1547500	0.3624±0.2049	2542500	0.8121±0.174	4562500	1.3133±0.2385	5400000	0.8913±0.3015	3882500	0.9191±0.1886
Geraniinic acid C	Phenolic acids	22400	0.0052±0.0169	-	-	-	-	23275	0.0038±0.0052	58225	0.0138±0.0187
Glucosyloxybenzoic acid	Phenolic acids	66875	0.0157±0.0297	60200	0.0192±0.017	280250	0.0807±0.0322	214250	0.0354±0.0197	60575	0.0143±0.0093
5-(2-Hydroxyethyl)-2-O-glucosylohenol	Phenolic acids	674750	0.1583±0.1346	827500	0.2645±0.228	589000	0.1695±0.0456	437000	0.0722±0.0698	41700	0.0099±0.0235
Cimidahurinine	Phenolic acids	944000	0.2213±0.2002	1187500	0.3793±0.1199	828000	0.2384±0.105	639000	0.1052±0.143	46950	0.0111±0.0097

Dihydrokaempferol-7-O-glucoside	Flavonoids	329250	0.0771±0.0512	1427500	0.4556±0.29	116000	0.0334±0.0216	1507500	0.2489±0.0325	44750	0.0106±0.0098
Hederagenin	Others	7357500	1.7237±0.7654	10975000	3.5036±1.2325	4592500	1.3217±0.2309	11925000	1.9697±0.6376	11775000	2.7889±0.8828
Biondnoid I	Flavonoids	647250	0.1516±0.0449	709250	0.2263±0.1743	1177500	0.3389±0.1435	4007500	0.6618±0.3477	1970000	0.4667±0.3402
Sinapaldehyde Glucoside	Phenolic acids	-	-	249500	0.0797±0.0337	-	-	-	-	19725	0.0047±0.0091
Plantainoside A	Phenolic acids	139500	0.0326±0.0287	53975	0.0172±0.0211	29225	0.0084±0.0101	93925	0.0155±0.0122	-	-
Quercetin-3-O-α-L-rhamnopyranoside	Flavonoids	2807500	0.6574±0.3078	907750	0.2902±0.3404	1777500	0.5114±0.3584	3817500	0.6302±0.2868	2540000	0.6012±0.1278
Quercetin-3-O-(2-O-α-L-rhamnopyranosyl)-β-D-galactopyranoside	Flavonoids	10833	0.0025±0.0049	11548	0.0037±0.0047	248500	0.0715±0.0904	9508	0.0016±0.002	153250	0.0363±0.0473
6-O-Galloyl-β-D-glucose	Phenolic acids	6970000	1.6319±0.3206	1607500	0.5135±0.217	1127500	0.3246±0.1428	6510000	1.0749±0.2902	667000	0.158±0.1037
Maplexin D (2,4-Di-O-Galloyl-1,5-Anhydro-D-Glucitol)	Phenolic acids	489250	0.1145±0.0655	63925	0.0204±0.0377	-	-	79700	0.0131±0.0482	-	-
Maplexin C (2,3-Di-O-Galloyl-1,5-Anhydro-D-Glucitol)	Phenolic acids	633500	0.1479±0.185	112975	0.0361±0.0538	-	-	119750	0.0198±0.0475	-	-
2,3-Di-O-Galloyl-D-Glucose	Phenolic acids	144250	0.0336±0.055	51050	0.0163±0.0195	20525	0.0059±0.0144	877000	0.1448±0.1171	142500	0.0337±0.0411
Methyl 4,6-di-O-galloyl-D-glucoside	Phenolic acids	402250	0.0943±0.0475	67075	0.0214±0.0334	22025	0.0063±0.0189	21150	0.0035±0.0046	9753	0.0023±0.0093
Maplexin H	Phenolic acids	340500	0.0797±0.0953	174750	0.0558±0.0697	24575	0.0071±0.0137	545250	0.0901±0.0666	65050	0.0154±0.0276
Maplexin G	Phenolic acids	70350	0.0164±0.0206	26100	0.0083±0.0116	23375	0.0067±0.0142	169500	0.028±0.0283	51550	0.0122±0.0128
Geniposide	Terpenoids	48475	0.0114±0.0119	22375	0.0071±0.0111	27775	0.008±0.0116	1412500	0.2334±0.3439	110000	0.0261±0.0717
7'-O-Sinapoyljasminoside L	Terpenoids	343500	0.0805±0.0946	36350	0.0116±0.0125	-	-	431500	0.0712±0.1239	8953	0.0021±0.0027
Quercetin-3-sambubioside	Flavonoids	-	-	7178	0.0023±0.0043	58050	0.0167±0.0252	78050	0.0129±0.0154	1152500	0.2729±0.1644
Betulin	Terpenoids	14750	0.0035±0.0035	11280	0.0036±0.0051	6995	0.002±0.0061	67550	0.0112±0.0077	100725	0.0238±0.0153
Uncargenin D	Terpenoids	57575	0.0135±0.0163	113250	0.0362±0.0181	-	-	199000	0.0329±0.0294	35700	0.0085±0.0068
Obtusilin	Terpenoids	50800	0.0119±0.0027	97675	0.0312±0.0352	5290	0.0015±0.0037	93025	0.0154±0.0051	190750	0.0452±0.017
Dihydromyricetin-O-glucoside	Flavonoids	32600	0.0077±0.0148	20525	0.0066±0.0075	10118	0.0029±0.0084	38775	0.0064±0.0026	25025	0.0059±0.0127

Syringic Aldehyde-glucoside	Phenolic acids	47975	0.0113±0.0192	54825	0.0175±0.0383	246500	0.0708±0.1023	-	-	-	-
1'-O-vanilloyl-β-D-glucoside	Phenolic acids	24000	0.0056±0.0146	12148	0.0039±0.0167	13275	0.0038±0.0087	517500	0.0854±0.0269	31625	0.0075±0.0096
3-Prenyl-4-O-β-D-glucopyranosyloxy-4-hydroxyl-benzoic acid	Phenolic acids	19750	0.0046±0.0084	-	-	224750	0.0647±0.0659	-	-	31675	0.0075±0.0184
Sieboldin	Flavonoids	12475	0.0029±0.0069	13498	0.0043±0.0095	155250	0.0446±0.0523	126500	0.0209±0.0117	-	-
Kaempferol-malonyl-3-O-glucoside	Flavonoids	37600	0.0088±0.0166	19550	0.0062±0.0066	46300	0.0133±0.0131	51000	0.0084±0.0041	12210	0.0029±0.0041
Isorhamnetin O-malonylglucoside	Flavonoids	3805	0.0009±0.0019	2863	0.0009±0.001	10730	0.0031±0.0066	7090	0.0012±0.001	-	-
Kaempferol 3-O-(6''-trans-p-Coumaroyl)-β-D-glucopyranoside	Flavonoids	29425	0.0069±0.0135	17650	0.0056±0.0027	87225	0.0251±0.0241	221500	0.0365±0.0414	36725	0.0087±0.0192
Kampferol 3-O-(6′′-galloyl)-β-D-galactopyranoside	Flavonoids	164250	0.0384±0.0573	58225	0.0186±0.021	9803	0.0028±0.0072	265500	0.0437±0.053	-	-
Kampferol 3-O-(2′′-galloyl)-β-D-galactopyranoside	Flavonoids	184250	0.0432±0.0027	66700	0.0213±0.0304	-	-	252500	0.0416±0.0234	-	-
Quercetin 3-O-(6′′-galloyl)-β-D-galactopyranoside	Flavonoids	1046750	0.2451±0.1907	361500	0.1155±0.0829	13308	0.0038±0.0095	2032500	0.3356±0.1082	23375	0.0055±0.0117
Quercetin-O-pentosyl-O-rhamnoside-O-glucoside	Flavonoids	-	-	7253	0.0023±0.0014	13365	0.0038±0.0153	-	-	93425	0.0222±0.0383
Cinchonain Ic	Flavonoids	344500	0.0806±0.0442	111750	0.0357±0.0127	9093	0.0026±0.0032	64500	0.0107±0.0073	3258	0.0008±0.0015
dihydrodehydrodiconiferyl alcohol 4-O-β-D-glucopyranosides	Lignans	506000	0.1186±0.0464	224000	0.0716±0.04	540250	0.1555±0.086	1795000	0.2962±0.1168	545750	0.1292±0.0553
(-)-secoisolariciresinol 4-O-β-D-giucopyranoside	Lignans	92900	0.0218±0.0201	44600	0.0142±0.035	2630000	0.7569±0.2002	331000	0.0546±0.0258	101425	0.024±0.0153
Roseoside	Others	484750	0.1136±0.0568	361250	0.1154±0.0397	87875	0.0253±0.0231	152500	0.0252±0.0258	74975	0.0177±0.0117
p-Coumaroyleuscaphic acid	Terpenoids	103525	0.0242±0.0327	69675	0.0223±0.0287	251000	0.0721±0.1789	105400	0.0174±0.0135	88775	0.0211±0.0277
Caffeoyl hawthorn acid	Terpenoids	652750	0.1534±0.2224	432500	0.1383±0.1954	97625	0.0281±0.0286	1485000	0.2453±0.3308	494500	0.1171±0.0909
3-O-Trans-feruloyl euscaphic acid	Terpenoids	87050	0.0204±0.007	33275	0.0106±0.0238	12750	0.0037±0.0037	43325	0.0072±0.0068	17300	0.0041±0.0066
3β-Hydroxy-28-norurs-17,19,21-trien	Terpenoids	106500	0.0249±0.0089	262250	0.0837±0.0742	-	-	-	-	-	-
Sanguisorbigenin	Terpenoids	816750	0.1912±0.1316	1157500	0.3695±0.2369	233500	0.0672±0.0283	2315000	0.3823±0.0388	1337500	0.3167±0.1319
Lup-12-en-15α,19β-diol-3,11-dioxo-28-oic acid	Terpenoids	31125	0.0073±0.0031	97575	0.0312±0.0104	-	-	8480	0.0014±0.0016	114750	0.0272±0.0095

Solatuberenol A	Others	792000	0.1859±0.1562	496250	0.1582±0.2936	87325	0.0251±0.003	201750	0.0333±0.0227	107250	0.0254±0.0475
Cis-p-coumaric acid 4-O-glucoside	Others	511250	0.12±0.1038	48775	0.0156±0.0141	88575	0.0255±0.026	286750	0.0473±0.0192	92275	0.0219±0.032
6-Deoxyfagomine	Alkaloids	10460	0.0025±0.004	26075	0.0083±0.0076	11018	0.0032±0.0042	30800	0.0051±0.0021	26750	0.0063±0.0207
3-Indoleacrylic acid	Alkaloids	3473	0.0008±0.0017	4493	0.0014±0.0052	19175	0.0055±0.0045	83575	0.0138±0.0037	7253	0.0017±0.0166
L-Ascorbic acid	Vitamins	402750	0.0944±0.0515	625750	0.1997±0.1218	124250	0.0358±0.0214	726500	0.12±0.1355	293500	0.0695±0.0625
Isorhamnetin 3-O-β-D-Glucoside	Flavonoids	1161000	0.2721±0.2774	432500	0.1383±0.1785	1332500	0.3835±0.3344	1335000	0.2201±0.1542	107875	0.0256±0.0476
Rhamnetin 3-O-β-D-Glucoside	Flavonoids	1172500	0.2747±0.0727	409750	0.1309±0.0318	1432500	0.4125±0.2283	1387500	0.2289±0.1785	106350	0.0252±0.0111
Kaempferol 3-O-β-D-neohesperidoside	Flavonoids	166500	0.0391±0.0765	301500	0.0963±0.1533	3740000	1.0773±1.0323	394250	0.0653±0.129	792000	0.1876±0.1149
Quercetin 3-O-β-D-xylopyranosyl(1→2)-β-D-galactopyranoside	Flavonoids	-	-	9708	0.0031±0.0097	51825	0.0149±0.011	-	-	1133750	0.2683±0.3025
Quercetin 3-O-β-D-neohesperidoside	Flavonoids	608750	0.1422±0.1855	1180000	0.377±0.1728	4190000	1.2055±0.8166	2292500	0.3789±0.2258	1387500	0.3289±0.4255
Benzyl-O-β-D-glucopyranose-β-D-xyranoside	Others	111550	0.0262±0.0324	78475	0.0251±0.021	152500	0.0438±0.0425	234250	0.0386±0.042	11078	0.0026±0.0183
Isothankunic acid	Terpenoids	236250	0.0553±0.036	777000	0.248±0.1529	39900	0.0115±0.0126	64950	0.0107±0.0078	7700	0.0018±0.0027
Terminolic acid	Terpenoids	57700	0.0135±0.0053	153750	0.0491±0.021	16625	0.0048±0.0058	12925	0.0021±0.0017	91375	0.0216±0.0049
Corosolic acid	Terpenoids	-	-	-	-	1880000	0.541±0.083	361500	0.0597±0.0429	-	-
Osthole	Coumarins	7198	0.0017±0.0015	20825	0.0067±0.0044	-	-	5855	0.001±0.001	11625	0.0028±0.0063
Luteolin-O-rutinoside-O-rhamnoside	Flavonoids	-	-	-	-	190750	0.0549±0.0176	-	-	7888	0.0019±0.006
Geraniin	Phenolic acids	40475	0.0095±0.0345	-	-	-	-	20800	0.0034±0.0056	50275	0.0119±0.0155
Methyl neochebulagate	Others	48950	0.0114±0.0211	-	-	-	-	-	-	-	-
1,4-di-O-galloyl-β-D-glcose	Phenolic acids	-	-	54150	0.0173±0.0177	527750	0.1518±0.0437	45900	0.0076±0.0159	34300	0.0081±0.0114
pomolic acid	Terpenoids	7362500	1.7257±1.2565	11175000	3.5684±0.8233	4722500	1.359±0.3049	11575000	1.911±0.2642	11700000	2.7713±1.0873
alphitolic acid	Terpenoids	7315000	1.7133±0.7495	10975000	3.5049±0.9639	4575000	1.3161±0.4683	11700000	1.9324±0.5606	11750000	2.7823±0.5112

2α-hydroxyursolic acid	Terpenoids	332000	0.0778±0.0476	573000	0.183±0.0126	51925	0.0149±0.0921	7710000	1.2732±0.3261	589750	0.1396±0.0376
isoceanothic acid	Terpenoids	2845000	0.6667±0.1161	6472500	2.0672±0.4412	134750	0.0388±0.014	1145000	0.1891±0.0322	2412500	0.5712±0.0981
2α-hydroxypyracrenic acid	Terpenoids	318000	0.0747±0.0659	188750	0.0602±0.0469	72025	0.0207±0.0081	734500	0.1214±0.0826	248000	0.0586±0.069
5,7,3',4',5'-pentahydroxydihydroflavone	Flavonoids	58025	0.0136±0.0084	13150	0.0042±0.0083	-	-	13850	0.0023±0.0056	-	-
Myricetin-O-rhamnoside	Flavonoids	288000	0.0674±0.023	169000	0.0542±0.2838	170000	0.0489±0.0427	969000	0.1599±0.1023	6530000	1.5452±0.7095
Annuionone D	Others	101925	0.0239±0.011	216000	0.069±0.0835	210250	0.0605±0.0364	755750	0.1246±0.1709	636500	0.1507±0.0824
Taxifolin-3'-O-β-D-glucoside	Flavonoids	325250	0.0761±0.058	133250	0.0426±0.039	147750	0.0425±0.0339	329000	0.0544±0.0292	160750	0.0381±0.0486
7S,8R-threo-3',9,9'-trihydroxy-3-methoxy-4',7-epoxy-neolignan-4-O-α-L-rhamnopyranoside	Lignans	9767500	2.2825±2.4914	1765000	0.5639±0.2742	-	-	-	-	-	-
secoisolariciresinol 9-O-β-D-glucopyranoside	Lignans	21950	0.0051±0.0037	5478	0.0018±0.0061	489500	0.141±0.1137	308000	0.0509±0.0446	-	-
Ligraminol E	Others	24250	0.0057±0.0039	7250	0.0023±0.0017	4320	0.0012±0.0033	19225	0.0032±0.0005	3508	0.0008±0.0022
4,7,9,9'-Tetrahydroxy-3,3'-dimethoxy-8-O-4'-neolignan	Others	296750	0.0695±0.0265	55350	0.0177±0.0216	53925	0.0155±0.0075	451750	0.0746±0.0282	15000	0.0036±0.0072
N-Benzylmethylene isomethylamine	Alkaloids	8440	0.002±0.0021	22525	0.0072±0.0032	9265	0.0027±0.0021	74425	0.0123±0.0023	50575	0.0119±0.0409
2-Hydroxy-5,8,11,14,17-icosapentaenoyloxy]propyl-2-(trimethylammonio)ethyl phosphate	Alkaloids	1532500	0.3589±0.1584	2290000	0.731±0.3274	295500	0.0851±0.026	995750	0.1644±0.0434	1822500	0.4316±0.083
monogalloyl-diglucose	Phenolic acids	253750	0.0594±0.0389	-	-	-	-	19725	0.0033±0.0047	-	-
Epicatechin glucoside	Flavonoids	1572500	0.3692±0.3158	685500	0.219±0.0854	823000	0.2368±0.0641	446250	0.0736±0.0678	16425	0.0039±0.0071
(2R)-Pinocembrin-7-neohesperidoside	Flavonoids	74525	0.0175±0.0056	119000	0.038±0.0116	25275	0.0073±0.0023	4560	0.0008±0.0024	-	-
Caffeoylferuloylshikimic?acid	Phenolic acids	9225	0.0022±0.0026	3068	0.001±0.0037	2373	0.0007±0.0008	4408	0.0007±0.0014	114500	0.0271±0.0123
Scopoletin?Beta-D-Glucuronide	Coumarins	25100	0.0059±0.0041	83575	0.0267±0.0123	-	-	11755	0.0019±0.0043	26800	0.0064±0.0107
Dihydrokaempferol-3-O-β-D-glucoside	Flavonoids	189750	0.0443±0.0765	56075	0.0179±0.0241	146500	0.0422±0.0327	242750	0.0401±0.0431	227000	0.0537±0.0779
Salireposide	Phenolic acids	2810000	0.6586±0.3409	1937500	0.6184±0.4354	53925	0.0155±0.0148	-	-	-	-
Chrysin-7-glucoside	Flavonoids	59500	0.0139±0.0054	80050	0.0256±0.0096	45225	0.013±0.0076	170500	0.0282±0.0085	23625	0.0056±0.0041

Galangin-7-glucoside	Flavonoids	41075	0.0096±0.0134	83050	0.0266±0.0607	379750	0.1094±0.1043	88925	0.0147±0.0179	30400	0.0072±0.0045
Kaempferol-7-O-β-D-glucopyranoside	Flavonoids	7420000	1.738±0.7047	3037500	0.9699±0.315	5520000	1.5898±2.4703	11950000	1.9719±0.6003	8612500	2.0376±1.2452
Kaempferol-4'-O-β-D-glucopyranoside	Flavonoids	7382500	1.7298±0.1624	2647500	0.8457±1.1259	5960000	1.7157±0.8786	11725000	1.9355±0.3103	8562500	2.0267±0.5315
(Kaempferol-3-O-β-D-galactoside-4'O-β-D-glucoside)	Flavonoids	14200	0.0034±0.0085	20975	0.0067±0.0031	23550	0.0068±0.0091	76625	0.0126±0.0136	30775	0.0073±0.015
Tricetin?4'-methyl?ether-3'-O-β-D-glucoside	Flavonoids	1235000	0.2892±0.0835	411000	0.1313±0.1037	1420000	0.4087±0.172	1380000	0.2278±0.1863	105600	0.025±0.0395
Luteolin-caffeoyl-O-rhamnoside	Flavonoids	2885000	0.6754±0.2196	3147500	1.0052±0.2823	6105000	1.7563±0.5438	18375000	3.0344±0.489	9907500	2.3454±0.4833
Luteolin-O-glucuronate-O-rhamnoside	Flavonoids	-	-	-	-	134000	0.0386±0.0478	-	-	-	-
Camaldulenic acid	Terpenoids	4095000	0.959±0.4114	8117500	2.5915±0.7059	548000	0.1576±0.1341	19875000	3.282±0.3974	9115000	2.1582±0.3947
Isoscopoletin (6-Hydroxy-7-Methoxycoumarin)	Coumarins	85925	0.0202±0.0092	19250	0.0062±0.0108	115750	0.0333±0.0386	262500	0.0434±0.027	43475	0.0103±0.0123
Dunalianoside C	Phenolic acids	385250	0.0903±0.0822	565000	0.1803±0.0821	366500	0.1054±0.0467	2952500	0.4874±0.031	96750	0.0229±0.0276
Dunalianoside B	Phenolic acids	335500	0.0786±0.0284	1357500	0.4334±0.1325	108375	0.0312±0.0222	1492500	0.2465±0.1391	44875	0.0106±0.0151
Poncirin(Isosakuranetin-7-neohesperidoside)	Flavonoids	2930000	0.6867±0.1807	3292500	1.0512±0.2344	6330000	1.8218±0.5443	18575000	3.0669±0.2776	10152500	2.4034±0.1908
Hesperetin 7-O-neohesperidoside(Neohesperidin)	Flavonoids	339500	0.0796±0.0337	97175	0.0311±0.0252	586000	0.1686±0.0839	1067500	0.1762±0.0241	771500	0.1827±0.0628
Dihydrokaempferol	Flavonoids	39675	0.0093±0.0039	17425	0.0056±0.0024	551500	0.1587±0.0694	206250	0.0341±0.0094	18775	0.0044±0.0057
5,7,8,4'-Tetramethoxyflavone	Flavonoids	15050	0.0035±0.0013	1725	0.0006±0.0015	2045	0.0006±0.0005	2658	0.0004±0.0003	1550	0.0004±0.001
Luteolin 7-O-neohesperidoside(Lonicerin)	Flavonoids	7218	0.0017±0.0038	10168	0.0033±0.0076	111625	0.0321±0.0497	11375	0.0019±0.0016	22375	0.0053±0.007
Neodiosmin (Diosmetin-7-O-Neohesperidoside)	Flavonoids	515000	0.1205±0.0639	232250	0.0742±0.0522	7515	0.0022±0.007	3670	0.0006±0.0029	6598	0.0016±0.0015
Limonin	Others	135250	0.0317±0.0102	151750	0.0485±0.0258	60425	0.0174±0.0158	22975	0.0038±0.0026	190500	0.0451±0.0135
Koaburaside	Phenolic acids	221000	0.0518±0.0309	140750	0.045±0.0289	421750	0.1214±0.0321	504250	0.0833±0.0202	209250	0.0496±0.0216
(+)-Isolariciresinol	Lignans	103250	0.0242±0.0154	18375	0.0059±0.0052	52625	0.0151±0.0096	202750	0.0335±0.0284	8860	0.0021±0.0013
Lyoniresinol	Lignans	175750	0.0413±0.0627	52575	0.0168±0.0224	227000	0.0653±0.0668	232500	0.0384±0.0576	14995	0.0036±0.0119

Isolariciresinol 9'-O-Glucoside	Lignans	504000	0.1181±0.0321	205750	0.0657±0.0349	534750	0.1539±0.028	1760000	0.2907±0.1032	534250	0.1263±0.1059
5'-methoxyisolariciresinol-9'-O-β-D-xylopyranoside	Lignans	73875	0.0173±0.0141	16025	0.0051±0.0097	-	-	-	-	-	-
lyoniresinol-9'-O-β-D-xylopyranoside	Lignans	863500	0.2024±0.1573	259500	0.0829±0.0166	-	-	86600	0.0143±0.0036	-	-
5'-methoxyisolariciresinol-9'-O-β-D-glucopyranoside	Lignans	65550	0.0154±0.0139	31950	0.0102±0.0164	142750	0.041±0.032	231000	0.0382±0.0097	166500	0.0394±0.0439
Machilusolide D	Others	361000	0.0846±0.0494	186500	0.0596±0.0365	203500.0000	0.0585±0.0398	492000	0.0832±0.0589	650667	0.1538±0.0365
Byzantionoside A	Others	1455000	0.3417±0.2952	440000	0.1407±0.1986	79150.0000	0.0228±0.0427	277667	0.0455±0.0228	38233	0.0093±0.0128

Table S5. Potential markers and corresponding activities of five *Rosa* fruits.

					Containing or not				
NO.	Tentative identification	Class	Sub class	Pharmacological activity	RRT-F	RSS-F	RLM-F	RDP-F	RSL-F
1	Biochanin A	Flavonoids	Isoflavones	Fatty acid amide hydrolase (FAAH) inhibitor [1].				√	
2	Delphinidin 3-O-glucoside (Mirtillin)	Flavonoids	Anthocyanins	-	√				
3	Epicatechin gallate (ECG)	Flavonoids	Flavanols	Inhibit cyclooxygenase-1 [2].				√	
4	Genistein 7-O-Glucoside (Genistin)	Flavonoids	Isoflavones	Promote apoptosis of breast cancer cells [3];<br>Anti-adipogenic activity [4].			√		
5	Naringenin chalcone	Flavonoids	Flavanones	Anti-inflammatory and antiallergic activities [5].				√	
6	Quercetin 4'-O-glucoside (Spiraeoside)	Flavonoids	Flavonols	Antiallergic, anti-inflammatory and antitumor activities [6].					√
7	Quercetin 7-O-β-D-Glucuronide	Flavonoids	Flavonols	Modulate the LDL oxidation [7].					√
8	5-O-p-Coumaroyl shikimic acid	Phenolic acids	Hydroxycyclic acids	-	√				
9	Ethyl 3,4-Dihydroxybenzoate (Ethyl protocatechuate)	Phenolic acids	Hydroxybenzoic acids	Inhibits prolyl-hydroxylase and induces cell autophagy and apoptosis in ESCC cells [8];<br>Bone protecting-effect [9].				√	
10	1-O-Feruloyl quinic acid	Phenolic acids	Hydroxycyclic acids	-		√			
11	Catechin gallate	Phenolic acids	Hydroxybenzoic acids	Inhibit the activity of COX-1 and COX-2 enzymes [10].				√	
12	Formononetin	Flavonoids	Isoflavones	Inhibits FGFR2, angiogenesis and tumor growth in preclinical models [11].					√

13	7-O-Methyleriodictyol	Flavonoids	Flavanols	A flavanone phytoalexin associated with disease resistance in rice plants [12].		√			
14	Cannabiscitrin	Others	Others	-				√	
15	O-Phosphocholine	Alkaloids	Alkaloids	-		√			
16	Citramalate	Organic acids	Organic acids	-			√		
17	(±)-jasmonic acid	Organic acids	Organic acids	-		√			
18	Methyl jasmonate	Organic acids	Organic acids	Enhance secondary metabolites and antioxidant activity [13].		√			
19	Sweroside	Terpenoids	monoterpenes	Cytoprotective and anti-osteoporotic [14]; hepatoprotective effect [15].					√
20	Phlorizin	Flavonoids	Chalcones	Antioxidant properties [16];<br>Non-selective SGLT inhibitor [17].				√	
21	Eriodictyol 7-O-glucoside	Flavonoids	Flavanones	Nrf2 activator [18].				√	
22	Pinobanksin	Flavonoids	Flavanols	Apoptotic induction in a B-cell lymphoma cell line [19];<br>Antiangiogenic effect [20].				√	
23	4-Hydroxybenzaldehyde	Phenolic acids	Hydroxybenzoic acids	-					√
24	Cinnamic acid	Phenolic acids	Hydroxycyclic acids	Anticancer effects in vitro and antibacterial activity [21].		√			
25	Riboprine	Phenolic acids	HAs	An autophagy inhibitor with anti-melanoma activity [22].					√
26	Sinapinaldehyde	Phenolic acids	Hydroxycyclic acids	-					√
27	Brevifolin carboxylic acid	Phenolic acids	Hydroxybenzoic acids	-	√				
28	5-Galloylshikimic acid	Phenolic acids	Hydroxybenzoic acids	-	√				
29	3-Galloylshikimic acid	Phenolic acids	Hydroxybenzoic acids	-	√				
30	Trihydroxycinnamoylquinic acid	Phenolic acids	Hydroxycyclic acids	-				√	
31	1,6-Di-O-Galloyl-D-Glucose	Phenolic acids	Hydroxybenzoic acids	Inhibitor of trypanosomal phosphofructokinase [23].			√		
32	Apigenin 7-O-glucoside(Cosmosiin)	Flavonoids	Flavones	Anti-proliferative and antioxidant activity [24];<br>Scavenge reactive oxygen species [25].			√		
33	Apigenin 5-O-glucoside	Flavonoids	Flavones	Anti-inflammatory activity [26].			√		
34	Tetahydroxy-flavone-7-O-β-D-glucuronide	Flavonoids	Flavones	-			√		
35	Luteolin-O-sinapoylhexoside	Flavonoids	Flavones	-	√				
36	Myricetin	Flavonoids	Flavonols	Anti-oxidant activity [27];<br>Anti-cancer activity [28];<br>Anti-inflammatory activities [29].			√		
37	Kaempferol-3-O-rutinoside(Nicotiflorin)	Flavonoids	Flavonols	Play a pharmacological role in VEGF-C-mediated anti-inflammation [30].			√		
38	Kaempferol-3-O-robinobioside(Biorobin)	Flavonoids	Flavonols	Inhibit the human lymphocyte proliferation in vitro [31].			√		

39	Kaempferol-3-O-glucoside-7-O-rhamnoside	Flavonoids	Flavonols	-			√		
40	Isorhamnetin-3-O-rutinoside (Narcissin)	Flavonoids	Flavonols	Antioxidant activity [32].			√		
41	6-Hydroxykaempferol-3,6-O-Diglucoside	Flavonoids	Flavonols	Antioxidant and anticancer activities [33].				√	
42	Robinin(Kaempferol-3-O-gal-rham-7-O-rham)	Flavonoids	Flavonols	Ameliorate oxidized low-density lipoprotein induced inflammatory [34];<br>Improve the reduction of inflammation in experimental arthritis [35].			√		
43	1-Methylhistamine	Others	Others	-					√
44	4-Methyl-5-thiazoleethanol	Others	Others	-			√		
45	Ethyl gallate	Phenolic acids	Hydroxybenzoic acids	Scavenger of hydrogen peroxide; Induce apoptosis in HL-60 cell [36].				√	
46	Ursolic acid	Terpenoids	triterpenoids	Resist cancer cells [37];<br>Decreases the serum levels of tumor necrosis factor-α, interleukin-6, and interleukin-1β [38].				√	
47	Methoxyursolic acid	Terpenoids	triterpenoids	-					√
48	Pyridoxine	Vitamins	Vitamins	Antioxidant effects in cell model of Alzheimer's disease [39].	√				
49	Riboflavin	Vitamins	Vitamins	Antinociceptive activity [40];<br>Ameliorates cognitive impairment [41].			√		
50	Prunetin	Flavonoids	Isoflavones	Anti-inflammatory activity [42];<br>A potent human aldehyde dehydrogenases inhibitor [43].				√	
51	γ-Aminobutyric acid	Organic acids	Organic acids	Calming effect by blocking specific signals of central nervous system [44].		√			
52	Citraconic acid	Organic acids	Organic acids	Human endogenous metabolite [45].	√				
53	4-Acetamidobutyric acid	Organic acids	Organic acids	Exhibit potential for early diabetic kidney disease diagnosis [46].				√	
54	L-(+)-Tartaric acid	Organic acids	Organic acids	Antihyperglycemic and antidyslipidemic effects [47].				√	
55	SubericAcid	Organic acids	Organic acids	-					√
56	Anchoic acid	Organic acids	Organic acids	-					√
57	6-hydroxy-5,7,4'-trimethoxyflavone	Flavonoids	Flavones	-				√	
58	Tetramethylluteolin (3',4',5,7-Tetramethoxyflavone)	Flavonoids	Flavones	-	√				
59	Kaempferol-3-O-β-D-glucuronide	Flavonoids	Flavonols	Inhibit pro-inflammatory mediators like IL-1β, NO, PGE2, and LTB4 [48].			√		
60	Tercatain	Phenolic acids	Hydroxybenzoic acids	-	√				
61	benzoylmalic acid	Phenolic acids	Hydroxybenzoic acids	-	√				
62	feruloylmalic acid	Phenolic acids	Hydroxycyclic acids	-					√

63	feruloylsinapoyltartaric acid	Phenolic acids	Hydroxycyclic acids	-				√	
64	Galloyl-HHDP(Hexahydroxydiphenoyl)-glueopy ranose	Phenolic acids	Hydroxybenzoic acids	-	√				
65	Angelicin	Coumarins	Coumarins	Anti-cancer activity [49];<br>Antiviral activity [50];<br>Anti-inflammatory activity [51].			√		
66	2'-Hydoxy,5-methoxy Genistein-O-rhamnosyl-glucoside	Flavonoids	Isoflavones	-			√		
67	Farrerol 7-O-glucoside	Flavonoids	Flavanones	-	√				
68	Eucommia	Others	Others	-					√
69	Luteolin-7-O-β-D-gentiobioside	Flavonoids	Flavones	-				√	
70	Vanillic acid glycoside	Phenolic acids	Hydroxybenzoic acids	-				√	
71	Kaempferol-3-O-(6''-acetyl)-glucoside	Flavonoids	Flavonols	-			√		
72	Sexangularetin 3-glucoside-7-rhamnoside	Flavonoids	Flavonols	-			√		
73	Limocitrin 7-glucoside	Flavonoids	Flavonols	-		√			
74	Dihydrosedinine	Alkaloids	Alkaloids	-			√		
75	Santamarin	Terpenoids	sesquiterpenes	-			√		
76	Ixerin D	Terpenoids	diterpenes	-	√				
77	Silibinin	Flavonoids	Flavanols	Apoptosis, hepatoprotective, antioxidant, anti-inflammatory and anti-cancer activity[52, 53].			√		
78	Scutellarin(Scutellarein-7-O-glucuronide)	Flavonoids	Flavones	Work against HIV-1IIIB, HIV-1(74V) and HIV-1KM018 [54];<br>Inhibit RANKL-mediated MAPK and NF-κB signaling pathway, including JNK1/2, p38, ERK1/2, and IκBα phosphorylation [55].			√		
79	Pratensein 7-O-glucopyranoside	Flavonoids	Flavones	-	√				
80	Diosmetin-7-O-glucuronide	Flavonoids	Flavones	-	√				
81	Patuletin-3-O-β-D-glucopyranoside	Flavonoids	Flavones	Therapeutic potential against oxidative stress-related and inflammatory disorders [56].	√				
82	Luteolin-7-O-rutinoside	Flavonoids	Flavones	Reduce the release of TNF-alpha, IL-6, and IL-1 beta; Inhibit PI3K/AKT/AMPK/NF-kappa B pathway, and reduce oxidative stress [57].			√		

83	5,2'-Dihydroxy-7,8-dimethoxyflavone glycosides	Flavonoids	Flavones	-				√	
84	3-O-Digalloyl quinic acid	Phenolic acids	Hydroxybenzoic acids	-			√		
85	Di-O-Glucose-quinic acid	Phenolic acids	HAs	-	√				
86	Gemin D	Phenolic acids	Hydroxybenzoic acids	Antioxidant and antitumoral activities [58].	√				
87	Nobotanin D	Phenolic acids	Hydroxybenzoic acids	Antioxidant and antiglycation activities [59].	√				
88	Cuspinin	Phenolic acids	Hydroxybenzoic acids	-	√				
89	Ailanindole	Alkaloids	Alkaloids	-				√	
90	Ayapin	Coumarins	Coumarins	-					√
91	Tubuloside C	Phenolic acids	Hydroxycyclic acids	-			√		
92	Sinapaldehyde Glucoside	Phenolic acids	Hydroxycyclic acids	-		√			
93	Maplexin C (2,3-Di-O-Galloyl-1,5-Anhydro-D-Glucitol)	Phenolic acids	Hydroxybenzoic acids	Anticancer in vitro and antiproliferative effects [60].	√				
94	2,3-Di-O-Galloyl-D-Glucose	Phenolic acids	Hydroxybenzoic acids	-				√	
95	Methyl 4,6-di-O-galloyl-D-glucoside	Phenolic acids	Hydroxybenzoic acids	-	√				
96	Geniposide	Terpenoids	monoterpenes	Anti-diabetic; Reduces oxidative stress, inflammation and fibrosis [61].				√	
97	Quercetin-3-sambubioside	Flavonoids	Flavonols	-					√
98	Syringic Aldehyde-glucoside	Phenolic acids	Hydroxybenzoic acids	-			√		
99	1'-O-vanilloyl-β-D-glucoside	Phenolic acids	Hydroxybenzoic acids	-				√	
100	3-Prenyl-4-O-β-D-glucopyranosyloxy-4-hydroxyl-benzoic acid	Phenolic acids	Hydroxybenzoic acids	-			√		
101	Quercetin-O-pentosyl-O-rhamnoside-O-glucoside	Flavonoids	Flavonols	-					√
102	dihydrodehydrodiconiferyl alcohol 4-O-β-D-glucopyranosides	Lignans	Lignans	-				√	
103	(-)-secoisolariciresinol 4-O-β-D-glucopyranoside	Lignans	Lignans	-			√		
104	p-Coumaroyleuscaphic acid	Terpenoids	triterpenoids	-			√		
105	3-Indoleacrylic acid	Alkaloids	Alkaloids	-				√	
106	Kaempferol 3-O-β-D-neohesperidoside	Flavonoids	Flavonols	Insulinomimetic effect on the rat soleus muscle [62].			√		
107	Quercetin 3-O-β-D-xylopyranosyl(1→2)-β-D-galactopyranoside	Flavonoids	Flavonols	-					√

108	Corosolic acid	Terpenoids	triterpenoids	Kinase C inhibitor [63];<br>Anti-angiogenic, anti-lymphangiogenic and anti-cancer effects [64].			√		
109	Luteolin-O-rutinoside-O-rhamnoside	Flavonoids	Flavones	-			√		
110	Methyl neochebulagate	Others	Others	Cytotoxic activities to HL60 cell line [65].	√				
111	1,4-di-O-galloyl-β-D-glucose	Phenolic acids	Hydroxybenzoic acids	-			√		
112	2α-hydroxyursolic acid	Terpenoids	triterpenoids	Induce non-apoptotic cell death in cancer cells [66].				√	
113	5,7,3',4',5'-pentahydroxydihydroflavone	Flavonoids	Flavanones	-	√				
114	Myricetin-O-rhamnoside	Flavonoids	Flavonols	DPPH free radical scavenging activity [67].					√
115	7S,8R-threo-3',9,9'-trihydroxy-3-methoxy-4',7-epoxy-neolignan-4-O-α-L-rhamnopyranoside	Lignans	Lignans	-	√				
116	monogalloyl-diglucose	Phenolic acids	Hydroxybenzoic acids	-	√				
117	Caffeoyl feruloyl shikimic acid	Phenolic acids	Hydroxycyclic acids	-					√
118	Scopoletin-Beta-D-Glucuronide	Coumarins	Coumarins	-		√			
119	Galangin-7-glucoside	Flavonoids	Flavones	-			√		
120	Luteolin-O-glucuronate-O-rhamnoside	Flavonoids	Flavones	-			√		
121	Dunalianoside C	Phenolic acids	Hydroxycyclic acids	-				√	
122	5,7,8,4'-Tetramethoxyflavone	Flavonoids	Flavones	-	√				
123	Luteolin 7-O-neohesperidoside(Lonicerin)	Flavonoids	Flavones	Prevent inflammation [68];<br>Apoptosis in LPS-induced acute lung injury [69].			√		
124	Isolariciresinol 9'-O-Glucoside	Lignans	Lignans	-				√	
125	5'-methoxyisolariciresinol-9'-O-β-D-xylopyranoside	Lignans	Lignans	-	√				

References

1. Thors, L.; Burston, J.J.; Alter, B.J.; McKinney, M.K.; Cravatt, B.F.; Ross, R.A.; Pertwee, R.G.; Gereau, R.W.; Wiley, J.L.; Fowler, C.J. Biochanin A, a naturally occurring inhibitor of fatty acid amide hydrolase. Brit. J. *Pharmacol*. **2010**, *160(3)*, 549-560. doi: https://doi.org/10.1111/j.1476-5381.2010.00716.x

2. Waffo-Teguo, P.; Hawthorne, M.E.; Cuendet, M.; Merillon, J.M.; Kinghorn, A.D.; Pezzuto, J.M.; Mehta, R.G. Potential cancer-chemopreventive activities of wine stilbenoids and flavans extracted from grape (*Vitis vinifera*) cell cultures. *Nutr. Cancer*. **2001**, *40(2)*, 173-179.doi: 10.1207/S15327914NC402_14

3.Hwang, S.T.; Yang, M.H.; Baek, S.H.; Um, J.; Ahn, K.S. Genistin attenuates cellular growth and promotes apoptotic cell death breast cancer cells through modulation of ERalpha signaling pathway. *Life Sci*. **2020**, *263*, 118594. doi:

https://doi.org/10.1016/j.lfs.2020.118594
4.Choi, Y.R.; Shim, J.; Kim, M.J. Genistin: a novel potent anti-adipogenic and anti-lipogenic agent. *Molecules*. **2020**, *25(9),* 2042. doi: 10.3390/molecules25092042
5. Escribano-Ferrer, E.; Regué, J.Q.; Garcia-Sala, X.; Montañés, A.B.; Lamuela-Raventos, R.M. *In vivo* anti-inflammatory and antiallergic activity of pure naringenin, naringenin chalcone, and quercetin in mice. *J. Nat. Prod*. **2019**, *82(2)*, 177-182. doi: 10.1021/acs.jnatprod.8b00366
6. Liu, H.Y.; Zhang, Z.Y.; Zhang, L.; Yao, X.L.; Zhong, X.M.; Cheng, G.C.; Wang, L.F.; Wan, Q.L. Spiraeoside protects human cardiomyocytes against high glucose-induced injury, oxidative stress, and apoptosis by activation of PI3K/Akt/Nrf2 Pathway. *J. Biochem. Mol. Toxic.* **2020**, *34(10)*, e22548. doi: 10.1002/jbt.22548
7. Janisch, K.M.; Williamson, G.; Needs, P.; Plumb, G.W. Properties of quercetin conjugates: modulation of LDL oxidation and binding to human serum albumin. *Free Radical Res*. **2004**, *38(8)*, 877-884. doi: 10.1080/10715760410001728415
8. Han, B.; Li, W.; Sun, Y.L.; Zhou, L.P.; Xu, Y.; Zhao, X.H. A prolyl-hydroxylase inhibitor, ethyl-3,4-dihydroxybenzoate, induces cell autophagy and apoptosis in esophageal squamous cell carcinoma cells via up-regulation of BNIP3 and N-myc downstream-regulated gene-1. *Plos One*. **2014**, *9(9)*, e107204. doi: 10.1371/journal.pone.0107204
9. Kwon, B.J.; Lee, M.H.; Koo, M.A.; Han, J.J.; Park, J.C. Ethyl-3,4-dihydroxybenzoate with a dual function of induction of osteogenic differentiation and inhibition of osteoclast differentiation for bone tissue engineering. *Tissue Eng. Pt. A*. **2014**, *20(21-22)*, 2975-2984. doi: 10.1089/ten.tea.2013.0567
10. Babich, H.; Zuckerbraun, H.L.; Weinerman, S.M. *In vitro* cytotoxicity of (-)-catechin gallate, a minor polyphenol in green tea. *Toxicol. Lett*. **2007**, *171(3)*, 171-180. doi: https://doi.org/10.1016/j.toxlet.2007.05.125
11. Wu, X.Y.; Xu, H.; Wu, Z.F.; Chen, C.; Liu, J.Y.; Wu, G.N.; Yao, X.Q.; Li, G.; Shen, L. Formononetin, a novel FGFR2 inhibitor, potently inhibits angiogenesis and tumor growth in preclinical models. *Oncotarget*. **2015**, *6(42)*, 44563–44578. https://doi.org/10.18632/oncotarget.6310
12. Katsumata, S.; Hamana, K.; Horie, K.; Toshima, H.; Hasegawa, M. Identification of sternbin and naringenin as detoxified metabolites from the rice flavanone phytoalexin sakuranetin by pyricularia oryzae. *Chem. Biodivers*. **2017**, *14(2)*, e1600240. doi: https://doi.org/10.1002/cbdv.201600240
13. Reyes-Díaz, M.; Lobos, T.; Cardemil, L.; Nunes-Nesi, A.; Retamales, J.; Jaakola, L.; Alberdi, M.; Ribera-Fonseca, A. Methyl Jasmonate: an alternative for improving the quality and health properties of fresh fruits. *Molecules*. **2016**, *21(6)*, 567. doi: 10.3390/molecules21060567
14. Öztürk, N.; Korkmaz, S.; Ozturk, Y.; Baser, K.H.C. Effects of gentiopicroside, sweroside and swertiamarine, secoiridoids from gentian (*Gentiana lutea* ssp. symphyandra), on cultured chicken embryonic fibroblasts. *Planta Med*. **2006**, *72(4)*, 289-294. doi: 10.1055/s-2005-916198
15. Jeong, Y.T.; Jeong, S.C.; Hwang, J.S.; Kim, J.H. Modulation effects of sweroside isolated from the Lonicera japonica on melanin synthesis. *Chem-Biol. Interact*. **2015**, *238*, 33-39. doi: 10.1016/j.cbi.2015.05.022
16. Zhang, J.; Wu, D.; Tang, L.; Hu, X.; Zeng, Z.; Wu, W.; Geng, F.; Li, H. Evaluation of the binding affinity and antioxidant activity of phlorizin to pepsin and trypsin. *Food Science and Human Wellness*. **2024**, *13(1)*, 392-400. doi: 10.26599/fshw.2022.9250033
17. Katsuda, Y.; Sasase, T.; Tadaki, H.; Mera, Y.; Motohashi, Y.; Kemmochi, Y.; Toyoda, K.; Kakimoto, K.; Kume, S.; Ohta, T. Contribution of hyperglycemia on diabetic complications in obese type 2 diabetic SDT fatty rats: effects of SGLT inhibitor phlorizin. *Exp. Anim. Tokyo*. **2015**, *64(2)*, 161-169. doi: 10.1538/expanim.14-0084
18. Hu, Q.W.; Zhang, D.D.; Wang, L.M.; Lou, H.X.; Ren, D.M. Eriodictyol-7-O-glucoside, a novel Nrf2 activator, confers protection against cisplatin-induced toxicity. *Food Chem. Toxico*l. **2012**, *50(6)*, 1927-1932. doi: 10.1016/j.fct.2012.03.059
19. Alday, E.; Valencia, D.; Carreño, A.L.; Picerno, P.; Piccinelli, A.L.; Rastrelli, L.; Robles-Zepeda, R.; Hernandez, J.; Velazquez, C. Apoptotic induction by pinobanksin and some of its ester derivatives from Sonoran propolis in a B-cell lymphoma cell line. *Chem-Biol. Interact*. **2015**, *242*, 35-44. doi: 10.1016/j.cbi.2015.09.013
20. Bang, H.J.; Ahn, M.R. Antiangiogenic effect of pinobanksin on human umbilical vein endothelial cells. *J. Funct. Foods*. **2021**, *79*, 104408. doi: 10.1016/j.jff.2021.104408
21. Liu, L.; Hudgins, W.R.; Shack, S.; Yin, M.Q.; Samid, D. Cinnamic acid: a natural product with potential use in cancer intervention. *Inter. J. Cancer*. **1995**, *62(3)*, 345-350. doi: 10.1002/ijc.2910620319
22. Cheng, H.P.; Yang, X.H.; Lan, L.; Xie, L.J.; Chen, C.; Liu, C.; Chu, J.F.; Li, Z.Y.; Liu, L.; Zhang, T.Q.; Luo, D.Q.; Cheng, L. Chemical deprenylation of N6-isopentenyladenosine (i6A) RNA. *Angew. Chem. Int. Edit*. **2020**, *59(26)*, 10645-10650. doi: https://doi.org/10.1002/anie.202003360
23. El-Hawary, S.S.; Mohammed, R.; Lithy, N.M.; AbouZid, S.F.; Mansour, M.A.; Almahmoud, S.A.; Huwaimel, B.; Amin, E. Digalloyl Glycoside: A Potential Inhibitor of Trypanosomal PFK from Euphorbia abyssinica. *J.F. Gmel. Plants*. **2022**, *11*, 173. https://doi.org/10.3390/plants11020173
24. Samet, I.; Villareal, M. O.; Motojima, H.; Han, J.; Sayadi, S.; Isoda, H. Olive leaf components apigenin 7-glucoside and luteolin 7-glucoside direct human hematopoietic stem cell differentiation towards erythroid lineage. *Differentiation*. **2015**, *89(5)*, 146-155. doi: 10.1016/j.diff.2015.07.001
25. Nasr Bouzaiene, N.; Chaabane, F.; Sassi, A.; Chekir-Ghedira, L.; Ghedira, K. Effect of apigenin-7-glucoside, genkwanin and naringenin on tyrosinase activity and melanin synthesis in B16F10 melanoma cells. *Life Sci*. **2016**, *144*, 80-85. doi: https://doi.org/10.1016/j.lfs.2015.11.030
26. Feng, Q.M.; Li, B.X.; Feng, Y.; Li, X.Y.; Ma, X.R.; Wang, H.F.; Chen, G. Isolation and identification of two new compounds from the twigs and leaves of *Cephalotaxus fortunei*. *J. Nat. Med-Tokyo*. **2019**, *73(3)*, 653-660. doi: 10.1007/s11418-019-01308-5
27. Semwal, D.K.; Semwal, R.B.; Combrinck, S.; Viljoen, A. Myricetin: A dietary molecule with diverse biological activities. *Nutrients*. **2016**, *8(2)*, 90. doi: 10.3390/nu8020090
28. Phillips, P.A.; Sangwan, V.; Borja-Cacho, D.; Dudeja, V.; Vickers, S.M.; Saluja, A.K. Myricetin induces pancreatic cancer cell death via the induction of apoptosis and inhibition of the phosphatidylinositol 3-kinase (PI3K) signaling pathway.

Cancer Lett. **2011**, *308(2)*, 181-188. doi: 10.1016/j.canlet.2011.05.002

29. Zhong, R.T.; Miao, L.C.; Zhang, H.L.; Tan, L.H.; Zhao, Y.X.; Tu, Y.B.; Prieto, M.A.; Simal-Gandara, J.; Chen, L.; He, C.W.; Cao, H. Anti-inflammatory activity of flavonols via inhibiting MAPK and NF-Kappa B signaling pathways in RAW264.7 macrophages. *Current Research in Food Science*. **2022**, *5*, 1176-1184. doi: 10.1016/j.crfs.2022.07.007
30. Hu, W.H.; Dai, D.K.; Zheng, B.; Duan, R.; Chan, G.; Dong, T.; Qin, Q.W.; Tsim, K. The binding of kaempferol-3-O-rutinoside to vascular endothelial growth factor potentiates anti-inflammatory efficiencies in lipopolysaccharide-treated mouse macrophage RAW264.7 cells. *Phytomedicine*. **2021**, *80*, 153400. doi: 10.1016/j.phymed.2020.153400
31. Brochado, C.D.O.; Almeida, A.P.D.; Barreto, B.P.; Costa, L.P.; Ribeiro, L.S.; Pereira, R.L.D.C.; Koatz, V.L.G.; Costa, S.S. Flavonol robinobiosides and rutinosides from *Alternanthera brasiliana* (*Amaranthaceae*) and their effects on lymphocyte proliferation *in vitro*. *J. Brazil. Chem. Soc*. **2003**, *14(3)*, 449-451. doi: 10.1590/S0103-50532003000300018
32. Su, B.N.; Pawlus, A.D.; Jung, H.A.; Keller, W.J.; McLaughlin, J.L.; Kinghorn, A.D. Chemical constituents of the fruits of *Morinda citrifolia* (Noni) and their antioxidant activity. *J. Nat. Prod*. **2005**, *68(4)*, 592-595. doi: 10.1021/np0495985
33. Zhou, X.H.; Wang, M.; Li, H.; Ye, S.L.; Tang, W.R. Widely targeted metabolomics reveals the antioxidant and anticancer activities of different colors of *Dianthus caryophyllus*. *Frontiers in Nutrition*. **2023**, *10*. doi: 10.3389/fnut.2023.1166375
34. Janeesh, P.A.; Sasikala, V.; Dhanya, C.R.; Abraham, A. Robinin modulates TLR/NF-κB signaling pathway in oxidized LDL induced human peripheral blood mononuclear cells. *Int. Immunopharmacol*. **2014**, *18(1)*, 191-197. doi: https://doi.org/10.1016/j.intimp.2013.11.023
35. Tsiklauri, L.; Svík, K.; Chrastina, M.; Ponist, S.; Dráfi, F.; Slovák, L.; Alnaia, M.; Kemertelidze, E.; Bauerova, K. Bioflavonoid robinin from *Astragalus falcatus* lam. mildly improves the effect of metothrexate in rats with adjuvant arthritis. *Nutrients*. **2021**, *13(4)*, 1268. doi: 10.3390/nu13041268
36. Kim, W.H.; Song, H.O.; Choi, H.J.; Bang, H.I.; Choi, D.Y.; Park, H. Ethyl gallate induces apoptosis of HL-60 cells by promoting the expression of caspases-8, -9, -3, apoptosis-inducing factor and endonuclease G. *Int. J. Mol. Sci*. **2012**, *13(9)*, 11912-11922. doi: 10.3390/ijms130911912
37. Li, R.; Wang, X.; Zhang, X.H.; Chen, H.H.; Liu, Y.D. Ursolic acid promotes apoptosis of SGC-7901 gastric cancer cells through ROCK/PTEN mediated mitochondrial translocation of cofilin-1. *Asian Pacific Journal of Cancer Prevention*. **2014**, *15(22)*, 9593-9597. doi: 10.7314/APJCP.2014.15.22.9593
38. Hu, Z.S.; Gu, Z.L.; Sun, M.N.; Zhang, K.; Gao, P.H.; Yang, Q.W.; Yuan, Y. Ursolic acid improves survival and attenuates lung injury in septic rats induced by cecal ligation and puncture. *J. Surg. Res*. **2015**, *194(2)*, 528-536. doi: 10.1016/j.jss.2014.10.027
39. Li, C.; Wang, R.L.; Hu, C.T.; Wang, H.; Ma, Q.Y.; Chen, S.S.; He, Y. Pyridoxine exerts antioxidant effects in cell model of Alzheimer's disease via the Nrf-2/HO-1 pathway. *Cell. Mol. Biol*. **2018**, *64(10)*, 119-124. doi: 10.14715/cmb/2018.64.10.19
40. Braga, A.V.; Costa, S.; Rodrigues, F.F.; Melo, I.; Morais, M.I.; Coelho, M.M.; Machado, R.R. Thiamine, riboflavin, and nicotinamide inhibit paclitaxel-induced allodynia by reducing TNF-alpha and CXCL-1 in dorsal root ganglia and thalamus and activating ATP-sensitive potassium channels. *Inflammopharmacology*. **2020**, *28(1)*, 201-213. doi: 10.1007/s10787-019-00625-1
41. Zhang, M.R.; Chen, H.Q.; Zhang, W.L.; Liu, Y.; Ding, L.Y.; Gong, J.W.; Ma, R.F.; Zheng, S.H.; Zhang, Y.L. Biomimetic remodeling of microglial riboflavin metabolism ameliorates cognitive impairment by modulating neuroinflammation. *Adv. Sci*. **2023**, *10(12)*. doi: 10.1002/advs.202300180
42. Hu, H.; Li, H. Prunetin inhibits lipopolysaccharide-induced inflammatory cytokine production and MUC5AC expression by inactivating the TLR4/MyD88 pathway in human nasal epithelial cells. *Biomed. Pharmacother*. **2018**, *106*, 1469-1477. doi: https://doi.org/10.1016/j.biopha.2018.07.093
43. Sheikh, S.; Weiner, H. Allosteric inhibition of human liver aldehyde dehydrogenase by the isoflavone prunetin. *Biochem. Pharmacol*. **1997**, *53(4)*, 471-478. doi: https://doi.org/10.1016/S0006-2952(96)00837-4
44. Okada, R.; Awasaki, T.; Ito, K. Gamma-aminobutyric acid (GABA)-mediated neural connections in the Drosophila antennal lobe. *J. Comp. Neurol*. **2009**, *514(1)*, 74-91. doi: https://doi.org/10.1002/cne.21971
45. Pulat, M.; Akalın, G.O.; Karahan, N.D. Lipase release through semi-interpenetrating polymer network hydrogels based on chitosan, acrylamide, and citraconic acid. *Rtif. Cell. Nanomed. B*. **2014**, *42(2)*, 121-127. doi: 10.3109/21691401.2013.794356
46. Pan, Y.J.; Yang, H.; Chen, T.C.; Jin, J.; Ruan, L.Y.; Hu, L.; Chen, L. Extracellular vesicles metabolic changes reveals plasma signature in stage-dependent diabetic kidney disease. *Renal Failure*. **2022**, *44(1)*, 1840-1849. doi: 10.1080/0886022X.2022.2118067
47. Amssayef, A.; Eddouks, M. *In vivo* antihyperglycemic and antidyslipidemic effects of L-Tartaric acid. *Cardiovascular & Hematological Disorders Drug Targets*. **2022**, *22(3)*, 185-198. doi: 10.2174/1871529X23666221202091848
48. Khajuria, V.; Gupta, S.; Sharma, N.; Tiwari, H.; Bhardwaj, S.; Dutt, P.; Satti, N.; Nargotra, A.; Bhagat, A.; Ahmed, Z. Kaempferol-3-O-β-D-glucuronate exhibit potential anti-inflammatory effect in LPS stimulated RAW 264.7 cells and mice model. *Int. Immunopharmacol*. **2018**, *57*, 62-71. doi: 10.1016/j.intimp.2018.01.041
49. Rahman, M.A.; Kim, N.H.; Yang, H.; Huh, S.O. Angelicin induces apoptosis through intrinsic caspase-dependent pathway in human SH-SY5Y neuroblastoma cells. *Mol. Cell. Biochem*. **2012**, *369(1-2)*, 95-104. doi: 10.1007/s11010-012-1372-1
50. Cho, H.; Jeong, S.; Park, J.; Han, J.; Kang, H.; Lee, D.; Song, M.J. Antiviral activity of angelicin against gammaherpesviruses. *Antivir. Res*. **2013**, *100(1)*, 75-83. doi: https://doi.org/10.1016/j.antiviral.2013.07.009
51. Liu, F.; Sun, G.Q.; Gao, H.Y.; Li, R.S.; Soromou, L.W.; Chen, N.; Deng, Y.H.; Feng, H.H. Angelicin regulates LPS-induced inflammation via inhibiting MAPK/NF-κB pathways. *J. Surg. Res*. **2013**, *185(1)*, 300-309. doi: https://doi.org/10.1016/j.jss.2013.05.083
52. Zappavigna, S.; Vanacore, D.; Lama, S.; Potenza, N.; Russo, A.; Ferranti, P.; Dallio, M.; Federico, A.; Loguercio, C.; Sperlongano, P. Caraglia, M.; stiuso, P. Silybin-induced apoptosis occurs in parallel to the increase of ceramides synthesis and miRNAs secretion in human hepatocarcinoma cells. *Int. J. Mol. Sci*. **2019**, *20(9)*, 2190. doi: 10.3390/ijms20092190
53. Sun, R.B.; Xu, D.; Wei, Q.L.; Zhang, B.L.; Aa, J.Y.; Wang, G.J.; Xie, Y. Silybin ameliorates hepatic lipid accumulation and modulates global metabolism in an NAFLD mouse model. *Biomed. Pharmacother*. **2020**, *123*, 109721. doi:

10.1016/j.biopha.2019.109721

54. Zhang, G.H.; Wang, Q.; Chen, J.J.; Zhang, X.M.; Tam, S.C.; Zheng, Y.T. The anti-HIV-1 effect of scutellarin. *Biochem. Bioph. Res. Co*. **2005**, *334(3)*, 812-816. doi: 10.1016/j.bbrc.2005.06.166

55. Zhao, S.; Sun, Y.; Li, X.L.; Wang, J.C.; Yan, L.Q.; Zhang, Z.; Wang, D.X.; Dai, J.H.; He, J.; Wang, S. G. Scutellarin inhibits RANKL-mediated osteoclastogenesis and titanium particle-induced osteolysis via suppression of NF-κB and MAPK signaling pathway. *Int. Immunopharmacol*. **2016**, *40*, 458-465. doi: 10.1016/j.intimp.2016.09.031

56. Corrêa, W.R.; Serain, A.F.; Netto, L.A.; Marinho, J.V.N.; Arena, A.C.; Aquino, D.F.D.S.; Oliveira, A.M.K.; Júnior, A.J.; Bernal, L.P.T.; Kassuya, C.A.L.; Salvador, M. J. Anti-inflammatory and antioxidant properties of the extract, tiliroside, and patuletin 3-o-β-d-glucopyranoside from *Pfaffia townsendii* (*Amaranthaceae*). *Evid-Based. Compl. Alt.* **2018**, *2018*, 6057579. doi: 10.1155/2018/6057579

57. Xiong, Z.W.; Cui, Y.S.; Wu, J.H.; Shi, L.Y.; Wen, Q.; Yang, S.L.; Feng, Y.L. Luteolin-7-O-rutinoside from *Pteris cretica* L. var. *nervosa* attenuates LPS/D-gal-induced acute liver injury by inhibiting PI3K/AKT/AMPK/NF-kappa B signaling pathway. *N-S. Arch. Pharmacol*. **2022**, *395(10)*, 1283-1295. doi: 10.1007/s00210-022-02266-8

58. Carneiro, C.C.; Moraes, A.V.; Fernandes, A.S.; Santos, S.D.; Silva, D.; Chen, L.C. Cytotoxic and chemopreventive effects of Gemin D against different mutagens using *in vitro* and *in vivo* assays. *Anti-Cancer Agent. Me*. **2017**, *17(5)*, 712-718. doi: 10.2174/1871520616666160906092502

59. Yasuda, M.; Ikeoka, M.; Kondo, S.I. Skin-related enzyme inhibitory activity by hydrolyzable polyphenols in water chestnut (*Trapa natans*) husk. *Biosci. Biotech. Bioch*. **2021**, *85(3)*, 666-674. doi: 10.1093/bbb/zbaa076

60. González-Sarrías, A.; Yuan, T.; Seeram, N.P. Cytotoxicity and structure activity relationship studies of maplexins A–I, gallotannins from red maple (*Acer rubrum*). *Food Chem. Toxicol*. **2012**, *50(5)*, 1369-1376. doi: https://doi.org/10.1016/j.fct.2012.02.031

61. Dusabimana, T.; Park, E.J.; Je, J.; Jeong, K.; Yun, S.P.; Kim, H.J.; Kim, H.; Park, S.W. Geniposide improves diabetic nephropathy by enhancing ulk1-mediated autophagy and reducing oxidative stress through ampk activation. *Int. J. Mol. Sci*. **2021**, *22(4)*, 1651. doi: 10.3390/ijms22041651

62. Zanatta, L.; Rosso, A.; Folador, P.; Figueiredo, M.S.; Pizzolatti, M.G.; Leite, L.D.; Silva, F.R. Insulinomimetic effect of kaempferol 3-neohesperidoside on the rat soleus muscle. *J. Nat. Prod*. **2008**, *71(4)*, 532–535. https://doi.org/10.1021/np070358+

63. Ahn, K.S.; Hahm, M.S.; Park, E.J.; Lee, H.K.; Kim, I.H. Corosolic acid isolated from the fruit of Crataegus pinnatifida var. psilosa is a protein kinase C inhibitor as well as a cytotoxic agent. *Planta Med*. **1998**, *64(5)*, 468-470. doi: 10.1055/s-2006-957487

64. Yoo, K.H.; Park, J.; Lee, D.Y.; Hwang-Bo, J.; Baek, N.I.; Chung, I.S. Corosolic acid exhibits anti-angiogenic and anti-lymphangiogenic effects on *in vitro* endothelial cells and on an *in vivo* ct-26 colon carcinoma animal model. *Phytotherapy Res*. **2015**, *29(5)*, 714-723. doi: https://doi.org/10.1002/ptr.5306

65. Manosroi, A.; Jantrawut, P.; Ogihara, E.; Yamamoto, A.; Fukatsu, M.; Yasukawa, K.; Tokuda, H.; Suzuki, N.; Manosroi, J.; Akihisa, T. Biological activities of phenolic compounds and triterpenoids from the galls of *Terminalia chebula*. *Chem. Biodivers*. **2013**, *10(8)*, 1448-1463. doi: 10.1002/cbdv.201300149

66. Woo, S.M.; Seo, S.U.; Min, K.-j.; Im, S.-S.; Nam, J.-O.; Chang, J.-S.; Kim, S.; Park, J.-W.; Kwon, T.K. Corosolic acid induces non-apoptotic cell death through generation of lipid reactive oxygen species production in human renal carcinoma caki cells. *Int. J. Mol. Sci*. **2018**, *19(5)*, 1309. doi: 10.3390/ijms19051309

67. Bouziane, A.; Bakchiche, B.; Dias, M.I.; Barros, L.; Ferreira, I.C.F.R.; AlSalamat, H.A.; Bardaweel, S.K. Phenolic compounds and bioactivity of *cytisus villosus* pourr. *Molecules*. **2018**, *23(8)*. doi: 10.3390/molecules23081994

68. Xu, Z.R.; Li, K.; Pan, T.W.; Liu, J.; Li, B.; Li, C.X.; Wang, S.Y.; Diao, Y.P.; Liu, X.G. Lonicerin, an anti-algE flavonoid against *Pseudomonas aeruginosa* virulence screened from Shuanghuanglian formula by molecule docking based strategy. *Journal of Ethnopharmacology*. **2019**, *239*, 111909. doi: https://doi.org/10.1016/j.jep.2019.111909

69. Gu, L.Z.; Sun, H. Lonicerin prevents inflammation and apoptosis in LPS-induced acute lung injury. *Front. Biosci-Landmrk*. **2020**, *25*, 480-497. doi: 10.2741/4815

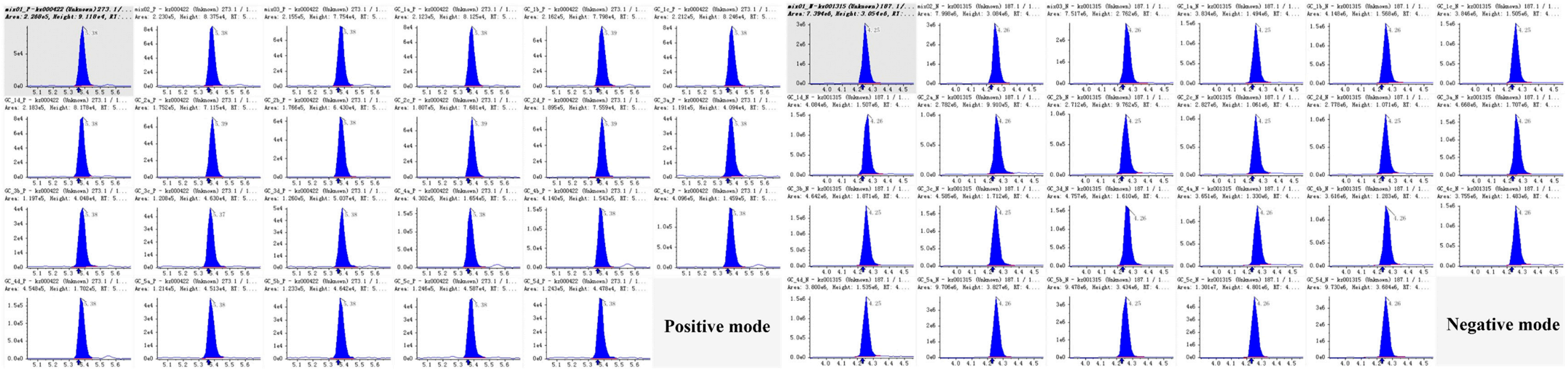

Figure S1. Integrated correction chart for metabolite quantitative analysis.

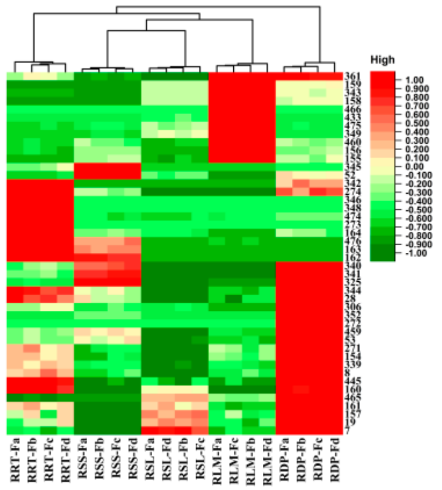

A

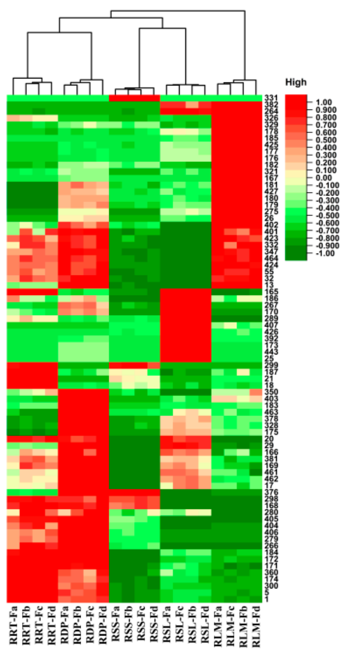

B

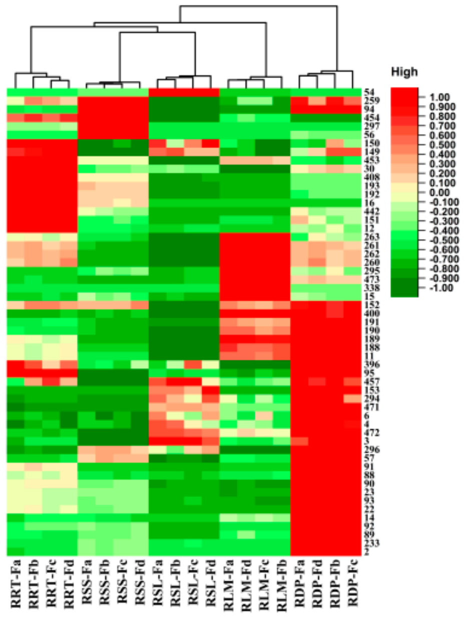

C

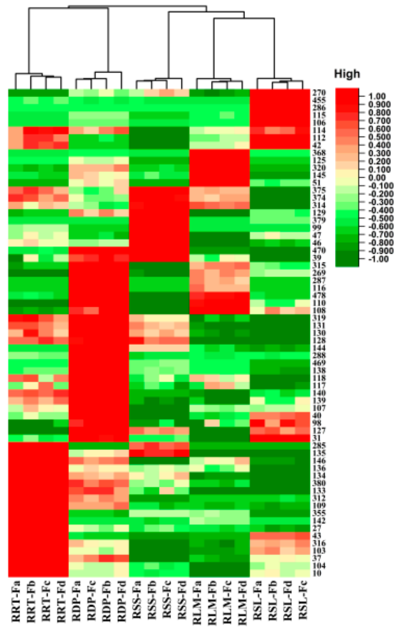

D

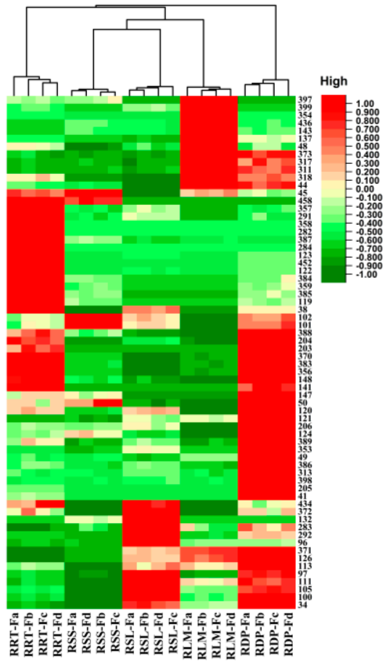

E

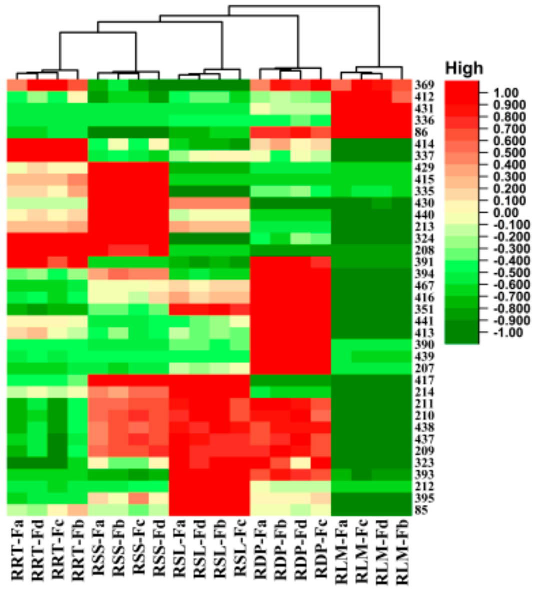

F

Figure S2. Heatmap of the relative contents of the differential metabolites of **(A)** flavones; **(B)** flavonols; **(C)** flavanones, isoflavones, anthocyanins, flavanones, proanthocyanidins and chalcones; **(D)** hydroxycyclic acid derivatives (HCs) and others phenolic acids (HAs); **(E)** hydroxybenzoic acid derivatives (HBs); **(F)** terpenoids. The numbers repre-sent the compounds in **Supplementary Table S4**.

A

B

C

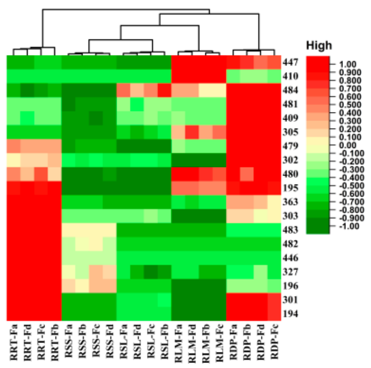

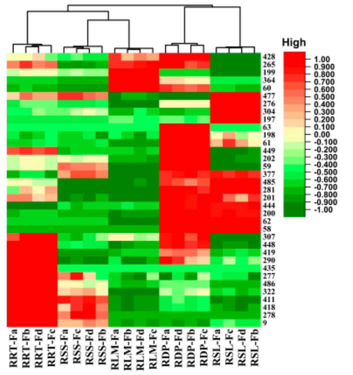

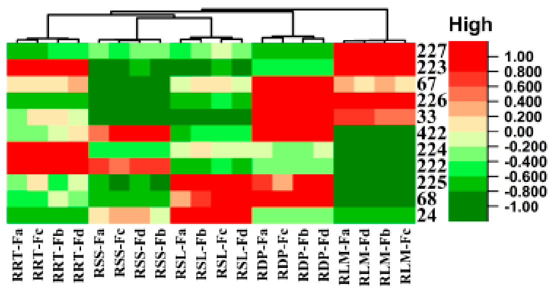

D

E

F

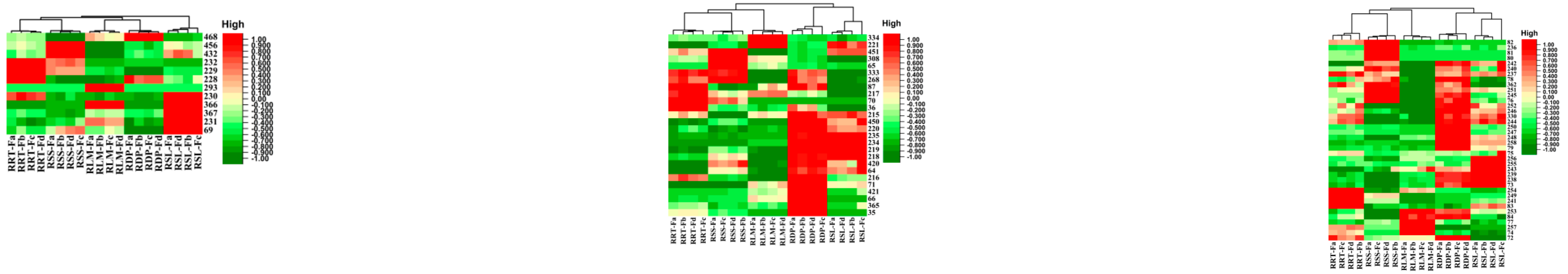

Figure S3. Heat map analysis of **(A)** lignans; **(B)** others; **(C)** vitamins; **(D)** coumarins; **(E)** alkaloids; **(F)** organic acids. The numbers represent the compounds in **Supplementary Table S4**.
